# A positive feedback between PDIA3P1 and OCT4 promotes the cancer stem cell properties of esophageal squamous cell carcinoma

**DOI:** 10.1186/s12964-024-01475-3

**Published:** 2024-01-22

**Authors:** Tao Huang, Qi You, Dengjun Huang, Yan Zhang, Zhijie He, Xuguang Shen, Fei Li, Qiang Shen, Ifeanyi Christian Onyebuchi, Chengwei Wu, Feng Liu, Shaojin Zhu

**Affiliations:** 1 Department of Thoracic Surgery, The First Affiliated Hospital of Wannan Medical College (Yijishan Hospital of Wannan Medical College), Wuhu, 241001 China; 2 Department of Gastrointestinal Surgery, The First Affiliated Hospital of Wannan Medical College (Yijishan Hospital of Wannan Medical College), Wuhu, 241001 China; 3 Department of Thoracic Surgery, Lishui Branch, Zhongda Hospital Affiliated to Southeast University, Nanjing, 211200 China

**Keywords:** PDIA3P1, OCT4, WWP2, Cancer stem cell, Ubiquitination, Esophageal squamous cell carcinoma

## Abstract

**Background:**

Increasing evidence has indicated that long non-coding RNAs (lncRNAs) have been proven to regulate esophageal cancer progression. The lncRNA protein disulfide isomerase family A member 3 pseudogene 1 (PDIA3P1) has been shown to promote cancer stem cell properties; however, its mechanism of action remains unclear. In this study, we investigated the regulation of esophageal cancer stem cell properties by the interaction of PDIA3P1 with proteins.

**Methods:**

The GEPIA2 and Gene Expression Omnibus databases were used to analyze gene expression. PDIA3P1 expression in human esophageal squamous cell carcinoma (ESCC) tissues and cell lines was detected by quantitative real-time polymerase chain reaction (qRT-PCR). Loss-of-function experiments were performed to determine the effects of PDIA3P1 on ESCC cell proliferation, migration, and invasion. The sphere formation assay, number of side population cells, and CD271 + /CD44 + cells were detected by flow cytometry to identify the cancer stem cell properties. RNA immunoprecipitation (RIP), RNA pull-down, co-immunoprecipitation (co-IP), dual luciferase reporter, and cleavage under targets and tagmentation (CUT&Tag) assays were performed to elucidate the underlying molecular mechanisms.

**Results:**

PDIA3P1 expression was upregulated in ESCC cell lines and tissues. Functionally, higher PDIA3P1 expression promoted cell proliferation, invasion, and metastasis and inhibited apoptosis in esophageal cancer. Importantly, PDIA3P1 promoted cancer stem cell properties in ESCC. Mechanistically, PDIA3P1 interacted with and stabilized octamer-binding transcription factor 4 (OCT4) by eliminating its ubiquitination by the ubiquitinating enzyme WW domain-containing protein 2 (WWP2). Moreover, as a transcription factor, OCT4 bound to the PDIA3P1 promoter and promoted its transcription.

**Conclusions:**

Our research revealed a novel mechanism by which a positive feedback loop exists between PDIA3P1 and OCT4. It also demonstrated that the PDIA3P1-WWP2-OCT4 loop is beneficial for promoting the cancer stem cell properties of ESCC. Owing to this regulatory relationship, the PDIA3P1-WWP2-OCT4-positive feedback loop might be used in the diagnosis and prognosis, as well as in the development of novel therapeutics for esophageal cancer.

**Supplementary Information:**

The online version contains supplementary material available at 10.1186/s12964-024-01475-3.

## Background

Esophageal cancer (EC) is one of the most lethal malignant tumors in the world with high mortality and morbidity [1, 2]. EC has two major pathological subtypes: esophageal adenocarcinoma (EA) and esophageal squamous cell carcinoma (ESCC). In China, the main type of EC is ESCC, which accounts for approximately 90% of EC cases and is one of the deadliest malignant tumors [3, 4]. According to previous reports, multiple risk factors lead to its occurrence and development, including smoking, alcohol consumption, obesity, and low fruit/vegetable intake [5]. Patients with ESCC have no typical symptoms in the early stage, and most are diagnosed in the late stage. Currently, the standard treatment methods for ESCC include surgery, radiation therapy, and chemotherapy [6]. Despite substantial progress in the diagnosis and treatment of ESCC, the prognosis remains poor and the overall 5-year survival rate is low [7, 8]. Therefore, elucidating the potential molecular mechanisms of progression and identifying new therapeutic targets are crucial for the development of more effective treatments for ESCC.

Long non-coding RNA (lncRNA) is a class of non-coding RNA (ncRNA) with a length of more than 200 nucleotides (nt) that usually lack coding ability. LncRNAs participate in different biological processes through different mechanisms. Currently, it is clearly recognized that lncRNAs are closely associated with cancer progression. In ESCC, lncRNAs affect cell proliferation, metastasis, and resistance to radiotherapy and chemotherapy [9–11]. LncRNAs play important roles in the regulation of ESCC stem cell properties. Small nucleolar RNA host gene 16 (SNHG16) acts as a sponge for micro (mi)-RNA-802 to upregulate protein patched homolog 1 and activate the Hedgehog pathway, thereby promoting the stem cell properties of ESCC [12]. Long intergenic non-protein coding RNA (linc-ROR) regulates the expression of SRY-box transcription factor (SOX)-9 by directly sponging multiple miRNAs to promote the acquisition of cancer stem cell (CSC)-like properties in ESCC [13].

CSCs, also known as tumor-initiating cells or side populations (SPs), are a small group of cancer cells with biological characteristics of self-renewal and differentiation. They are crucial components of the tumor microenvironment that participate in tumor initiation, progression, recurrence, metastasis, and resistance to chemotherapy [14]. A growing body of research has shown that CSCs are carcinogenic in most types of human cancers, including esophageal, breast, lung, liver, gastric, and pancreatic cancers [15, 16]. LncRNAs play important roles in CSCs during tumor progression [17]. Octamer-binding transcription factor 4 (OCT4), together with cellular (c)-MYC, Kruppel-like factor 4 (KFL4), and SOX2, is essential for inducing pluripotency in human and mouse somatic cells [18]. OCT4 has been shown to be a driver of CSC properties and has been well studied as an indispensable transcription factor for CSC self-renewal and pluripotency.

Protein disulfide isomerase family A member 3 pseudogene 1 (PDIA3P1) is an lncRNA with a length of 2099 bp, located on chromosome 1q21.1. PDIA3P1 is upregulated in various malignant tumors, including lung cancer [19], oral squamous cell carcinoma [20], liver cancer [21], glioma [22, 23] and multiple myeloma [24]. The expression of PDIA3P1 in ESCC and its role in stem cell properties have not yet been reported. Therefore, the role and potential molecular mechanism of PDIA3P1 in ESCC stem cell properties need to be further elucidated. This study aimed to investigate the regulation of ESCC properties by the interaction of PDIA3P1 with proteins.

In this study, we found that PDIA3P1 was highly expressed in both ESCC tissues and cell lines, which promoted the proliferation, invasion and metastasis of ESCC cells and inhibited apoptosis. Through cell sphere culture and flow cytometry, we found that PDIA3P1 promotes the stem cell properties of ESCC. Mechanistically, the OCT4-WWP2 complex is disrupted by the interaction of PDIA3P1 with OCT4 and stabilizes the Oct4 protein by preventing WWP2-mediated ubiquitination. In turn, OCT4, as a transcription factor, positively activates PDIA3P1 transcription by binding to its promoter. Our study demonstrates that the positive feedback loop between PDIA3P1 and OCT4 plays a key role in adjusting cancer stem cell properties of ESCC.

## Materials and methods

### Tissues collection

18 pairs of ESCC and matched normal tissue samples were collected from ESCC patients who underwent surgery from May 2023 to December 2023 in the The First Affiliated Hospital of Wannan Medical College. This study was approved by the Institutional Ethics Committee of the The First Affiliated Hospital of Wannan Medical College. Written informed consent was obtained from each subject. The methods were carried out in accordance with the approved guidelines.

### Quantitative real time RT-PCR (qRT-PCR)

Total RNA was prepared from the various treated cells using RNAprep Pure Cell Kit (TIANGEN, DP430) according to the manufacturer’s instructions. Each RNA sample was then reverse transcribed into cDNAs using RevertAid™ First Strand cDNA Synthesis Kit (Thermo Scientific, K16225). cDNA and appropriate primers were plated in a 96-well plate and gene expression levels were measured using Universal SYBR Green Fast qPCR Mix (ABclonal, RK21203) with a Bio-Rad CXF96 PCR system (Hercules, CA, USA). Each sample was repeated three times. 2^−ΔΔCt^ method was used to quantify the relative gene expression level, with β-actin as the reference gene. All qRT-PCR Primer sequences are showed in Additional file 1: Table S1.

### Cloning and DNA construction

To construct different length of PDIA3P1 promoters, fragments were amplified from genome DNA of HEEC (normal esophageal epithelial) cells by PCR and were then cloned into pGL3-Basic Vector (Promega, Madison, WI, USA) at the Kpn I and Hind III sites. Three point mutations in the PDIA3P1 promoters generated by site-specific mutagenesis were obtained from MiaoLingBio (Wuhan, China). For construction of the PDIA3P1 expression vector, the truncated fragments PDIA3P1 was amplified by PCR, digested with Kpn I/ Hind III, and inserted into the pcDNA3.1( +) expression vector (Invitrogen). All Primer sequences are showed in Additional file 2: Table S2.

### RNA pull-down

To prepare the DNA template for in vitro RNA synthesis, PDIA3P1 was subcloned into pcDNA3.1 with inserted T7 promoter before and after the cloning site. PDIA3P1 of different fragments were amplified by PCR with primers containing F2 fragments, then the PCR products were recovered and transcribed with T7 High Yield RNA Transcription Kit (R7018S, Beyotime, China). Then, the purity and size of agarose gel were tested. F2-RNA pull down kit (FI8701, Fitgene, China) was used for RNA pull-down assay. Cell lysates were firstly incubated with F2-PDIA3P1 probe. Then the ligand conjugated magnetic beads were added to cell lysates. The retrieved proteins were then analyzed by Western Blot. All primer sequences containing F2 are showed in Additional file 3: Table S3.

### RNA immunoprecipitation (RIP)

RNA immunoprecipitation kit (P0101, Geneseed, China) was leveraged for RIP assay according to the manufacturer’s instruction. Briefly, cells were harvested and lysed by RIP lysis buffer, then incubated with magnetic beads conjugated with the OCT4 antibody or the IgG. PDIA3P1 RNA levels in the precipitates were measured by qRT-PCR.

### Dual luciferase reporter assays

The various reporter constructs were co-transfected with Renilla luciferase vector into the cells. After 48 h, cells were lysed and activities of firefly luciferase and Renilla luciferase were analyzed Dual Luciferase Reporter Gene Assay Kit (11402ES60, YEASEN, China) according to the manufacturer’s instruction. All the results are presented as average value of triplicates ± SD.

### Cleavage under targets and tagmentation (CUT&Tag)

We performed the enzyme-tethering strategy known as CUT&Tag to confirm that OCT4 binds to the promoter of PDIA3P1 in vivo following the instructions for the Novo CUT&Tag High-Sensitivity Kit (N259-YH0, Novoprotein, China). Cells were harvested and washed in Wash Buffer. ConA magnetic bead-bound cells were resuspended in 50 μL precooled Primary Antibody Buffer containing the appropriate primary antibody (OCT4). IgG was used as the control antibody. The primary antibody was removed, followed by incubation with the secondary antibody. Next, cells were resuspended in Tagmentation buffer and incubated at 37 °C for 1 h. Beads were added to each tube by vortexing, and quickly spun to extract the DNA. The purified DNA was used as a template for standard PCR and qRT-PCR. The standard PCR product was run on a 2% agarose gel for visualization and qRT-PCR results were analyzed according to the protocols. The primers for CUT&Tag detection are provided in Additional file 4: Table S4.

### Statistical analysis

All experimental data are presented as the mean ± SD from three independent replicates. Statistical analysis was performed using SPSS 22.0 (IBM Corporation, Armonk, NY, USA) and GraphPad Prism 6 (La Jolla, CA, USA). Student’s t-test was used to compare the data differences between two groups. The differences of PDIA3P1 expression levels between tumor and normal specimens were evaluated by paired t test. One-way ANOVA followed by post hoc test was used to compare the data differences between three groups. *P* value < 0.05 was considered statistically significant. *P < 0.05, **P < 0.01 and ***P < 0.001. ^##^*P* < 0.01 and ^###^*P* < 0.001.

Supplementary materials and methods are described in Additional file 5.

## Results

### PDIA3P1 is highly expressed and required for malignant behaviors in ESCC cells

**Fig. 1:**
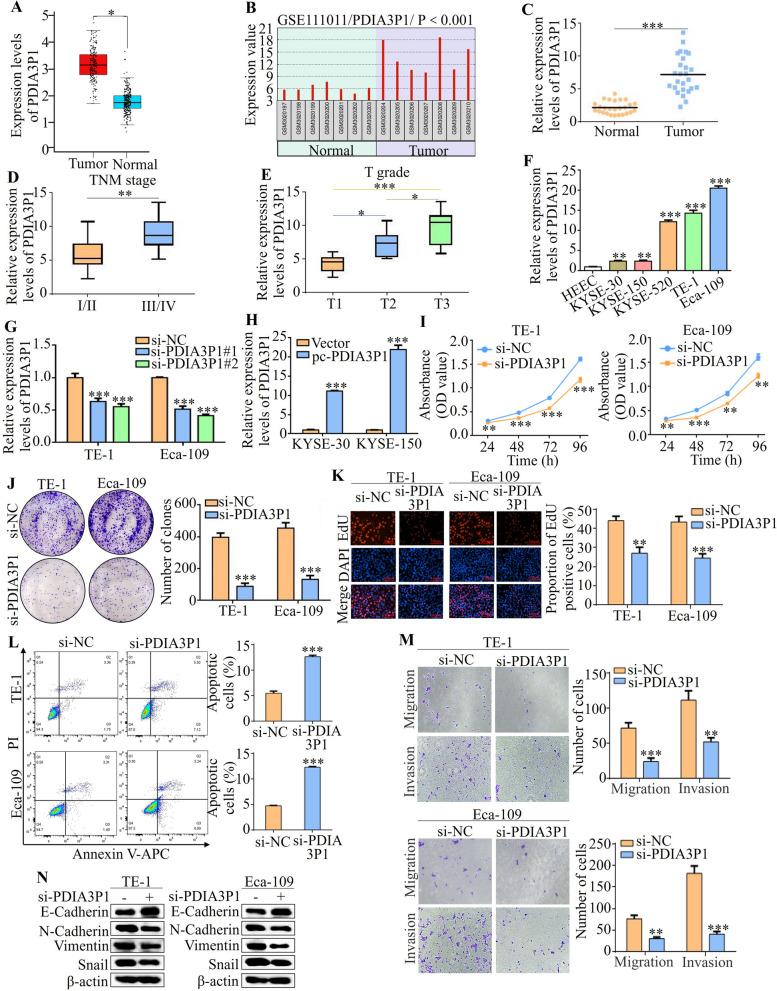
PDIA3P1 is highly expressed and required for malignant behaviors in ESCC cells. **A** PDIA3P1 expression level in esophageal cancer (*n* = 182) compared with normal tissues (*n* = 286) in GEPIA database (*p < 0.05, student t-test). **B** Analysis of PDIA3P1 gene expression in GEO datasets (GSE111011). **C** qRT-PCR was used to detect the relative expression of PDIA3P1 in 26 paired ESCC tissues and non-tumor specimens (***p < 0.001, paired t-test). **D** Expression levels of PDIA3P1 were compared between patients in the TNM stage I/II group (*n* = 15) and the TNM III/IV group (*n* = 11) (*p < 0.01, student t-test). **E** PDIA3P1 expression levels were assessed in the T1 group (*n* = 8), the T2 group (*n* = 9) and T3 group (*n* = 9) (*p < 0.05, ***p < 0.001, for difference from the T1 grade by ANOVA with Dunnett's correction for multiple comparisons). **F** Expression levels of PDIA3P1 in normal cell line HEEC and five ESCC cell lines (KYSE-30, KYSE-150, KYSE-520, TE-1 and Eca-109) were examined using qRT-PCR (**p < 0.01,***p < 0.001, for difference from the HEEC cells by ANOVA with Dunnett's correction for multiple comparisons). **G** The expression of PDIA3P1 was knocked down using two siRNAs in TE-1 and Eca-109 cells (***p < 0.001, for difference from the transfected with si-NC by ANOVA with Dunnett's correction for multiple comparisons). **H** Overexpression of PDIA3P1 by transfected expression plasmid in KYSE-30 and KYSE-150 cells (***p < 0.001, student t-test). **I** CCK-8 assay of the cell proliferation after knocking down PDIA3P1 in ESCC cells (**p < 0.01, ***p < 0.001, for difference from the transfected with si-NC by ANOVA with Dunnett's correction for multiple comparisons). **J** Colony formation assay after silencing of PDIA3P1 in TE-1 and Eca-109 cells (***p < 0.001, student t-test). **K** EdU assays were performed to assess the proliferative ability of ESCC cells with PDIA3P1 knockdown (**p < 0.01, ***p < 0.001, student t-test). **L** The apoptosis analysis of PDIA3P1 knockdown by flow cytometry (***p < 0.001, student t-test). **M** Transwell assays were conducted to examine the effects of PDIA3P1 knockdown on ESCC cells migration and invasion (**p < 0.01, ***p < 0.001, student t-test). **N** Western blot shows expression levels of E-Cadherin, N-Cadherin, Vimentin and Snail after transfection with PDIA3P1 siRNA. **F**-**M**, these data represent the mean ± S.D. of triplicates

We examined the expression of PDIA3P1 to determine whether it promotes the malignant progression of ESCC. The GEPIA 2 database showed that PDIA3P1 expression was higher in EC tissue than in normal tissue (Fig. 1A). Moreover, interrogation of the Gene Expression Omnibus (GEO) database for ESCC indicated that the PDIA3P1 level is higher in tumor tissue than in normal tissues (Fig. 1B). Next, we performed qRT-PCR and showed that PDIA3P1 expression was upregulated in ESCC tissue compared with adjacent normal tissue (Fig. 1C). Especially, PDIA3P1 expression was higher in TNM III/IV stage and increased with the depth of infiltration (T grade) (Fig. 1D, E). As indicated in Additional file 6: Table S5, high PDIA3P1 expression was associated with tumor differentiation (*P* = 0.047), TNM stage (*P* = 0.006) and T grade (*P* = 0.039). Consistently, PDIA3P1 expression was also significantly upregulated in human ESCC cell lines (KYSE-30, KYSE-150, KYSE-520, TE-1, and Eca-109) compared with normal esophageal epithelial cells (Fig. 1F). We chose TE-1 and Eca-109 cell lines to silence PDIA3P1 through small interfering (si)-RNA. Compared with the control (si-NC) group, after 48 h of transfection with si-PDIA3P1 1# and si-PDIA3P1 2#, the expression of PDIA3P1 was decreased in both cell lines (Fig. 1G). Since si-PDIA3P1 2# showed a stronger knockdown effect, si-PDIA3P1 2# was selected for the subsequent experiment. KYSE-30 and KYES-150 cell lines were transfected with forced-expression PDIA3P1 using the plasmid, and the expression of PDIA3P1 significantly increased after transfection with the PDIA3P1 plasmid (Fig. 1H).

Cell proliferation and apoptosis were assessed using Cell Counting Kit (CCK)-8 assays, colony formation assays, EdU assays, and flow cytometry in TE-1 and Eca-109 cells after PDIA3P1 knockdown. CCK-8 assays demonstrated that silencing PDIA3P1 significantly reduced the proliferative capabilities of TE-1 and Eca-109 cells (Fig. 1I). PDIA3P1 knockdown suppressed colony formation in TE-1 and Eca-109 cells (Fig. 1J). The EdU assay indicated that PDIA3P1 knockdown significantly inhibited DNA replication in ESCC cells (Fig. 1K). We further detected apoptosis levels using flow cytometry and found that silencing PDIA3P1 increased the percentage of apoptotic cells (Fig. 1L). Next, we investigated whether PDIA3P1 influences the migration and invasion of ESCC cells. Transwell assays with or without Matrigel showed that ESCC cells transfected with si-PDIA3P1 presented a markedly decreased migration and invasion abilities (Fig. 1M). Western blotting further confirmed that PDIA3P1 knockdown significantly inhibited the expressions of N-cadherin, Vimentin, and Snail and increased the expression of E-cadherin (Fig. 1N). Collectively, these results indicated that PDIA3P1 facilitates ESCC proliferation, apoptosis, migration and invasion.

### PDIA3P1 upregulates OCT4 to promote the CSC properties in ESCC cells

**Fig. 2:**
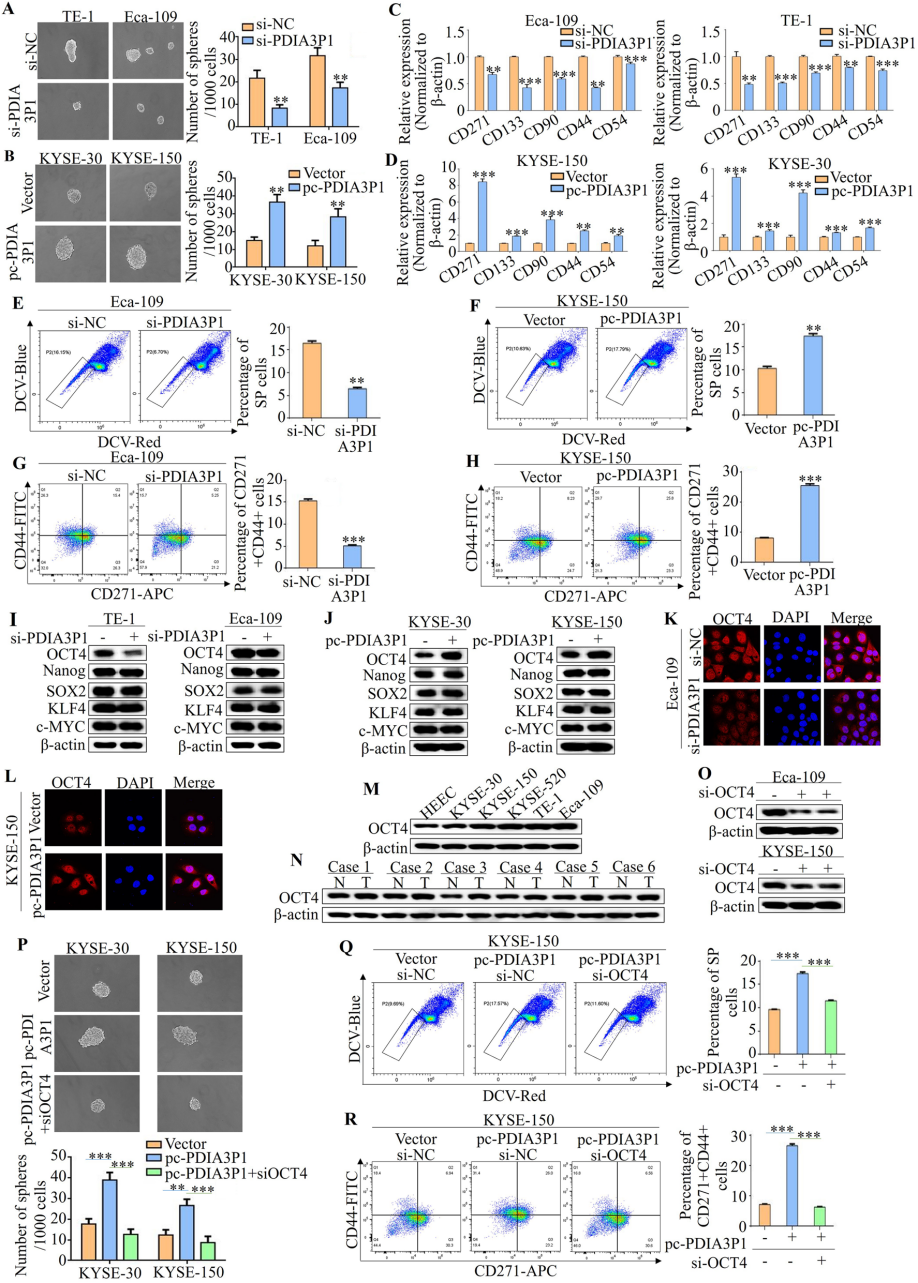
PDIA3P1 upregulates OCT4 to promote the cancer stem cell properties in ESCC cells. **A**,** B** Representative images of spheres and histogram analysis of sphere-formation rates in the indicated cells. Silencing PDIA3P1 in TE-1 and Eca-109 cells (**A**) and overexpression of PDIA3P1 in KYSE-30 and KYSE-150 cells (**B**) (**p < 0.01, student t-test). **C**,** D** qRT-PCR shows expression levels of esophageal CSCs cell surface markers after transfection with PDIA3P1 siRNA (**C**) and expression plasmid (**D**) (**p < 0.01, ***p < 0.001, student t-test). **E** Flow cytometry analysis of percentage of SP cells after PDIA3P1 knockdown (**p < 0.01, student t-test). **F** The percentage of SP cells was detected by flow cytometry after overexpression of PDIA3P1 (**p < 0.01, student t-test). **G**,** H** Flow cytometry analysis percentage of CD271 + /CD44 + cells, (**G**) PDIA3P1 was knocked down in TE-1 and Eca-109 cells, and overexpression of PDIA3P1 in KYSE-30 and KYSE-150 cells (**H**) (***p < 0.001, student t-test). **I**,** J** Expression levels of several key components of drivers of cancer stem cell properties in cells were analyzed by western blotting. **I** Silencing of PDIA3P1. **J** Over-expression of PDIA3P1. **K** Representative images of immunofluorescence staining revealing the effect of PDIA3P1 knockdown on the expression of OCT4 in Eca-109. **L** Representative images of immunofluorescence staining revealing the effect of PDIA3P1 overexpressed on the expression of OCT4 in KYSE-150. **M** Western blotting shows the levels of OCT4 in normal cell line HEEC and five ESCC cell lines (KYSE-30, KYSE-150, KYSE-520, TE-1 and Eca-109). **N** OCT4 expression detected in 6 paired ESCC tissues and non-tumor specimens by western blotting. **O** Eca-109 and KYSE-150 cell lines were transfected with siOCT4, the expression of OCT4 was analyzed by western blotting.** P-R** KYSE-150 cells were transfected with a control, or PDIA3P1 expression plasmid or the combination of PDIA3P1 expression plasmid and OCT4 siRNA. After 48 h analyzed CSCs properties. **P** Sphere-formation. **Q** Percentage of SP cells. **R** Percentage of CD271 + /CD44 + cells (**p < 0.01, ***p < 0.001, for difference from the transfected with vector and si-NC cells by ANOVA with Dunnett's correction for multiple comparisons), All, the data represent the mean ± S.D. of triplicates

We investigated whether PDIA3P1 influences esophageal CSC properties. The sphere formation assay is an effective method for evaluating the cell's self-renewal capacity, which is a characteristic of CSCs. As examined by the tumor sphere culture, the siRNA-induced downregulation of PDIA3P1 significantly impaired sphere formation and reduced the sphere size of TE-1 and Eca-109 cells (Fig. 2A). Overexpression of PDIA3P1 accelerated sphere formation and increased the sphere size in KYSE-30 and KYSE-150 cells (Fig. 2B). Previous studies have reported that CD271, CD133, CD90, CD44 and CD54 are esophageal CSC surface markers [25]. Strikingly, qRT-PCR analysis confirmed that knockdown of PDIA3P1 markedly reduced expression and upregulated these genes in PDIA3P1‐overexpressing cells (Fig. 2C, D). SP cells are a marker for CSCs. To examine the effect of PDIA3P1 on SP cells, ESCC cells were transfected with the PDIA3P1 plasmid or siRNA, and the percentage of SP cells was determined. Figure 2E shows that silencing PDIA3P1 reduced the percentage of SP cells, whereas overexpression of PDIA3P1 increased the percentage of SP cells (Fig. 2F). It has been shown that detection of stem cell membrane surface markers CD271 and CD44 by flow cytometry can reflect CSC properties in EC [26, 27]. Meanwhile, as shown in Fig. 2C and D, PDIA3P1 could regulate CD271 and CD44 expression. Therefore, we selected CD271 + CD44 + cells as stem cells for flow cytometry analysis. Flow cytometric analysis indicated that the percentage of CD271 + CD44 + cells in the siPDIA3P1 group was remarkably lower than that in the si-NC group (Fig. 2G). ESCC cells overexpressing PDIA3P1 showed a higher percentage of CD271 + CD44 + cells (Fig. 2H). These data suggest that PDIA3P1 promotes the CSC properties of EC.

Next, we explored the mechanism by which PDIA3P1 promotes esophageal CSC properties. First, we detected the expression of the drivers of CSC properties (OCT4, Nanog, SOX2, KLF4, and c-MYC) using Western blotting. The results showed that only the expression of OCT4 was downregulated after silencing of PDIA3P1 and that the expression of OCT4 was upregulated after transfection with the PDIA3P1 plasmid (Fig. 2I, J). In Eca-109 and KYSE-150 cells, the same conclusion was verified by IF assays (Fig. 2K, L). Additionally, we detected a higher expression of OCT4 in ESCC cell lines and tissues (Fig. 2M, N). To confirm whether the CSC properties promoted by PDIA3P1 depend on OCT4, we performed simultaneous transfection of the PDIA3P1 plasmid and OCT4 siRNA. Compared with cells transfected with PDIA3P1 plasmid alone, the sphere formation and size, percentage of SP cells, and percentage of CD271 + CD44 + cells were reduced after simultaneous knockdown of OCT4 in EC cells (Fig. 2O–R). Collectively, these findings showed that PDIA3P1 regulates CSC properties by promoting OCT4 expression in ESCC cells.

### PDIA3P1 represses OCT4 degradation via the ubiquitin–proteasome pathway

**Fig. 3:**
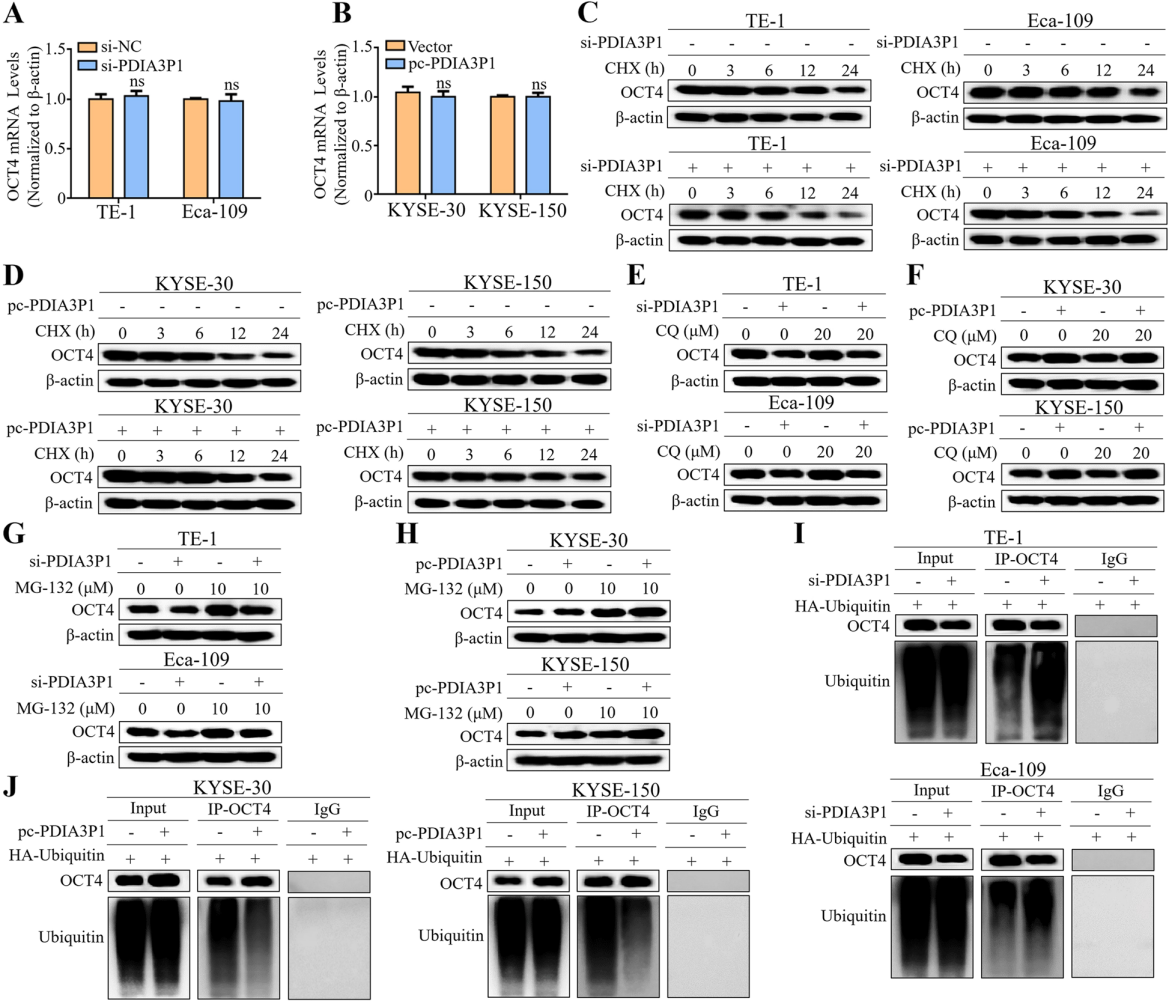
PDIA3P1 represses OCT4 degradation via the ubiquitin–proteasome pathway. **A**,** B** qRT-PCR analyses of OCT4 mRNA levels in cells with transfection with PDIA3P1-siRNA or PDIA3P1 expression plasmid (ns, no significance, student t-test, the data represent the mean ± S.D. of triplicates). **C**,** D** Western blot analysis of OCT4 protein stability using CHX (200 µg/mL) when PDIA3P1 was knocked down (**C**) or PDIA3P1 was overexpressed (**D**). **E**,** F** Western blot analysis of OCT4 in cells transfected with PDIA3P1 siRNA or expression plasmid in the presence of autophagy lysosomes inhibitor Chloroquine (CQ, 20 µM). **G** Treatment of the PDIA3P1-knockdown TE-1 and Eca-109 cells with the 26S protostome inhibitor MG132 (10 µM), western blot analysis expression of OCT4. **H** PDIA3P1 transduced KYSE-30 and KYSE-150 cells were treated with MG-132 (10 µM) and then the OCT4 expression level was examined by western blotting. **I**,** J** Western blot of the ubiquitination of OCT4 in cells that silenced PDIA3P1 or overexpressed PDIA3P1. IgG was used as a negative control

Since the ability of PDIA3P1 to promote CSC properties requires OCT4 upregulation, we investigated the molecular mechanism by which PDIA3P1 regulates OCT4 expression in EC cells. We first measured the messenger (m)-RNA levels of OCT4, and the qRT-PCR results showed that PDIA3P1 had no effect on OCT4 mRNA levels (Fig. 3A, B). Therefore, we speculated that PDIA3P1 regulates the protein stability of OCT4. To verify this, we next examined the half-life of OCT4 in ESCC cells treated with the protein synthesis inhibitor cycloheximide (CHX, 200 µg/mL). The half-life of OCT4 was significantly shortened following PDIA3P1 knockdown in TE-1 and Eca-109 cells (Fig. 3C). Conversely, treatment with CHX led to a longer OCT4 protein in PDIA3P1- overexpression cells (Fig. 3D). We then assessed the role of two critical protein degradation pathways: the proteasomal and autophagic degradation pathways [28]. The results showed that the autophagy inhibitor Chloroquine (CQ, 20 µM) had no effect on PDIA3P1-regulated OCT4 expression (Fig. 3E, F). In contrast, after treatment with the 26S protostome inhibitor MG-132 (10 µM) in TE-1 and Eca-109 cells, we found that the proteasomal degradation of OCT4 enhanced by PDIA3P1-siRNA was blocked by MG-132 (Fig. 3G). Moreover, MG-132 further increased OCT4 expression in PDIA3P1 overexpression cells (Fig. 3H).

On the basis of these results, we believe that the PDIA3P1-mediated ubiquitin–proteasome pathway may be involved in OCT4 stabilization. After endogenous OCT4 was immunoprecipitated in cells transfected with HA-Ubiquitin, strikingly increased ubiquitin signals of OCT4 were detected in PDIA3P1-siRNA cells (Fig. 3I). In line with this, the ubiquitination of OCT4 was attenuated in the transfected PDIA3P1 plasmid cells compared with that in the control cells (Fig. 3J). Taken together, these results indicate that PDIA3P1 upregulates OCT4 via the ubiquitin–proteasome pathway.

### PDIA3P1 interacted with OCT4 in ESCC cells

**Fig. 4:**
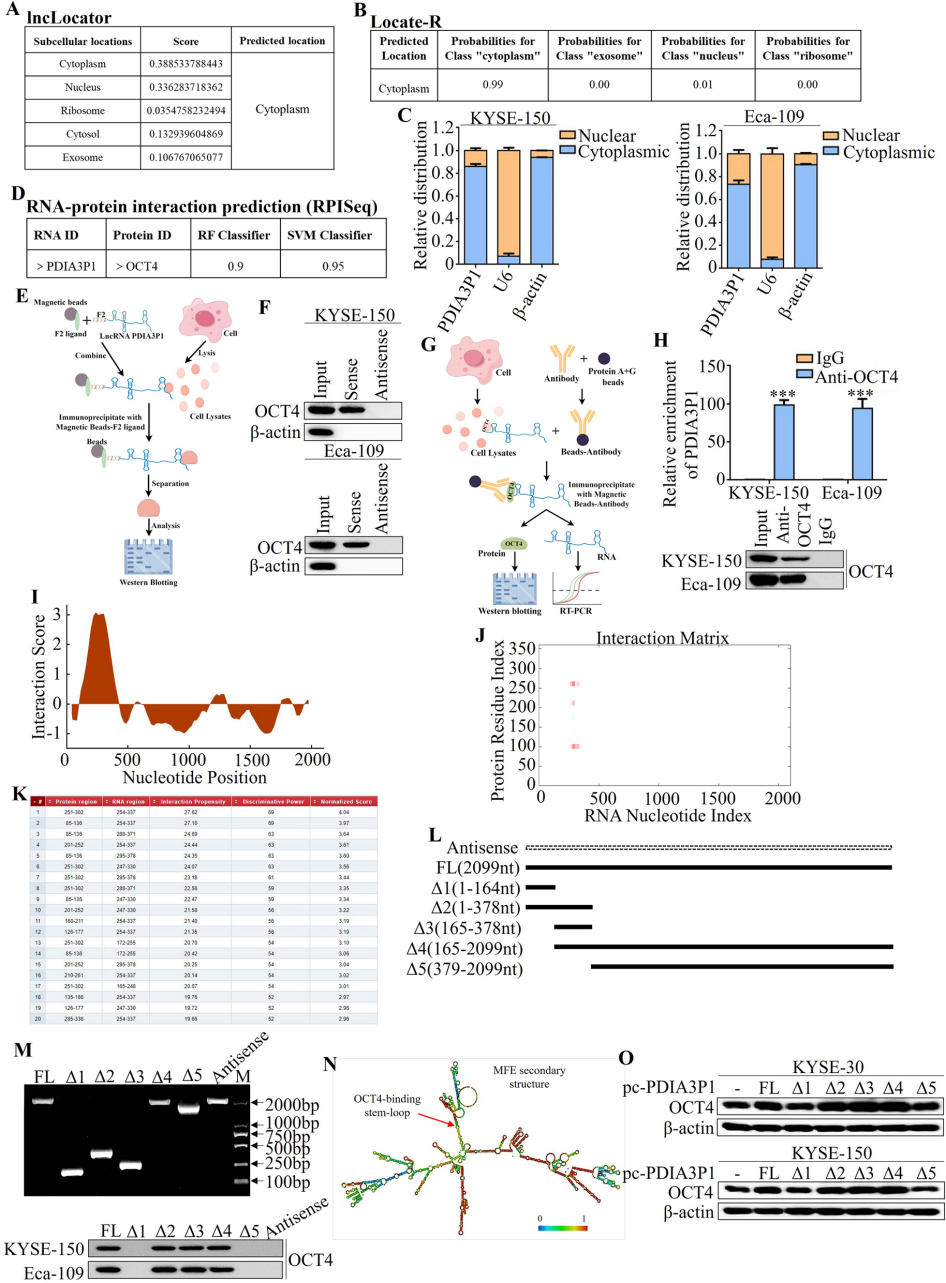
PDIA3P1 interacts with OCT4 in ESCC cells. **A**,** B** Online prediction of PDIA3P1 localization by lncLocator (**A**) and Locate-R (**B**). **C** Levels of PDIA3P1 from the nuclear and cytoplasmic fractions of KYSE-150 and Eca-109 cells were evaluated using qRT-PCR. β-actin and U6 were used as positive control for the cytoplasmic and nuclear fraction, respectively. **D** Online prediction of PDIA3P1 interacts with OCT4 by RPISeq. **E** Schematic representation of RNA-pulldown. **F** PDIA3P1 pull-down followed by Western blot validated its interaction with OCT4. **G** Schematic representation of RNA immunoprecipitation (RIP). **H** A RIP assay was performed using the OCT4 antibody in KYSE-150 and Eca-109 cells (***p < 0.001, student t-test), the data represent the mean ± S.D. of triplicates. **I**‐**K** The catRAPID was used to predict the interaction region between PDIA3P1 and OCT4. **L** Schematic diagrams of PDIA3P1 full-length and truncated fragments. **M** Truncated PDIA3P1 mapping of OCT4 binding domain. Top panel: RNA sizes of in vitro transcribed full-length and truncations of PDIA3P1. Bottom panel: Western blot of OCT4 pulled down by various PDIA3P1 fragments. **N** Prediction of PDIA3P1 structure was based on minimum free energy (MFE) and partition function. A red arrow indicates the OCT4-binding stem-loop structure. **O** The expression of OCT4 was detected by Western blot after transfected PDIA3P1 full-length or truncated fragments in KYSE-30 and KYSE-150 cells

Next, we investigated the mechanism by which PDIA3P1 regulates the ubiquitination of OCT4. First, two online databases, lncLocator (http://www.csbio.sjtu.edu.cn/bioinf/lncLocator/) and Locate-R (http://locater.azurewebsites.net/), were searched for online predictions; both of which indicated that PDIA3P1 was mainly located in the cytoplasm (Fig. 4A, B). Subcellular fractionation and qRT-PCR were used to determine PDIA3P1 distribution in ESCC cells. Similar to a study in glioma cells [22], the results showed that PDIA3P1 was mainly localized in the cytoplasm of KYSE-150 and Eca-109 cells (Fig. 4C). Accumulating evidence has suggested that the binding of proteins to lncRNAs plays a role in RNA-binding proteins and can participate in protein ubiquitination [29, 30]. RPISeq, a family of classifiers for predicting RNA–protein interactions using only sequence information, was predicted between PDIA3P1 and OCT4 interaction probabilities, and the results showed that the probabilities of 0.9 to RF Classifier and 0.95 to SVM Classifier (probabilities > 0.5 were considered “positive”) (Fig. 4D). We performed RNA pull-down assays and subsequent western blot analyses to verify that OCT4 is a PDIA3P1-binding protein in KYSE-150 and Eca-109 cells (Fig. 4E, F). Importantly, and qRT-PCR assays indicated that there was significant enrichment of PDIA3P1 in RNA–protein complexes precipitated with the anti-OCT4 antibody compared with the IgG control (Fig. 4G, H).

To identify the unique binding sites, the detailed location of the interaction between PDIA3P1 and OCT4 was predicted using catRAPID (Fig. 4I–K). We used a series of deletion mutants of PDIA3P1 to map the OCT4 binding region (Fig. 4L). RNA pull-down results showed nucleotides 165–378 of PDIA3P1 mutants Δ2, Δ3 and Δ4 bound to OCT4 as efficiently as full-length PDIA3P1, and elucidated that the middle region of PDIA3P1 RNA (nucleotides 165–378) is required for the interaction between PDIA3P1 and OCT4 (Fig. 4M). Prediction of the PDIA3P1 structure was based on the minimum free energy (MFE) and partition function (http://rna.tbi.univie.ac.at/). The data in Fig. 4N suggest that the OCT4 binding stem-loop structure is the 165–378 nt fragment of PDIA3P1. Interestingly, western blot analysis showed that overexpression of PDIA3P1 mutant-Δ2, Δ3, and Δ4 promoted OCT4 expression as efficiently as full-length PDIA3P1 in both KYSE-30 and KYSE-150 cells (Fig. 4O). Collectively, our results indicated that PDIA3P1 directly binds to OCT4.

### PDIA3P1 disrupts the interaction between OCT4 and the E3 ligase WWP2

**Fig. 5:**
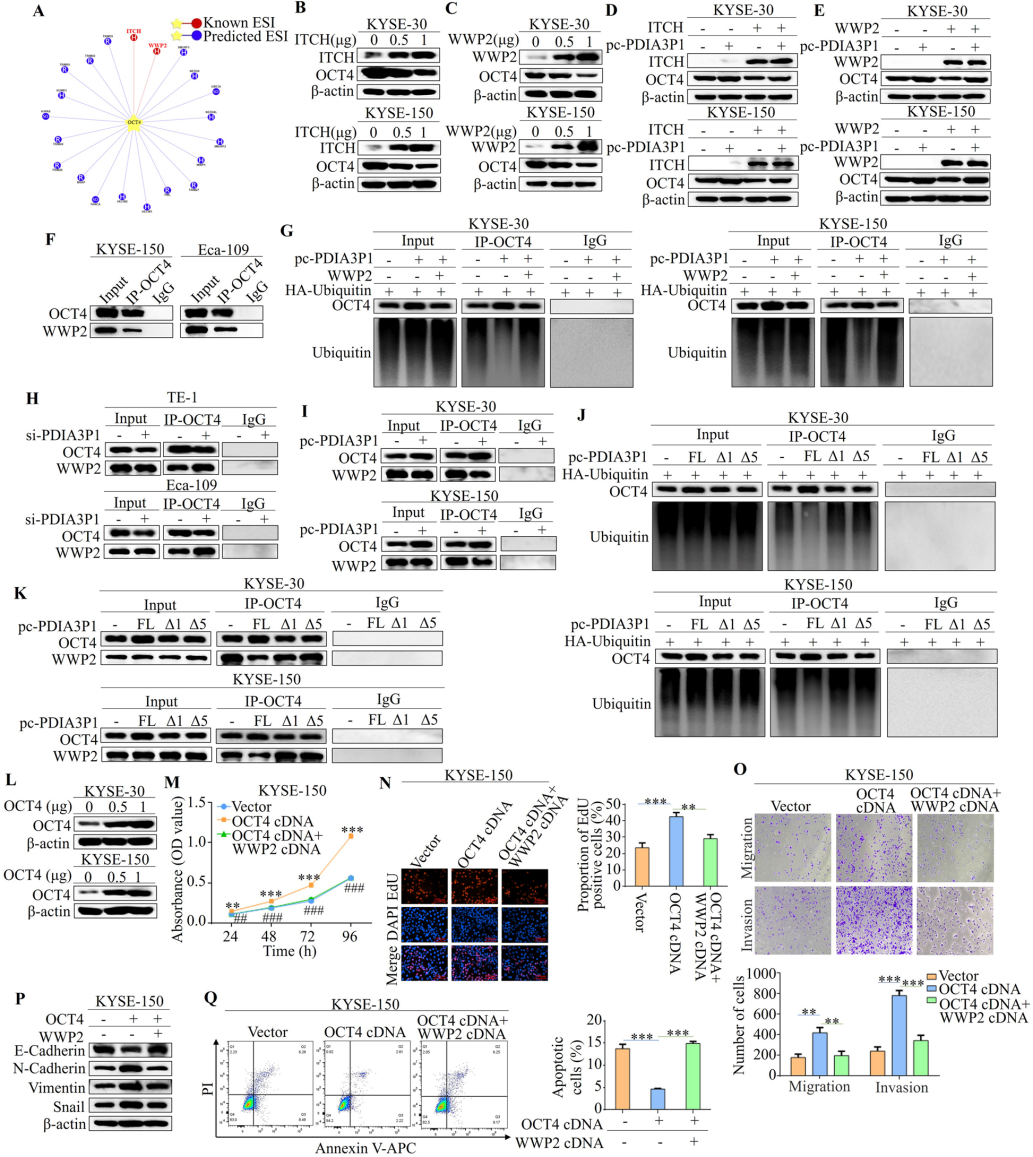
PDIA3P1 disrupts the interaction between OCT4 and the E3 ligase WWP2. **A** UbiBrowser 2.0 was used to analyze the E3 ligase that interacts with OCT4. **B** After cells were transfected with ITCH cDNA, western blot showed OCT4 protein decreased in a dose‐dependent way. **C** Overexpressing of WWP2 significantly suppressed OCT4 protein expression in esophageal cancer cells. **D, E** Cells were transfected with a control, or PDIA3P1 expression plasmid or the combination of PDIA3P1 expression plasmid and ITCH cDNA (**D**) or WWP2 cDNA (**E**). OCT4 levels were detected by western blot. **F** Interactions between OCT4 and WWP2 in ESCC cells were verified via Co-IP assays. **G** OCT4 was immunoprecipitated and immunoblotted with the indicated antibodies in KYSE-30 and KYSE-150 cells cotransfected with PDIA3P1 and WWP2. **H** co-IP with anti-OCT4 antibody for detecting the interaction of WWP2 in the PDIA3P1 silenced cells. **I** The effect of PDIA3P1 overexpression on the interaction between OCT4 and WWP2 was determined by co-immunoprecipitation assay in KYSE-30 and KYSE-150 cells. **J** Cells transfected with full-length or mutants Δ1 or Δ5 of PDIA3P1, respectively in ESCC. Immunoprecipitated and western blot of the ubiquitination of OCT4. **K** The effect of full-length or mutants Δ1 or Δ5 of PDIA3P1, respectively overexpression on the interaction between OCT4 and WWP2 was determined by co-immunoprecipitation assay in KYSE-30 and KYSE-150 cells. **L** KYSE-30 and KYSE-150 cell lines were transfected with OCT4 cDNA, the expression of OCT4 was analyzed by western blotting. **M** CCK-8 assay of the cell proliferation after simultaneous overexpression of OCT4 and WWP2 in ESCC cells (* for difference between transfected vector and OCT4 cDNA; ^#^ for difference between transfected OCT4 cDNA and co-transfected OCT4 cDNA and WWP2 cDNA). **N** EdU assays were performed to assess the proliferative ability of ESCC cells with simultaneous overexpression of OCT4 and WWP2. **O**, **P** KYSE-150 cells co-transfected with OCT4 cDNA and WWP2 cDNA, transwell assays were conducted to examine the effects of cell migration and invasion (**O**), and western blot shows expression levels of E-Cadherin, N-Cadherin, Vimentin and Snail (**P**). **Q** The apoptosis analysis of OCT4 and WWP2 simultaneous overexpression by flow cytometry in KYSE-150 cells. **M**-**O** and** Q**, all datas represent the mean ± S.D. of triplicates, for difference from the transfected with vector cells by ANOVA with Dunnett's correction for multiple comparisons. **p < 0.01, ***p < 0.001; ^##^*p* < 0.01, ^###^*p* < 0.001

To reveal how PDIA3P1 retards the ubiquitin–proteasome degradation of OCT4, we analyzed the E3 ligase that interacts with OCT4 using a computational predictive system, UbiBrowser (http://ubibrowser.bio-it.cn/ubibrowser_v3). As shown in Fig. 5A, OCT4 interacted with two known E3 ligases. Our results showing that graded overexpression of the two known E3 ligases diminished endogenous OCT4 levels in a dose-dependent manner also confirmed that ITCH and WWP2 are E3 ligases of OCT4 (Fig. 5B, C). 3–proteasome degradation of OCT4 via one or both E3 ligases in ITCH and WWP2. We also evaluated the effects of combining PDIA3P1 with ITCH or WWP2 on OCT4 expression. Notably, ITCH overexpression partially reversed the increase in OCT4 expression induced by PDIA3P1 overexpression (Fig. 5D). However, WWP2 overexpression did not exert this effect (Fig. 5E). Importantly, Co-IP assays showed that endogenous WWP2 could be precipitated with OCT4 in ESCC cells (Fig. 5F). Furthermore, overexpression of WWP2 by transfection cDNA reversed the PDIA3P1-reduced ubiquitination of OCT4 (Fig. 5G). These results indicated that PDIA3P1 may hinder the binding of WWP2 to OCT4. To further confirm this conjecture, a co-IP analysis was performed. These results suggested that more WWP2 protein could be precipitated with OCT4 in PDIA3P1-siRNA cells than in control cells (Fig. 5H). In contrast, less WWP2 protein was precipitated with OCT4 in the overexpression of PDIA3P1 cells compared with the control ESCC cells (Fig. 5I). Meanwhile, full-length PDIA3P1 or mutants Δ1 or Δ5 (excluding nucleotides 165–378) were transfected intracellularly, respectively. As shown in Fig. 5J, mutants Δ1 and Δ5 did not alter the level of ubiquitination of OCT4. Moreover, the precipitation of WWP2 protein with OCT4 was unchanged after overexpression of PDIA3P1 mutants Δ1 and Δ5 (Fig. 5K). Taken together, these findings suggest that PDIA3P1 promotes OCT4 stabilization by disrupting the OCT4-WWP2 complex in EC.

Next, we will explore the impact of the OCT4’s interaction with WWP2 on the progression of ESCC. CCK-8 assays and EdU assays showed that overexpression of OCT4 promoted cell proliferation, whereas OCT4-promoted cell proliferation was inhibited when WWP2 was also overexpressed (Fig. 5L-N). Transwell assays also revealed that WWP2 inhibited OCT4-promoted cell metastasis and invasion (Fig. 5O, P). Finally, we detected apoptosis levels using flow cytometry and found that overexpression WWP2 increased the percentage of apoptotic cells (Fig. 5Q). The above results indicated that the OCT4-promoted ESCC progression was reversed when both OCT4 and WWP2 were overexpressed in the cells.

### PDIA3P1 is a direct transcriptional target of OCT4

**Fig. 6:**
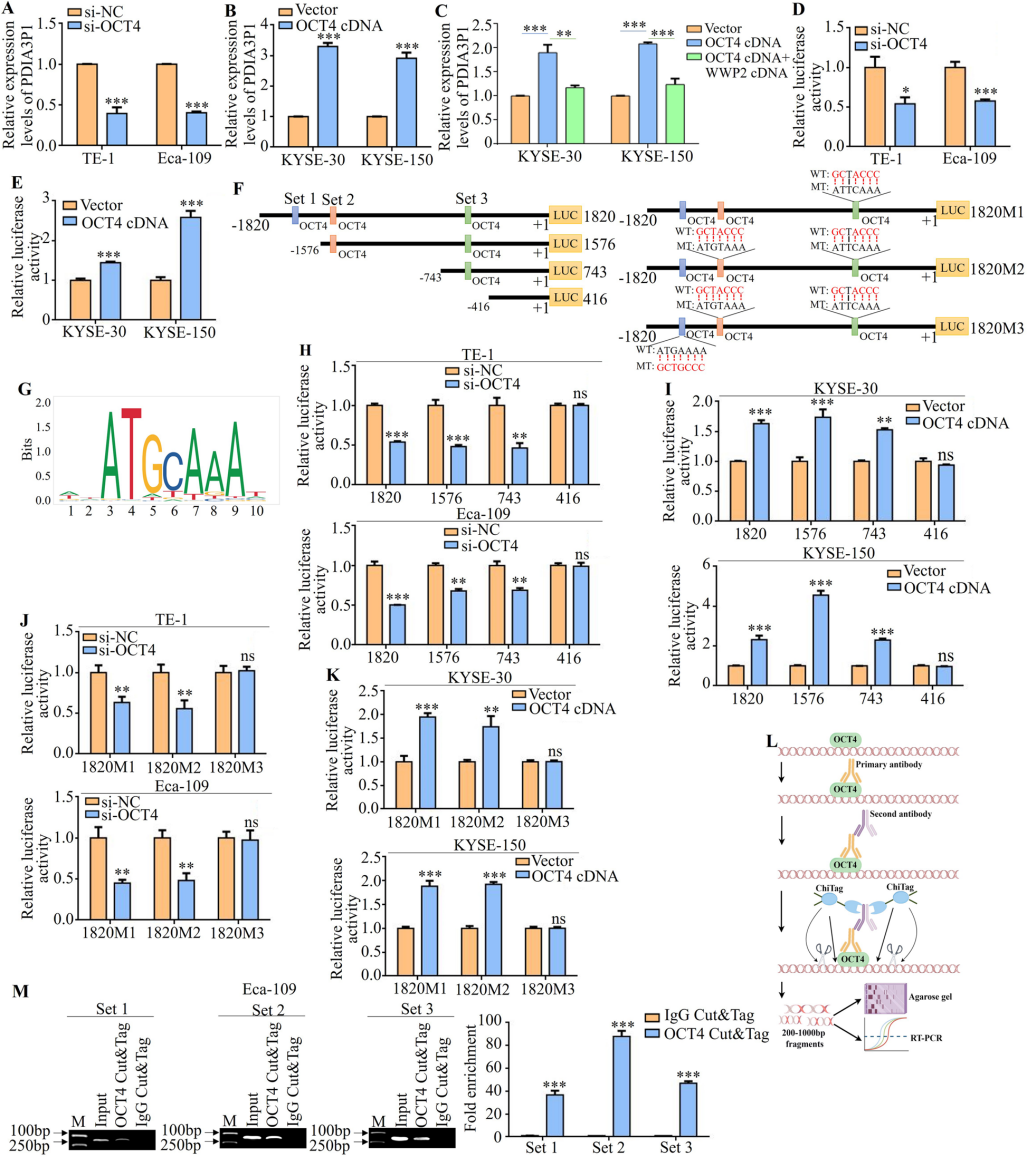
PDIA3P1 is a direct transcriptional target of the OCT4. **A** PDIA3P1 mRNA was measured by qRT‐PCR in cells with transfected OCT4 siRNA (***p < 0.001, student t-test). **B** PDIA3P1 mRNA was measured by qRT‐PCR in cells with transfected OCT4 Cdna (***p < 0.001, student t-test).**C** PDIA3P1 mRNA was measured by qRT‐PCR in cells with co-transfected with OCT4 cDNA and WWP2 cDNA (**p < 0.01,***p < 0.001, two-sided Student’s t-test). **D**,** E** PDIA3P1 promoter luciferase activities were measured after silencing of OCT4 (**D**) or overexpressed OCT4 (**E**) (***p < 0.001, student t-test). **F** Schematic representation of four different lengths of the PDIA3P1 promoter normal construct and three OCT4 mutant constructs. **G** The recognition motif of OCT4 obtained from the JASPAR. **H**, **I** Cells were transiently cotransfected with OCT4 siRNA (**H**) or OCT4 cDNA (**I**), four different lengths of the PDIA3P1 promoter luciferase reporter, and luciferase activity was determined (ns, no significance; **p < 0.01, ***p < 0.001, student t-test). **J**,** K** Cells were transiently cotransfected with OCT4 siRNA (**J**) or OCT4 cDNA (**K**), three mutant OCT4 binding site constructs of PDIA3P1 promoter luciferase reporter, and luciferase activity was determined (ns, no significance; **p < 0.01, ***p < 0.001, student t-test). **L** Schematic representation of Cleavage under targets and tagmentation (CUT&Tag). **M** Eca-109 cells were subjected to CUT&Tag assays by using anti‐OCT4 antibodies or control IgG. (Left panel) Standard PCR products were run and scanned. (Right panel) qRT‐PCR results were quantified and are indicated (***p < 0.001, student t-test). All, the datas represent the mean ± S.D. of triplicates

The mechanism of high expression of PDIA3P1 in esophageal cancer is still unclear. Interestingly, as a transcription factor, OCT4 was detected as a potential binding site in the promoter sequence of PDIA3P1 using bioinformatic analysis. OCT4 knockdown reduced PDIA3P1 mRNA levels (Fig. 6A). We also observed a strong induction of PDIA3P1 expression upon transfection with OCT4 cDNA (Fig. 6B). However, the OCT4-induced PDIA3P1 expression was suppressed after simultaneous overexpression of WWP2 (Fig. 6C). Therefore, we generated a luciferase reporter plasmid harboring WT promoter PDIA3P1. Strikingly, silencing OCT4 reduces PDIA3P1 promoter luciferase activity in both TE-1 and Eca-109 cells (Fig. 6D). The promoter activity of PDIA3P1 was markedly induced by OCT4 cDNA transfection in both KYSE-30 and KYSE-150 cells (Fig. 6E), indicating that OCT4 transcriptionally regulated PDIA3P1 expression.

We constructed four fragments of luciferase reporter plasmids of different lengths on the basis of the OCT4 binding motif (Fig. 6F, G). The four luciferase reporter plasmids were transfected into ESCC cells, and OCT4 had no significant regulatory effect on the luciferase activity of PDIA3P1, which does not contain an OCT4 binding site (Fig. 6 H, I). To specifically address whether OCT4 directly regulates PDIA3P1 transcription, a set of mutation constructs of the PDIA3P1 promoter luciferase reporter plasmids containing various predicted OCT4 binding sites was generated (Fig. 6G). Surprisingly, the activity of the PDIA3P1 (1820M1) promoter was responsive to OCT4 when the last OCT4 putative site was mutated. The promoter activity was still responsive to OCT4 when the last and penultimate sites were mutated (1820 M2) (Fig. 6J, K). However, mutation of the three binding sites (1820M3) abrogated OCT4‐regulation expression of the PDIA3P1 promoter reporter in both ESCC cells (Fig. 6J, K). Furthermore, cleavage under targets and tagmentation (Fig. 6L) performed on Eca-109 cells further confirmed that OCT4 bound to three binding sites in the PDIA3P1 promoter in vivo (Fig. 6M). In summary, our data suggest that OCT4 induces the upregulation of PDIA3P1 and directly binds to and initiates PDIA3P1 transcription. These observations support a model in which PDIA3P1 promotes OCT4 expression, and OCT4 induces PDIA3P1 promoter activity to constitute a positive feedback mechanism in the adjustment of stem cell properties of ESCC.

## Discussion

**Fig. 7:**
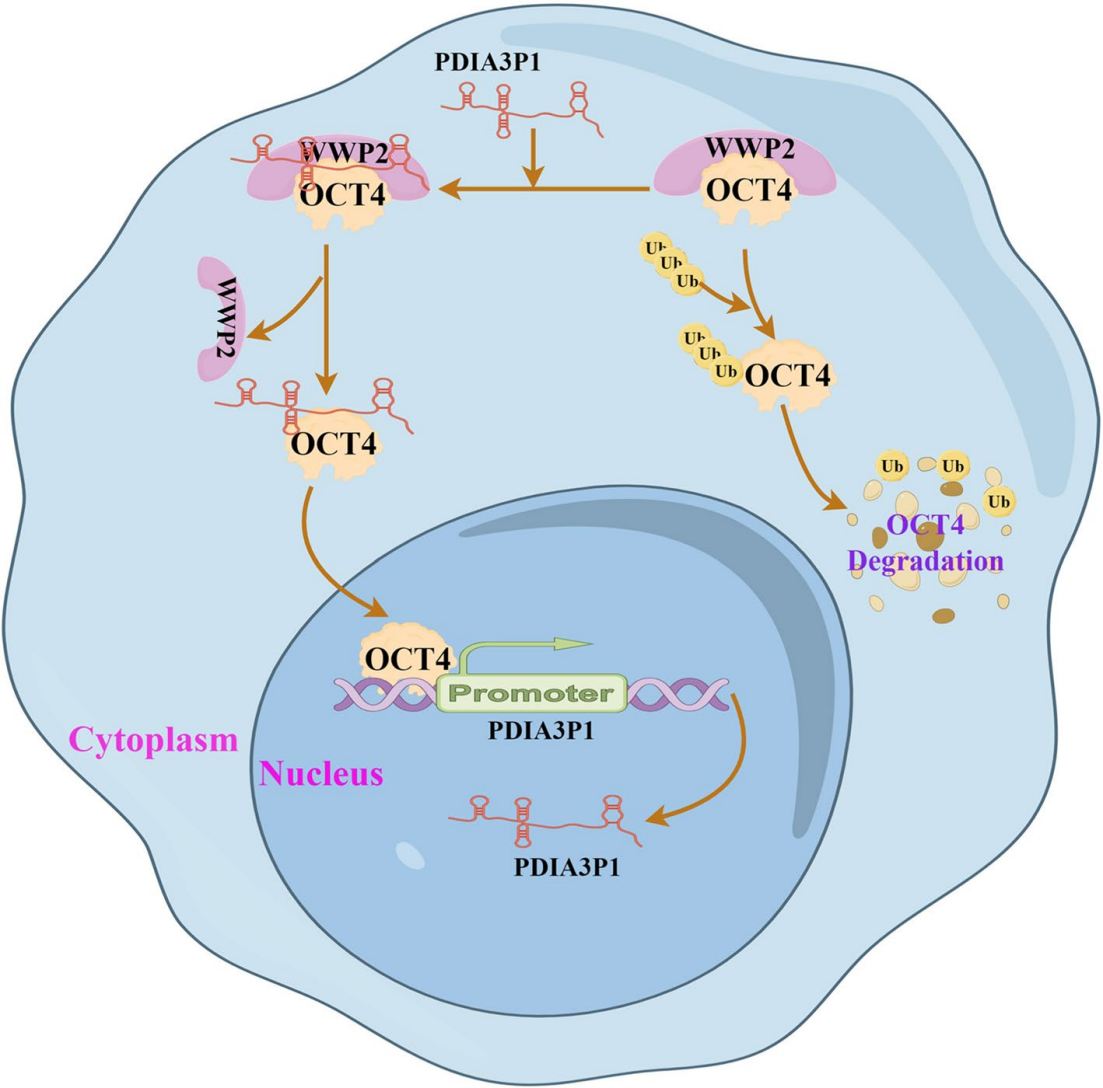
Schematic representation of PDIA3P1 and OCT4 feedback loop promotes the cancer stem cell properties of ESCC

EC is one of the most common aggressive digestive cancers worldwide [31]. LncRNAs, such as oncogenes or tumor suppressor genes, have been established as crucial regulators of pathogenesis, especially in malignancies [32]. In the present study, we used The Cancer Genome Atlas and GEO data to determine PDIA3P1 expression, which is significantly upregulated in EC. We then confirmed that PDIA3P1 is highly expressed in ESCC tissues and cell lines by qRT-PCR and confirmed its essential role in supporting the malignant phenotype of ESCC cells. Through loss-of-function experiments, we demonstrated that PDIA3P1 promotes proliferation, migration, and invasion and inhibits the apoptosis of ESCC cells in vitro. Previous studies have shown that PDIA3P1 plays a crucial role in regulating the CSC properties [21]. Further experiments indicated that PDIA3P1 promotes the CSC properties of ESCC. However, the underlying mechanisms remain unexplored. Here, we describe a novel mechanism by which PDIA3P1 promotes esophageal CSC properties: PDIA3P1 inhibits WWP2‐mediated ubiquitination and degradation, leading to OCT4 upregulation, and OCT4 promotes the transcription of PDIA3P1. This series of experiments provides novel insights into the stem cell-modulating role of PDIA3P1 in ESCC (Fig. 7).

An lncRNA transcript of PDIA3 pseudogene, PDIA3P1 was upregulated in various tumors and associated with malignant behavior. Previous studies have found that PDIA3P1 is highly expressed in hepatocellular carcinoma and regulates nuclear factor kappa light chain enhancer of activated B cell signaling and chemotherapy resistance through PDIA3P1-miR-125/124-TRAF6 [33]. Another study showed that PDIA3P1 promotes cancer cell proliferation and inhibits apoptosis by inhibiting the p53 pathway in hepatocellular carcinoma [23]. However, PDIA3P1 expression has not yet been reported in EC. Here, we found through qRT-PCR that PDIA3P1 is highly expressed in ESCC, and functional experiments demonstrated that high PDIA3P1 levels correlate with malignant behavior in EC. Stemness maintenance is an important regulatory property that determines the biological characteristics (metastasis, recurrence, and chemoresistance [34]) of malignant tumor cells, and the underlying molecular mechanisms remain unsolved. LncRNAs regulate the properties of human esophageal CSCs. For instance, FMR1-AS1 [35], SNHG16 [12], SNHG12 [36], SOX2OT [37], and Linc-ROR [13] contribute to the CSCs' properties of EC through various mechanisms. We identified the role of PDIA3P1 in promoting stem cell properties in ESCC through cell sphere formation and flow cytometry analysis of SP and CD271 + CD44 + cells. Our results provide direct evidence for the regulation of esophageal CSC properties by PDIA3P1.

There is increasing interest in OCT4 as an indispensable transcription factor that controls self-renewal and pluripotency in CSCs. OCT4 is similar to the core transcription factors SOX2 and Nanog expressed by pluripotent cells of the mammalian epiblast, which define and maintain the pluripotent state [38]. High OCT4 expression was associated with a higher histological grade in esophageal squamous cancer (*p* < 0.001) [39]. Increasing evidence has established that regulation of OCT4 expression plays a crucial role in the regulation of CSC properties in many human malignancies. Opa interacting protein 5-antisense RNA 1 (OIP5-AS1) promotes the stemness of lung cancer cells by enhancing OCT4 mRNA stability [40]. The results demonstrated that lncRNA OIP5-AS1 can confer lung cancer CSC-like traits by directly interacting with OCT4 mRNA, thus increasing OCT4 mRNA stability and expression, and knockdown of OCT4 could rescue the promoting effects of OIP5-AS1 overexpression on CSC-like traits. Another mechanism regulating OCT4 expression is the mediation of lncRNA metastasis-associated lung adenocarcinoma transcript 1 (MALAT1), promoting CSC-like properties. In colorectal cancer, MALAT1, a competing endogenous RNA, sponges miR-20b to partially regulate the expression of OCT4, thereby promoting maintenance of stemness [41]. Our results also indicated that lncRNA regulates CSC properties through OCT4; PDIA3P1 upregulates OCT4 expression, and after silencing OCT4, the cell sphere formation, percentage of SP cells, and CD271 + CD44 + cells promoted by PDIA3P1 are reversed, indicating that OCT4 mediates PDIA3P1 regulation of CSC properties in ESCC.

LncRNAs play important roles in tumor cells via different mechanisms, including microRNA sponging, cis- or trans-regulation, RNA decay, RNA scaffolding, epigenetic modifications, and post-translational modification [42–44]. Ubiquitination is the post-translational modification of proteins. Recently, the effects of lncRNAs on ubiquitination through various mechanisms have drawn considerable research attention. Mechanisms include [45] acting as competing endogenous RNA for miRNA of E3s, enhancing the interaction between E3 and its target, binding to the substrate and blocking its interaction with E3, linking the substrate to UPS components by lncRNA, binding to E3, recruiting deubiquitinase, and regulating ubiquitination via other PTMs of the target protein. Here, we revealed that the ubiquitination level of OCT4 was downregulated or upregulated after the overexpression or silencing of PDIA3P1. Mechanically, PDIA3P1 binding to OCT4 blocks its interaction with WWP2, leading to the accumulation of OCT4. Understanding how lncRNAs regulate ubiquitination will help us understand how lncRNAs are involved in cancer progression.

Furthermore, the mechanism underlying high expression of PDIA3P1 is still unclear in EC. In our study, we showed that the well-known stemness transcription factor OCT4 transcriptionally upregulates PDIA3P1 expression by binding to the promoter region. This result indicated that as a transcription factor related to stem cells, OCT4 not only plays a regulatory role in stem cells but also serves as a transcription factor to promote lncRNA transcription [46]. For example, the lncRNA nuclear enriched abundant transcript 1 was validated as an OCT4 transcriptional target through promoter activation [47]. Yao et al. [48] also reported that OCT4-induced MIAT transcriptional activation promotes 5-fluorouracil chemotherapy resistance in colon cancer. Therefore, we suggest that there is a positive feedback regulatory loop between PDIA3P1 and OCT4 that promotes esophageal CSC properties.

The identification of effective therapeutic strategies is critical to target CSCs. Mounting evidence suggests that CSCs contribute to the cancer therapeutic resistance and metastasis, leading to the recurrence and death in patients [49]. Our results suggest that PDIA3P1 positively regulates CSC properties by promoting OCT4 expression, indicating that PDIA3P1 might be a promising therapeutic target for esophageal cancer. Now, targeting lncRNAs by in vivo-optimized RNAi, locked nucleic acid (LNA), or other approaches could constitute a promising strategy for clinical therapy. The quality of RNA therapeutics depends on the strength of their target specificity and the presence of off-target effects. Many advances in the design of RNA therapeutics have improved target specificity and reduced unwanted off-target effects. Examples include Second-generation chemical modifications and third-generation LNA modifications. One of the biggest challenges in the field of RNA therapeutic delivery is not only to the organ and cell type of interest but also across the cell membrane to perform their intracellular functions. Different delivery systems are already either used in clinically approved therapeutics or have entered clinical testing, Such as Lipid nanoparticles (LNPs), Polymers and RNA conjugations. In our next study, more effective and stable approaches should be applied, such as LNA-based antisense oligonucleotide strategy to target lncRNAs or develop LNPs to encapsulate the si-RNAs, which lead to clinical translational applications of PDIA3P1 as an emerging target against CSCs in esophageal cancer.

## Conclusions

Our study provides evidence for the mutual promotion of PDIA3P1 and OCT4 in promoting the properties of esophageal CSCs. Additionally, we describe a new mechanism by which PDIA3P1 increases the stability and accumulation of OCT4 by disrupting the WWP2-OCT4 complex. OCT4 promotes PDIA3P1 expression at the transcriptional level, forming a positive feedback loop. Owing to this regulatory relationship, the PDIA3P1-WWP2-OCT4 positive feedback loop might be used in the diagnosis and prognosis, as well as in the development of novel therapeutics for EC.

### Supplementary Information

**Additional file 1:** **Table S1. **Primers used for qRT-PCR.

**Additional file 2:** **Table S2. **Primers used for PCR amplifications.

**Additional file 3:** **Table S3. **Primers used for RNA pull-down.

**Additional file 4:** **Table S4. **Primers used for CUT&Tag.

**Additional file 5.** Supplementary materials and methods.

**Additional file 6:** **Table S5. **The correlation between clinicopathological characteristics and PDIA3P1 expression level in 26 esophageal squamous cell carcinoma patients.

**Additional file 7.**

## Abbreviations

EC: Esophageal cancer
ESCC: Esophageal squamous cell carcinoma
lncRNA: Long non-coding RNA
nt: Nucleotides
SNHG16: Small nucleolar RNA host gene 16
Mi: Micro
linc-ROR: Long intergenic non-protein coding RNA
SOX: SRY-box transcription factor
CSC: Cancer stem cell
SP: Side population
OCT4: Octamer-binding transcription factor 4
C: Cellular
KFL4: Kruppel-like factor 4
PDIA3P1: Protein disulfide isomerase family A member 3 pseudogene
GEO: Gene Expression Omnibus
qRT-PCR: Quantitative real-time polymerase chain reaction
SI: Small interfering
CCK: Cell Counting Kit
Nanog: Homeobox protein NANOG
CHX: Cycloheximide
OIP5-AS1: Opa interacting protein 5-antisense
RNA 1 MALAT1: Metastasis-associated lung adenocarcinoma transcript 1

## References

[1] Sung H, Ferlay J, Siegel RL, Laversanne M, Soerjomataram I, Jemal A, Bray F (2021). Global Cancer Statistics 2020: GLOBOCAN Estimates of Incidence and Mortality Worldwide for 36 Cancers in 185 Countries. CA Cancer J Clin.

[2] Abnet CC, Arnold M, Wei WQ (2018). Epidemiology of Esophageal Squamous Cell Carcinoma. Gastroenterology.

[3] Smyth EC, Lagergren J, Fitzgerald RC, Lordick F, Shah MA, Lagergren P, Cunningham D (2017). Oesophageal cancer Nat Rev Dis Primers.

[4] Zhu J, Ma S, Zhou Y, Chen R, Xie S, Liu Z, Li X, Wei W (2021). The association between depression and esophageal cancer in China: a multicentre population-based study. BMC Psychiatry.

[5] Hu Y, Correa AM, Hoque A, Guan B, Ye F, Huang J, Swisher SG, Wu TT, Ajani JA, Xu XC (2011). Prognostic significance of differentially expressed miRNAs in esophageal cancer. Int J Cancer.

[6] Liu J, Wang Z, Wu K, Li J, Chen W, Shen Y, Guo S (2015). Paclitaxel or 5-fluorouracil/esophageal stent combinations as a novel approach for the treatment of esophageal cancer. Biomaterials.

[7] Kelly RJ, Ajani JA, Kuzdzal J, Zander T, Van Cutsem E, Piessen G, Mendez G, Feliciano J, Motoyama S, Lièvre A (2021). Adjuvant Nivolumab in Resected Esophageal or Gastroesophageal Junction Cancer. N Engl J Med.

[8] Li J, Xu J, Zheng Y, Gao Y, He S, Li H, Zou K, Li N, Tian J, Chen W, He J (2021). Esophageal cancer: Epidemiology, risk factors and screening. Chin J Cancer Res.

[9] Wu Q, Zhang H, Yang D, Min Q, Wang Y, Zhang W, Zhan Q (2022). The m6A-induced lncRNA CASC8 promotes proliferation and chemoresistance via upregulation of hnRNPL in esophageal squamous cell carcinoma. Int J Biol Sci.

[10] Li Z, Qin X, Bian W, Li Y, Shan B, Yao Z, Li S (2019). Exosomal lncRNA ZFAS1 regulates esophageal squamous cell carcinoma cell proliferation, invasion, migration and apoptosis via microRNA-124/STAT3 axis. J Exp Clin Cancer Res.

[11] Zhang H, Hua Y, Jiang Z, Yue J, Shi M, Zhen X, Zhang X, Yang L, Zhou R, Wu S (2019). Cancer-associated Fibroblast-promoted LncRNA DNM3OS Confers Radioresistance by Regulating DNA Damage Response in Esophageal Squamous Cell Carcinoma. Clin Cancer Res.

[12] Zhang L, Liang H, Zhang J, Yang Y, Ling X, Jiang H (2022). Long Non-coding RNA SNHG16 Facilitates Esophageal Cancer Cell Proliferation and Self-renewal through the microRNA-802/PTCH1 Axis. Curr Med Chem.

[13] Wang L, Yu X, Zhang Z, Pang L, Xu J, Jiang J, Liang W, Chai Y, Hou J, Li F (2017). Linc-ROR promotes esophageal squamous cell carcinoma progression through the derepression of SOX9. J Exp Clin Cancer Res.

[14] Henkin RI (2019). Clinical and Therapeutic Implications of Cancer Stem Cells. N Engl J Med.

[15] Zhao W, Li Y, Zhang X (2017). Stemness-Related Markers in Cancer. Cancer Transl Med.

[16] Huang T, Wu Z, Zhu S (2022). The roles and mechanisms of the lncRNA-miRNA axis in the progression of esophageal cancer: a narrative review. J Thorac Dis.

[17] McCabe EM, Rasmussen TP (2021). lncRNA involvement in cancer stem cell function and epithelial-mesenchymal transitions. Semin Cancer Biol.

[18] Takahashi K, Tanabe K, Ohnuki M, Narita M, Ichisaka T, Tomoda K, Yamanaka S (2007). Induction of pluripotent stem cells from adult human fibroblasts by defined factors. Cell.

[19] Yang X, Yang B (2019). lncRNA PDIA3P regulates cell proliferation and invasion in non-small cell lung cancer. Exp Ther Med.

[20] Sun CC, Zhang L, Li G, Li SJ, Chen ZL, Fu YF, Gong FY, Bai T, Zhang DY, Wu QM, Li DJ (2017). The lncRNA PDIA3P Interacts with miR-185-5p to Modulate Oral Squamous Cell Carcinoma Progression by Targeting Cyclin D2. Mol Ther Nucleic Acids.

[21] Gao Z, Xu J, Fan Y, Qi Y, Wang S, Zhao S, Guo X, Xue H, Deng L, Zhao R (2022). PDIA3P1 promotes Temozolomide resistance in glioblastoma by inhibiting C/EBPβ degradation to facilitate proneural-to-mesenchymal transition. J Exp Clin Cancer Res.

[22] Wang S, Qi Y, Gao X, Qiu W, Liu Q, Guo X, Qian M, Chen Z, Zhang Z, Wang H (2020). Hypoxia-induced lncRNA PDIA3P1 promotes mesenchymal transition via sponging of miR-124-3p in glioma. Cell Death Dis.

[23] Kong Y, Zhang L, Huang Y, He T, Zhang L, Zhao X, Zhou X, Zhou D, Yan Y, Zhou J (2017). Pseudogene PDIA3P1 promotes cell proliferation, migration and invasion, and suppresses apoptosis in hepatocellular carcinoma by regulating the p53 pathway. Cancer Lett.

[24] Yang X, Ye H, He M, Zhou X, Sun N, Guo W, Lin X, Huang H, Lin Y, Yao R, Wang H (2018). LncRNA PDIA3P interacts with c-Myc to regulate cell proliferation via induction of pentose phosphate pathway in multiple myeloma. Biochem Biophys Res Commun.

[25] Zhou C, Fan N, Liu F, Fang N, Plum PS, Thieme R, Gockel I, Gromnitza S, Hillmer AM, Chon S-H, et al. Linking Cancer Stem Cell Plasticity to Therapeutic Resistance-Mechanism and Novel Therapeutic Strategies in Esophageal Cancer. Cells. 2020;9(6):1481. 10.3390/cells9061481 PMC7349233 32560537

[26] Sun X, Sun Y, Li J, Zhao X, Shi X, Gong T, Pan S, Zheng Z, Zhang X. SOCS6 promotes radiosensitivity and decreases cancer cell stemness in esophageal squamous cell carcinoma by regulating c-Kit ubiquitylation. Cancer Cell Int. 2021;21:165. 10.1186/s12935-021-01859-2 PMC7953756 33712005

[27] Wang JL, Yu JP, Sun ZQ, Sun SP (2014). Radiobiological characteristics of cancer stem cells from esophageal cancer cell lines. World J Gastroenterol.

[28] Dikic I (2017). Proteasomal and Autophagic Degradation Systems. Annu Rev Biochem.

[29] Ni W, Yao S, Zhou Y, Liu Y, Huang P, Zhou A, Liu J, Che L, Li J (2019). Long noncoding RNA GAS5 inhibits progression of colorectal cancer by interacting with and triggering YAP phosphorylation and degradation and is negatively regulated by the m(6)A reader YTHDF3. Mol Cancer.

[30] Zhang N, Wang B, Ma C, Zeng J, Wang T, Han L, Yang M (2023). LINC00240 in the 6p22.1 risk locus promotes gastric cancer progression through USP10-mediated DDX21 stabilization. J Exp Clin Cancer Res.

[31] Liu CQ, Ma YL, Qin Q, Wang PH, Luo Y, Xu PF, Cui Y (2023). Epidemiology of esophageal cancer in 2020 and projections to 2030 and 2040. Thorac Cancer.

[32] Ghafouri-Fard S, Shoorei H, Dashti S, Branicki W, Taheri M (2020). Expression profile of lncRNAs and miRNAs in esophageal cancer: Implications in diagnosis, prognosis, and therapeutic response. J Cell Physiol.

[33] Xie C, Zhang LZ, Chen ZL, Zhong WJ, Fang JH, Zhu Y, Xiao MH, Guo ZW, Zhao N, He X, Zhuang SM (2020). A hMTR4-PDIA3P1-miR-125/124-TRAF6 Regulatory Axis and Its Function in NF kappa B Signaling and Chemoresistance. Hepatology.

[34] Chang JC (2016). Cancer stem cells: Role in tumor growth, recurrence, metastasis, and treatment resistance. Medicine (Baltimore).

[35] Li W, Zhang L, Guo B, Deng J, Wu S, Li F, Wang Y, Lu J, Zhou Y (2019). Exosomal FMR1-AS1 facilitates maintaining cancer stem-like cell dynamic equilibrium via TLR7/NFκB/c-Myc signaling in female esophageal carcinoma. Mol Cancer.

[36] Wu D, He X, Wang W, Hu X, Wang K, Wang M (2020). Long noncoding RNA SNHG12 induces proliferation, migration, epithelial-mesenchymal transition, and stemness of esophageal squamous cell carcinoma cells via post-transcriptional regulation of BMI1 and CTNNB1. Mol Oncol.

[37] Haghi B, Saghaeian Jazi M, Khosravi A, Jafari SM, Asadi J. SOX2OT lncRNA Inhibition Suppresses the Stemness Characteristics of Esophageal Tumorspheres. Noncoding RNA. 2022;8(6):80. 10.3390/ncrna8060080 PMC9782980 36548179

[38] Osorno R, Chambers I (2011). Transcription factor heterogeneity and epiblast pluripotency. Philos Trans R Soc Lond B Biol Sci.

[39] Wang Q, He W, Lu C, Wang Z, Wang J, Giercksky KE, Nesland JM, Suo Z (2009). Oct3/4 and Sox2 are significantly associated with an unfavorable clinical outcome in human esophageal squamous cell carcinoma. Anticancer Res.

[40] Mao C, Li X (2022). Long noncoding RNA OIP5-AS1 promotes the stemness of lung cancer cells through enhancing Oct4 mRNA stability. Environ Toxicol.

[41] Tang D, Yang Z, Long F, Luo L, Yang B, Zhu R, Sang X, Cao G, Wang K (2019). Long noncoding RNA MALAT1 mediates stem cell-like properties in human colorectal cancer cells by regulating miR-20b-5p/Oct4 axis. J Cell Physiol.

[42] Hu G, Niu F, Humburg BA, Liao K, Bendi S, Callen S, Fox HS, Buch S (2018). Molecular mechanisms of long noncoding RNAs and their role in disease pathogenesis. Oncotarget.

[43] Chen B, Li Y, He Y, Xue C, Xu F (2018). The emerging roles of long non-coding RNA in gallbladder cancer tumorigenesis. Cancer Biomark.

[44] Bär C, Chatterjee S, Thum T (2016). Long Noncoding RNAs in Cardiovascular Pathology, Diagnosis, and Therapy. Circulation.

[45] Ma X, Dang Y, Shao X, Chen X, Wu F, Li Y. Ubiquitination and Long Non-coding RNAs Regulate Actin Cytoskeleton Regulators in Cancer Progression. Int J Mol Sci. 2019;20(12):2997. 10.3390/ijms20122997 PMC6627692 31248165

[46] Sheik Mohamed J, Gaughwin PM, Lim B, Robson P, Lipovich L (2010). Conserved long noncoding RNAs transcriptionally regulated by Oct4 and Nanog modulate pluripotency in mouse embryonic stem cells. RNA.

[47] Jen J, Tang YA, Lu YH, Lin CC, Lai WW, Wang YC (2017). Oct4 transcriptionally regulates the expression of long non-coding RNAs NEAT1 and MALAT1 to promote lung cancer progression. Mol Cancer.

[48] Yao X, Tu Y, Xu Y, Guo Y, Yao F, Zhang X (2020). Endoplasmic reticulum stress confers 5-fluorouracil resistance in breast cancer cell via the GRP78/OCT4/lncRNA MIAT/AKT pathway. Am J Cancer Res.

[49] Bai X, Ni J, Beretov J, Graham P, Li Y (2018). Cancer stem cell in breast cancer therapeutic resistance. Cancer Treat Rev.

