# Supplementary material for: A positive feedback between PDIA3P1 and OCT4 promotes the cancer stem cell properties of esophageal squamous cell carcinoma
Source: Cell Commun Signal. 2024 Jan 22;22:60. doi: 10.1186/s12964-024-01475-3 (PMC10801955; doi:10.1186/s12964-024-01475-3)
Supplement: Supplementary file 1 — Additional file 1: Table S1. Primers used for qRT-PCR. [file 12964_2024_1475_MOESM1_ESM.docx]

**Additional file 1: Table S1. Primers used for qRT-PCR**

| Gene | Primer (5'>3') |
| --- | --- |
| PDIA3P1 | Forward: GGAAAACCACTGGGGAGGAC  Reverse: CAGTGCAGCTAAGAAATGGCT |
| CD271 | Forward: TCAGTGGCATGGCTCCAGTC  Reverse: GCAGTATCCAGTCTCAGCCCAAG |
| CD133 | Forward: TTGGCTCAGACTGGTAAATCCC  Reverse: ATAGGAAGGACTCGTTGCTGGT |
| CD90 | Forward: AAGCCAGGATTGGGGATGTG  Reverse: TGTGGCAGAGAAAGCTCCTG |
| CD44 | Forward: CCCCATTACCAAAGACCACGA  Reverse: TTCTGCAGGTTCCGTGTCTC |
| CD54 | Forward: CCTGATGGGCAGTCAACAGCTA  Reverse: ACAGCTGGCTCCCGTTTCA |
| OCT4  U6  β-actin | Forward: GAGAACCGAGTGAGAGGCAACC  Reverse: CATAGTCGCTGCTTGATCGCTTG  Forward: CTCGCTTCGGCAGCACA  Reverse: TGGTGTCGTGGAGTCG  Forward: CCTTCCTGGGCATGGAGTCCT  Reverse: GGAGCAATGATCTTGATCTTC |
