# Supplementary material for: A positive feedback between PDIA3P1 and OCT4 promotes the cancer stem cell properties of esophageal squamous cell carcinoma
Source: Cell Commun Signal. 2024 Jan 22;22:60. doi: 10.1186/s12964-024-01475-3 (PMC10801955; doi:10.1186/s12964-024-01475-3)
Supplement: Supplementary file 2 — Additional file 2: Table S2. Primers used for PCR amplifications. [file 12964_2024_1475_MOESM2_ESM.docx]

**Additional file 2: Table S2. Primers used for PCR amplifications**

| Gene | Name | Primer (5'>3') |
| --- | --- | --- |
| PDIA3P1 | PDIA3P1-Δ1 | Forward: CCCAAGCTTAAACTAAATCA  AACTTGAGTATGAAAC  Reverse: GGGGTACCTTAGTTTTTGTGT  TCCAGGATCAGATT |
| PDIA3P1 | PDIA3P1-Δ2 | Forward: CCCAAGCTTAAACTAAATCA  AACTTGAGTATGAAAC  Reverse: GGGGTACCTGCGACTCTCCA  AGTTGTCGTCCCTGA |
| PDIA3P1 | PDIA3P1-Δ3 | Forward: CCCAAGCTTATTAGTAGAAA  AGTGGTAAAATAGGTG  Reverse: GGGGTACCTGCGACTCTCCA  AGTTGTCGTCCCTGA |
| PDIA3P1 | PDIA3P1-Δ4 | Forward: CCCAAGCTTATTAGTAGAAA  AGTGGTAAAATAGGTG  Reverse: GGGGTACCAGTTTAAAGGGG  TCTTATTTATTGTCA |
| PDIA3P1 | PDIA3P1-Δ5 | Forward: CCCAAGCTTTCTCCGACACG  GGCTCTGCGGGCCTCA  Reverse: GGGGTACCAGTTTAAAGGGG  TCTTATTTATTGTCA |
| PDIA3P1 | Promoter-1820 | Forward: GGGGTACCTTGACTGCAGTG  GAGTTATTGATACTT  Reverse: CCCAAGCTTAACCTAATGTTC  TTTTTCTGTCCTGGG |
| PDIA3P1  PDIA3P1  PDIA3P1 | Promoter-1576  Promoter-743  Promoter-416 | Forward: GGGGTACCAGGAAGATAGAC  CCAATTCAAAGGTAC  Reverse: CCCAAGCTTAACCTAATGTTC  TTTTTCTGTCCTGGG  Forward: GGGGTACCCCAGAGCCCTTGA  GAAAAATGCCTAAT  Reverse: CCCAAGCTTAACCTAATGTTC  TTTTTCTGTCCTGGG  Forward: GGGGTACCTCTACCCTCGAGA  AGATGGAACATAAT  Reverse: CCCAAGCTTAACCTAATGTTC  TTTTTCTGTCCTGGG |
