# Supplementary material for: A positive feedback between PDIA3P1 and OCT4 promotes the cancer stem cell properties of esophageal squamous cell carcinoma
Source: Cell Commun Signal. 2024 Jan 22;22:60. doi: 10.1186/s12964-024-01475-3 (PMC10801955; doi:10.1186/s12964-024-01475-3)
Supplement: Supplementary file 3 — Additional file 3: Table S3. Primers used for RNA pull-down. [file 12964_2024_1475_MOESM3_ESM.docx]

**Additional file 3: Table S3. Primers used for RNA pull-down**

| Gene | Fragment | Primer (5'>3') |
| --- | --- | --- |
| PDIA3P1 | 1-2099 nt | Forward: TAATACGACTCACTATAGGGAAAC  TAAATCAAACTTGAGTATGAAAC  Reverse: GGCGCTTTGTCAGCGCCAGTTTAA  AGGGGTCTTATTTATTGTCA |
| PDIA3P1 | 1-164 nt | Forward: TAATACGACTCACTATAGGGAAAC  TAAATCAAACTTGAGTATGAAAC  Reverse: GGCGCTTTGTCAGCGCCTTAGTTTT  TGTGTTCCAGGATCAGATT |
| PDIA3P1 | 1-378 nt | Forward: TAATACGACTCACTATAGGGAAAC  TAAATCAAACTTGAGTATGAAAC  Reverse: GGCGCTTTGTCAGCGCCTGCGACTC  TCCAAGTTGTCGTCCCTGA |
| PDIA3P1 | 165-378 nt | Forward: TAATACGACTCACTATAGGGATTA  GTAGAAAAGTGGTAAAATAGGTG  Reverse: GGCGCTTTGTCAGCGCCTGCGACTC  TCCAAGTTGTCGTCCCTGA |
| PDIA3P1 | 165-2099 nt | Forward: TAATACGACTCACTATAGGGATTA  GTAGAAAAGTGGTAAAATAGGTG  Reverse: GGCGCTTTGTCAGCGCCAGTTTAA  AGGGGTCTTATTTATTGTCA |
| PDIA3P1 | 379-2099 nt | Forward: TAATACGACTCACTATAGGGTCTC  CGACACGGGCTCTGCGGGCCTCA  Reverse: GGCGCTTTGTCAGCGCCAGTTTAA  AGGGGTCTTATTTATTGTCA |
| PDIA3P1 | Antisense | Forward: GGCGCTTTGTCAGCGCCAAACTAA  ATCAAACTTGAGTATGAAAC  Reverse: TAATACGACTCACTATAGGGAGTT  TAAAGGGGTCTTATTTATTGTCA |
