# Supplementary material for: A positive feedback between PDIA3P1 and OCT4 promotes the cancer stem cell properties of esophageal squamous cell carcinoma
Source: Cell Commun Signal. 2024 Jan 22;22:60. doi: 10.1186/s12964-024-01475-3 (PMC10801955; doi:10.1186/s12964-024-01475-3)
Supplement: Supplementary file 4 — Additional file 4: Table S4. Primers used for CUT&Tag. [file 12964_2024_1475_MOESM4_ESM.docx]

**Additional file 4: Table S4. Primers used for CUT&Tag**

| Gene | Name | Primer (5'>3') |
| --- | --- | --- |
| PDIA3P1 | Promoter-Set1 | Forward: GTTATTGATACTTACCAAACTGTAC  Reverse: ATTTCTCTGCCTGTATAAAAACTAT |
| PDIA3P1 | Promoter-Set2 | Forward: TGCTCACCTAAAGGAAGATAGACCC  Reverse: AACCTGCACCTCATTTATAAAATGG |
| PDIA3P1 | Promoter-Set3 | Forward: TATCAAAGTGACATAGGAGCCAACT  Reverse: ATGTTCCATCTTCTCGAGGGTAGAG |
|  |  |  |
