# Supplementary material for: A positive feedback between PDIA3P1 and OCT4 promotes the cancer stem cell properties of esophageal squamous cell carcinoma
Source: Cell Commun Signal. 2024 Jan 22;22:60. doi: 10.1186/s12964-024-01475-3 (PMC10801955; doi:10.1186/s12964-024-01475-3)
Supplement: Supplementary file 5 — Additional file 5. Supplementary materials and methods. [file 12964_2024_1475_MOESM5_ESM.docx]

**Additional file 5: Supplementary materials and methods**

**Cells, transfection, antibodies, plasmids, reagents**

HEEC, KYSE-30, KYSE-150, KYSE-520, TE-1 and Eca109 (human esophageal squamous cell carcinoma) cell lines were routinely cultured in RPMI 1640 (Lonsera, China) with 10% fetal bovine serum (FBS). For transfection, cells were transfected with PolyJet™ DNA transfection reagent when the cell density reached 80-90% confluence (SignaGen Laboratories, Gaithersburg, MD). Anti-OCT4 (ab181557) antibody was purchased from Abcam (Cambridge, UK). Anti-SOX2 (A0561), Anti-WWP2 (A2425), Anti-Snail (A5243), Anti- N-Cadherin (A3045), Anti- E-Cadherin (A3044), Anti-Vimentin (A11423), Anti-Snail (A5243), Anti-β-Actin (AC004) and Rabbit Control IgG (AC005) antibodies were obtained from ABclonal (Wuhan, China). Anti-ITCH (K111513P) antibody was purchased from Solarbio (Beijing, China), while Anti-KLF4 (WL02532) and anti-c-Myc (WL01781) antibodies from Wanleibio (Shenyang, China). Anti-Nanog (ET1610-2), anti-Ubiquitin (ET1609-21) and TRITC Conjugated Goat Anti-Rabbit lgG H&L (HA1016) antibodies were obtained from Huabio (Hangzhou, China). The HA-Ubiquitin (P37795), HA-WWP2 (P47342), FLAG-ITCH (P33655), pCMV-FLAG-OCT4 (P49895) and pCMV-PDIA3P1 (P46811) were obtained from MiaoLingBio (Wuhan, China). The pRL-CMV vector was purchased from Promega (Madison, WI, USA). MG-132 (HY-13259) and Cycloheximide (CHX, HY-12320) were purchased from MedChemExpress. Chloroquine (CQ, CSN25650) from CSNpharm. Verapamil (V875827) is from Macklin.

**Small interfering RNA (siRNA)**

The cells (30%-50% density) were transfected with 100nM siRNA mixed with 4.5 μl GenMute (SignaGen, SL100568) in a cell culture plate. Cells were cultured for 48 h before plating for protein or RNA isolation and various assays. PDIA3P1 si#1: 5′- AAGGAGACUUAACAACUAAdTdT-3′; PDIA3P1 si#2: 5′- CAACGAUUAGAGGACACUAdTdT-3′; OCT4 si: 5′- UUAAGUUCUUCAUUCACUAAGdTdT-3′.

**Cell proliferation assay**

**Cell counting kit-8 (CCK-8) assay**

An Enhanced Cell Counting Kit-8 (CCK-8) (BL1055A, Bioshaarp, China) was used in the cell proliferation assay, wherein 5000 cells/well transfected with plasmid or siRNA was seeded in 48-well plates. Then the samples were incubated with CCK-8 reagent at 37 °C for 1 h after 24, 48, 72 and 96 hours of incubation. According to manufacturer’s instruction, the cell proliferation rate was determined at 450 nm absorbance.

**Clone formation assay**

Cells were counted, plated in triplicate in 6-well plate at 1000 cells transfected with siRNA per well, and cultured for 10 days. Then the cells were fixed with 4% paraformaldehyde for 15 min. The fixed cells were stained using crystal violet solution for each sample and repeated in triplicate. After washing out the dye, we counted the number of clones and compared the results.

**EdU staining assay**

Transfected cells were seeded in 96-well plates and incubated with RPMl 1640 medium containing EdU (Cell-LightTM Apollo 567 Stain Kit, C10310-1, RiboBio) for 2 h. Following cell fixation by 4% formaldehyde and cell membrane permeation by 0.5% Triton X-100, the cells were stained with Apollo dye solution and Hoechst33342, and detected cell proliferation following the manufacturer’s protocol. The images were photographed with BioTek citation 5 (BioTek, Winooski, VT).

**Flow cytometry**

**Cell apoptosis assay**

After cell transfection, 1 × 10^6^ cells were collected from each group. The cell apoptosis was detected using Annexin V-FITC Apoptosis Detection Kit (BL107A, Biosharp) according to the manufacturer’s instructions. 10 μL Annexin V-FITC and 5 μL PI were added to the 500 μL cell resuspension and incubated for 30 mins in the dark. The percentage of apoptotic cells were detected by a CytoFLEX flow cytometer (Beckman Coulter, CA, USA).

**CD271+/CD44+ cells assay**

1 × 10^6^ transfected cells were harvested and resuspended in PBS for analysis. Cells were stained with anti-CD44-FITC-conjugated (11-0441-82) and anti-CD271-APC-conjugated (17-9400-42) antibodies (both from Invitrogen) for 30 minutes. The percentage of CD271+/CD44+ cells with CSC characteristics from ESCC cells were analyzed using flow cytometry.

**Side population (SP) cells assay**

1 × 10^6^ cells with transfected with plasmid or siRNA were suspended in 10% FBS and then cells were stained using 5 µg/ml Vybrant DyeCycle Violet (V35003, Invitrogen) with or without 50 µM verapamil at 37 °C for 90 min. The SP cells were visualized after UV excitation on the basis of blue emission through a 450/65 filter and of red emission through a 670/30 filter. The cells were analyzed using flow cytometry.

**Transwell assay**

Cell migration was determined by a transwell assay using a transwell chamber with an 8.0-mm pore size (Jetbiofil, China). Upper transwell chambers coated with Matrigel (510201, Tecono, China) was used for invasion assay. 2 × 104 Cells were inoculated into the upper chamber in 250 μl of serum-free medium and 500 μl of 10% serum-containing medium was added to the lower chamber. After 48 hours, culture medium was discarded and the cells were fixed with 4% paraformaldehyde for 10 mins and stained with crystal violet (C0121, Beyotime, China) for 20 mins. Cell invasion was quantified by visual counting after being photographed. Experiments were performed in triplicate. Mean values for three random fields were obtained for each well. Cell migration assay was performed similarly without coating upper chambers with Matrigel, and was photographed and counted after 24 hours.

**Western blotting**

The total protein was extracted from variously treated cells using Laemmli 2 × Concentrate (S3401; Sigam). Protein per sample was separated by polyacrylamide gel electrophoresis and then transferred to nitrocellulose (NC) membrane (66485, PALL, USA). Then, these membranes were blocked with 5% non-fat milk, probed with primary antibodies at 4℃ overnight and incubated with the corresponding HRP-conjugated secondary antibody. After incubation with an enhanced chemiluminescence kit (ECL, Millipore, Burlington, USA), the protein bands were captured by a ChemiDoc Touch Imaging System (Bio-Rad, Hercules, USA).

**Sphere-forming assay**

The cells were then seeded in Ultra-low-attachment 6-well plates (Corning, USA) at 10000 cells/well. Cells were propagated in the serum-free DMEM/F12 (Gibco, Rockville, MD, USA) adding 20 ng/mL of epidermal growth factor (EGF; Sangon Biotech, China), 20 ng/mL of basic fibroblast growth factor (bFGF; P5453, Beyotime, China), 1% B-27 (Gibco, Rockville, MD, USA), and 1x Penicillin-Streptomycin Solution (C0222, Beyotime, China). After 10 d, cell spheres formed from the cells were counted.

**Immunofluorescence (IF) assay**

ESCC cells were seeded in a confocal dish and fixed in 4% paraformaldehyde for 15 min and washed three times in PBS. Then cells were permeabilized in Triton X-100 (P0096, Beyotime, China) for 10 min and blocked with 1% Bovine Serum Albumin for 30 minutes. Then the cells were incubated with indicated primary antibodies overnight at 4 °C. Cells were then incubated with secondary antibody at room temperature for 1 h. DAPI (BL105A, Biosharp, China) was used to counterstain nuclei. The cells were washed gently with PBS for 3 times, the confocal dish surface was sealed with Antifade Mounting Medium (P0128S, Beyotime, China). Fluorescence detection of immunofluorescence Microscope (Leica TCS SP8; Leica Microsystems, Mannheim, Germany).

**Immunoprecipitation (IP)**

The cells were collected in an IP lysis buffer containing the protease inhibitor PMSF (Beyotime, ST505) and protease inhibitor cocktail (P1005, Biosharp, China). 500 μg of whole cell lysate protein was pre-cleared by incubation with 1.0 μg of the OCT4 antibody overnight at 4°, IgG as a control. The mixture was further incubated with Protein A+G Agarose (P2012, Beyotime, China) for 5 h, and after washing three times with cold PBS buffer, the beads were boiled in 2 x SDS loading buffer. Protein expression analysis was performed by Western blotting.

**Isolation of cytoplasmic and nuclear RNA**

Cytoplasmic and nuclear RNAs of KYSE-150 and Eca-109 cells were separately isolated using the nuclear/cytoplasmic Isolation Kit (Norgen, NGB-21000, Canada) according to the manufacturer’s instructions. The relative RNA levels of PDIA3P1 were detected by qRT-PCR.
