# Supplementary material for: A positive feedback between PDIA3P1 and OCT4 promotes the cancer stem cell properties of esophageal squamous cell carcinoma
Source: Cell Commun Signal. 2024 Jan 22;22:60. doi: 10.1186/s12964-024-01475-3 (PMC10801955; doi:10.1186/s12964-024-01475-3)
Supplement: Supplementary file 6 — Additional file 6: Table S5. The correlation between clinicopathological characteristics and PDIA3P1 expression level in 26 esophageal squamous cell carcinoma patients. [file 12964_2024_1475_MOESM6_ESM.docx]

**Additional file 6: Table S5.** The correlation between clinicopathological characteristics and PDIA3P1 expression level in 26 esophageal squamous cell carcinoma patients.

| **Characteristics** | **No. of patients (%)** | **PDIA3P1 expression** | | ***P*** *^a^* |
| --- | --- | --- | --- | --- |
|  |  | **Low *n* = 13 (%)** | **High *n* = 13 (%)** |  |
| Age |  |  |  | 0.658 |
| <60 years | 7 (26.9) | 4 (30.8) | 3 (23.1) |  |
| ≥60 years | 19 (73.1) | 9 (69.2) | 10 (76.9) |  |
| Gender |  |  |  | 0.619 |
| Female | 5 (19.2) | 3 (23.1) | 2 (15.4) |  |
| Male | 21 (80.8) | 10 (76.9) | 11 (84.6) |  |
| Differentiation |  |  |  | 0.047* |
| Well | 11 (42.3) | 8 (61.5) | 3 (23.1) |  |
| Moderate/Poor | 15 (57.7) | 5 (38.5) | 10 (76.9) |  |
| TNM stage |  |  |  | 0.006** |
| I/II | 15 (57.7) | 11 (84.6) | 4 (30.8) |  |
| III/IV | 11 (42.3) | 2 (15.4) | 9 (69.2) |  |
| T grade |  |  |  | 0.039* |
| T1-2 | 17 (65.4) | 11 (84.6) | 6 (46.2) |  |
| T3-4 | 9 (34.6) | 2 (15.4) | 7 (53.8) |  |
| Lymph node metastasis |  |  |  | 0.116 |
| No | 12 (20.0) | 8 (22.5) | 4 (17.5) |  |
| Yes | 14 (80.0) | 5 (77.5) | 9 (82.5) |  |

^a^ Chi-square test results, *P < 0.05 and **P < 0.01.
