# Supplementary figures and images for: A positive feedback between PDIA3P1 and OCT4 promotes the cancer stem cell properties of esophageal squamous cell carcinoma (part 2 of 2)
Source: Cell Commun Signal. 2024 Jan 22;22:60. doi: 10.1186/s12964-024-01475-3 (PMC10801955; doi:10.1186/s12964-024-01475-3)

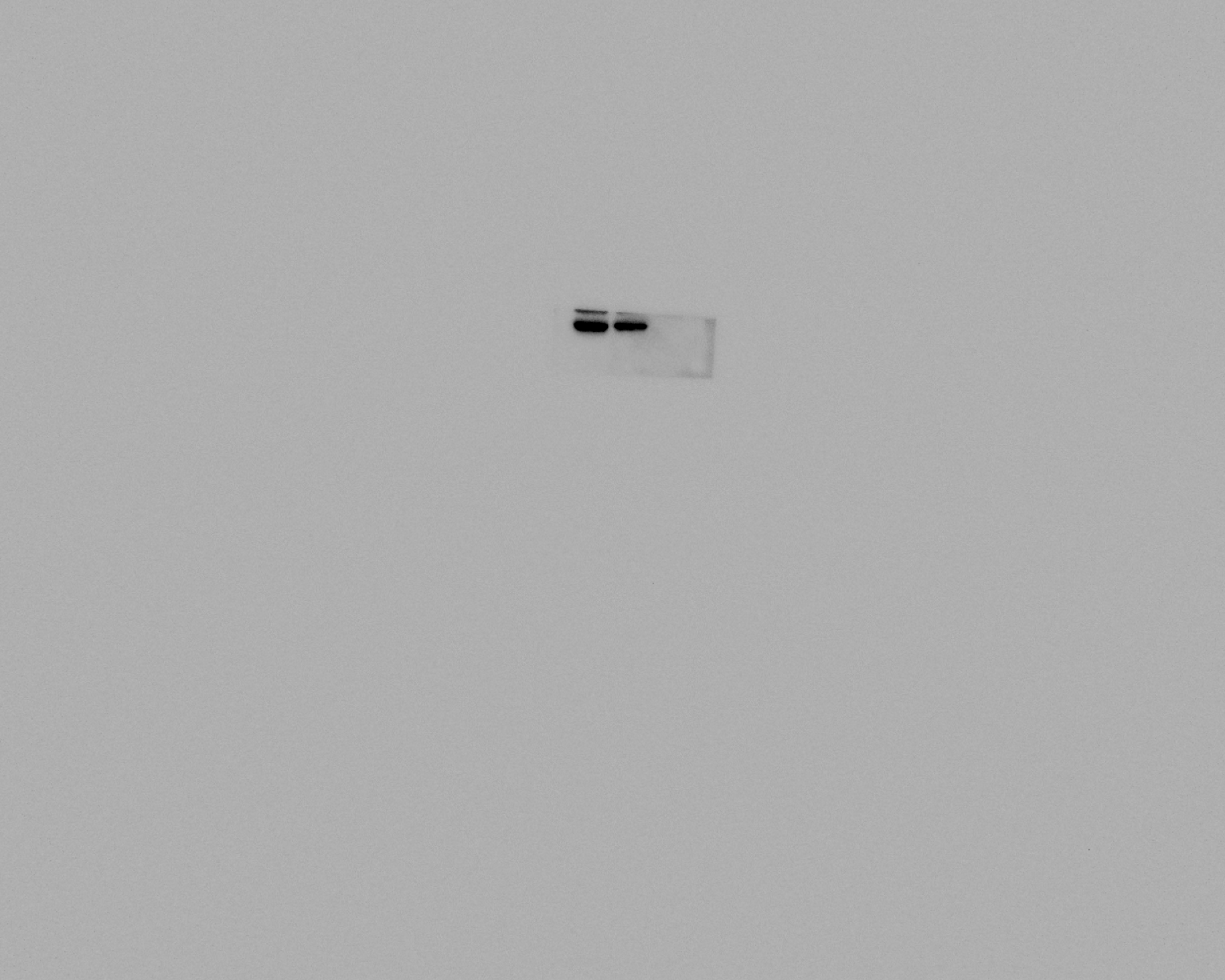

Supplement: Supplementary file 7 — Additional file 7. [file 12964_2024_1475_MOESM7_ESM.zip › Additional file 2/Figure 4F/KYSE-150/oct4.tif]

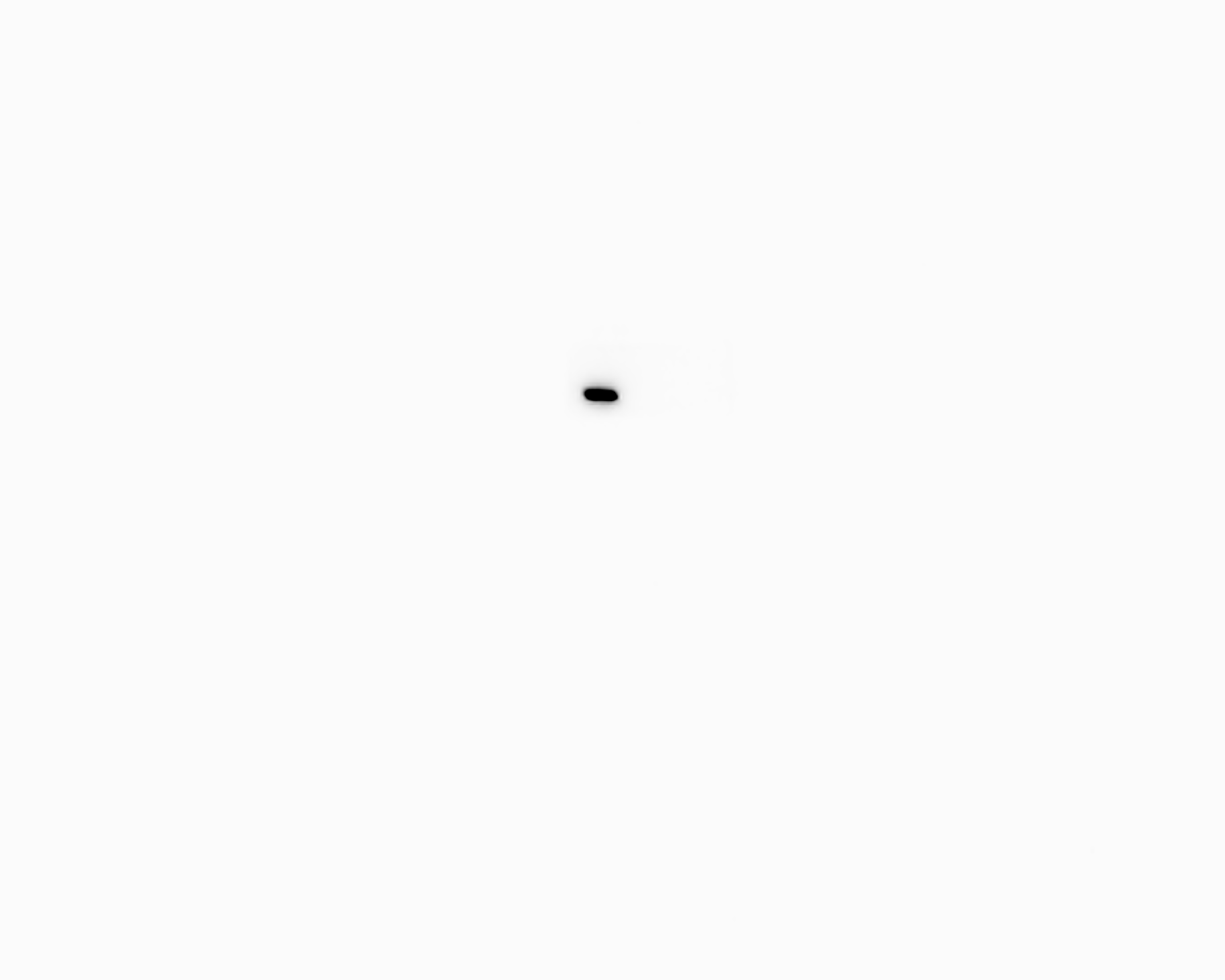

Supplement: Supplementary file 7 — Additional file 7. [file 12964_2024_1475_MOESM7_ESM.zip › Additional file 2/Figure 4F/KYSE-150/a┬-actin.tif]

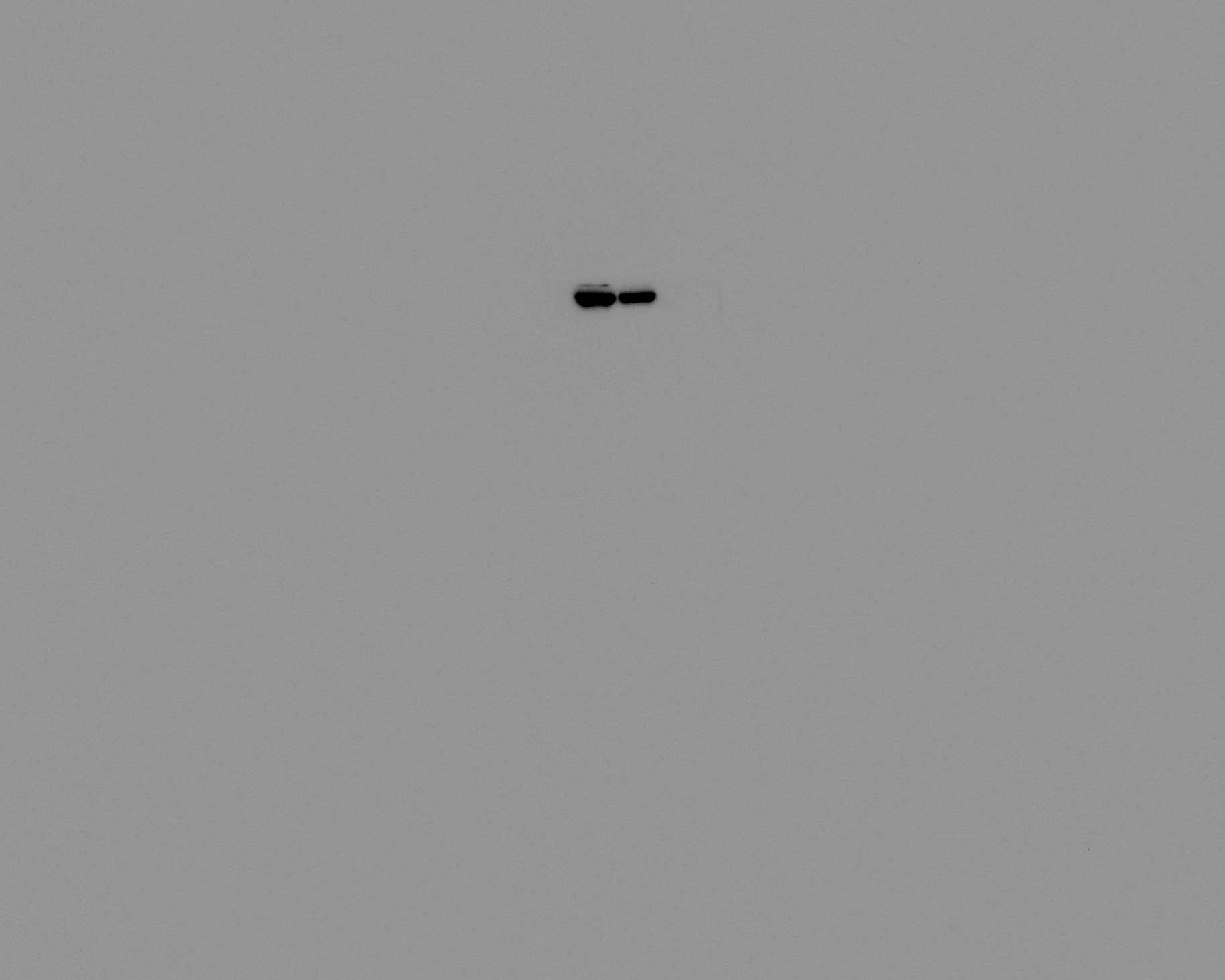

Supplement: Supplementary file 7 — Additional file 7. [file 12964_2024_1475_MOESM7_ESM.zip › Additional file 2/Figure 4H/Eca-109 oct4.tif]

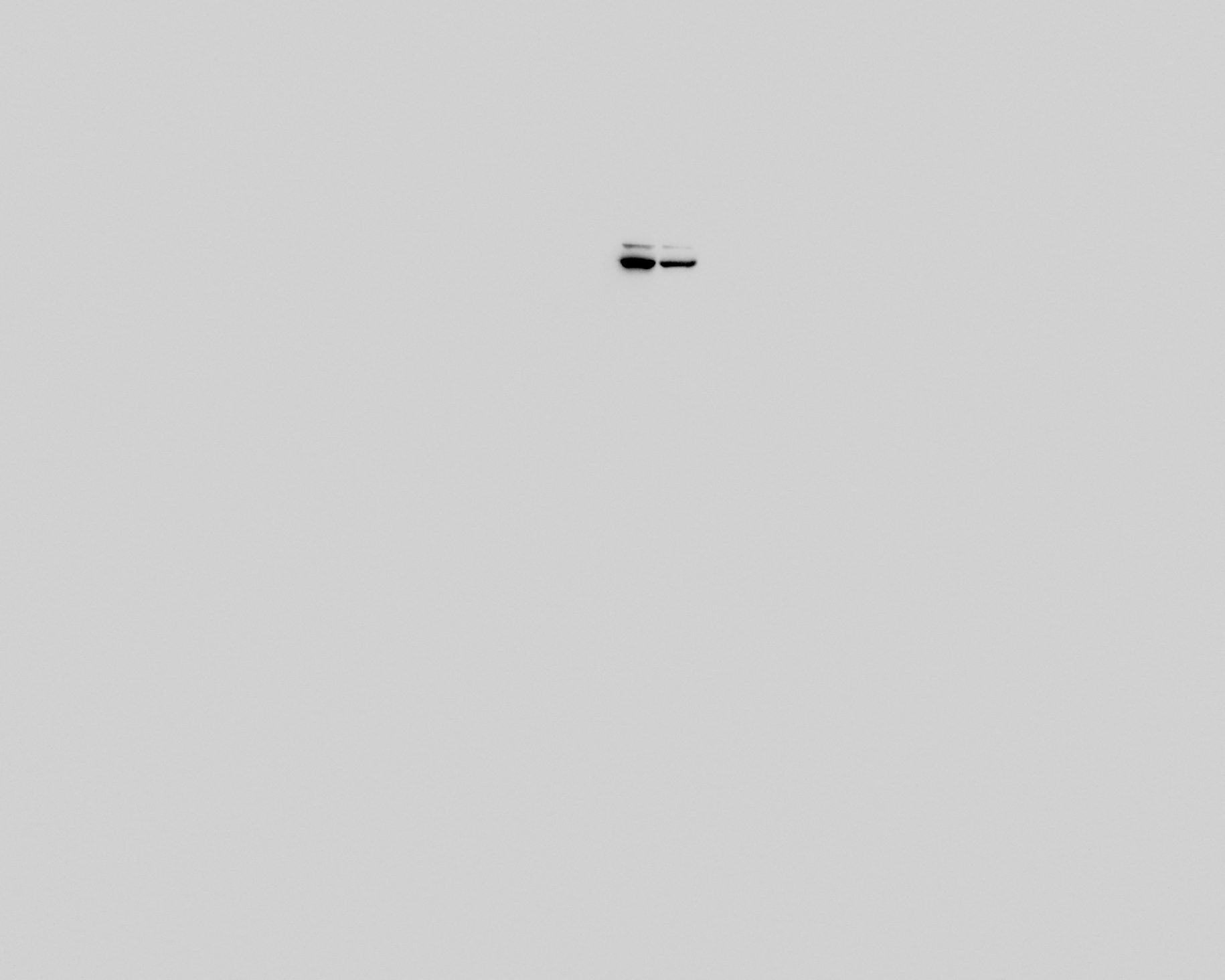

Supplement: Supplementary file 7 — Additional file 7. [file 12964_2024_1475_MOESM7_ESM.zip › Additional file 2/Figure 4H/KYSE-150 oct4.tif]

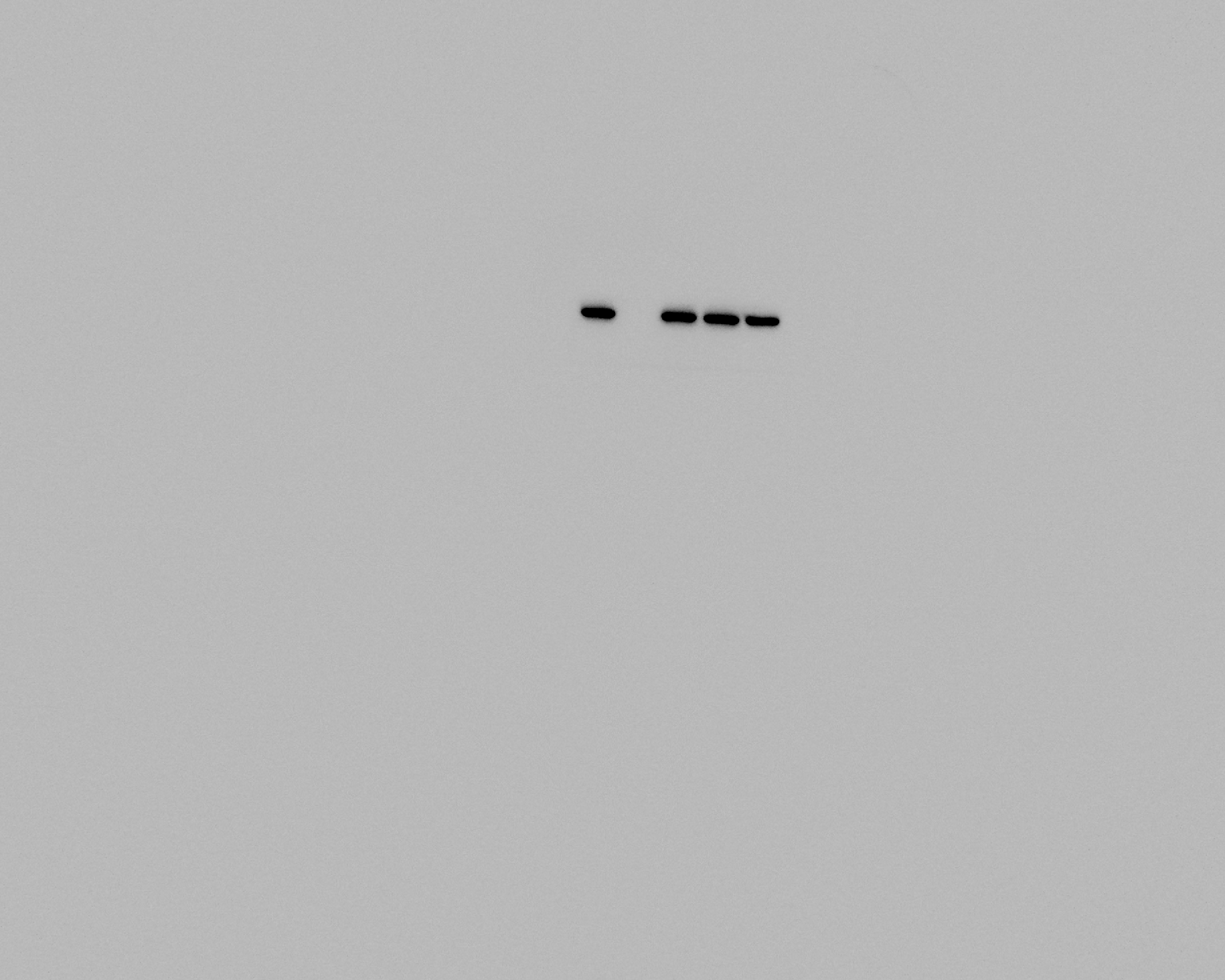

Supplement: Supplementary file 7 — Additional file 7. [file 12964_2024_1475_MOESM7_ESM.zip › Additional file 2/Figure 4M/Eca-109 oct4.tif]

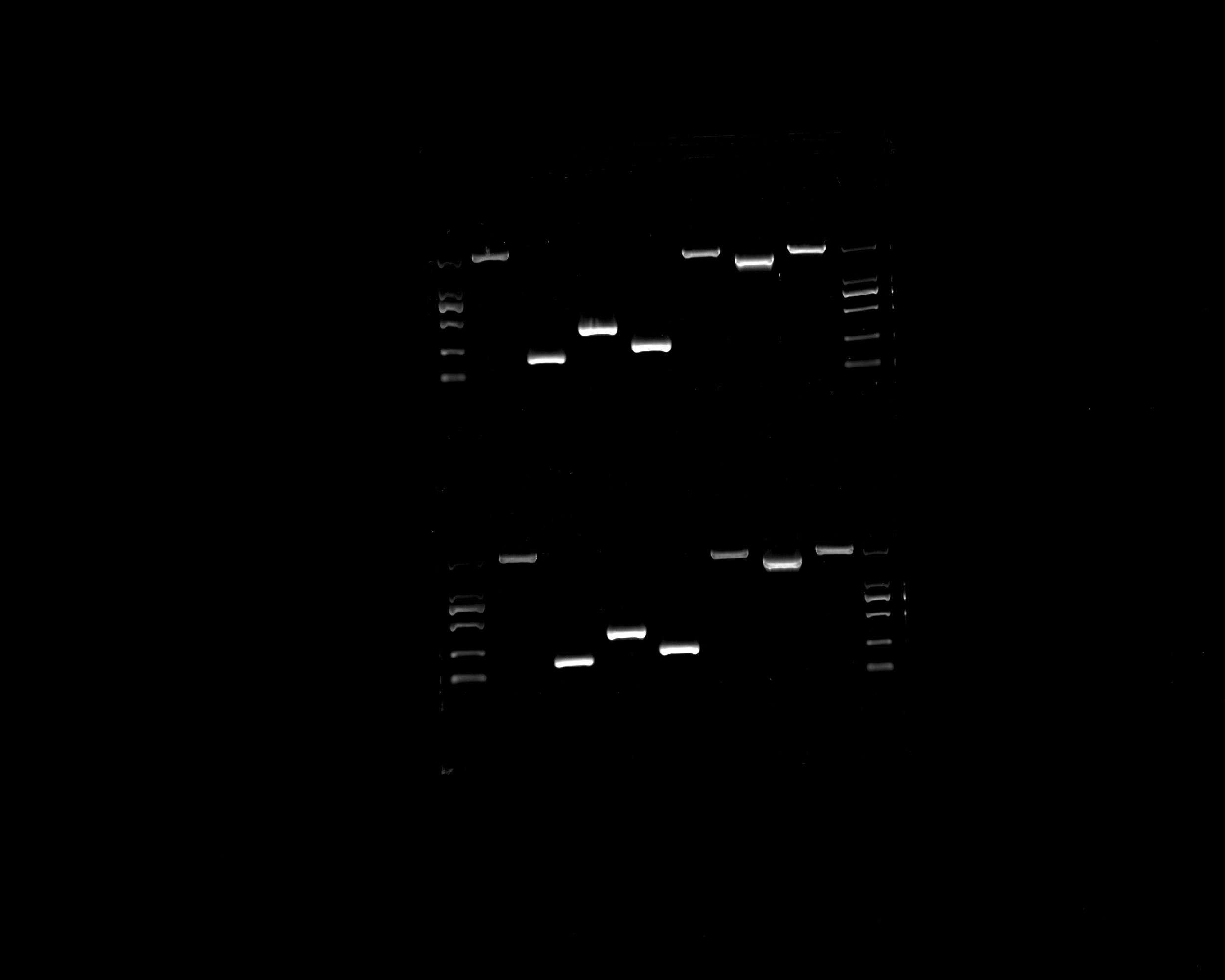

Supplement: Supplementary file 7 — Additional file 7. [file 12964_2024_1475_MOESM7_ESM.zip › Additional file 2/Figure 4M/GelGreen.tif]

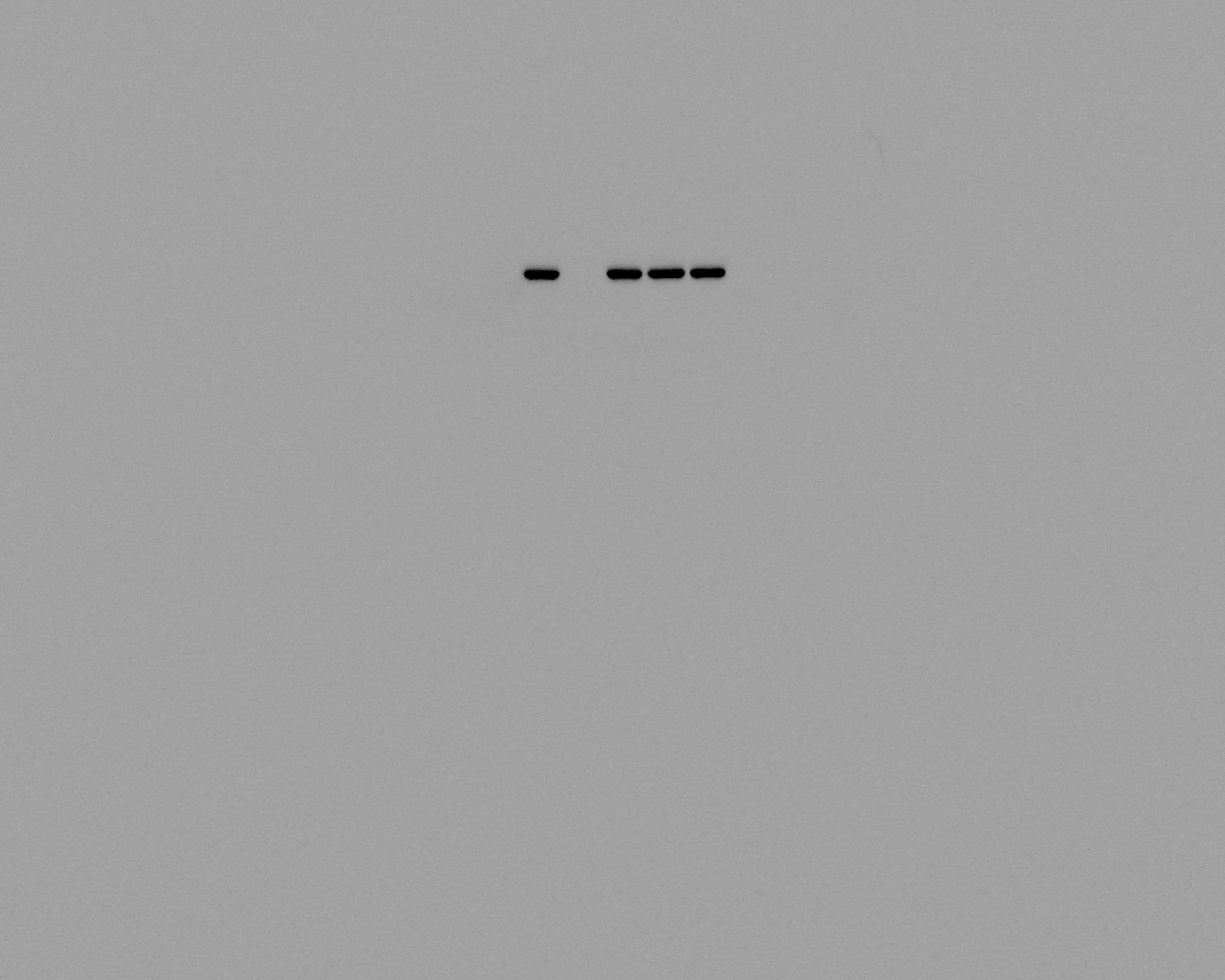

Supplement: Supplementary file 7 — Additional file 7. [file 12964_2024_1475_MOESM7_ESM.zip › Additional file 2/Figure 4M/KYSE-150 oct4.tif]

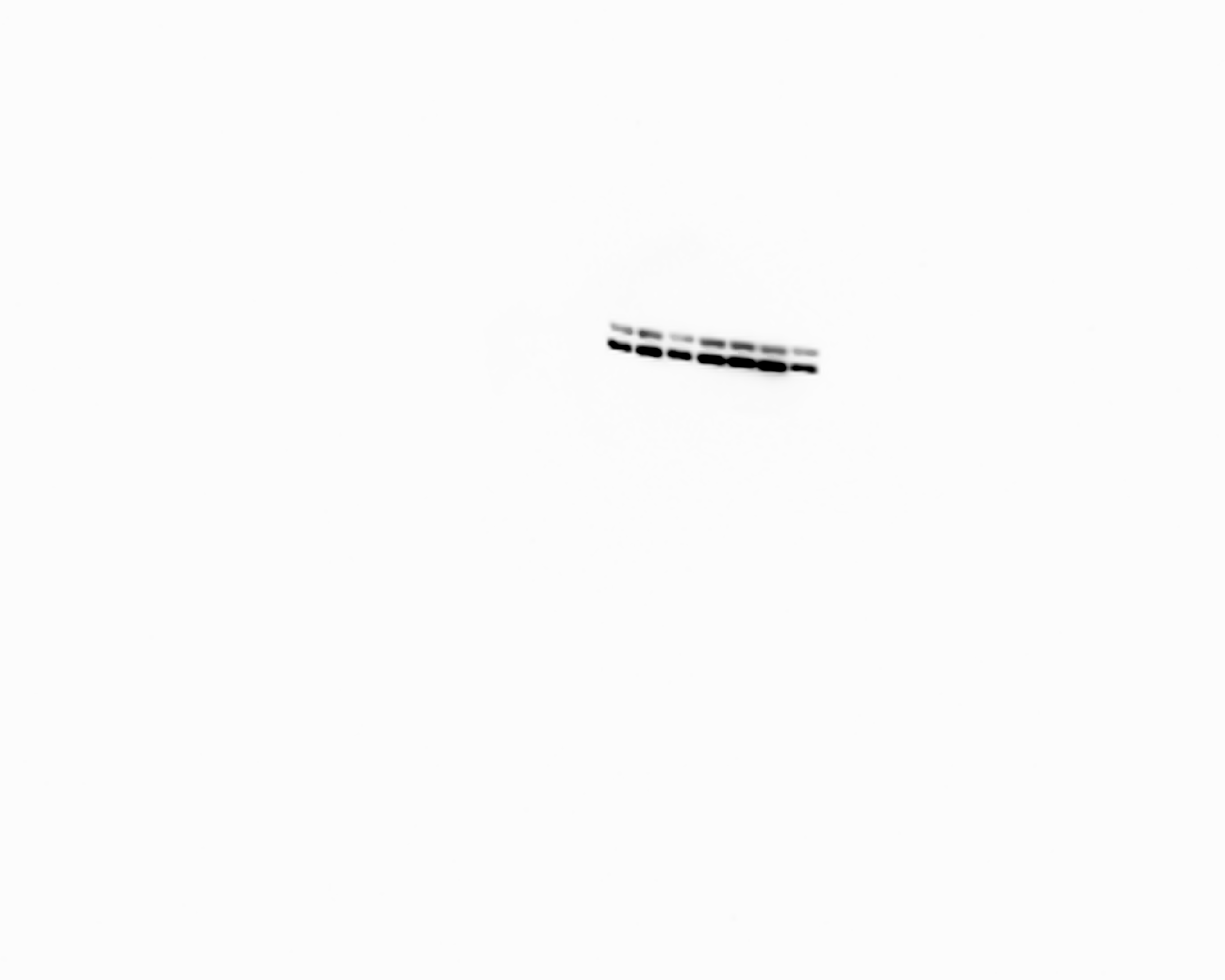

Supplement: Supplementary file 7 — Additional file 7. [file 12964_2024_1475_MOESM7_ESM.zip › Additional file 2/Figure 4O/KYSE-150/oct4.tif]

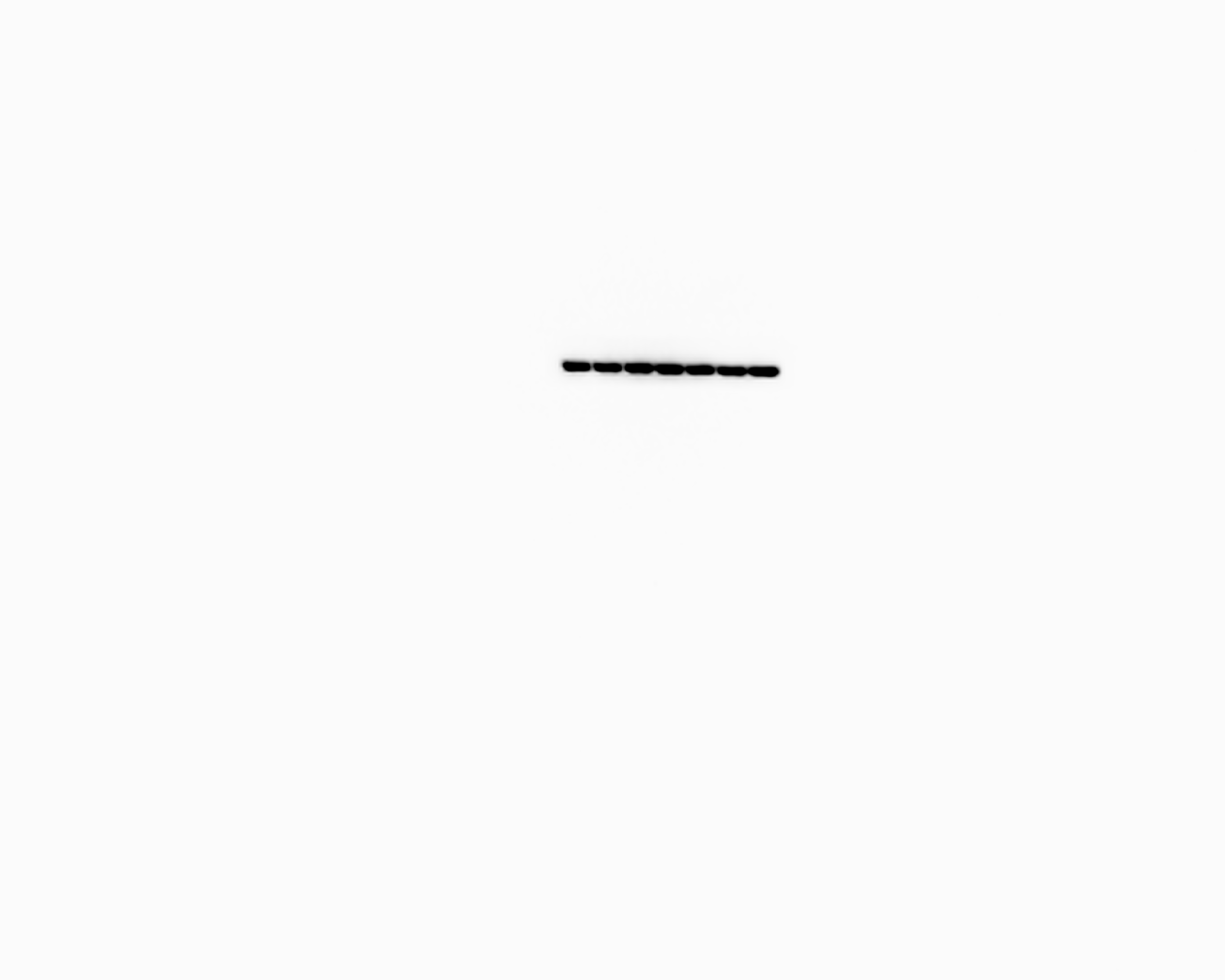

Supplement: Supplementary file 7 — Additional file 7. [file 12964_2024_1475_MOESM7_ESM.zip › Additional file 2/Figure 4O/KYSE-150/a┬-actin.tif]

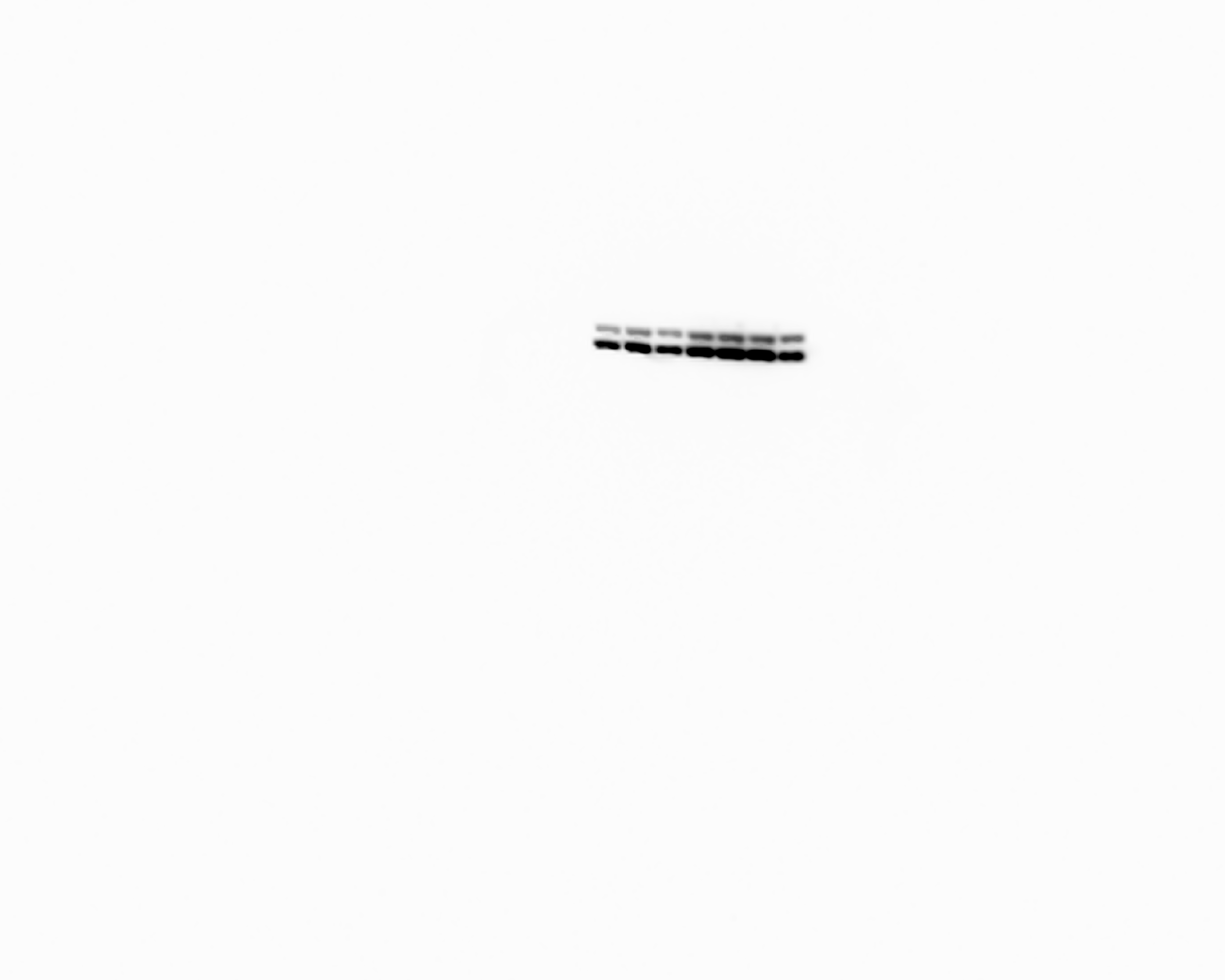

Supplement: Supplementary file 7 — Additional file 7. [file 12964_2024_1475_MOESM7_ESM.zip › Additional file 2/Figure 4O/KYSE-30/oct4.tif]

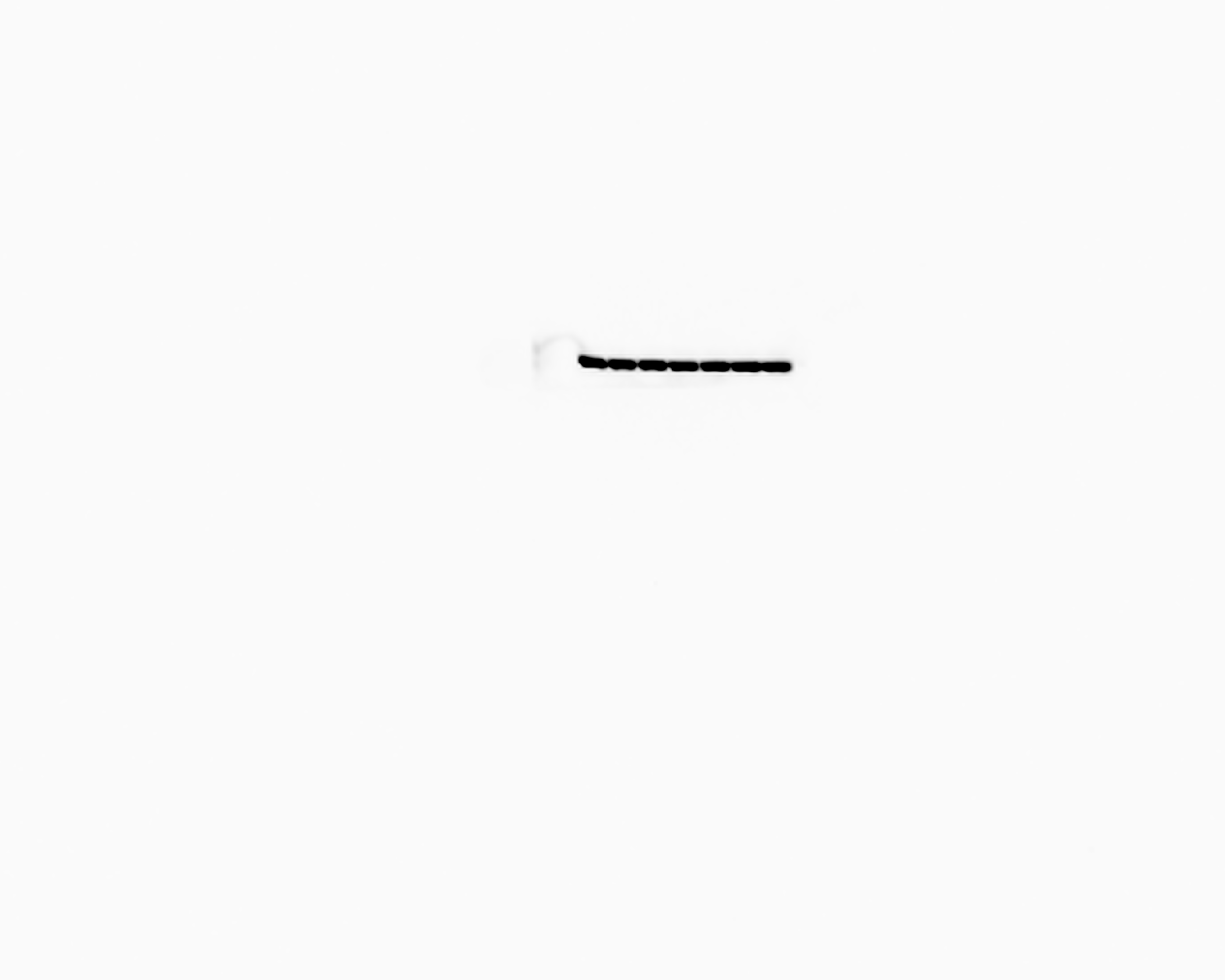

Supplement: Supplementary file 7 — Additional file 7. [file 12964_2024_1475_MOESM7_ESM.zip › Additional file 2/Figure 4O/KYSE-30/a┬-actin.tif]

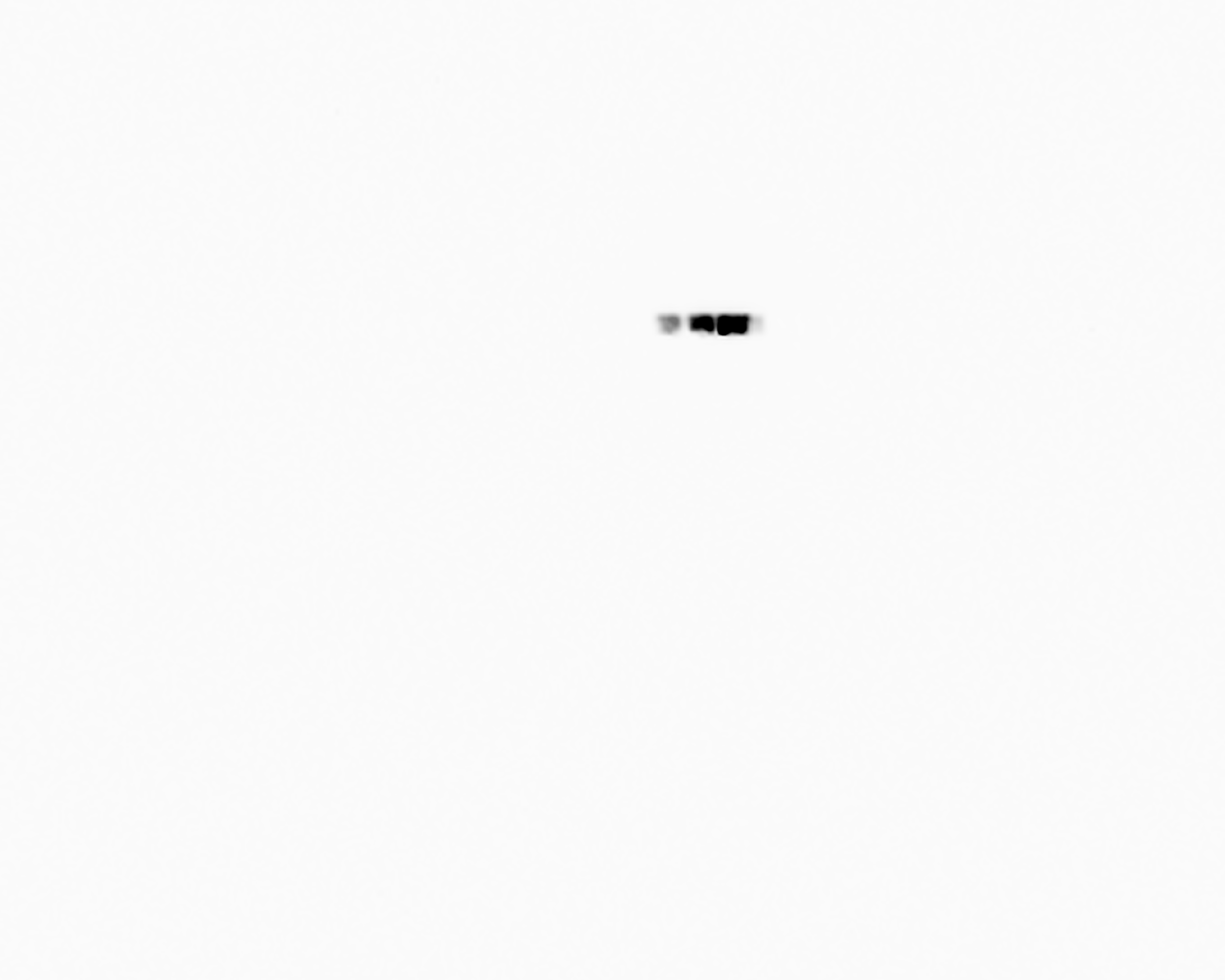

Supplement: Supplementary file 7 — Additional file 7. [file 12964_2024_1475_MOESM7_ESM.zip › Additional file 2/Figure 5B/KYSE-150/ITCH.tif]

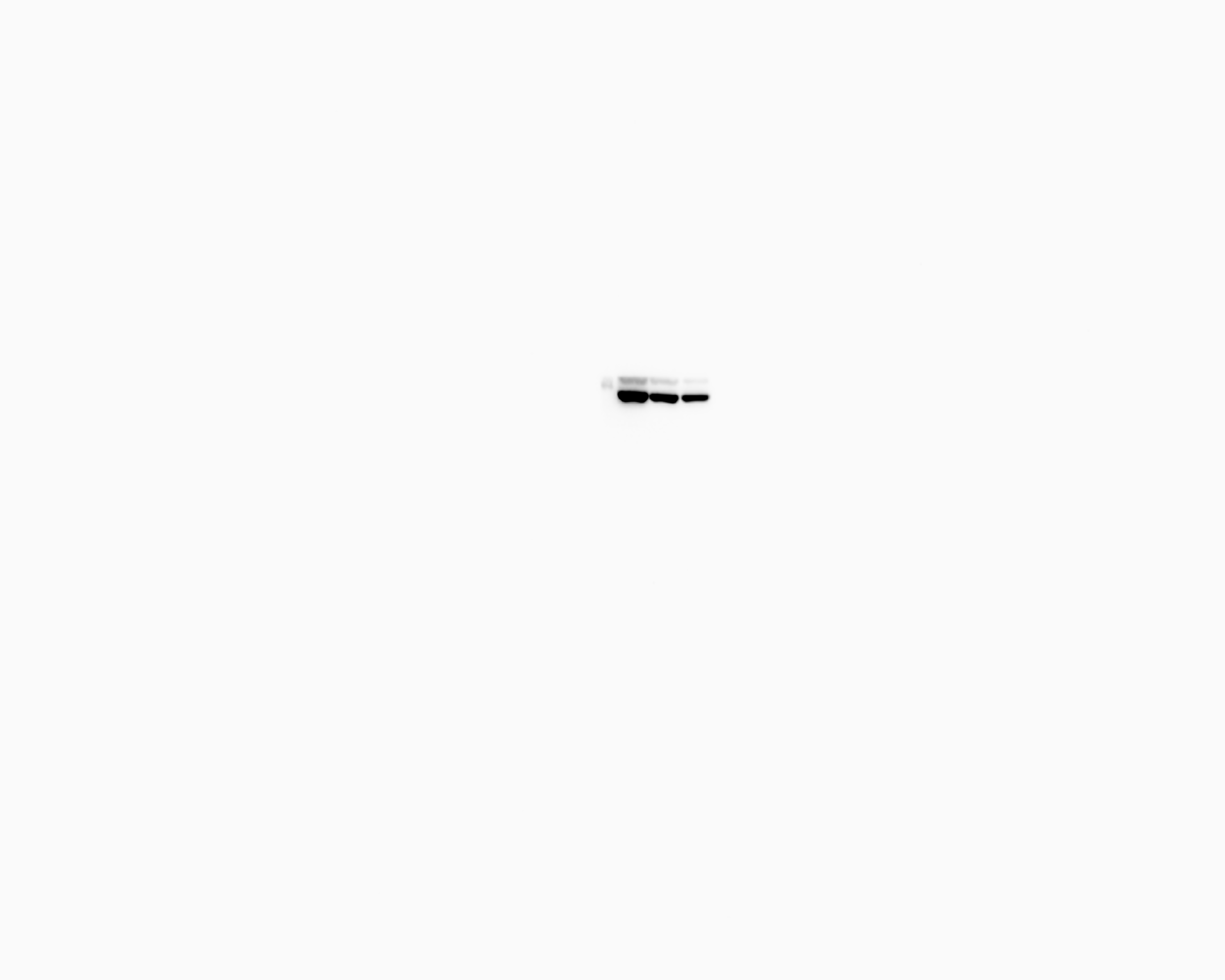

Supplement: Supplementary file 7 — Additional file 7. [file 12964_2024_1475_MOESM7_ESM.zip › Additional file 2/Figure 5B/KYSE-150/oct4.tif]

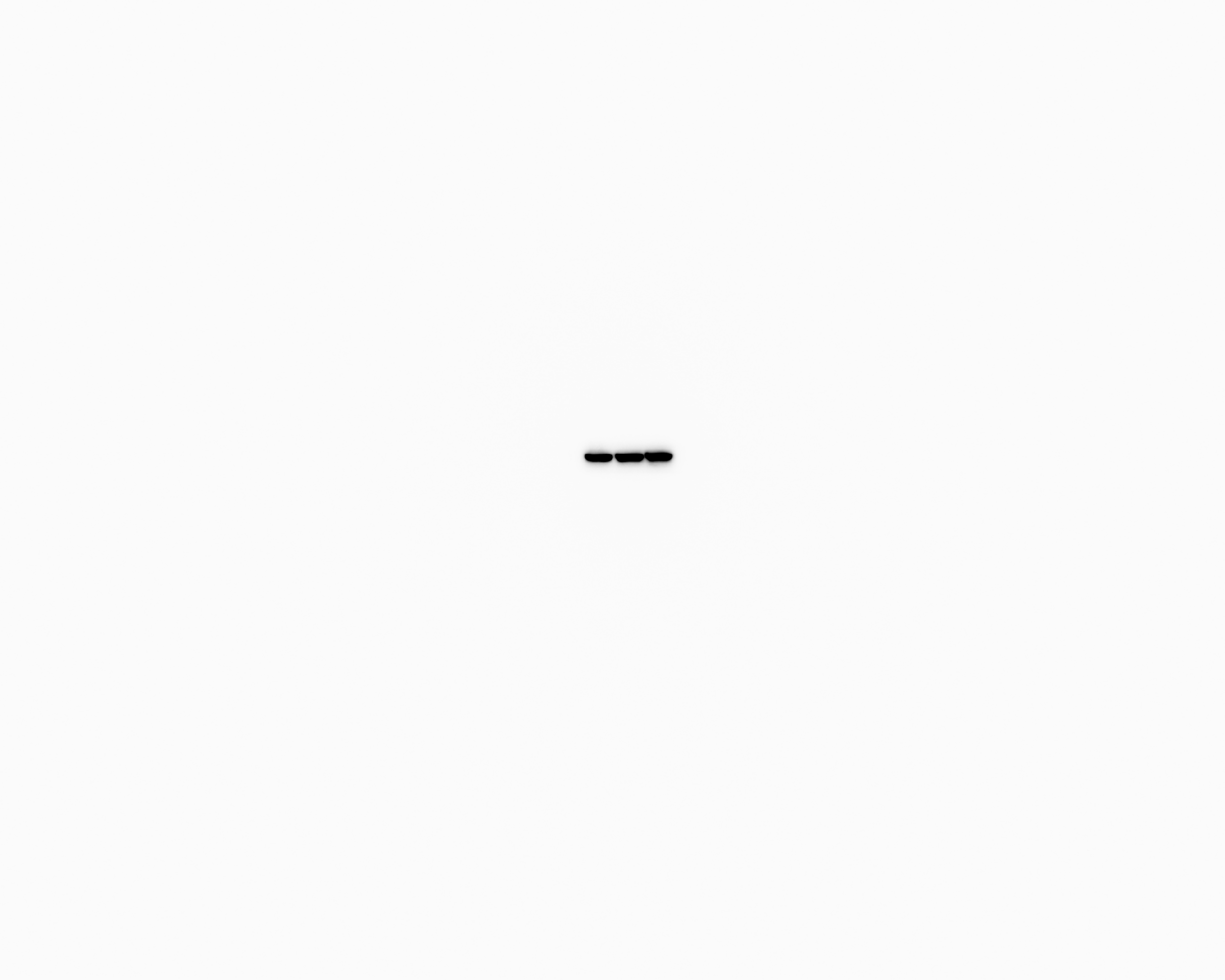

Supplement: Supplementary file 7 — Additional file 7. [file 12964_2024_1475_MOESM7_ESM.zip › Additional file 2/Figure 5B/KYSE-150/a┬-actin.tif]

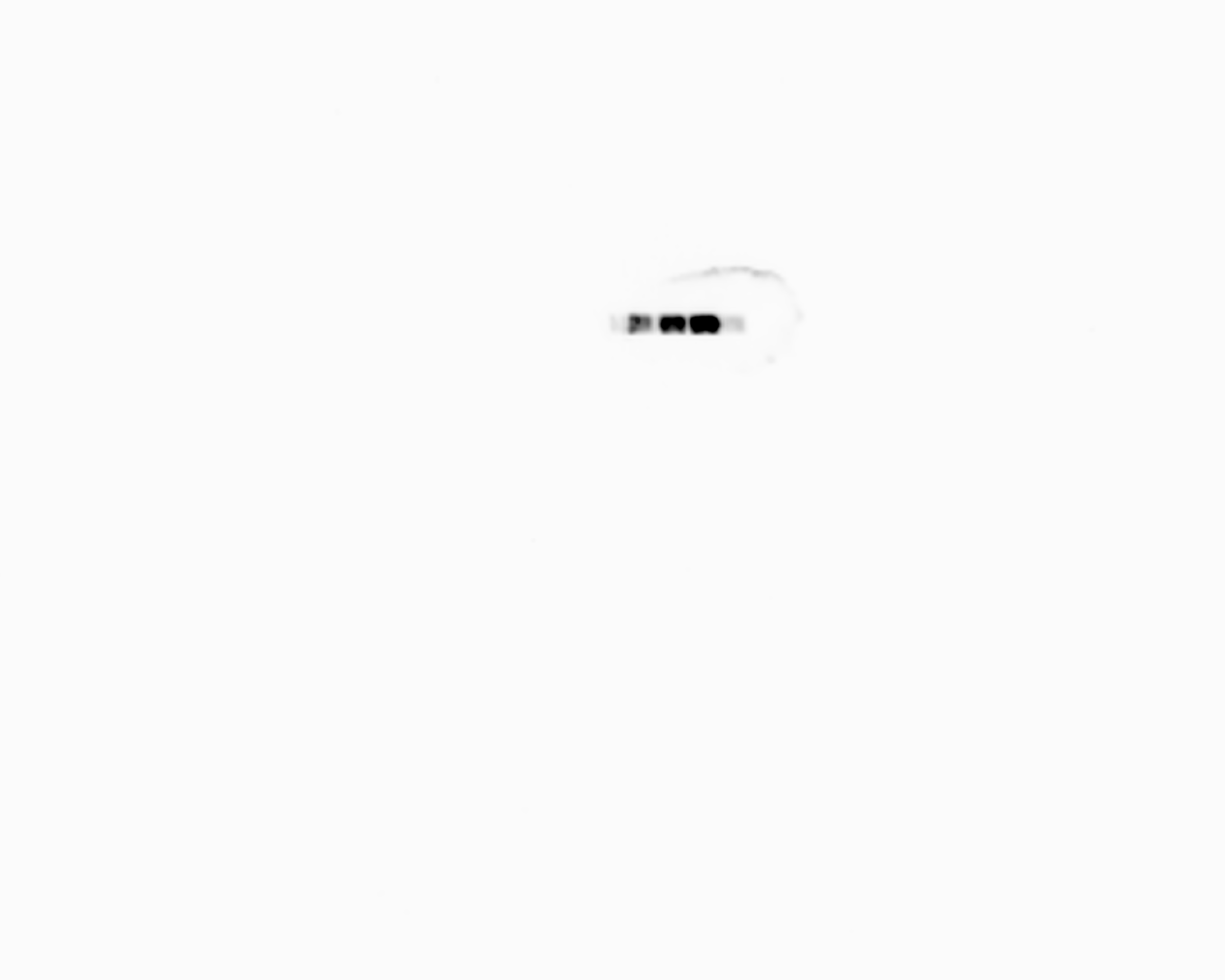

Supplement: Supplementary file 7 — Additional file 7. [file 12964_2024_1475_MOESM7_ESM.zip › Additional file 2/Figure 5B/KYSE-30/ITCH.tif]

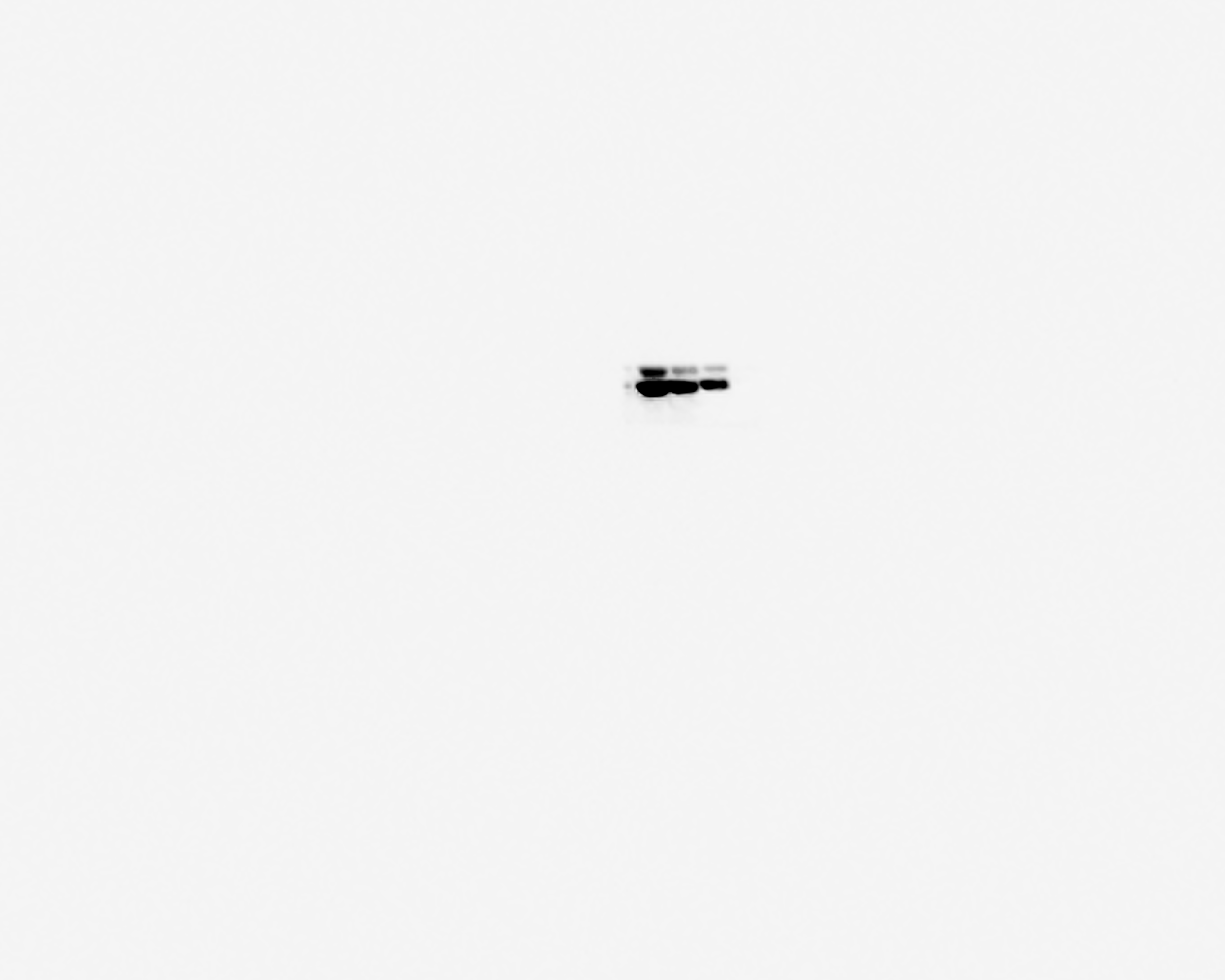

Supplement: Supplementary file 7 — Additional file 7. [file 12964_2024_1475_MOESM7_ESM.zip › Additional file 2/Figure 5B/KYSE-30/oct4.tif]

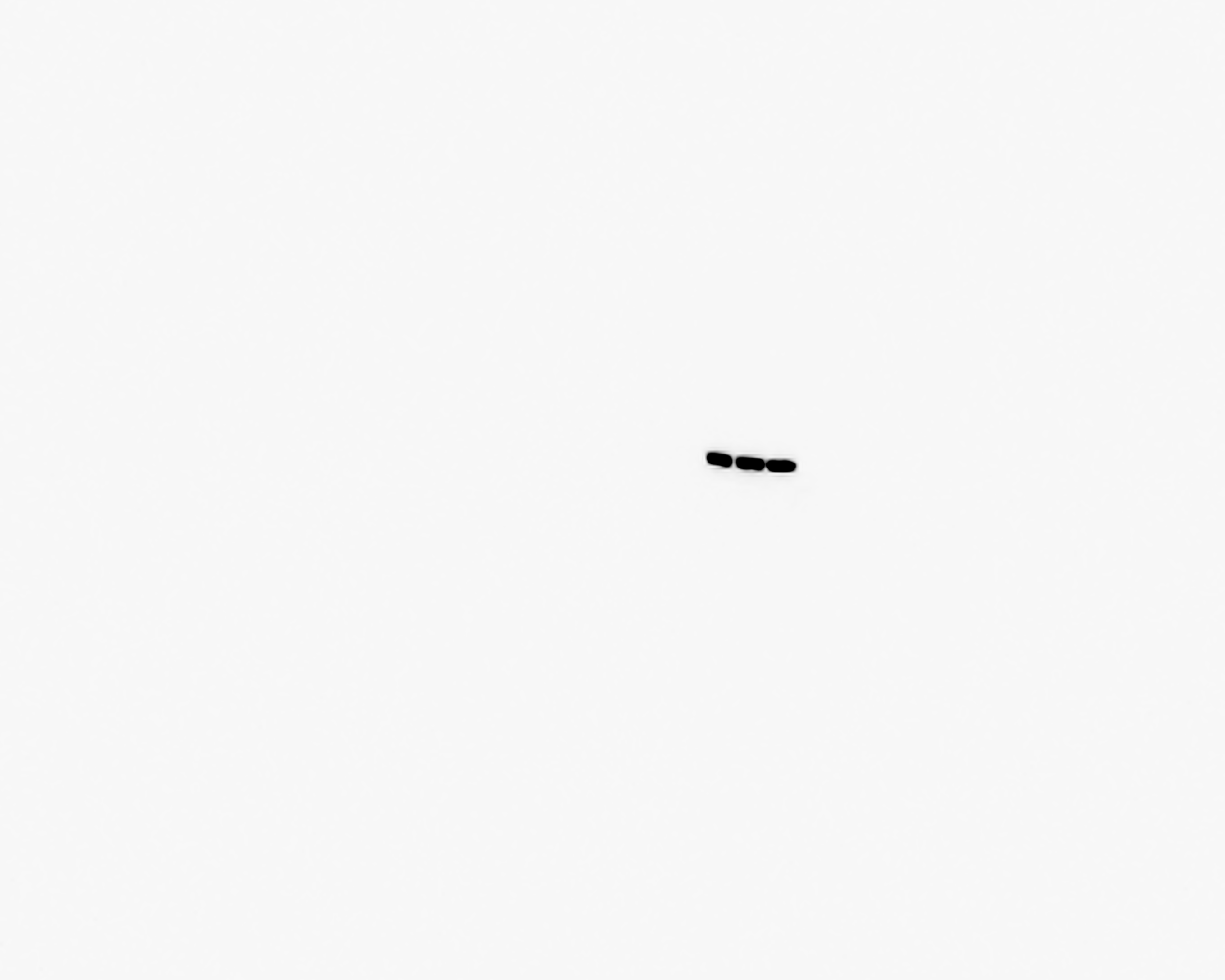

Supplement: Supplementary file 7 — Additional file 7. [file 12964_2024_1475_MOESM7_ESM.zip › Additional file 2/Figure 5B/KYSE-30/a┬-actin.tif]

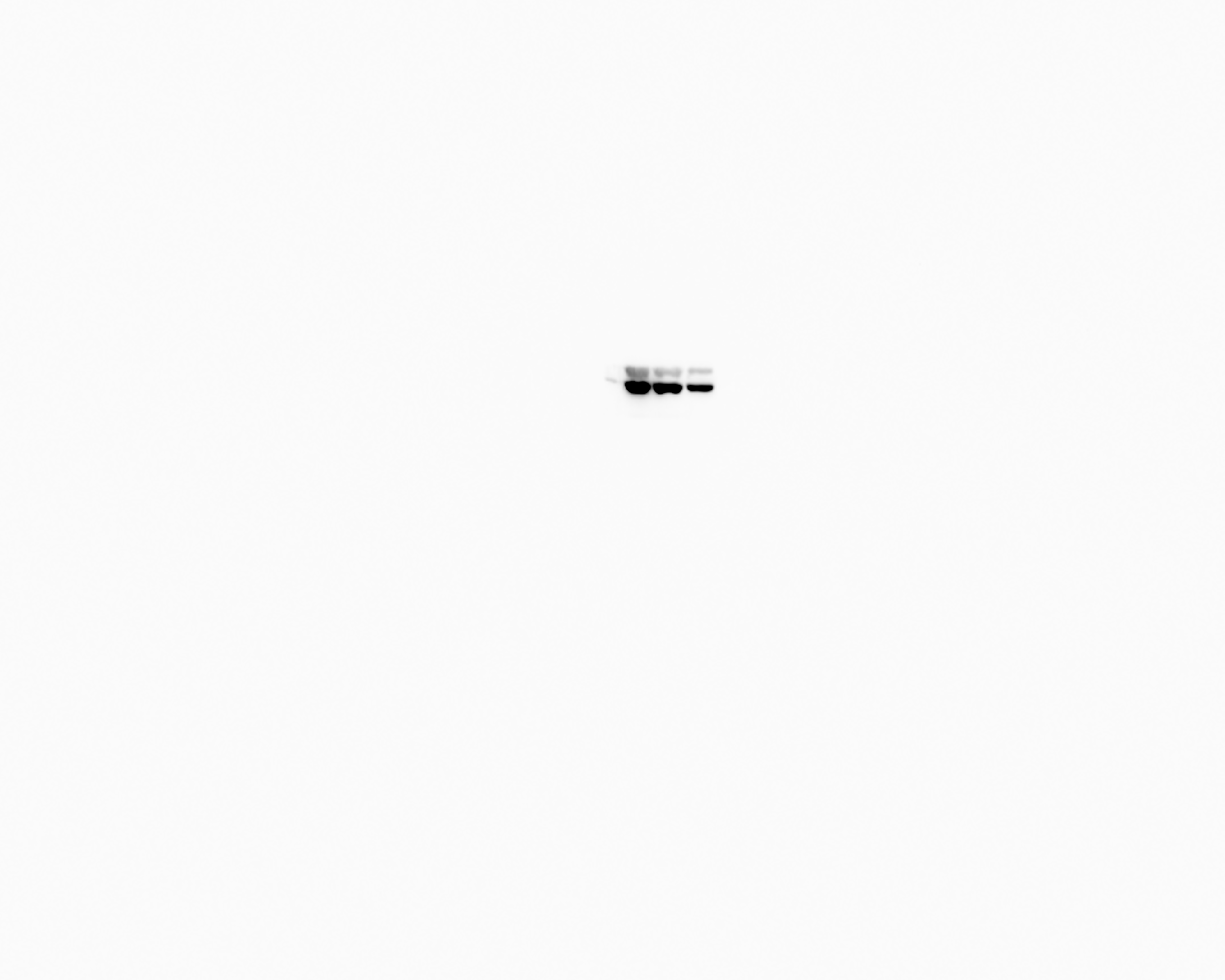

Supplement: Supplementary file 7 — Additional file 7. [file 12964_2024_1475_MOESM7_ESM.zip › Additional file 2/Figure 5C/KYSE-150/OCT4.tif]

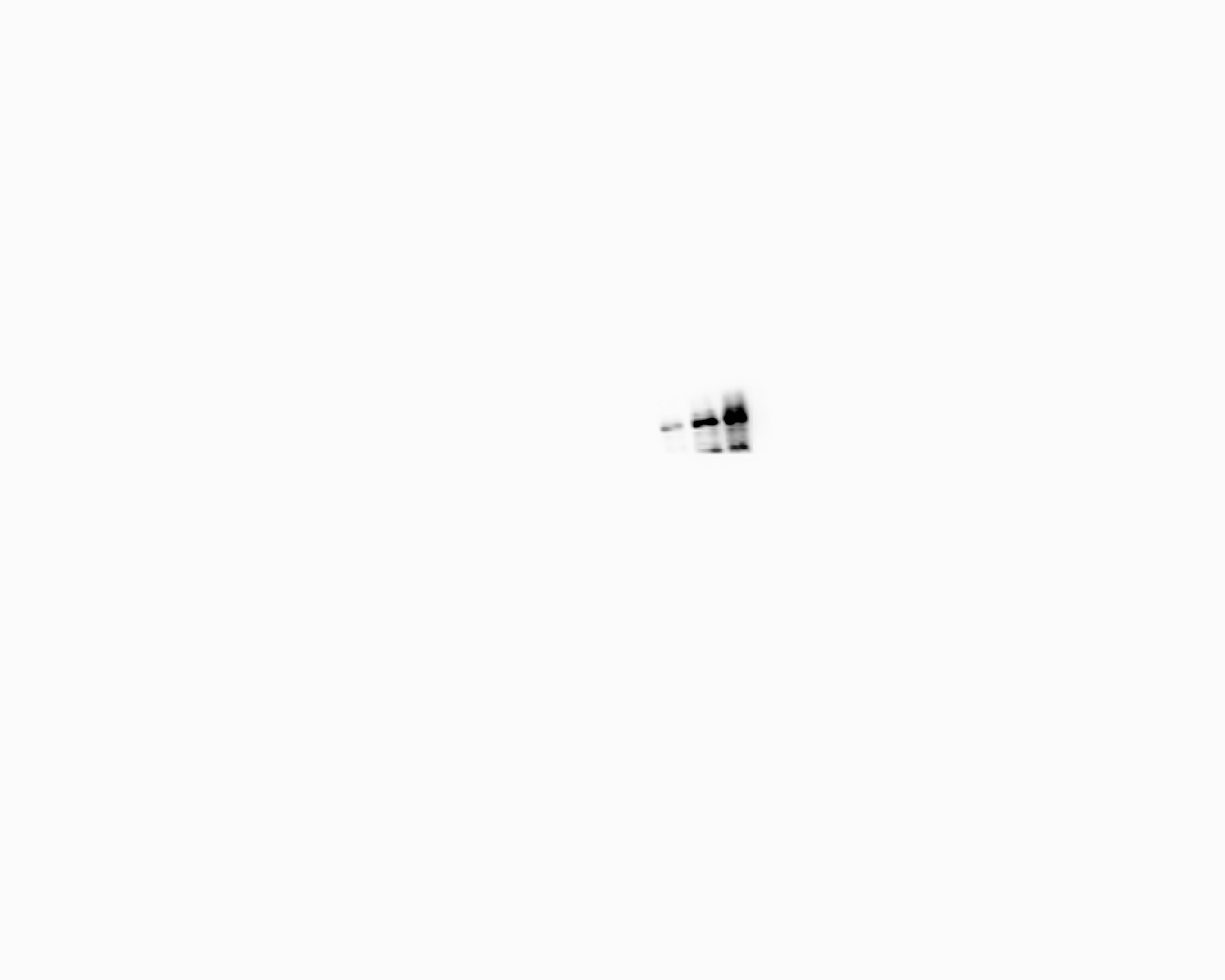

Supplement: Supplementary file 7 — Additional file 7. [file 12964_2024_1475_MOESM7_ESM.zip › Additional file 2/Figure 5C/KYSE-150/WWP2.tif]

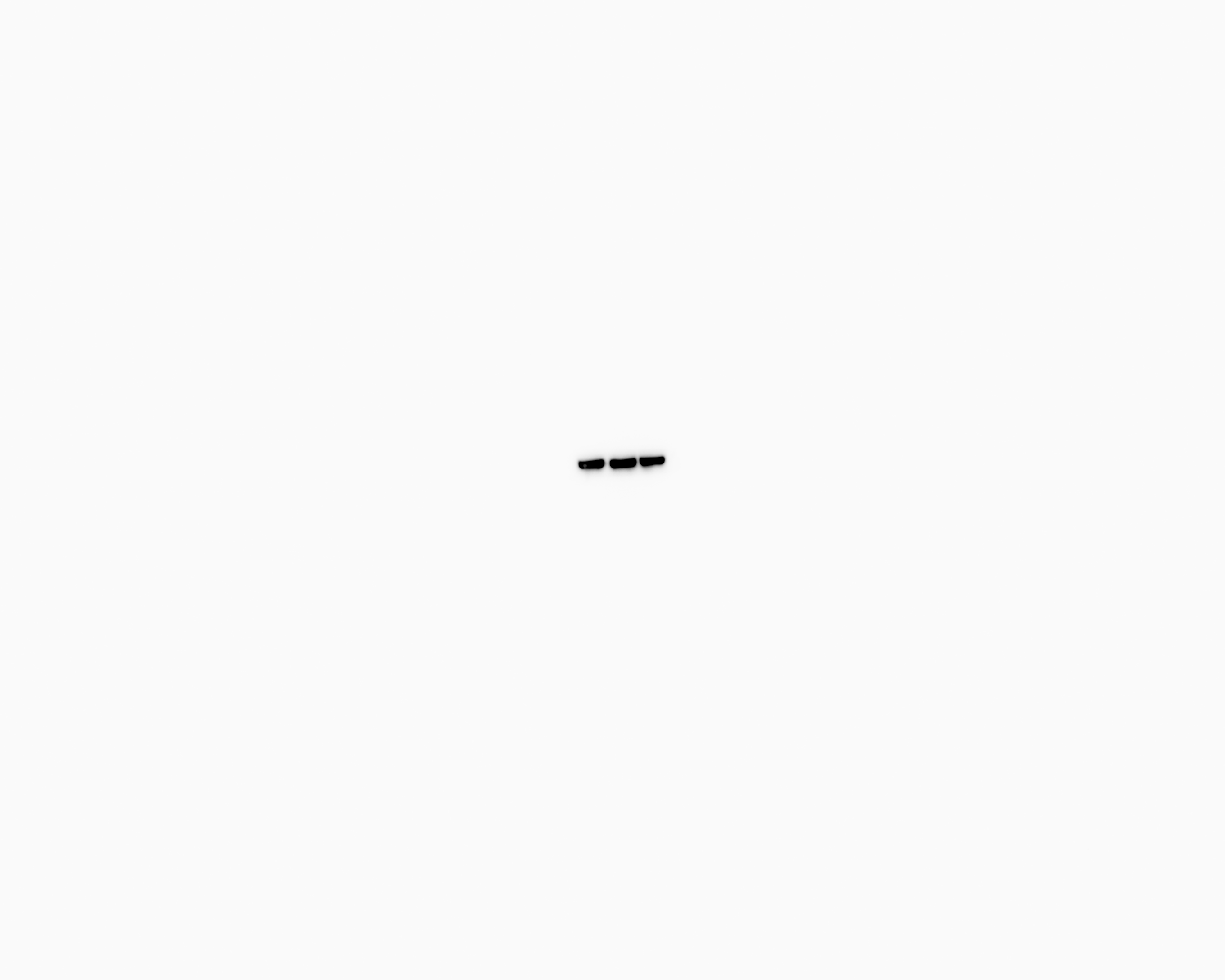

Supplement: Supplementary file 7 — Additional file 7. [file 12964_2024_1475_MOESM7_ESM.zip › Additional file 2/Figure 5C/KYSE-150/a┬-actin.tif]

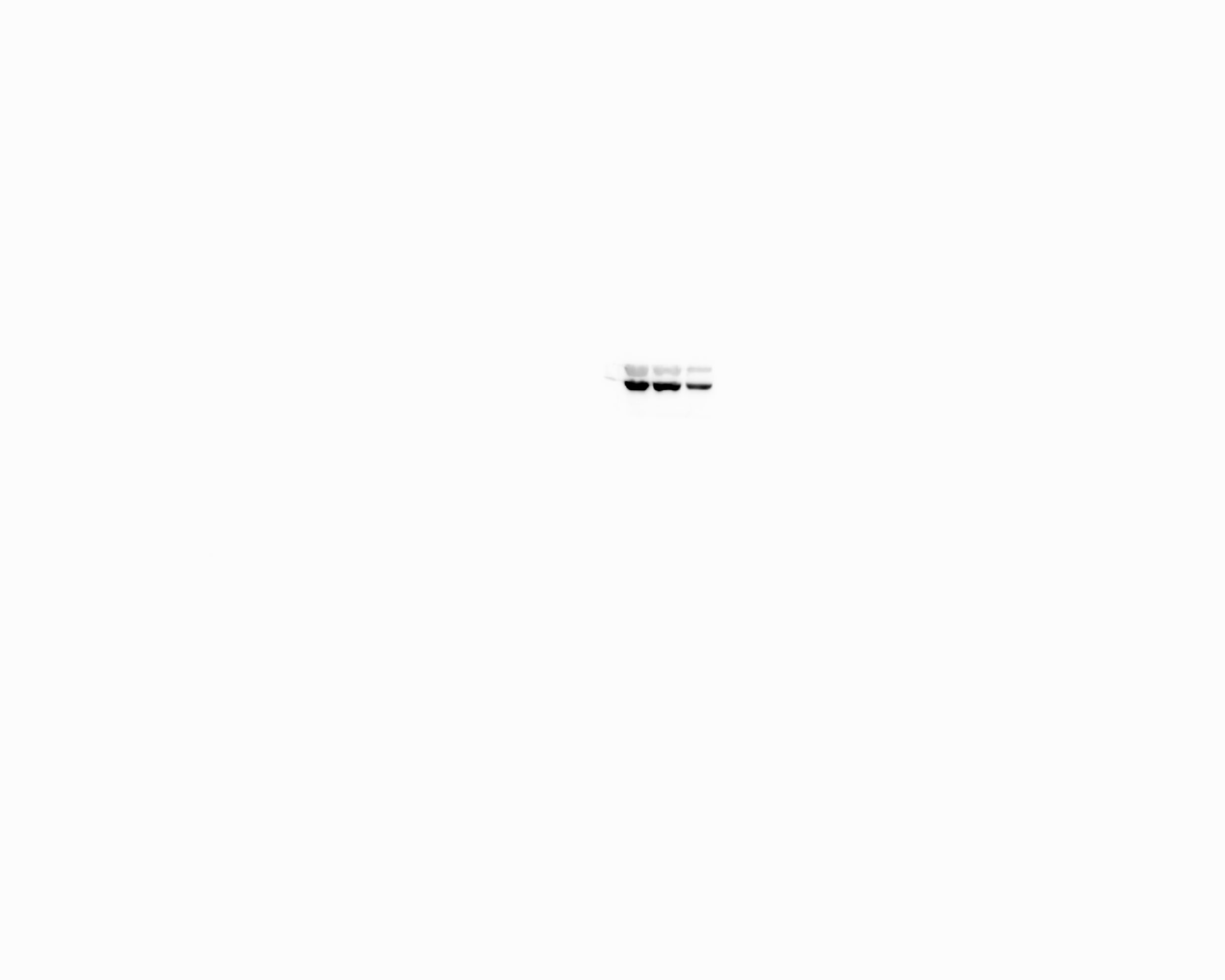

Supplement: Supplementary file 7 — Additional file 7. [file 12964_2024_1475_MOESM7_ESM.zip › Additional file 2/Figure 5C/KYSE-30/OCT4.tif]

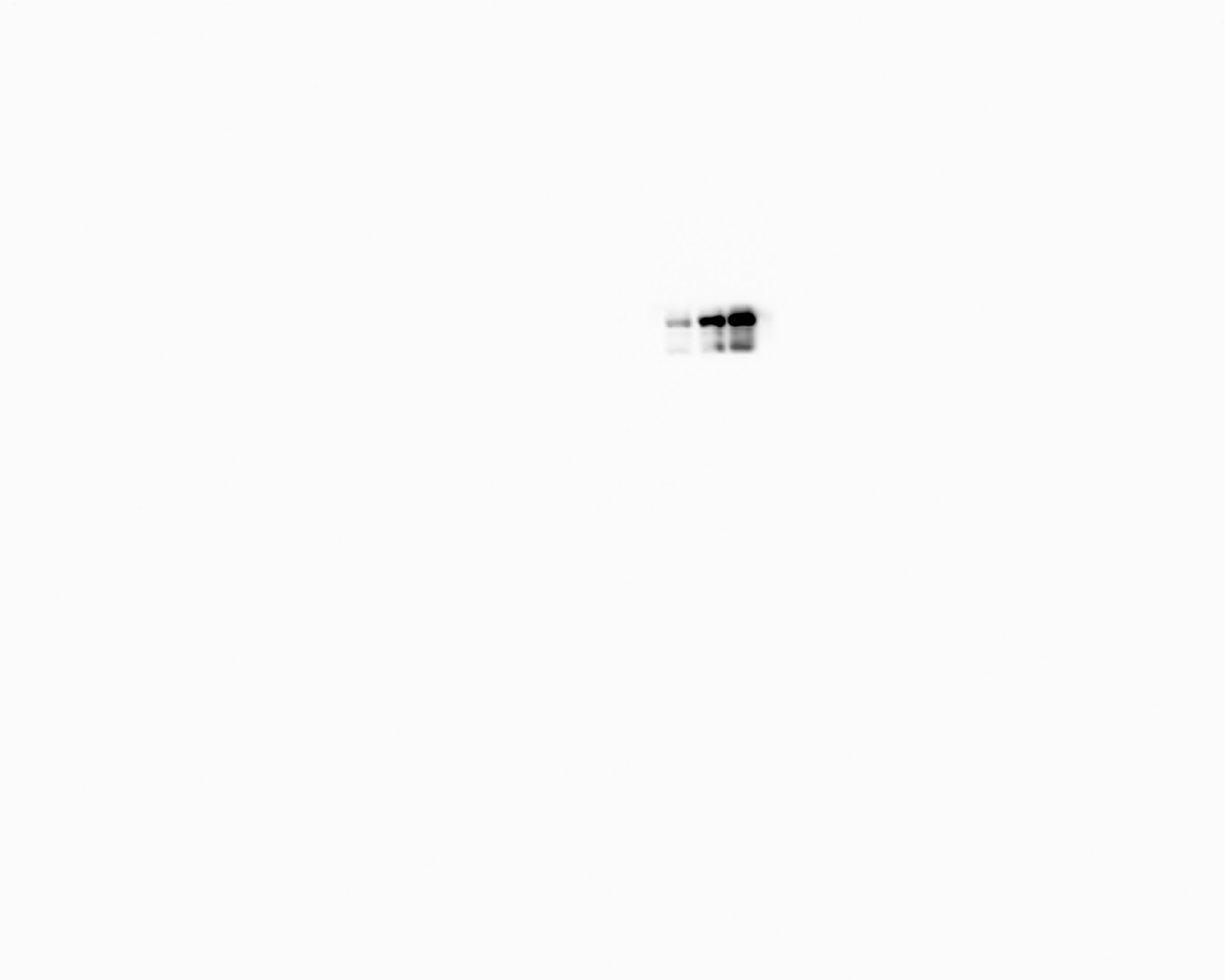

Supplement: Supplementary file 7 — Additional file 7. [file 12964_2024_1475_MOESM7_ESM.zip › Additional file 2/Figure 5C/KYSE-30/WWP2.tif]

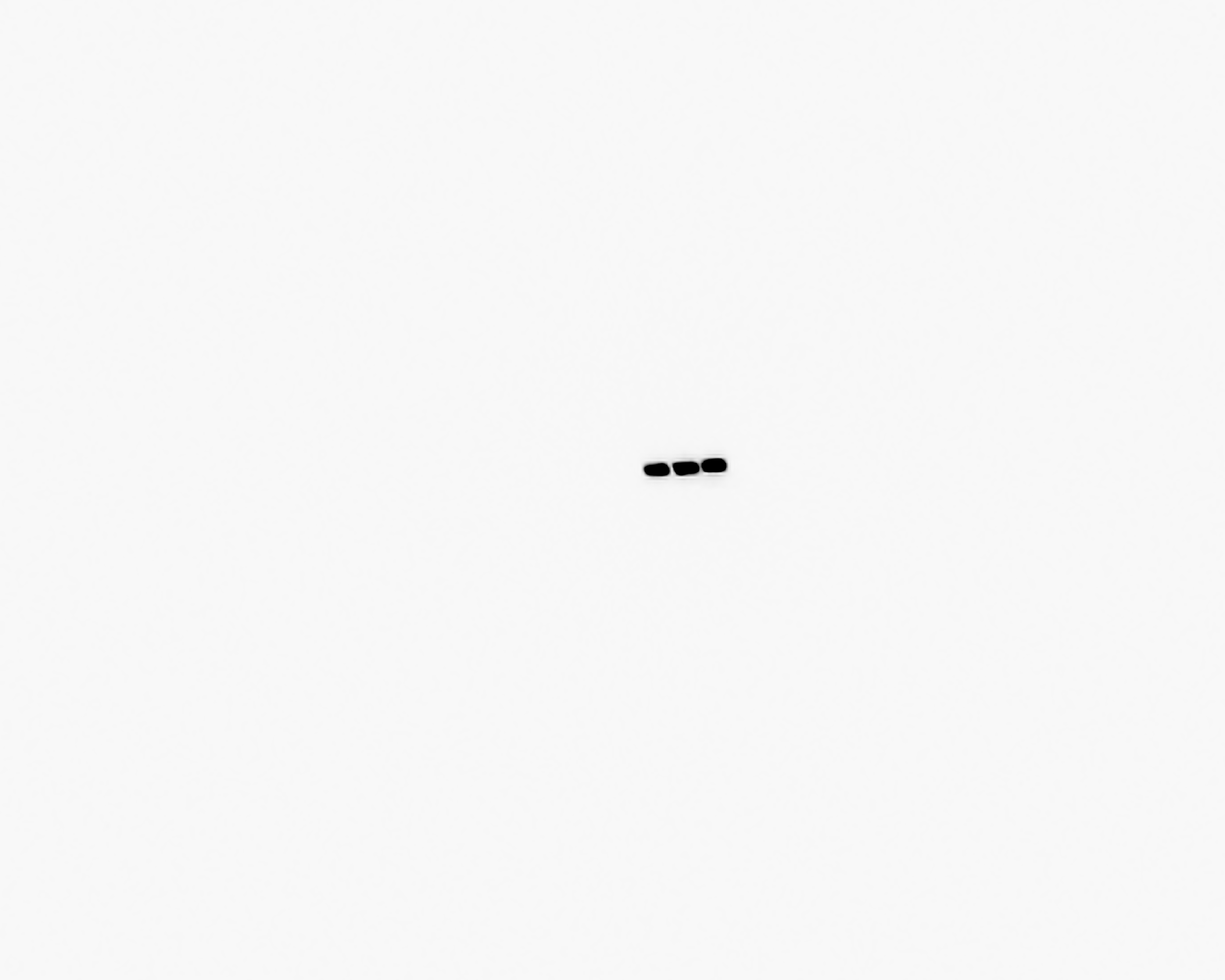

Supplement: Supplementary file 7 — Additional file 7. [file 12964_2024_1475_MOESM7_ESM.zip › Additional file 2/Figure 5C/KYSE-30/a┬-actin.tif]

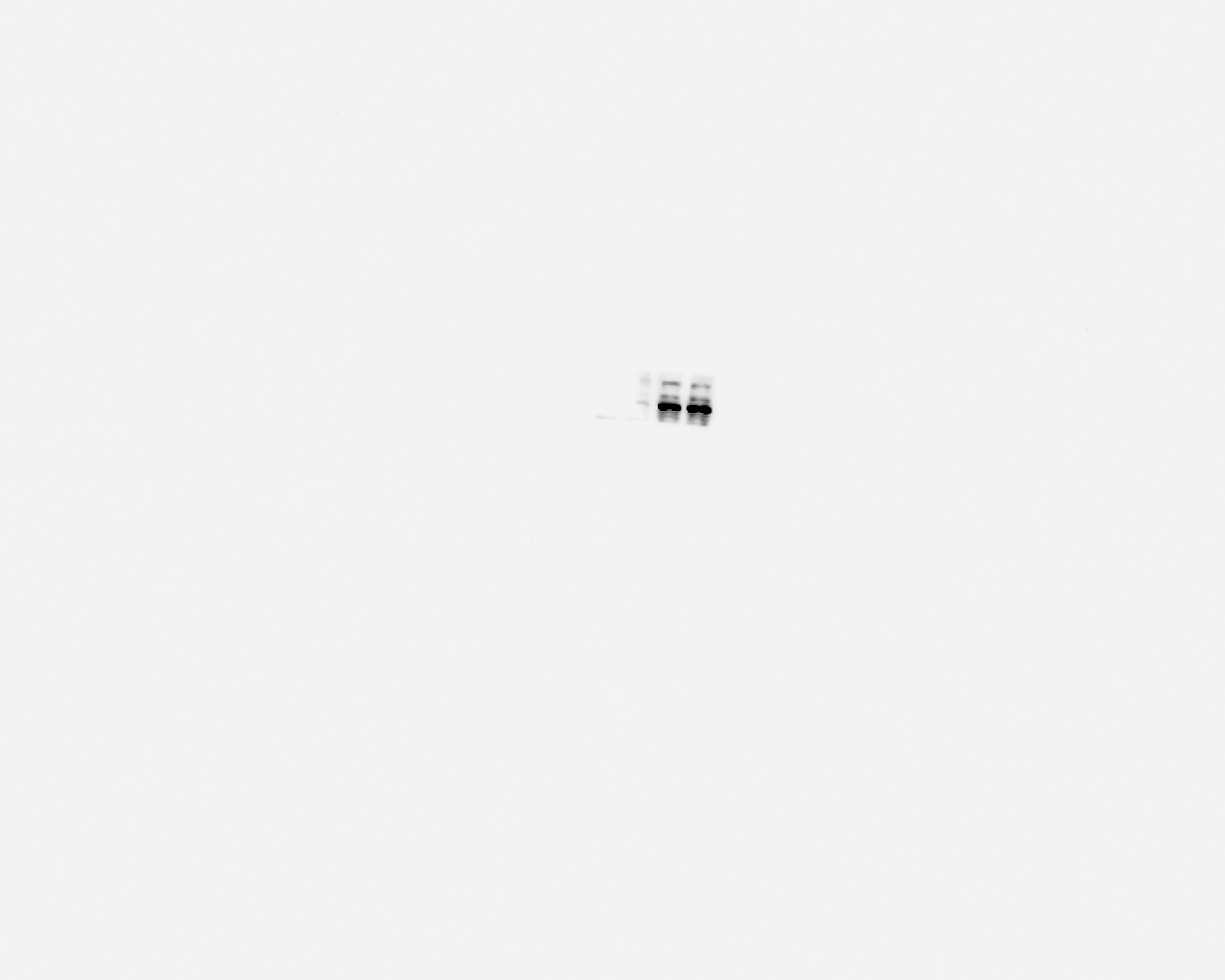

Supplement: Supplementary file 7 — Additional file 7. [file 12964_2024_1475_MOESM7_ESM.zip › Additional file 2/Figure 5D/KYSE-150/ITCH.tif]

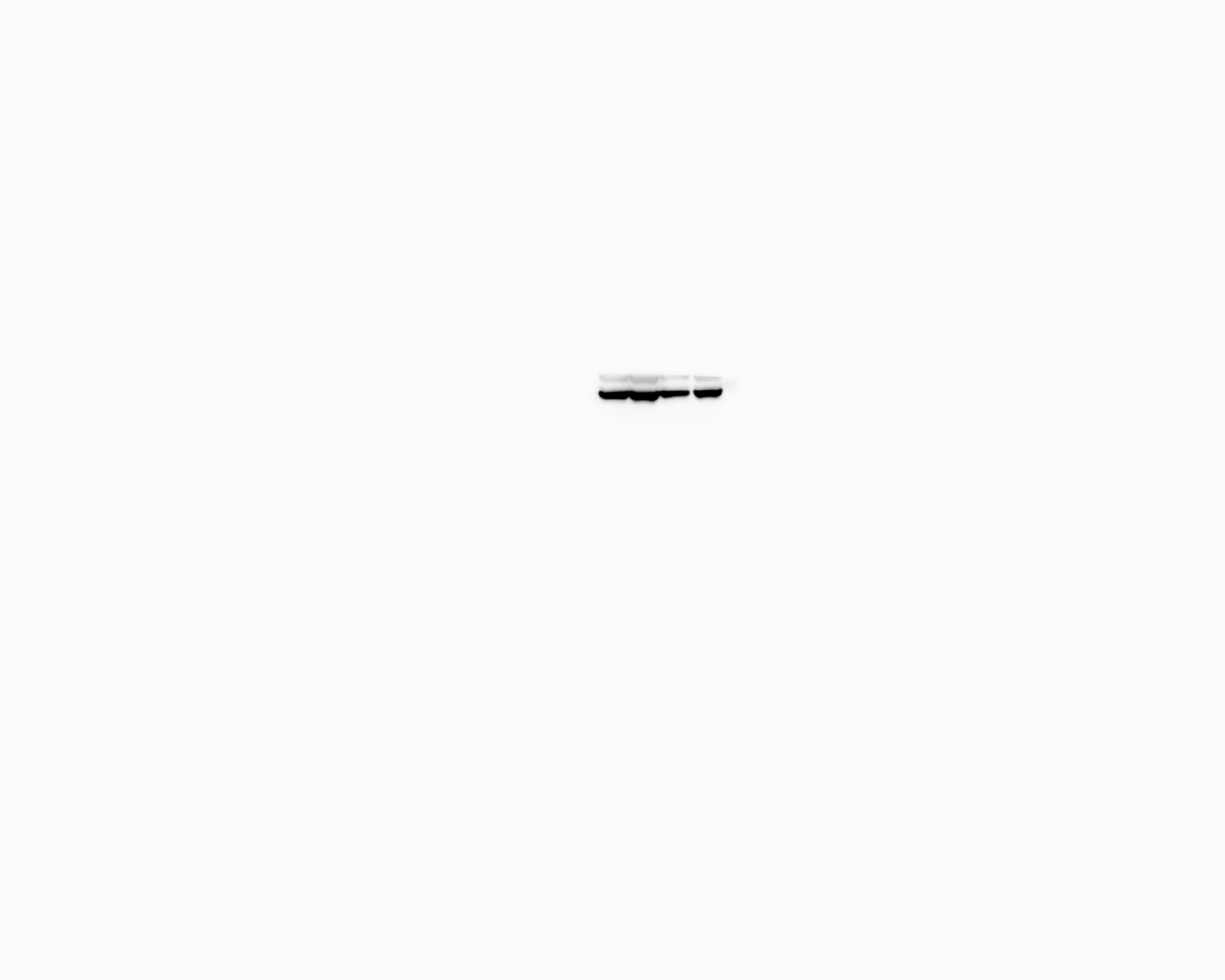

Supplement: Supplementary file 7 — Additional file 7. [file 12964_2024_1475_MOESM7_ESM.zip › Additional file 2/Figure 5D/KYSE-150/OCT4.tif]

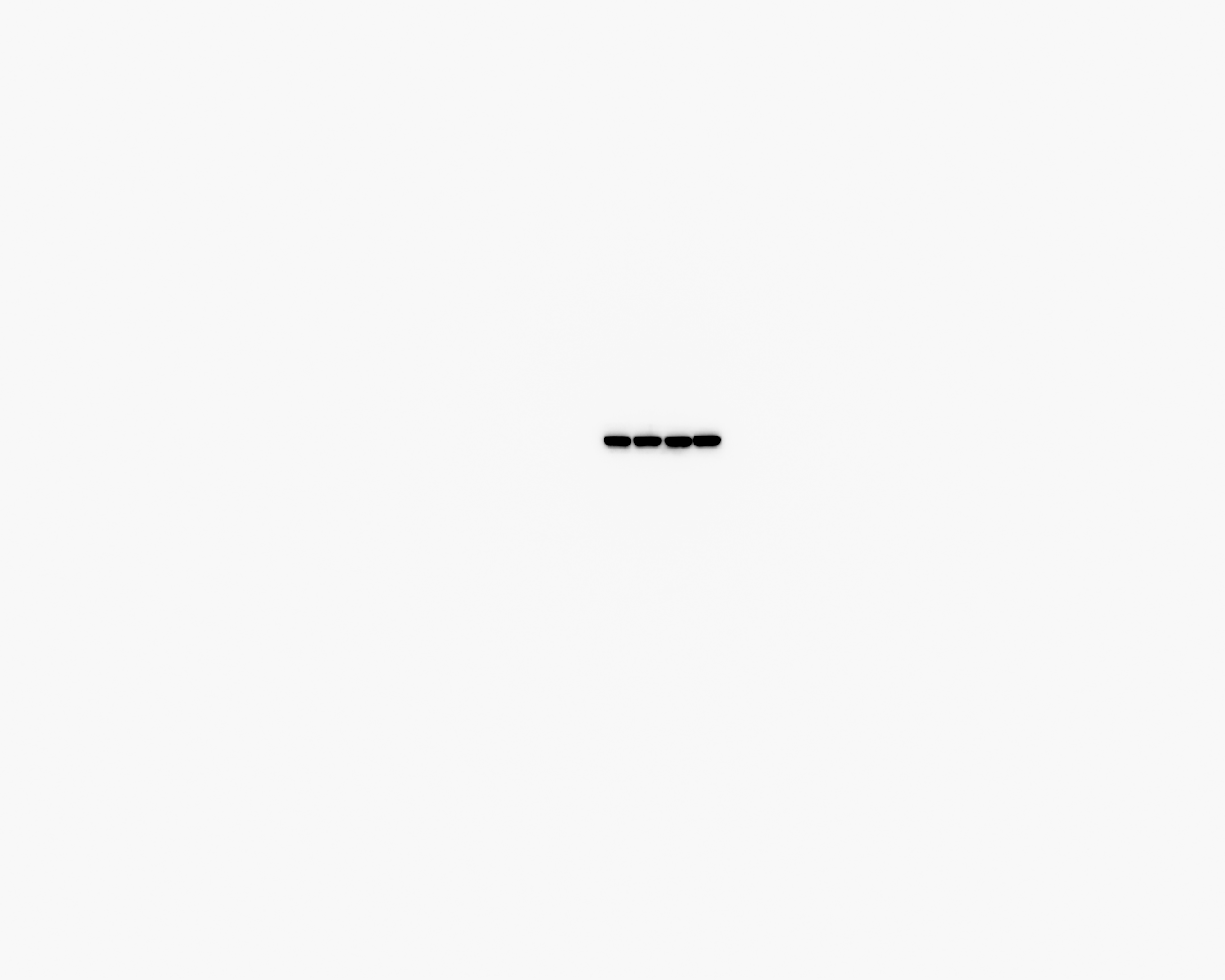

Supplement: Supplementary file 7 — Additional file 7. [file 12964_2024_1475_MOESM7_ESM.zip › Additional file 2/Figure 5D/KYSE-150/a┬-actin.tif]

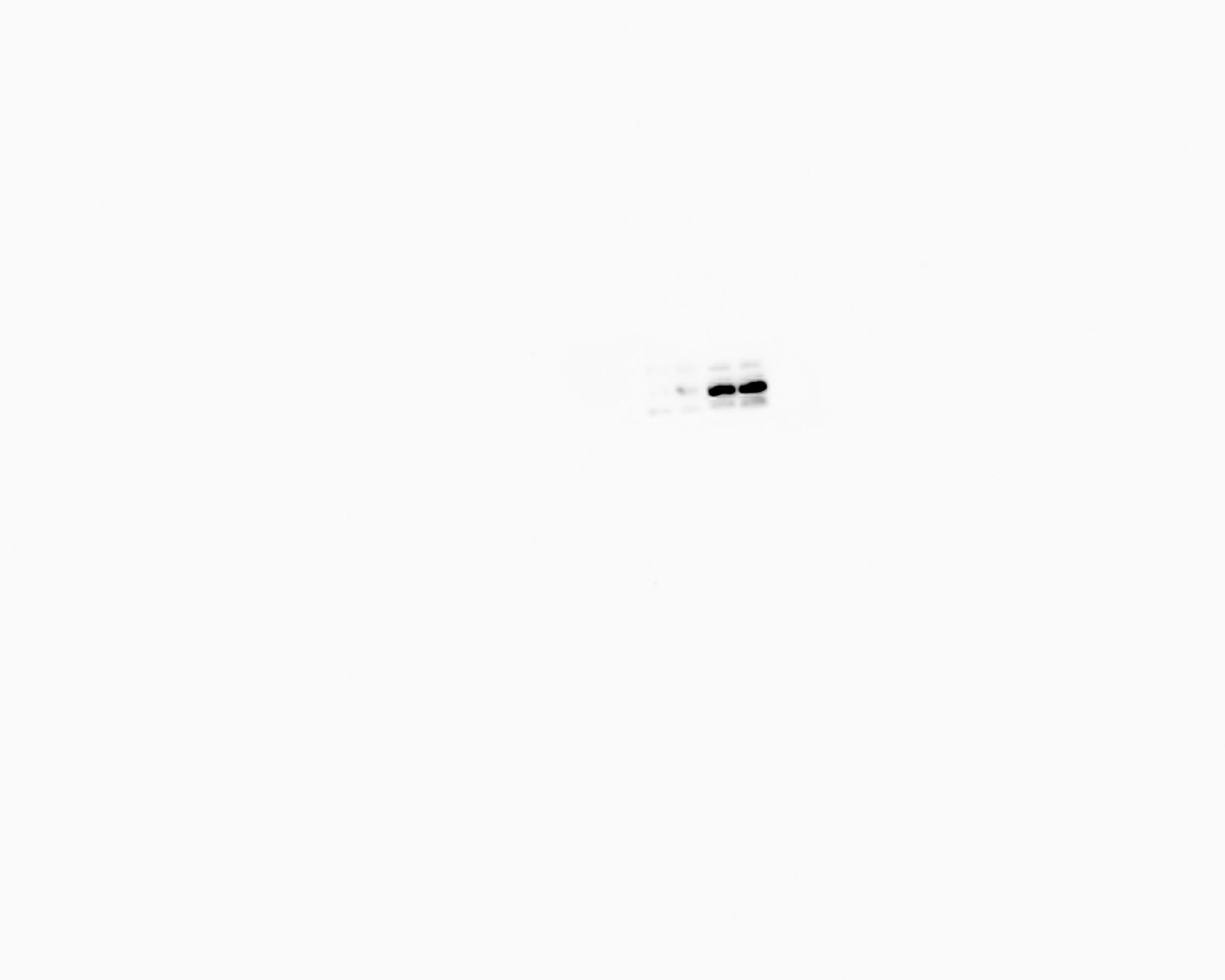

Supplement: Supplementary file 7 — Additional file 7. [file 12964_2024_1475_MOESM7_ESM.zip › Additional file 2/Figure 5D/KYSE-30/ITCH.tif]

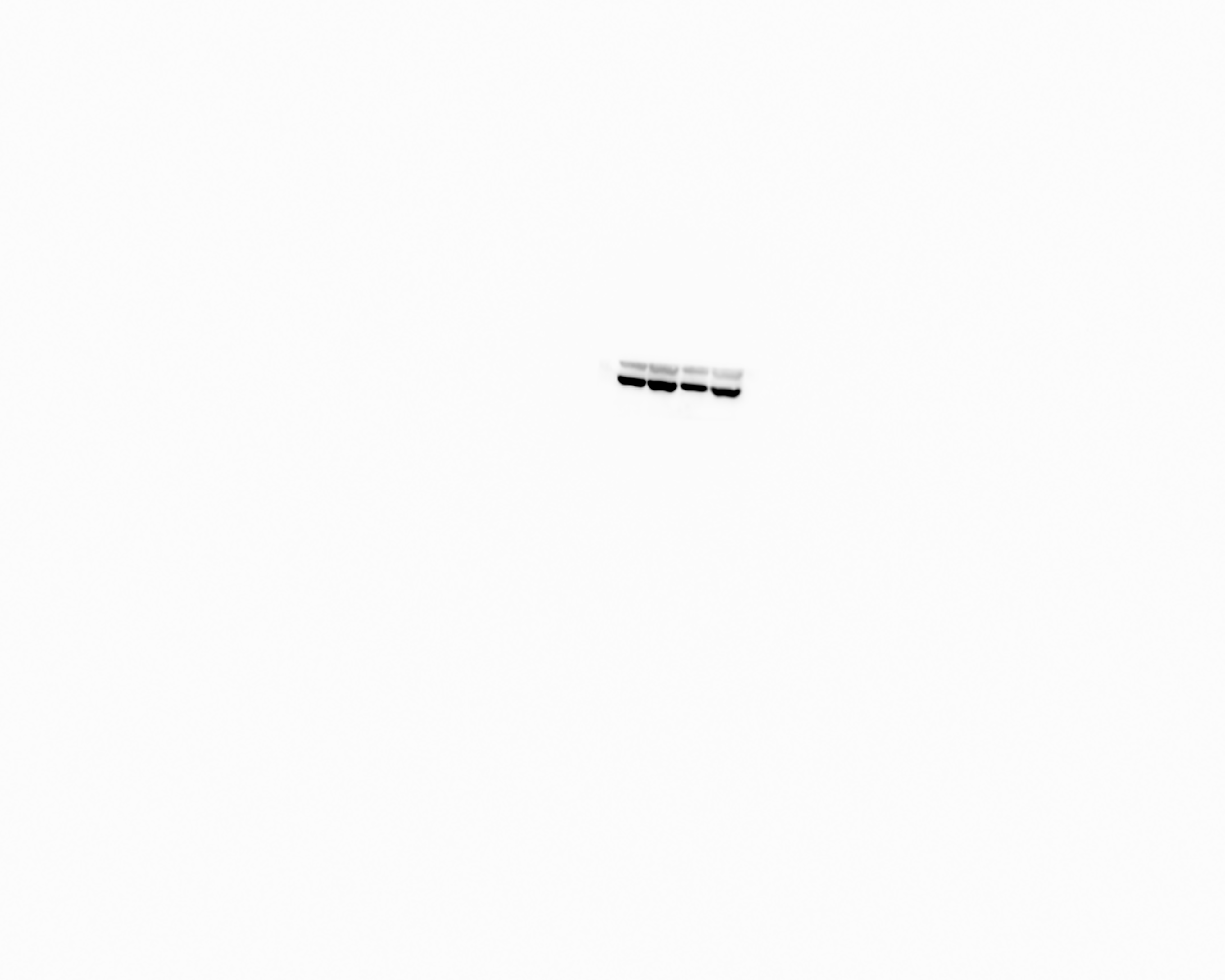

Supplement: Supplementary file 7 — Additional file 7. [file 12964_2024_1475_MOESM7_ESM.zip › Additional file 2/Figure 5D/KYSE-30/OCT4.tif]

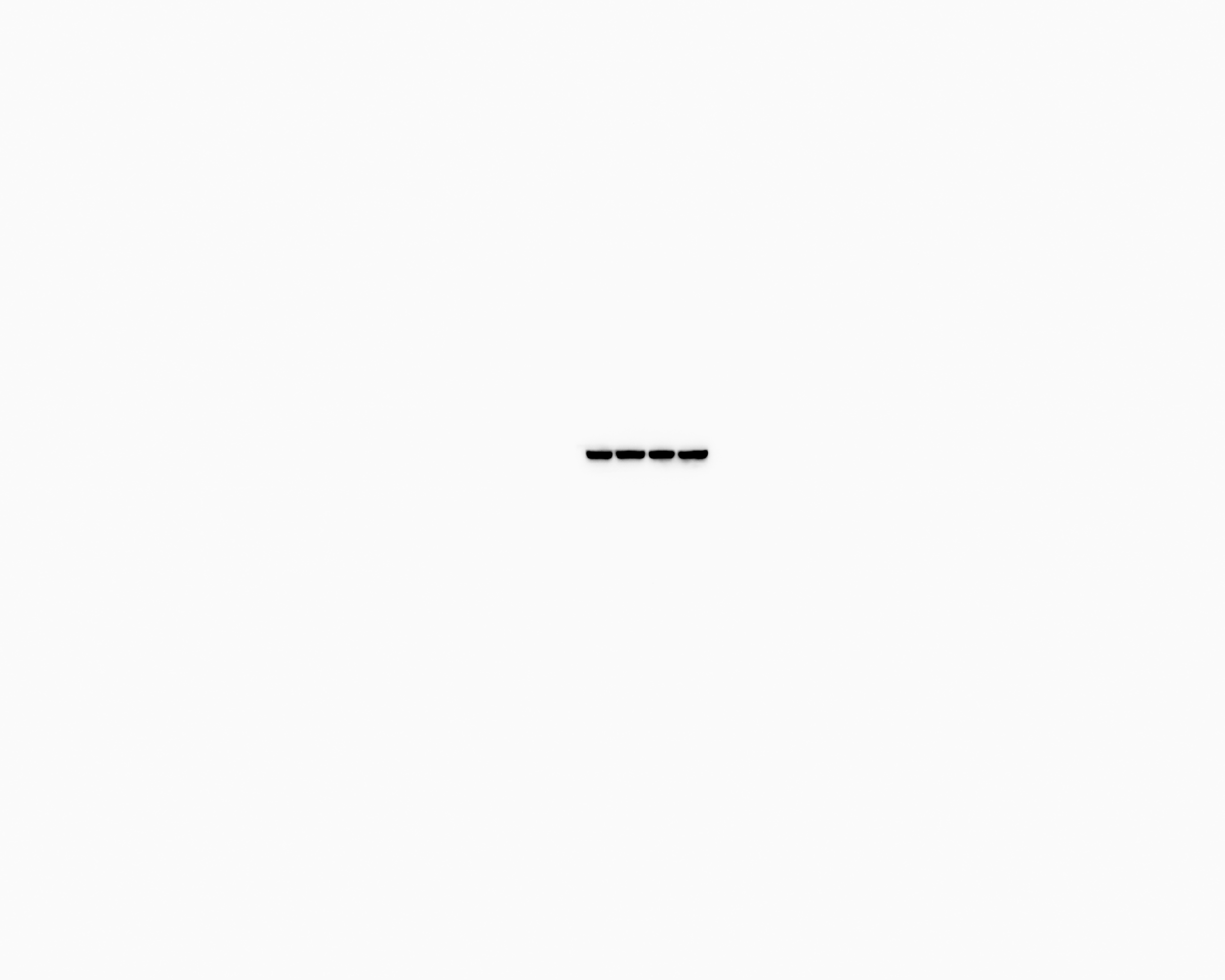

Supplement: Supplementary file 7 — Additional file 7. [file 12964_2024_1475_MOESM7_ESM.zip › Additional file 2/Figure 5D/KYSE-30/a┬-actin.tif]

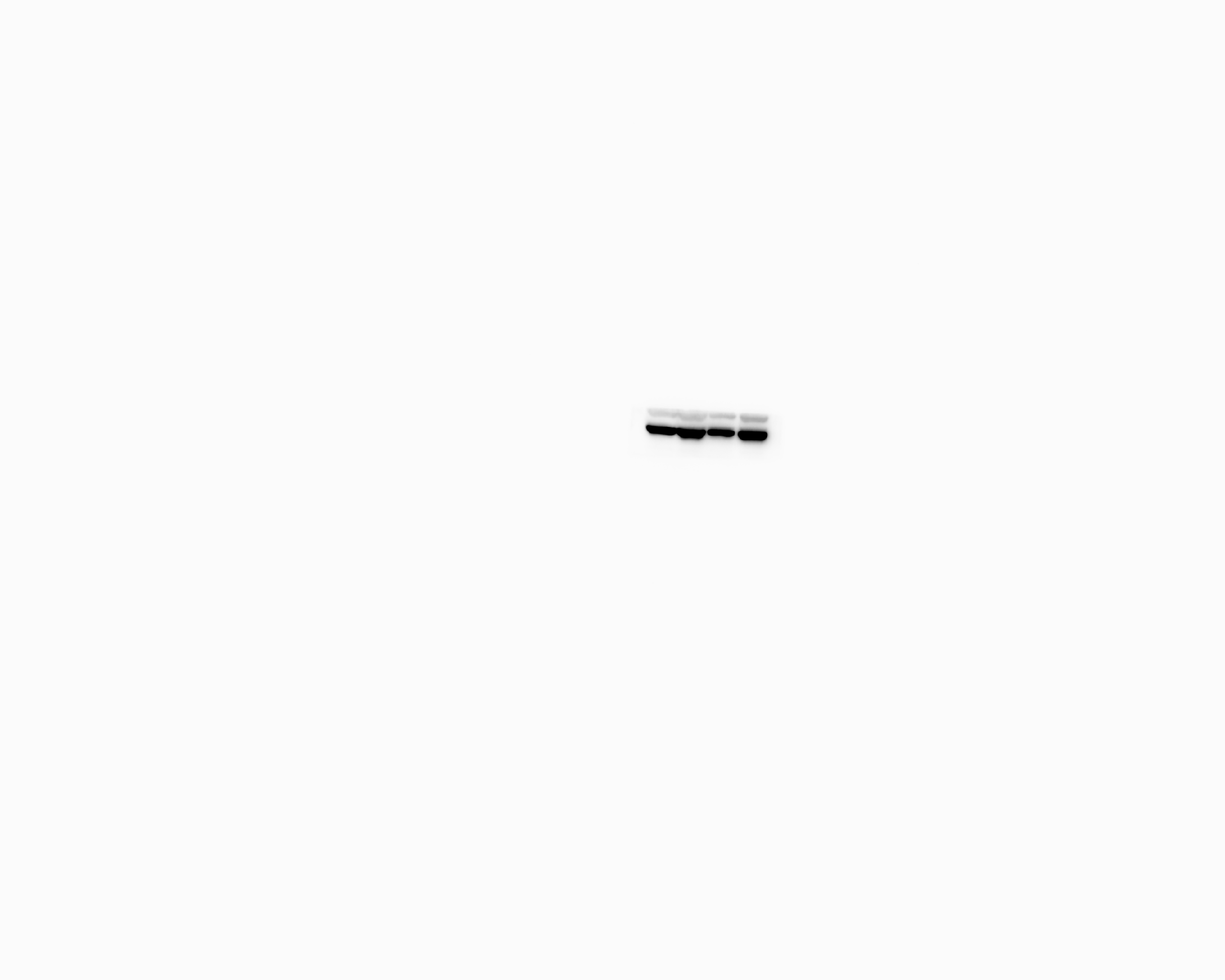

Supplement: Supplementary file 7 — Additional file 7. [file 12964_2024_1475_MOESM7_ESM.zip › Additional file 2/Figure 5E/KYSE-150/OCT4.tif]

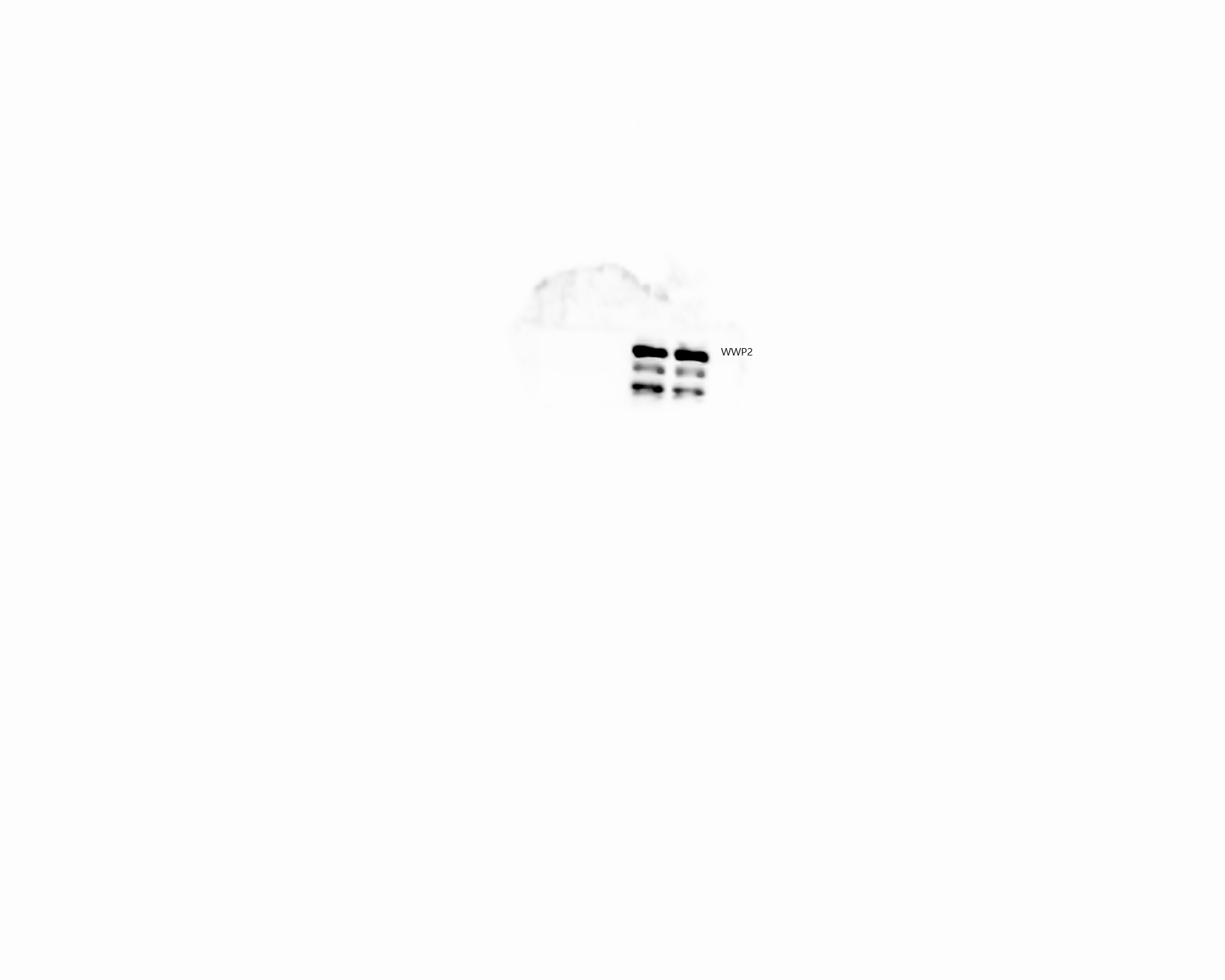

Supplement: Supplementary file 7 — Additional file 7. [file 12964_2024_1475_MOESM7_ESM.zip › Additional file 2/Figure 5E/KYSE-150/WWP2.tif]

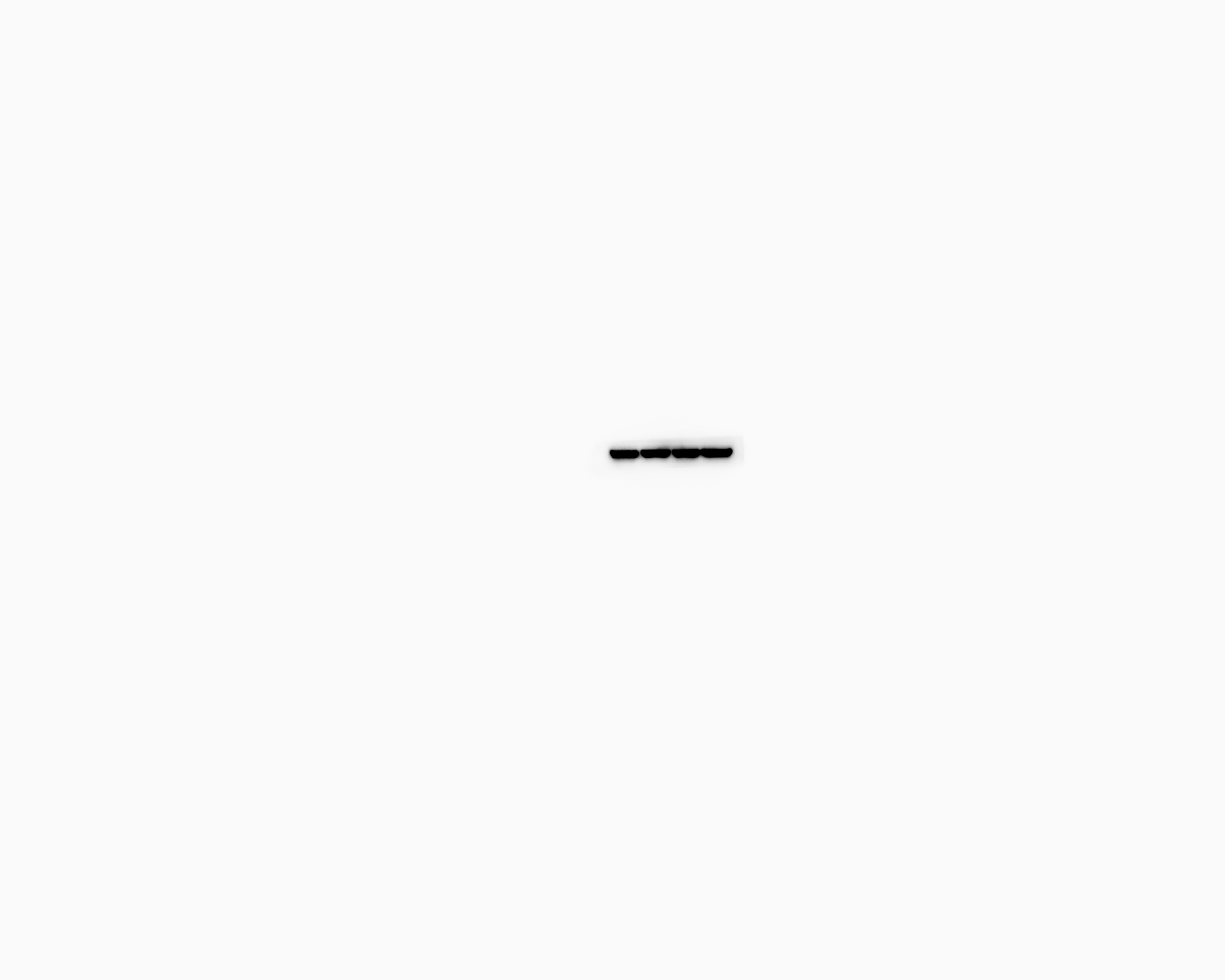

Supplement: Supplementary file 7 — Additional file 7. [file 12964_2024_1475_MOESM7_ESM.zip › Additional file 2/Figure 5E/KYSE-150/a┬-actin.tif]

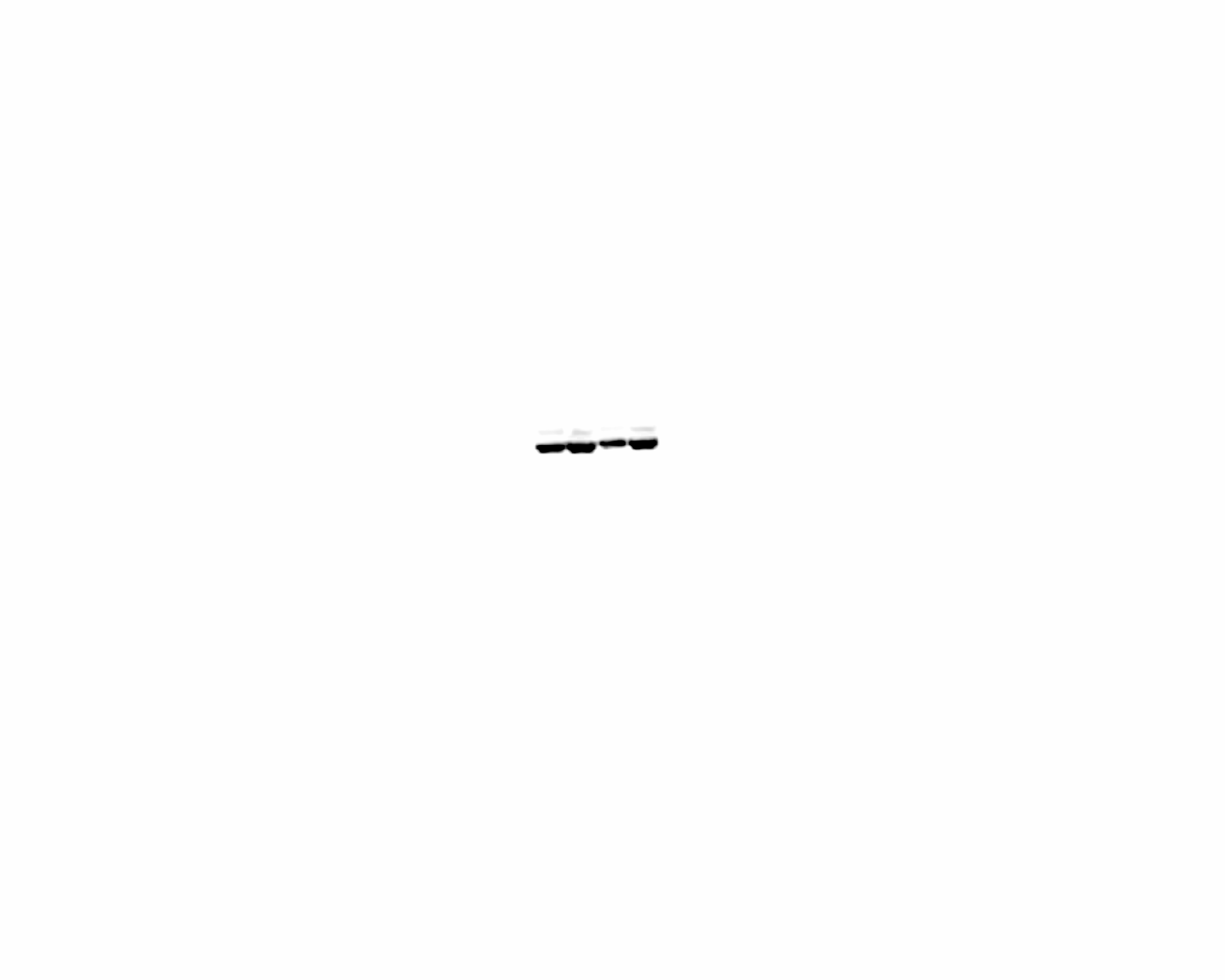

Supplement: Supplementary file 7 — Additional file 7. [file 12964_2024_1475_MOESM7_ESM.zip › Additional file 2/Figure 5E/KYSE-30/OCT4.tif]

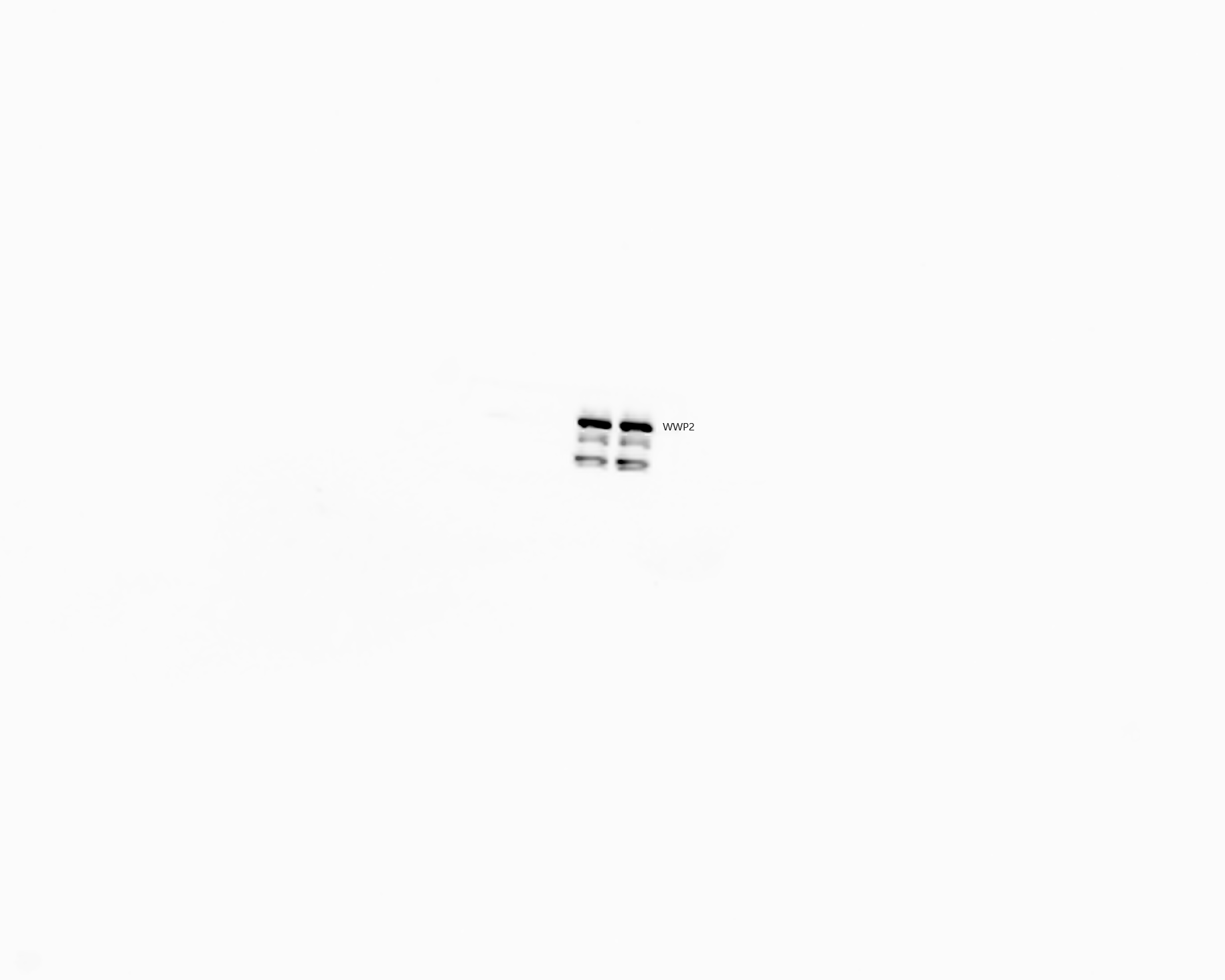

Supplement: Supplementary file 7 — Additional file 7. [file 12964_2024_1475_MOESM7_ESM.zip › Additional file 2/Figure 5E/KYSE-30/WWP2.tif]

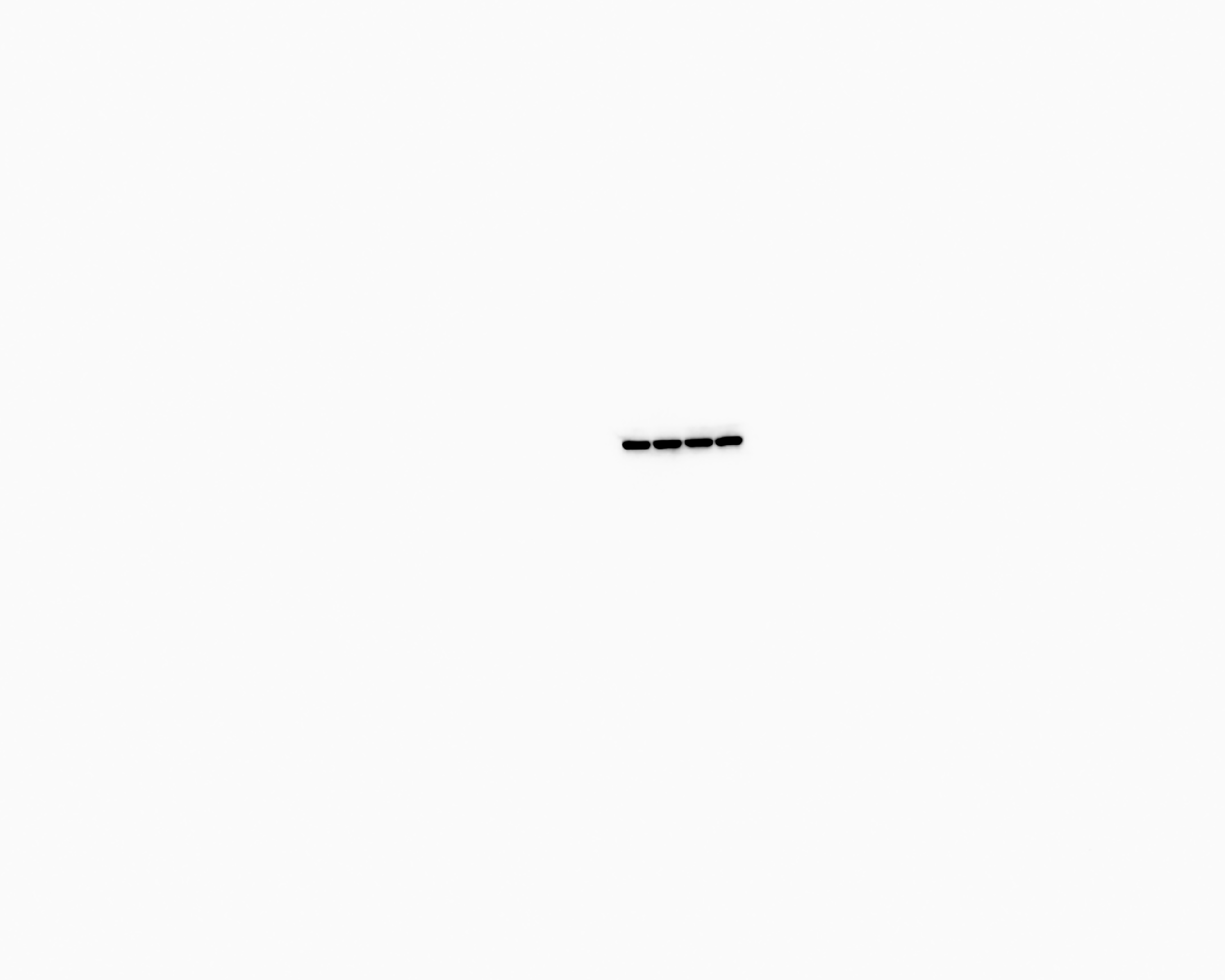

Supplement: Supplementary file 7 — Additional file 7. [file 12964_2024_1475_MOESM7_ESM.zip › Additional file 2/Figure 5E/KYSE-30/a┬-actin.tif]

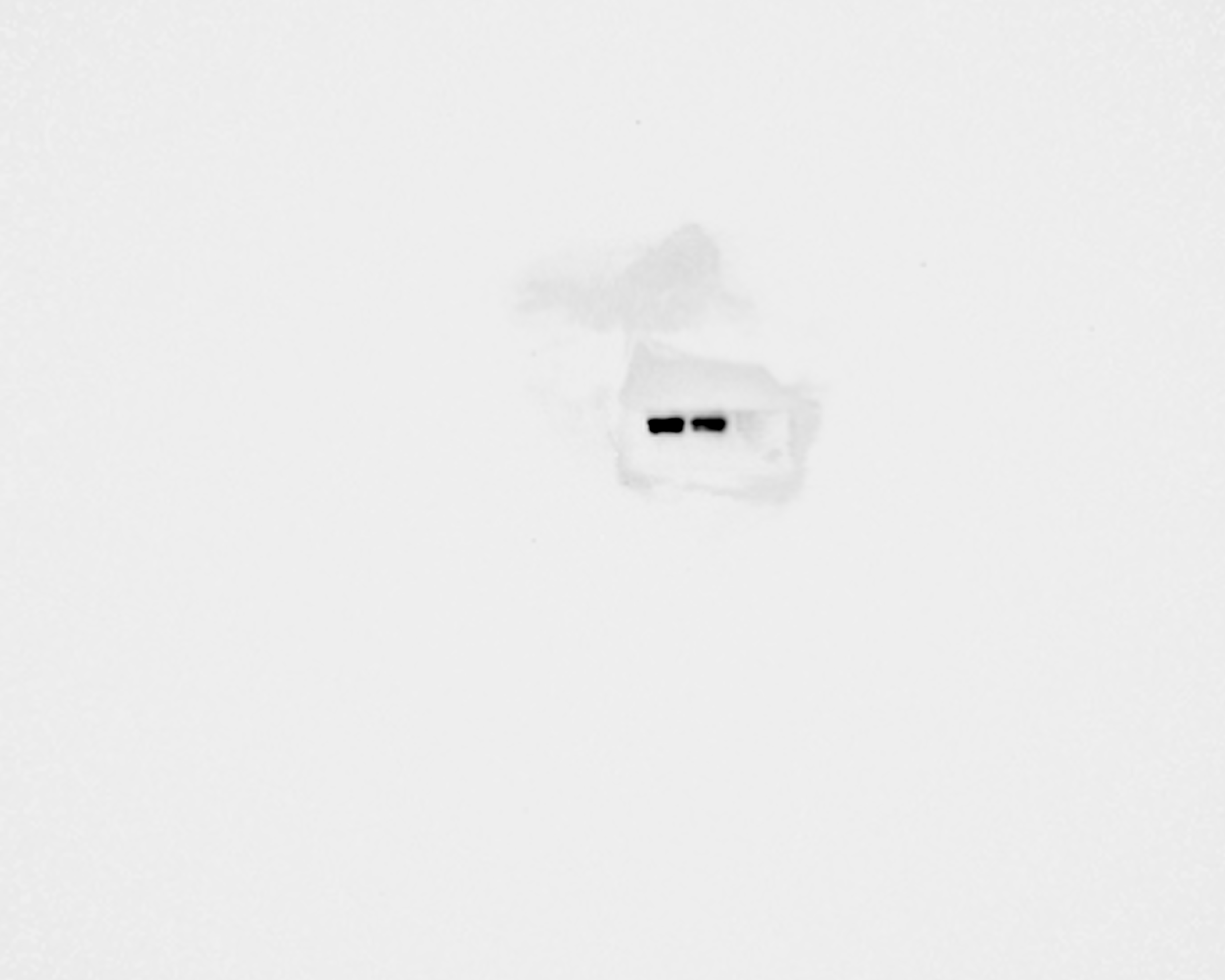

Supplement: Supplementary file 7 — Additional file 7. [file 12964_2024_1475_MOESM7_ESM.zip › Additional file 2/Figure 5F/Eca-109/oct4.tif]

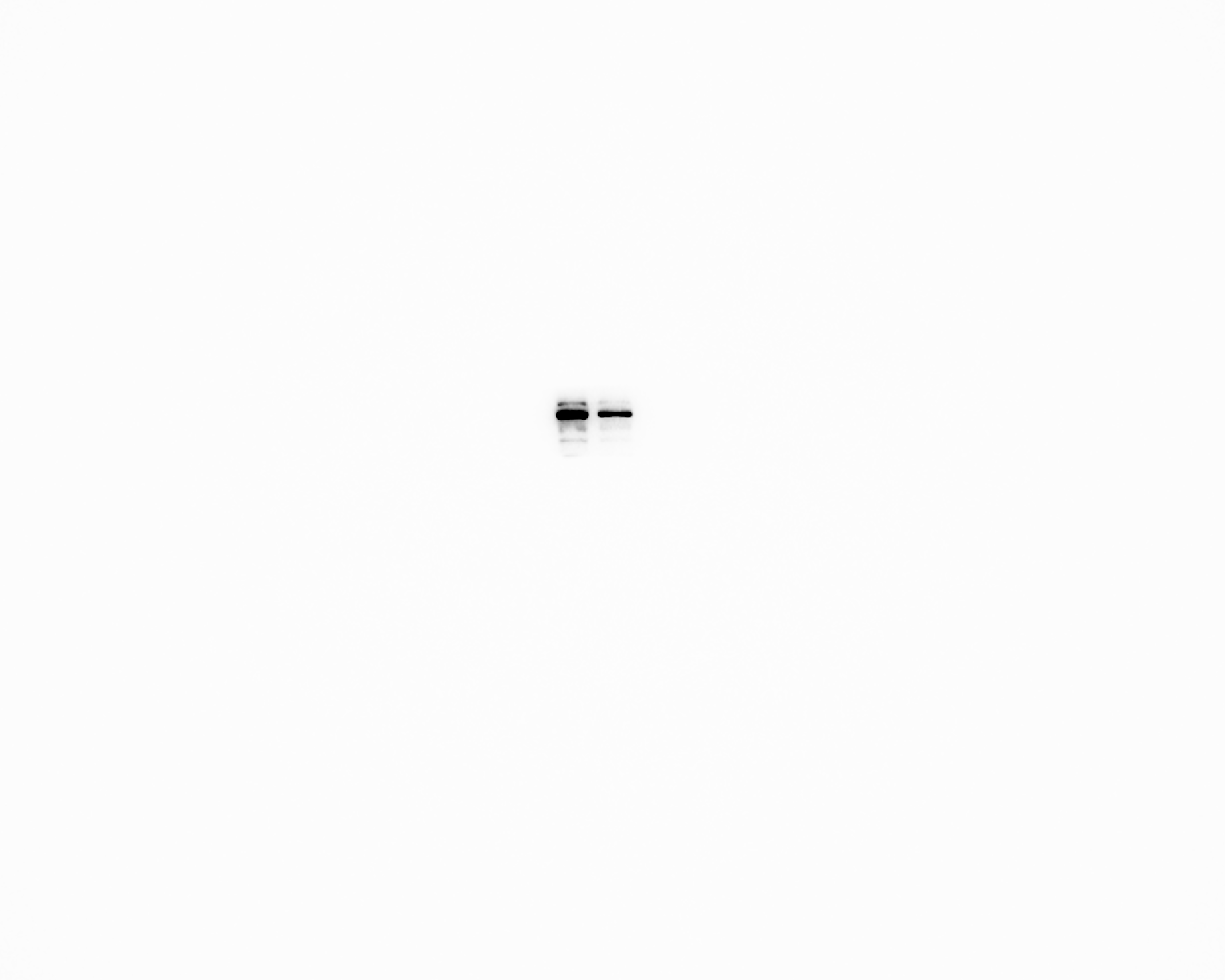

Supplement: Supplementary file 7 — Additional file 7. [file 12964_2024_1475_MOESM7_ESM.zip › Additional file 2/Figure 5F/Eca-109/wwp2.tif]

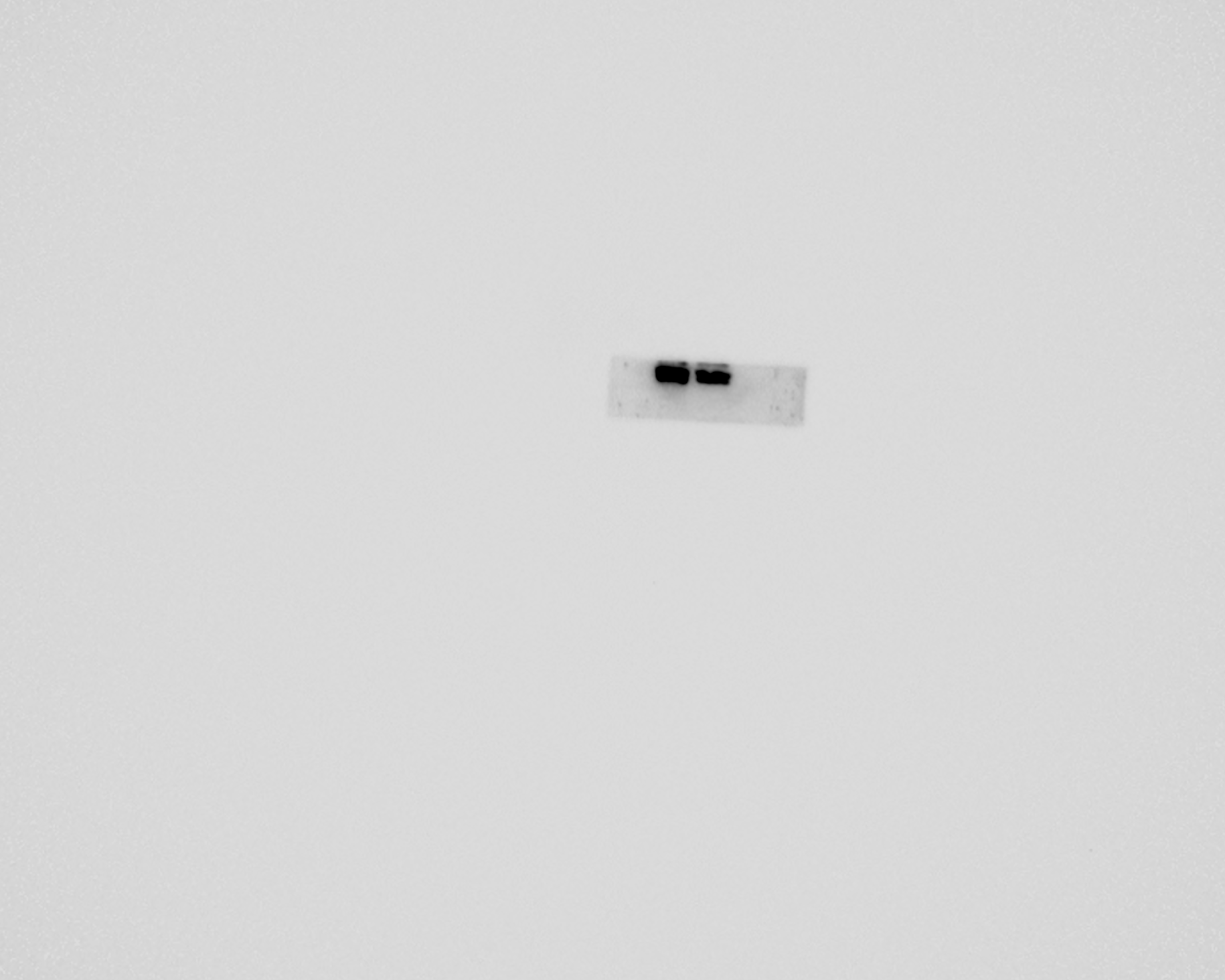

Supplement: Supplementary file 7 — Additional file 7. [file 12964_2024_1475_MOESM7_ESM.zip › Additional file 2/Figure 5F/KYSE-150/OCT4.tif]

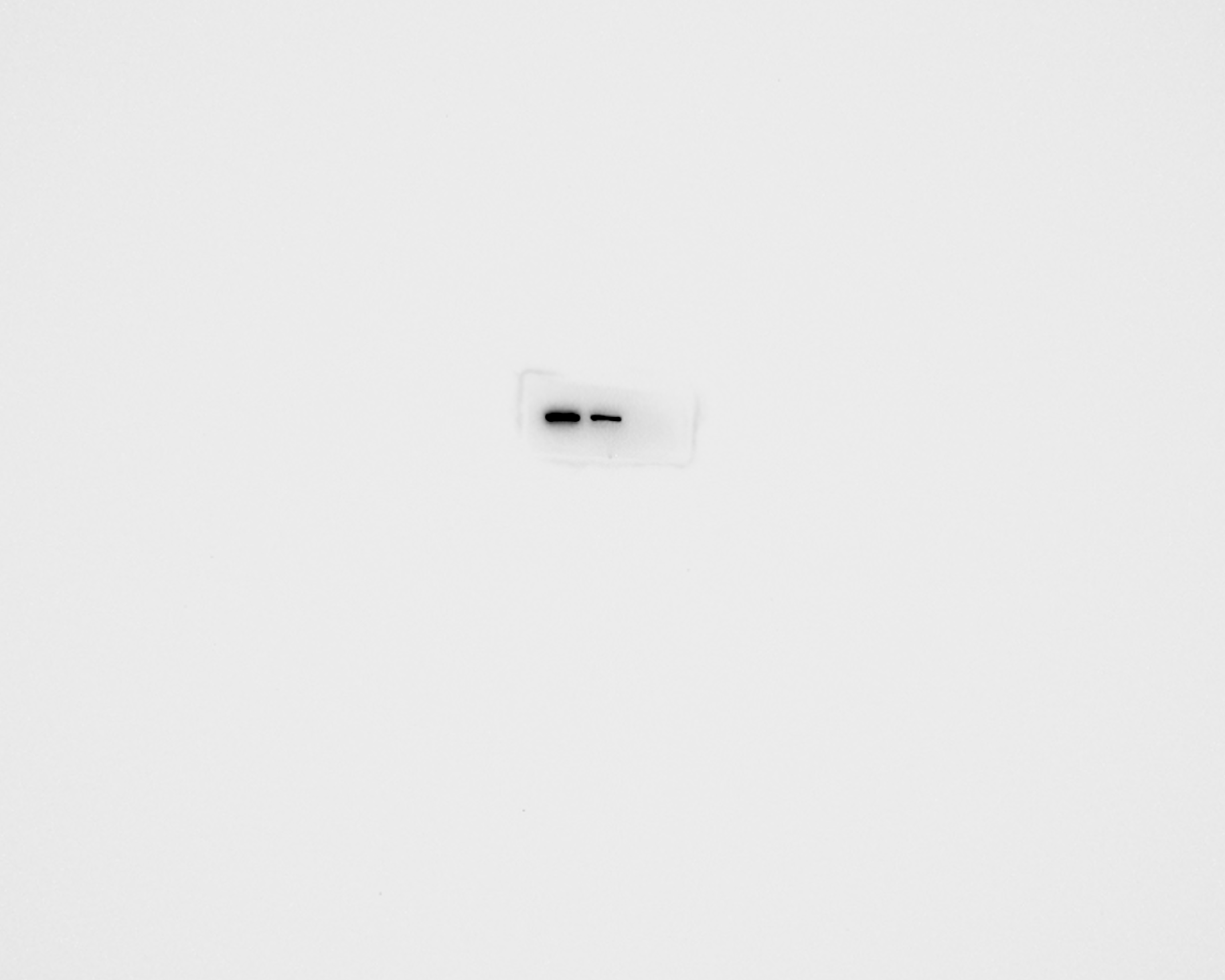

Supplement: Supplementary file 7 — Additional file 7. [file 12964_2024_1475_MOESM7_ESM.zip › Additional file 2/Figure 5F/KYSE-150/WWP2.tif]

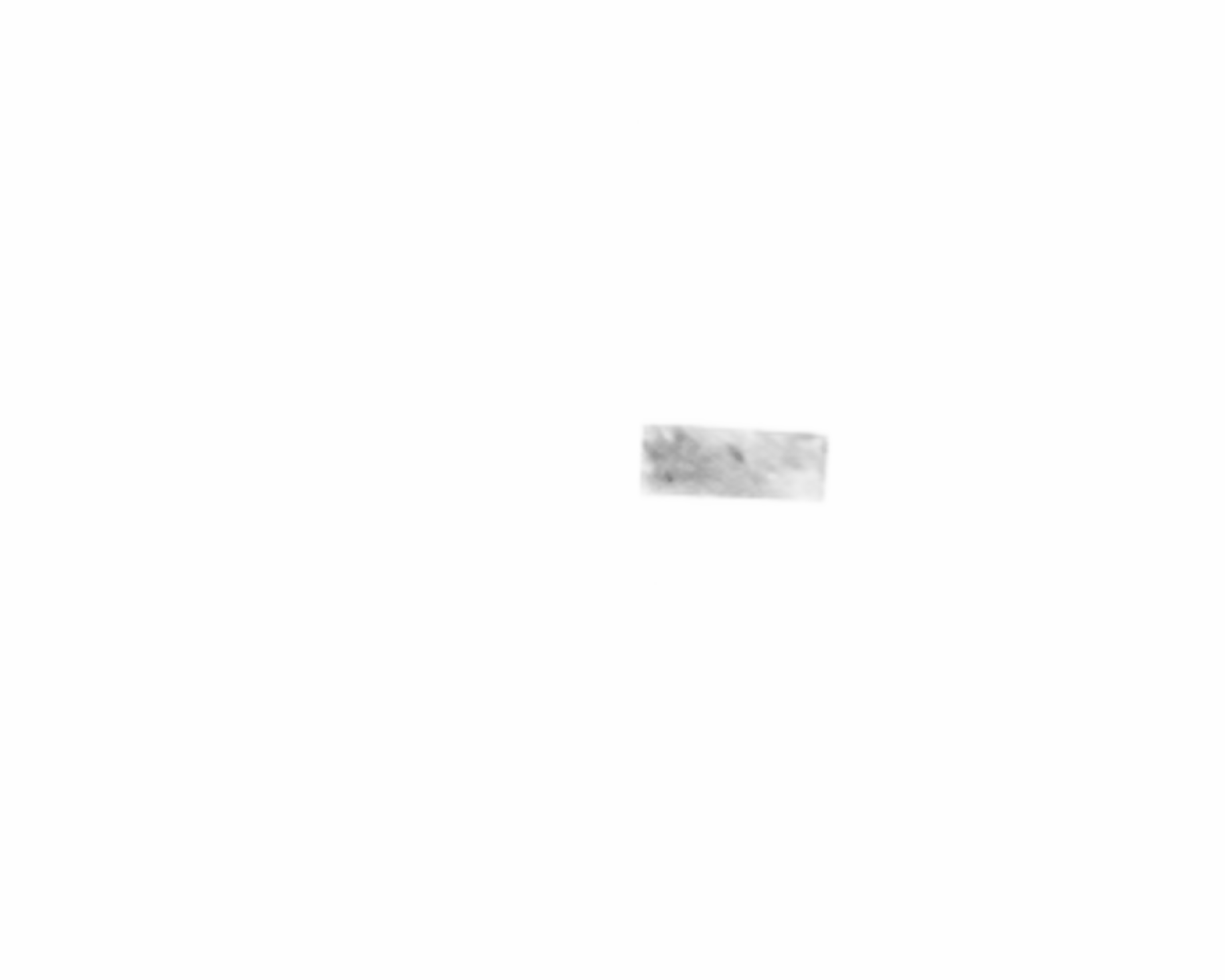

Supplement: Supplementary file 7 — Additional file 7. [file 12964_2024_1475_MOESM7_ESM.zip › Additional file 2/Figure 5G/KYSE-150/IgG oct4.tif]

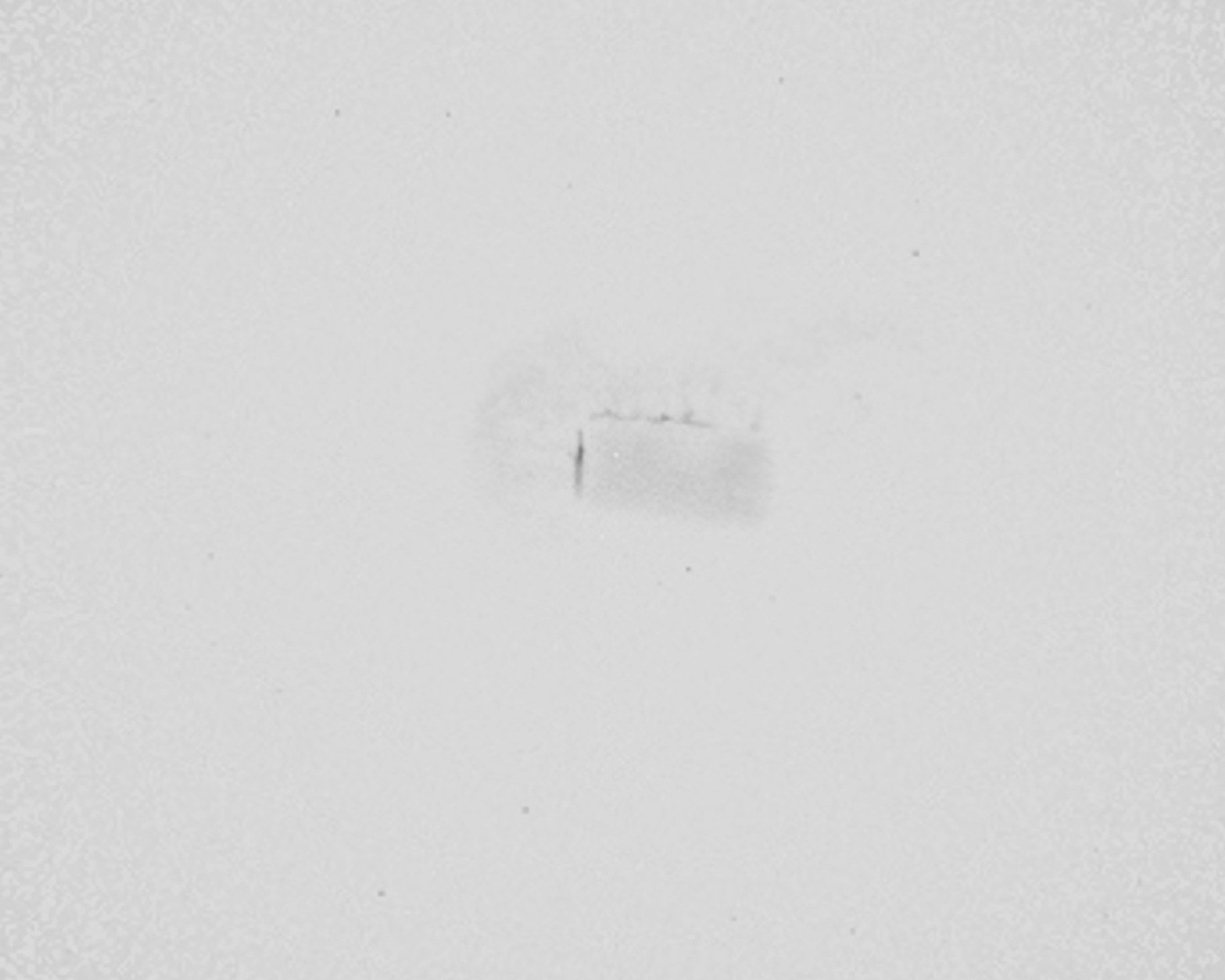

Supplement: Supplementary file 7 — Additional file 7. [file 12964_2024_1475_MOESM7_ESM.zip › Additional file 2/Figure 5G/KYSE-150/IgG ubiquitin.tif]

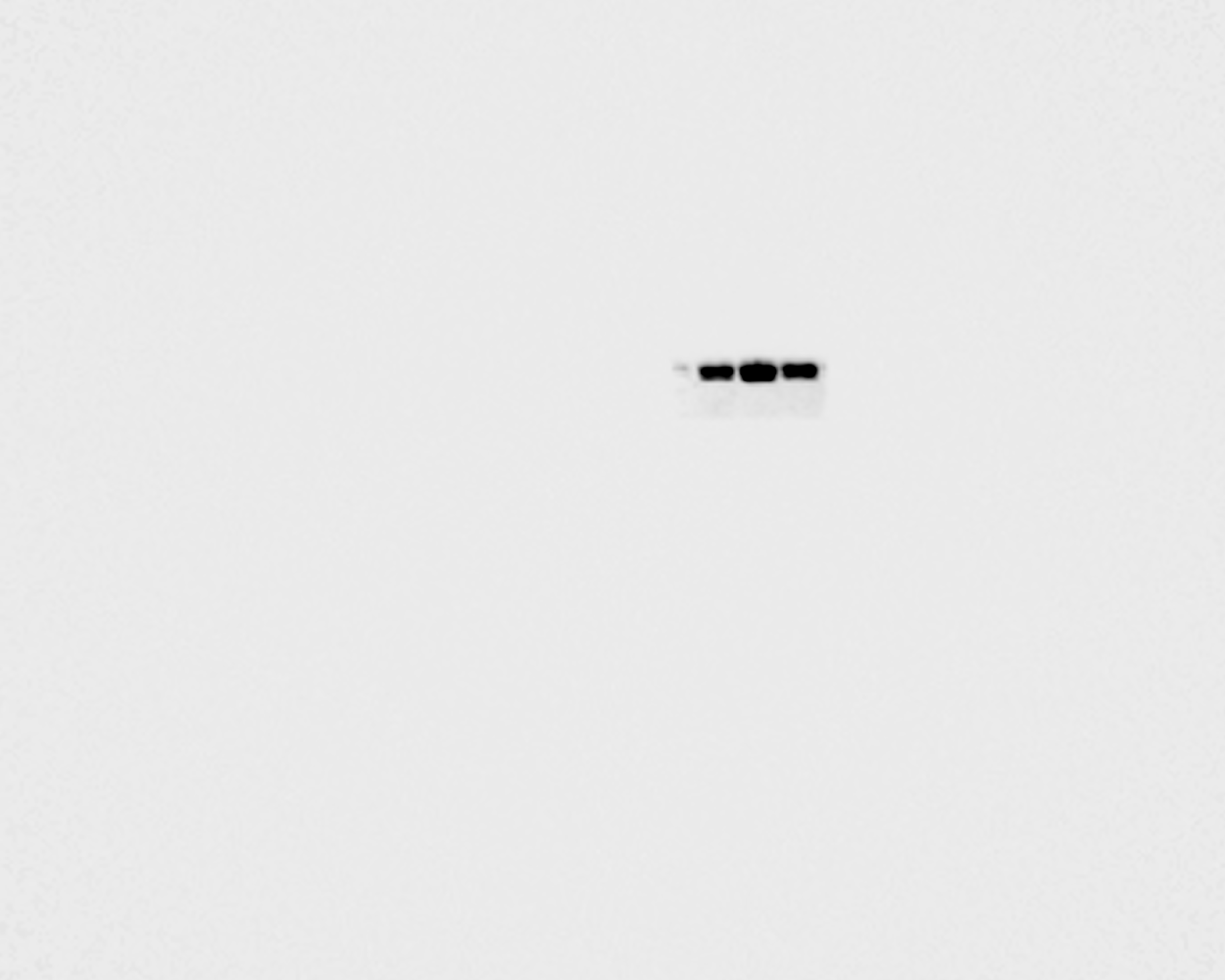

Supplement: Supplementary file 7 — Additional file 7. [file 12964_2024_1475_MOESM7_ESM.zip › Additional file 2/Figure 5G/KYSE-150/input oct4.tif]

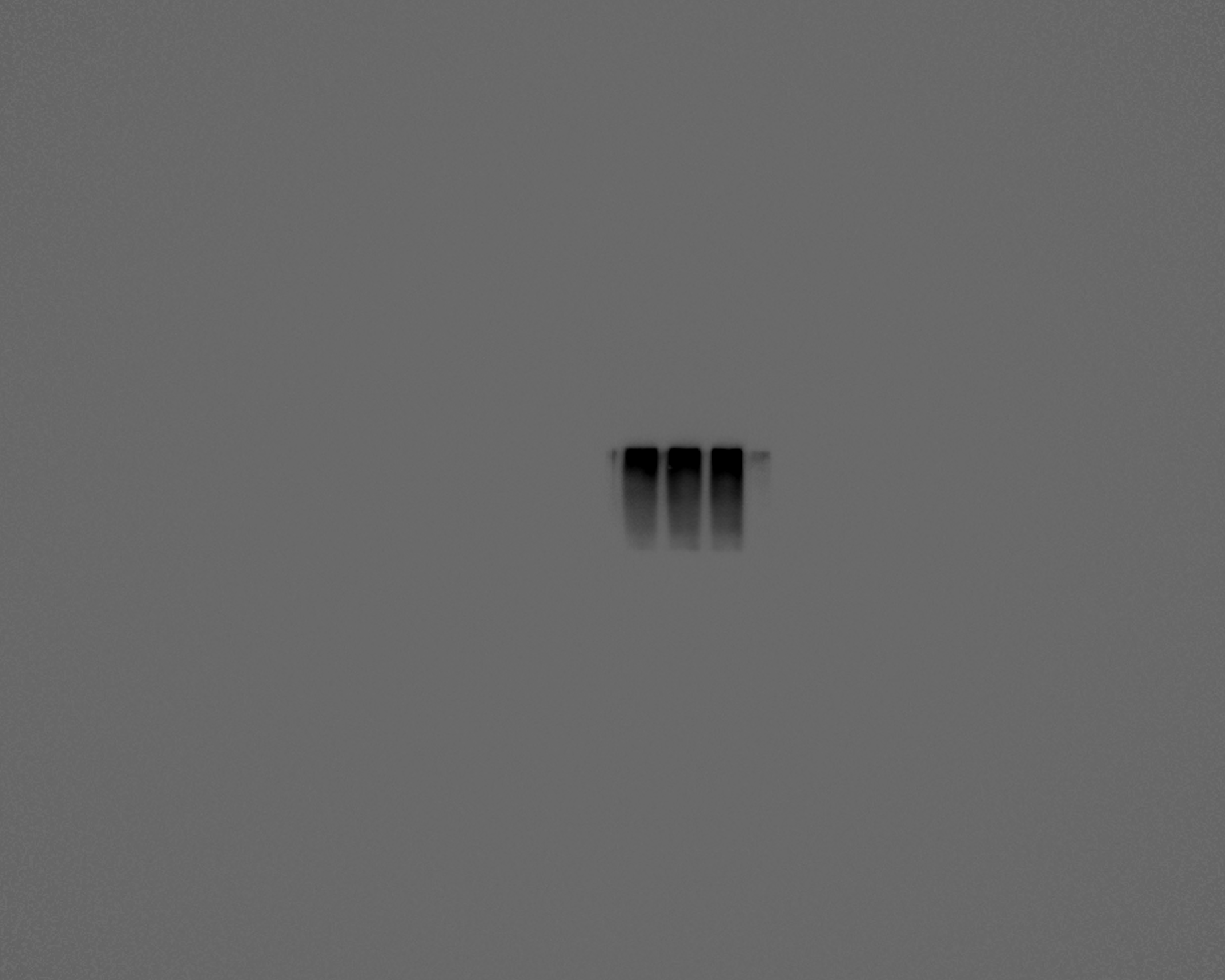

Supplement: Supplementary file 7 — Additional file 7. [file 12964_2024_1475_MOESM7_ESM.zip › Additional file 2/Figure 5G/KYSE-150/input ubiquitin.tif]

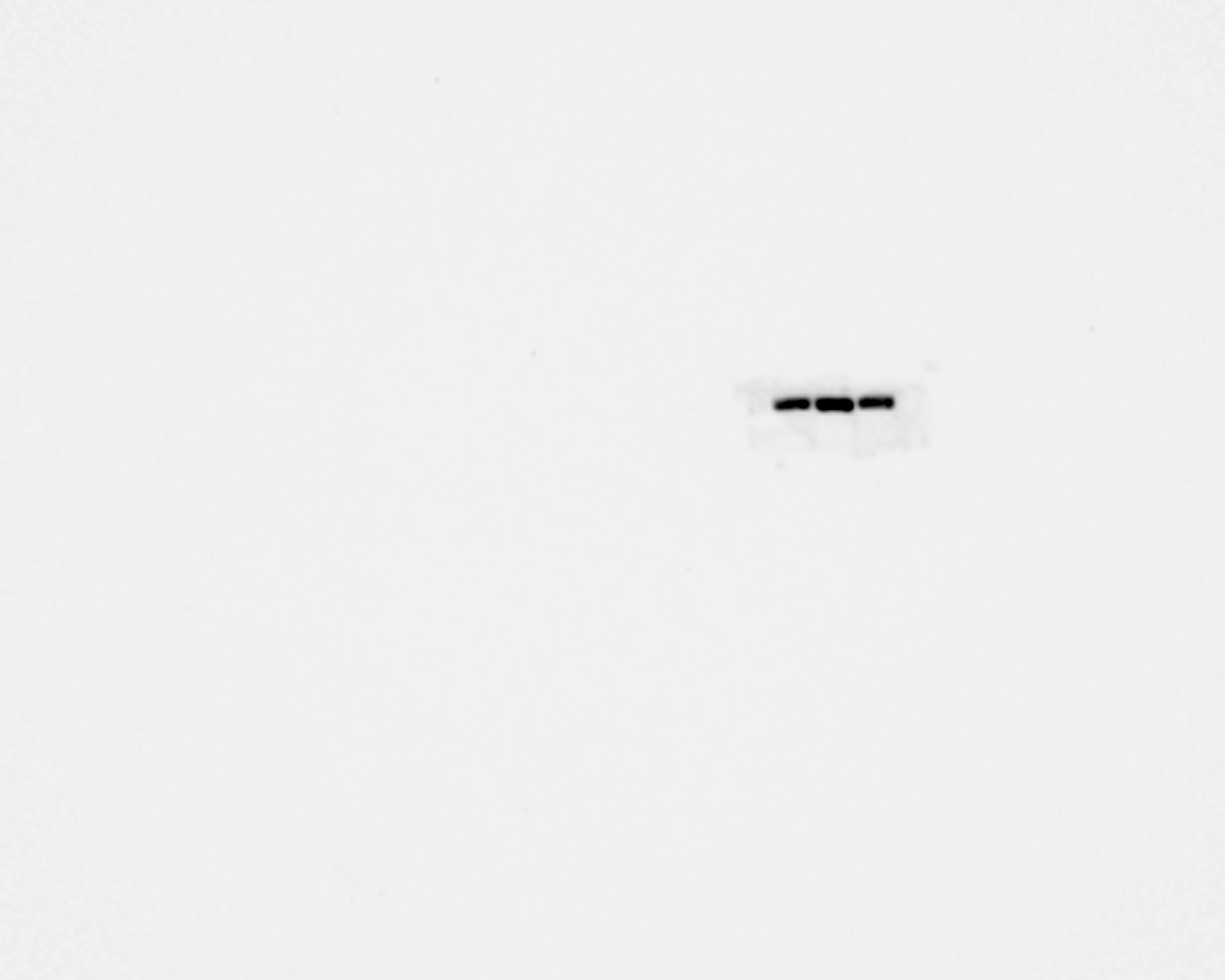

Supplement: Supplementary file 7 — Additional file 7. [file 12964_2024_1475_MOESM7_ESM.zip › Additional file 2/Figure 5G/KYSE-150/ip oct4.tif]

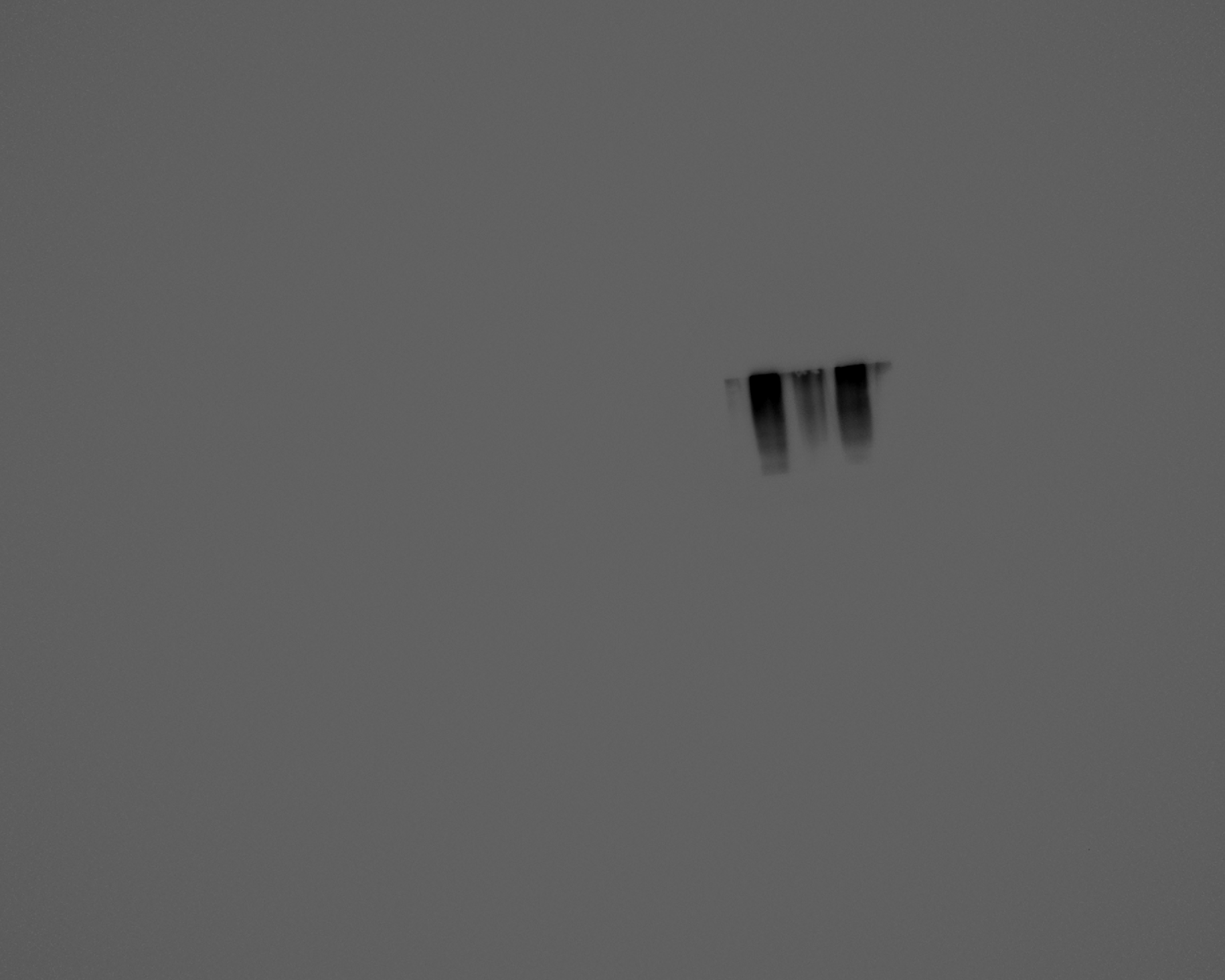

Supplement: Supplementary file 7 — Additional file 7. [file 12964_2024_1475_MOESM7_ESM.zip › Additional file 2/Figure 5G/KYSE-150/ip ubiquitin.tif]

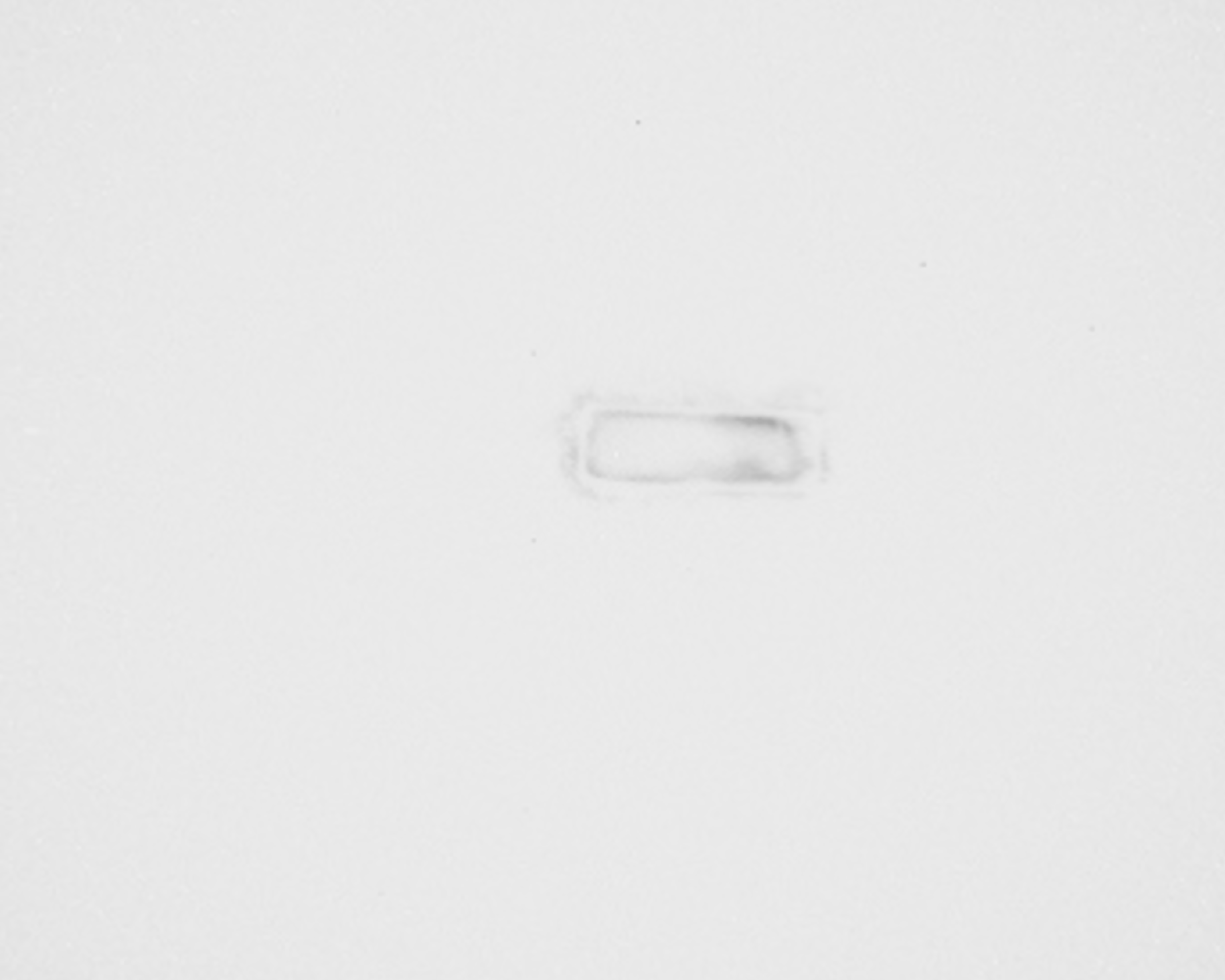

Supplement: Supplementary file 7 — Additional file 7. [file 12964_2024_1475_MOESM7_ESM.zip › Additional file 2/Figure 5G/KYSE-30/IgG oct4.tif]

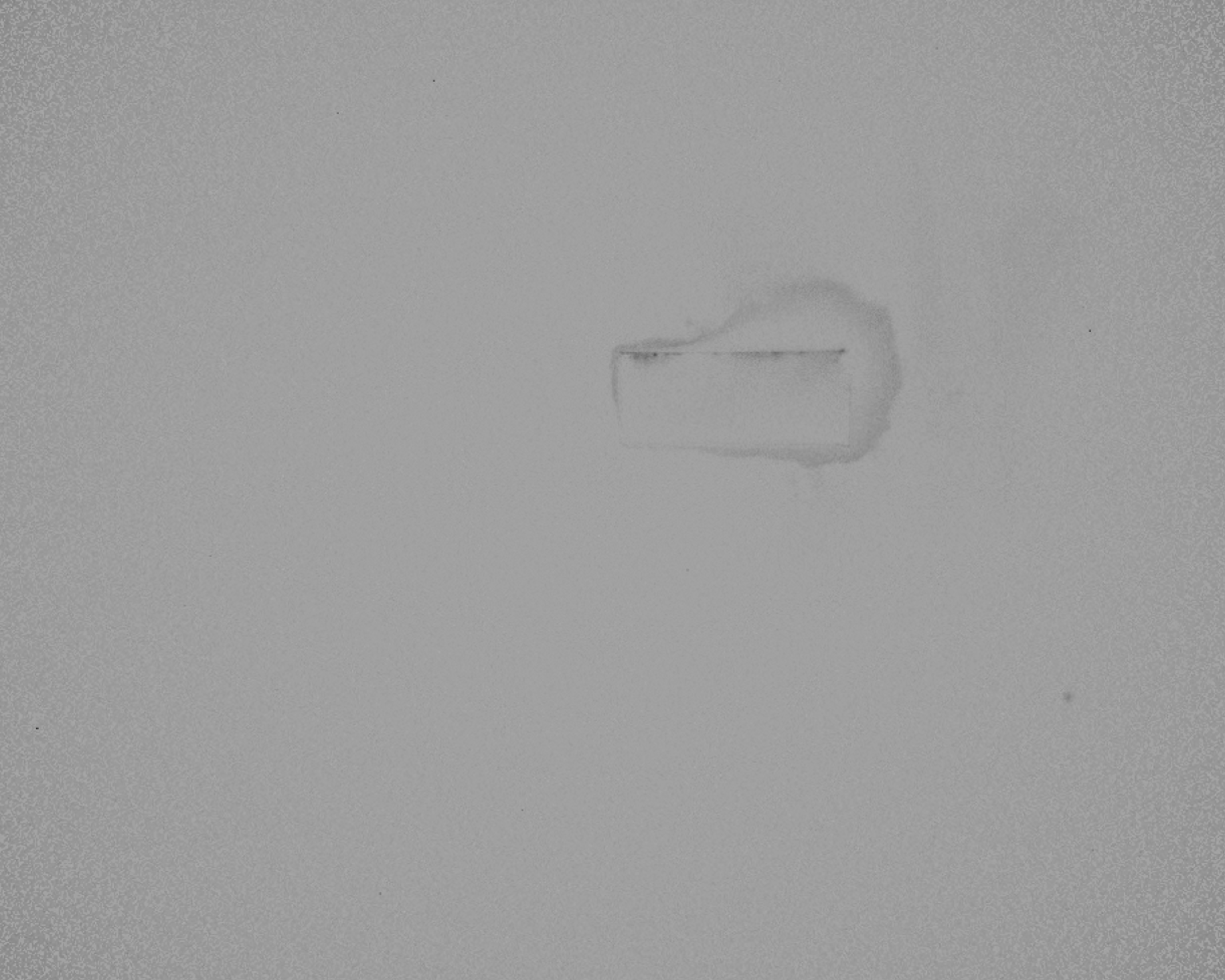

Supplement: Supplementary file 7 — Additional file 7. [file 12964_2024_1475_MOESM7_ESM.zip › Additional file 2/Figure 5G/KYSE-30/IgG ubiquitin.tif]

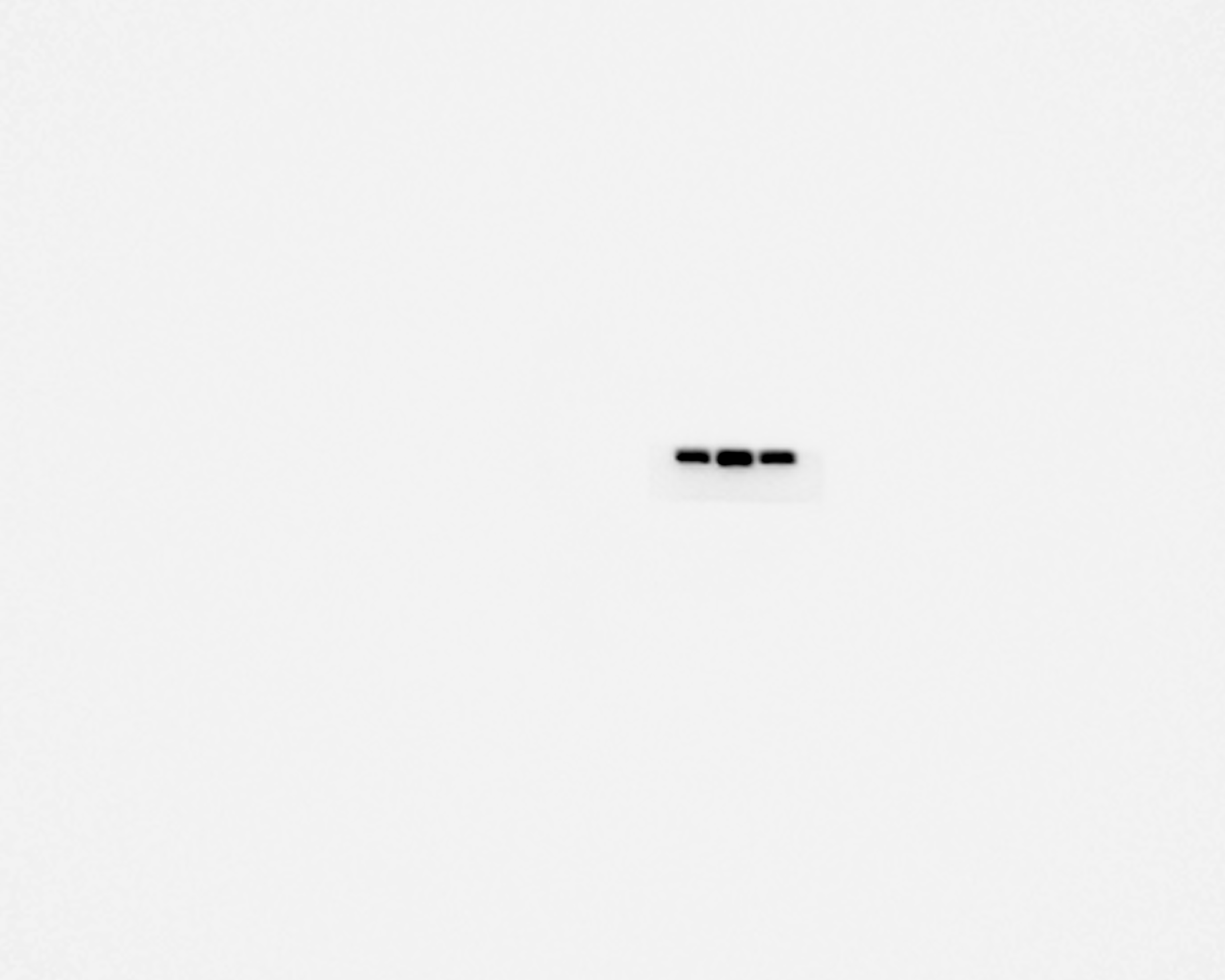

Supplement: Supplementary file 7 — Additional file 7. [file 12964_2024_1475_MOESM7_ESM.zip › Additional file 2/Figure 5G/KYSE-30/input oct4.tif]

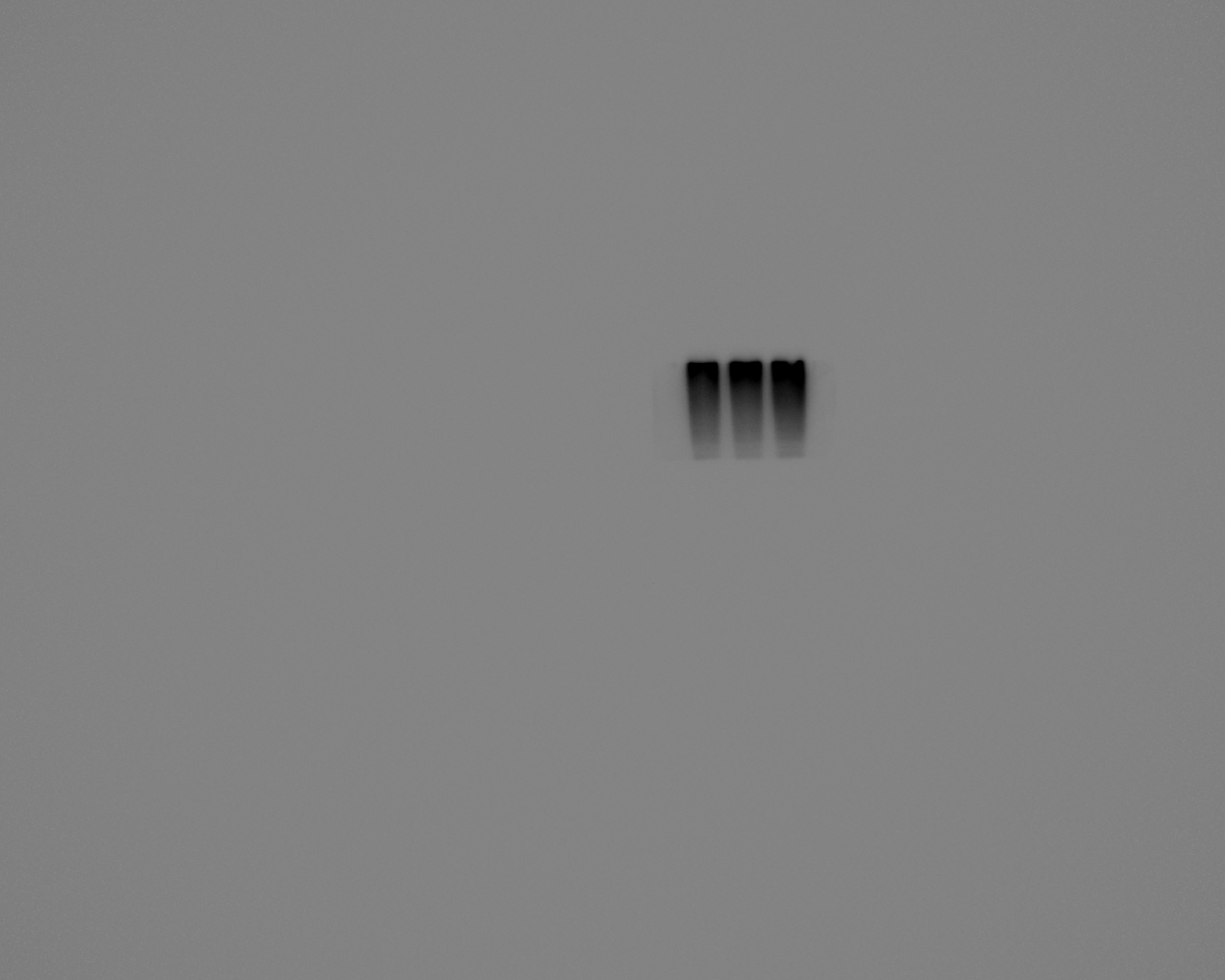

Supplement: Supplementary file 7 — Additional file 7. [file 12964_2024_1475_MOESM7_ESM.zip › Additional file 2/Figure 5G/KYSE-30/input ubiquitin.tif]

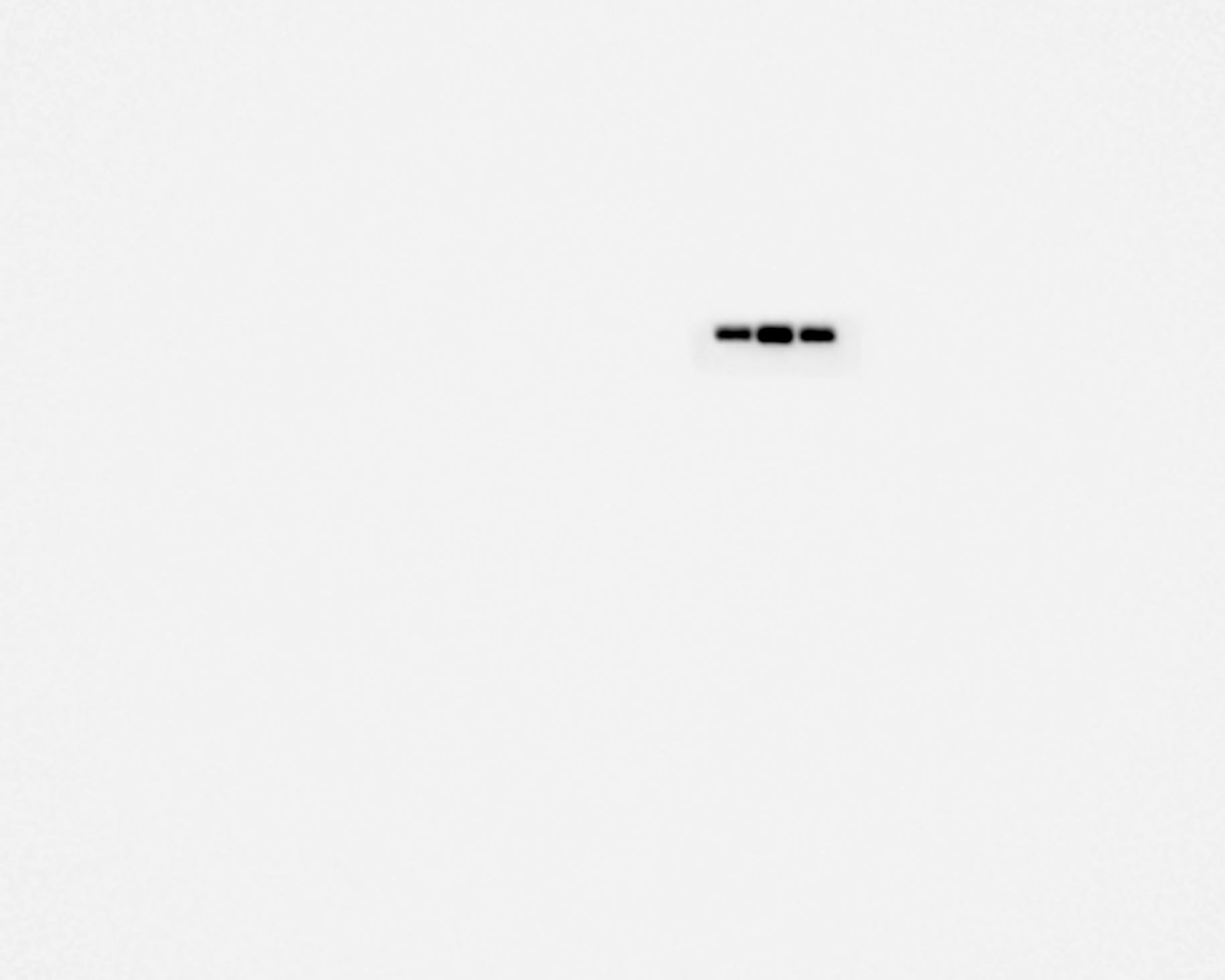

Supplement: Supplementary file 7 — Additional file 7. [file 12964_2024_1475_MOESM7_ESM.zip › Additional file 2/Figure 5G/KYSE-30/ip oct4.tif]

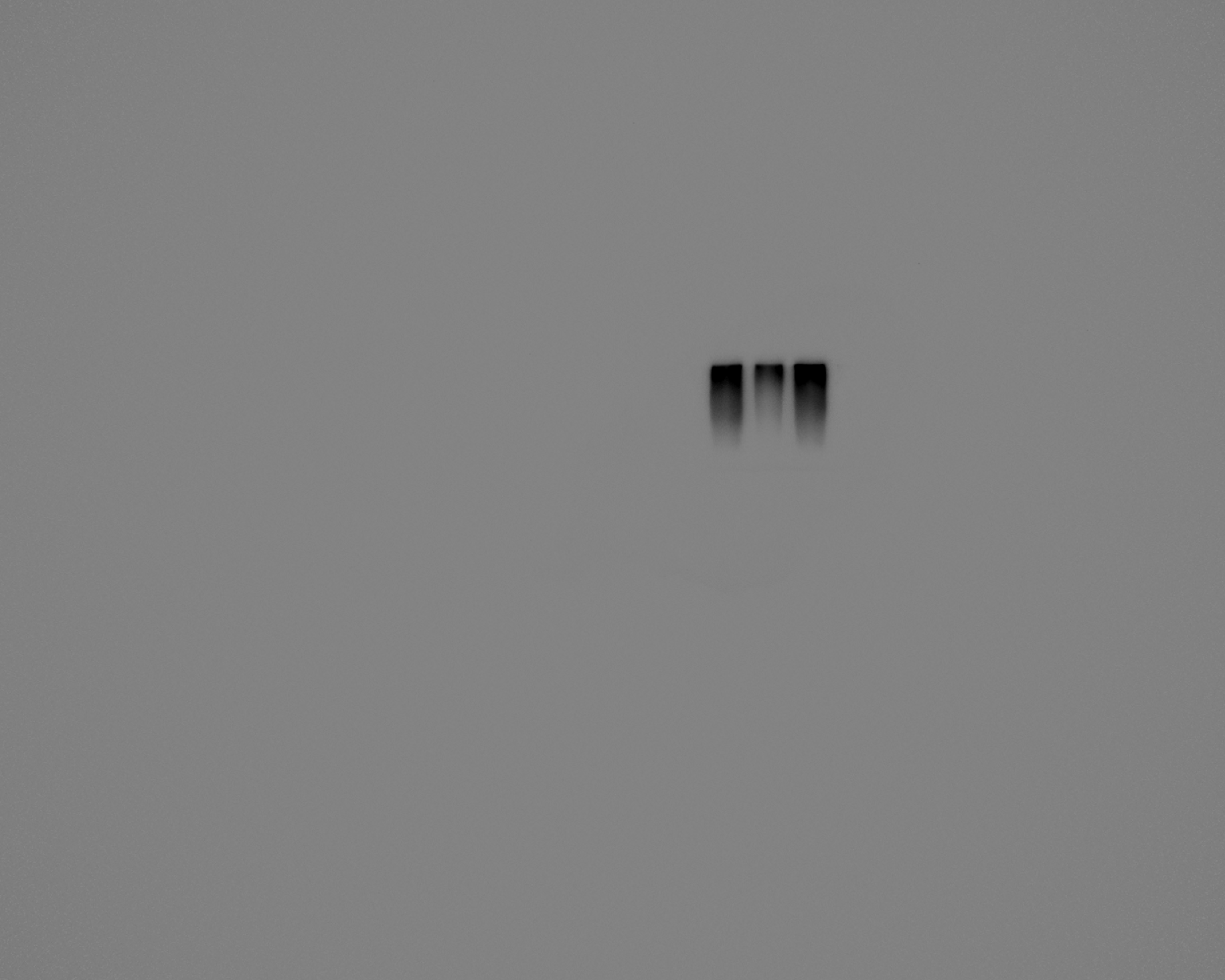

Supplement: Supplementary file 7 — Additional file 7. [file 12964_2024_1475_MOESM7_ESM.zip › Additional file 2/Figure 5G/KYSE-30/ip ubiquitin.tif]

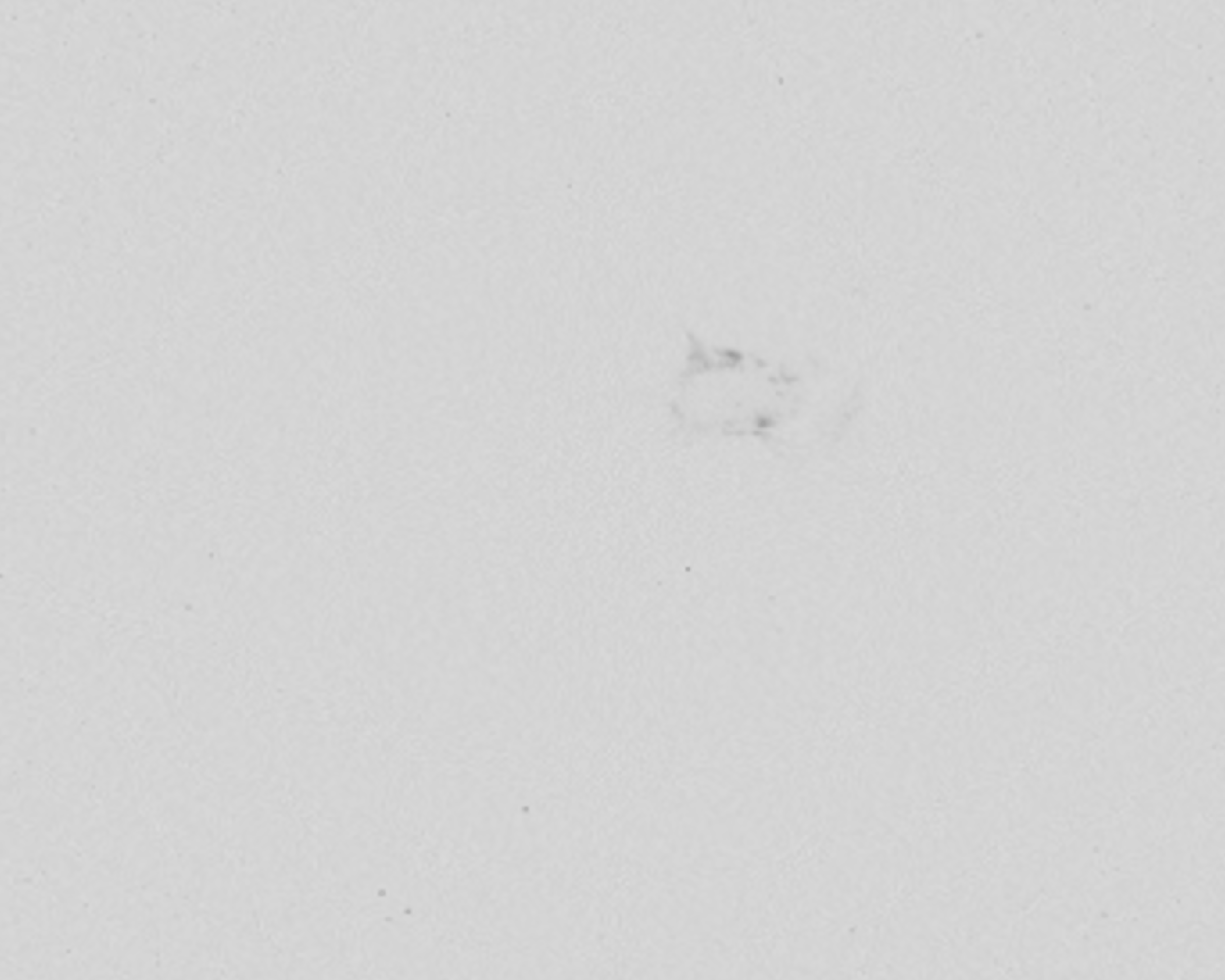

Supplement: Supplementary file 7 — Additional file 7. [file 12964_2024_1475_MOESM7_ESM.zip › Additional file 2/Figure 5H/Eca-109/IgG oct4.tif]

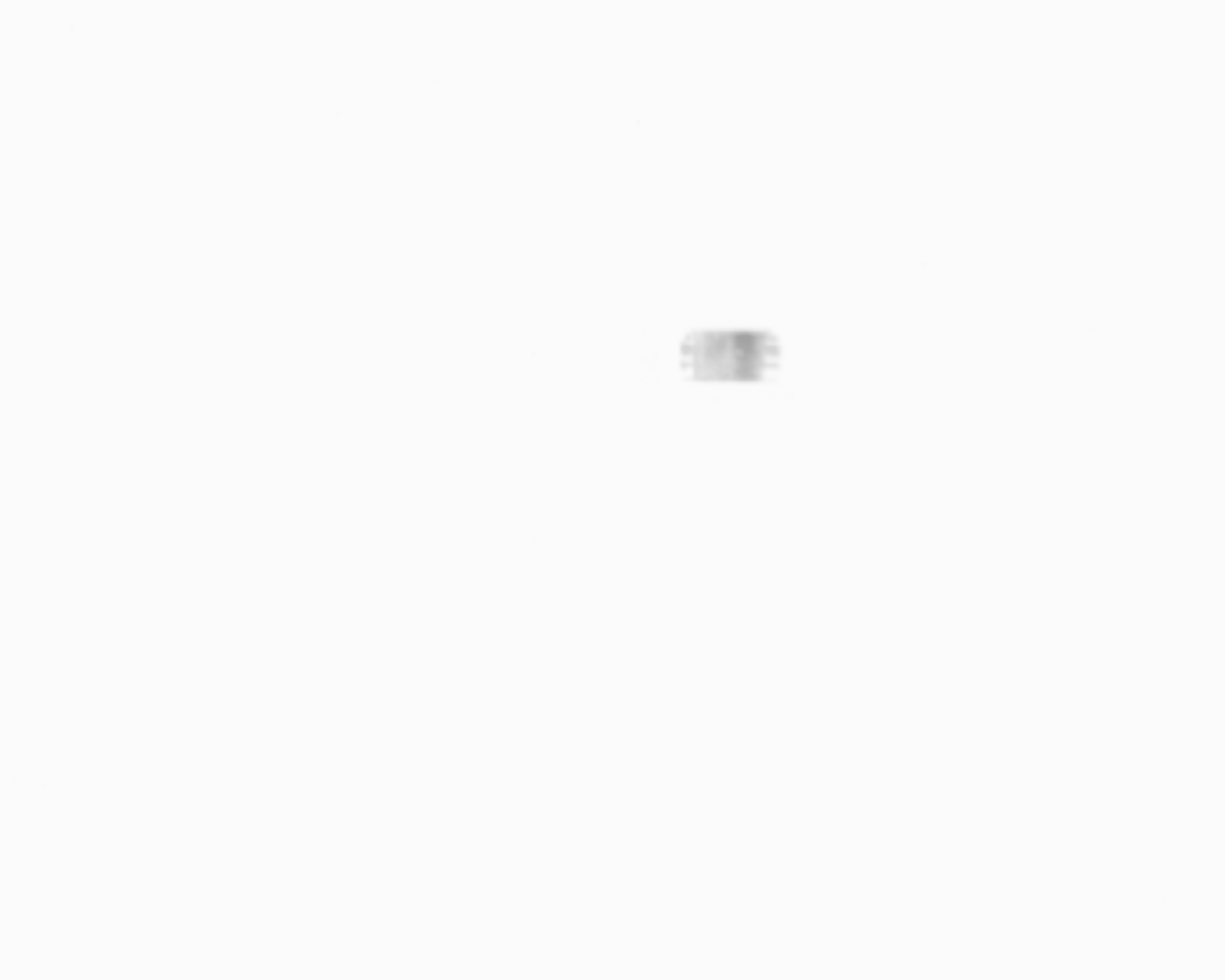

Supplement: Supplementary file 7 — Additional file 7. [file 12964_2024_1475_MOESM7_ESM.zip › Additional file 2/Figure 5H/Eca-109/IgG wwp2.tif]

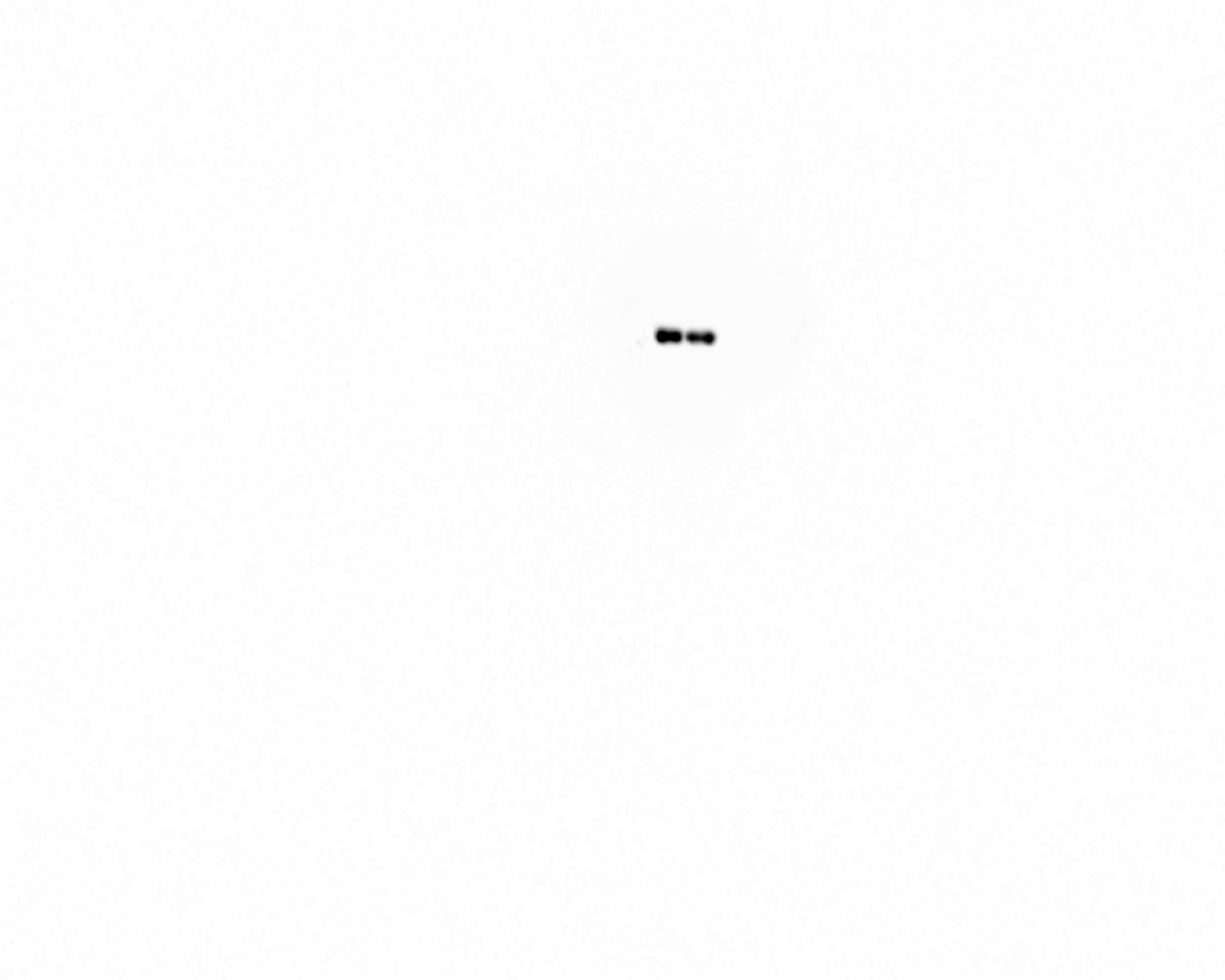

Supplement: Supplementary file 7 — Additional file 7. [file 12964_2024_1475_MOESM7_ESM.zip › Additional file 2/Figure 5H/Eca-109/input oct4.tif]

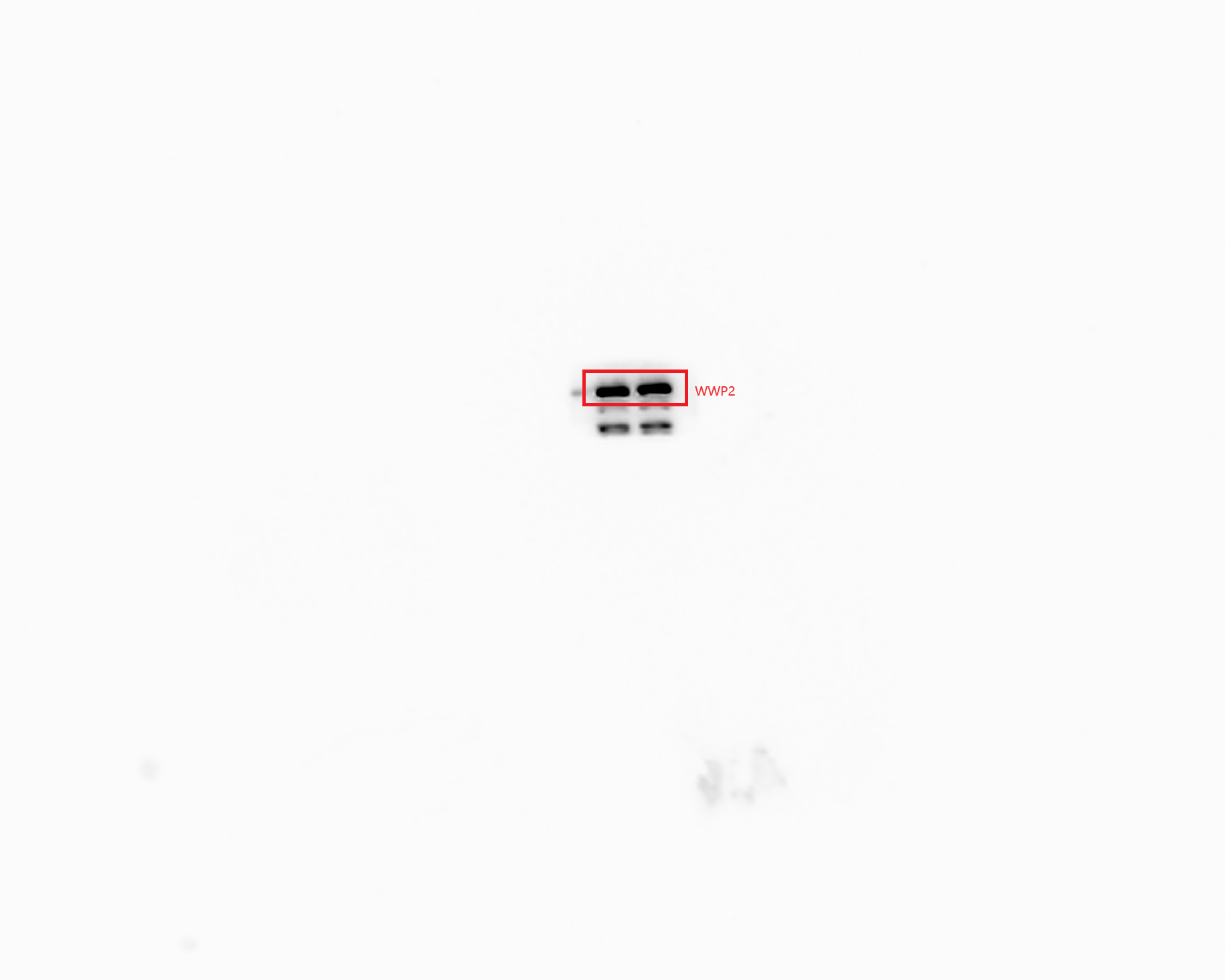

Supplement: Supplementary file 7 — Additional file 7. [file 12964_2024_1475_MOESM7_ESM.zip › Additional file 2/Figure 5H/Eca-109/input wwp2.tif]

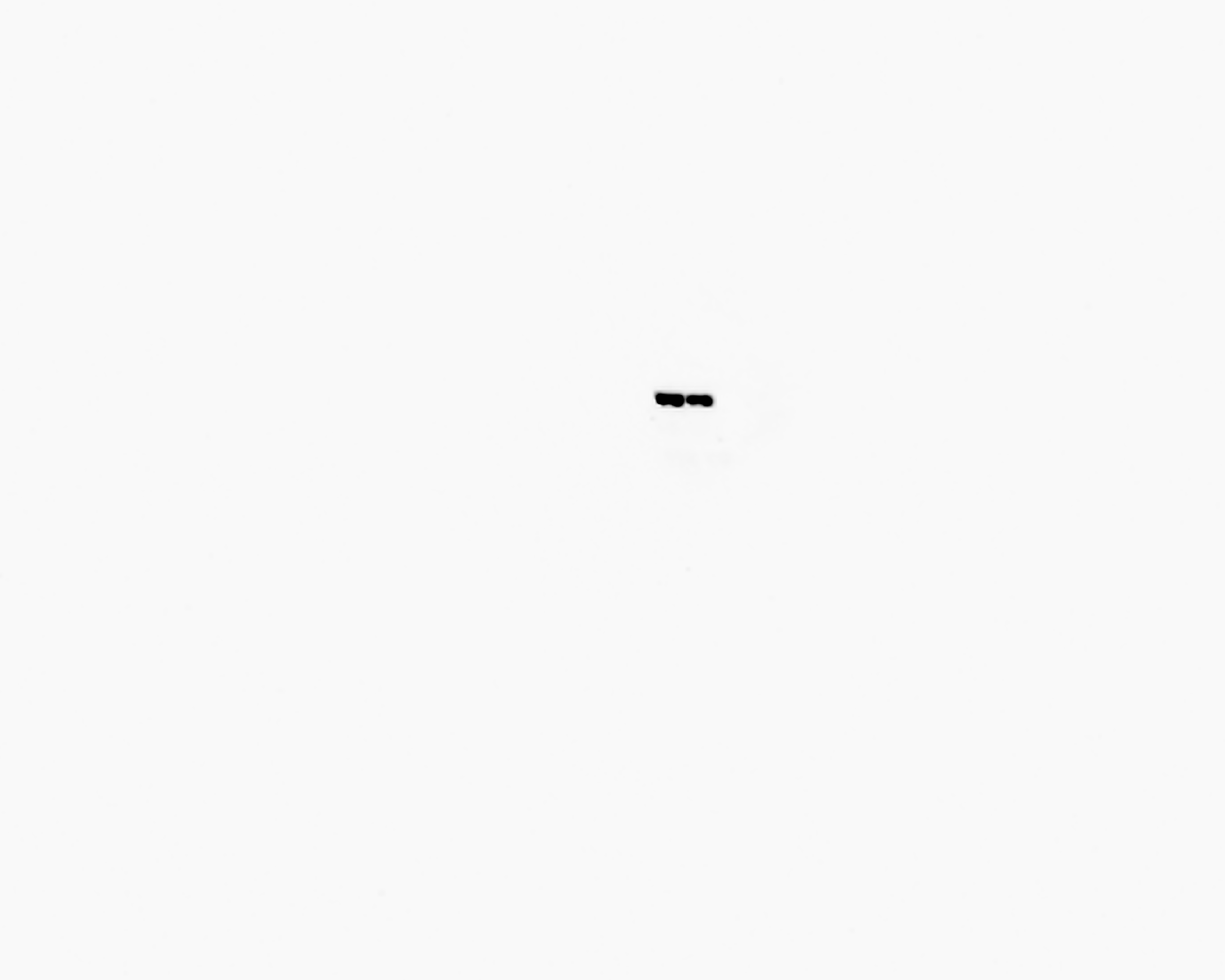

Supplement: Supplementary file 7 — Additional file 7. [file 12964_2024_1475_MOESM7_ESM.zip › Additional file 2/Figure 5H/Eca-109/ip oct4.tif]

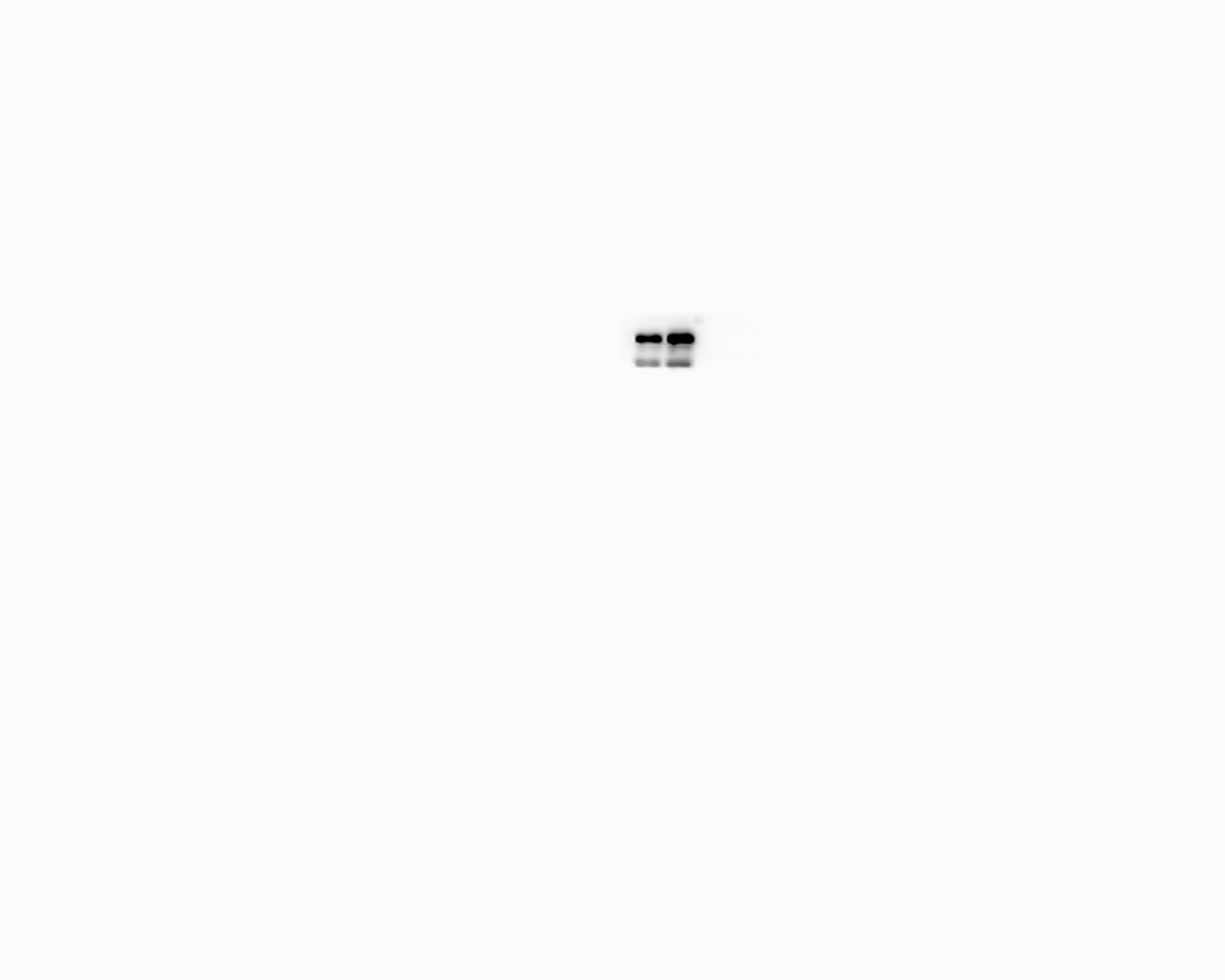

Supplement: Supplementary file 7 — Additional file 7. [file 12964_2024_1475_MOESM7_ESM.zip › Additional file 2/Figure 5H/Eca-109/ip wwp2.tif]

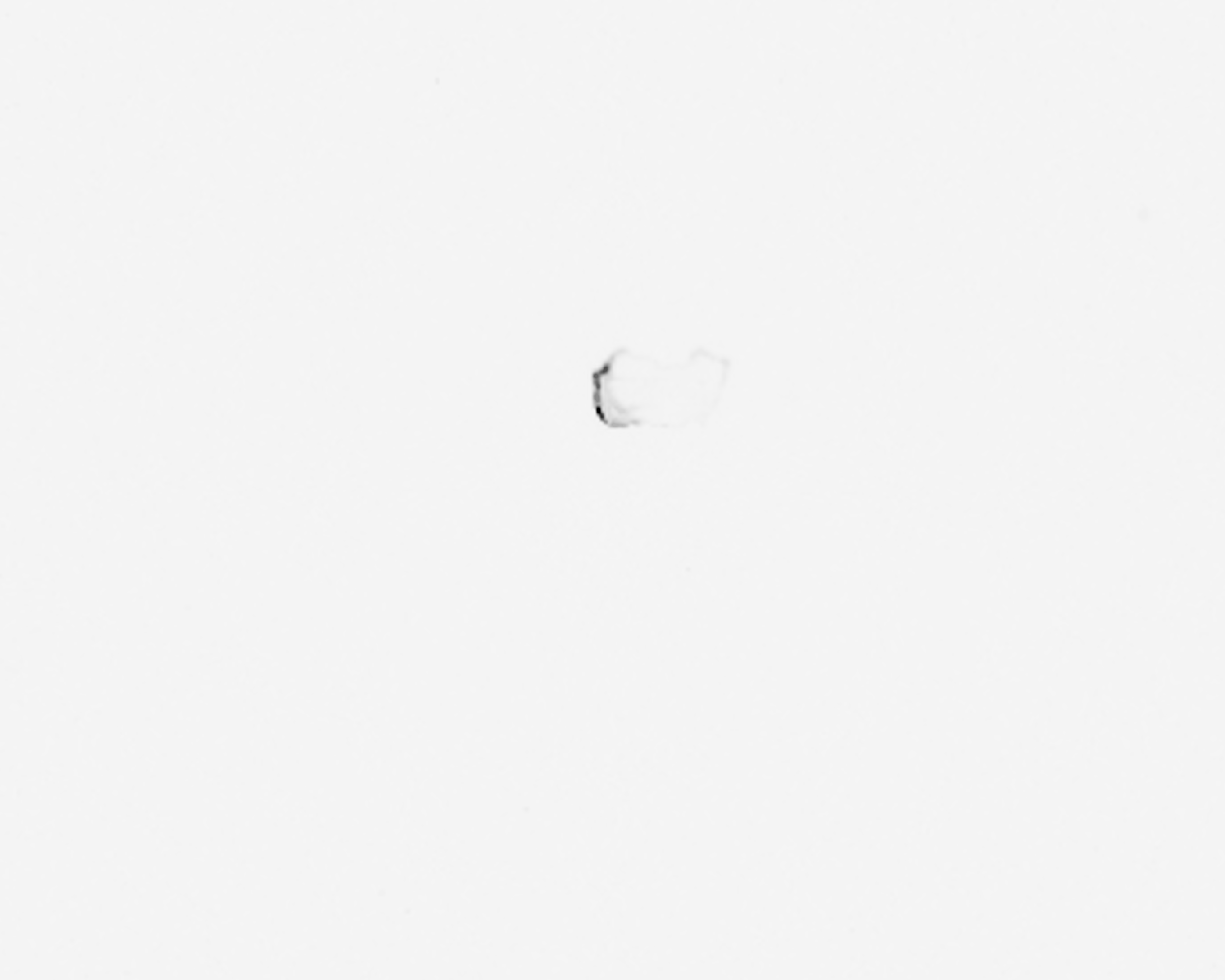

Supplement: Supplementary file 7 — Additional file 7. [file 12964_2024_1475_MOESM7_ESM.zip › Additional file 2/Figure 5H/TE-1/IgG oct4.tif]

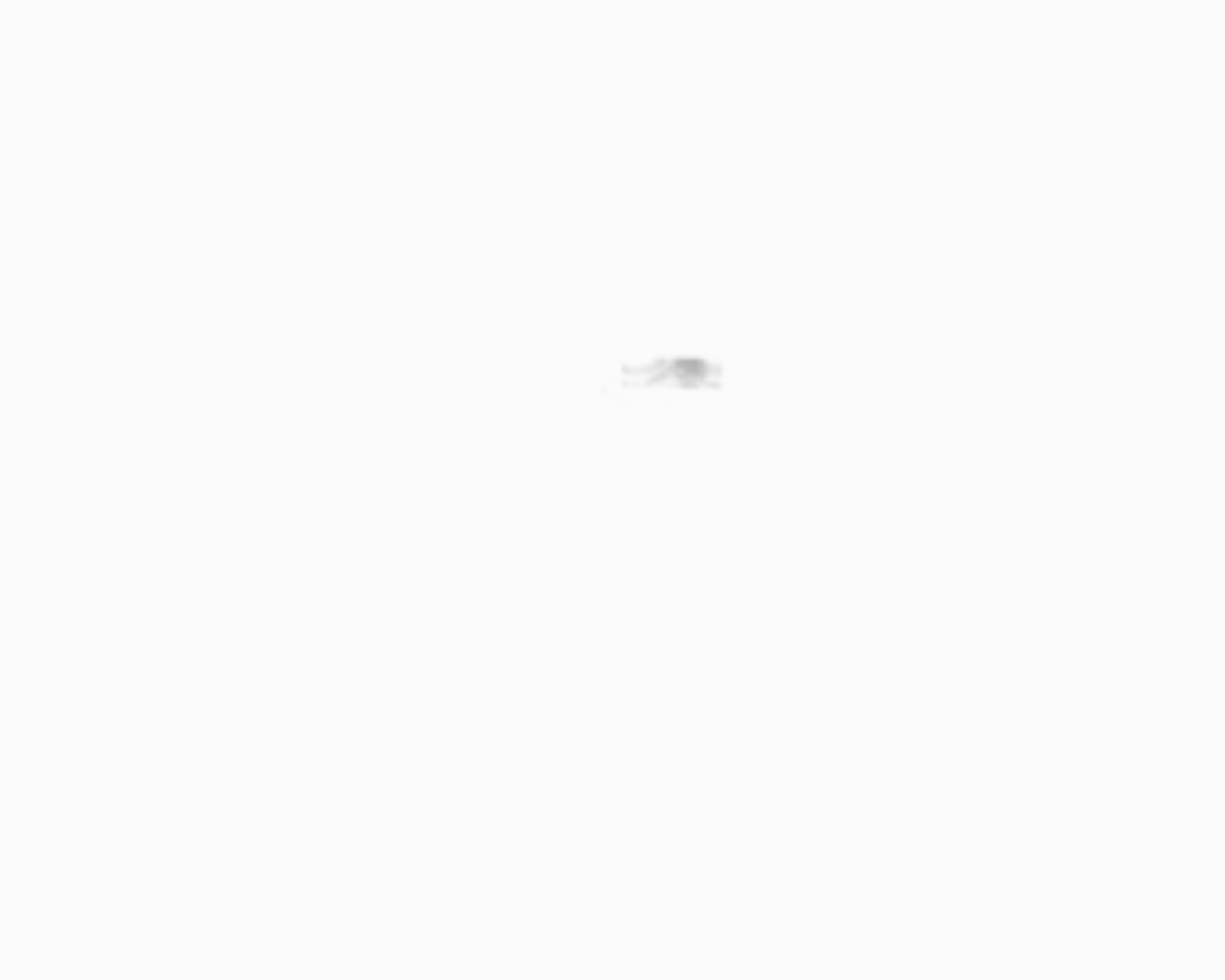

Supplement: Supplementary file 7 — Additional file 7. [file 12964_2024_1475_MOESM7_ESM.zip › Additional file 2/Figure 5H/TE-1/IgG wwp2.tif]

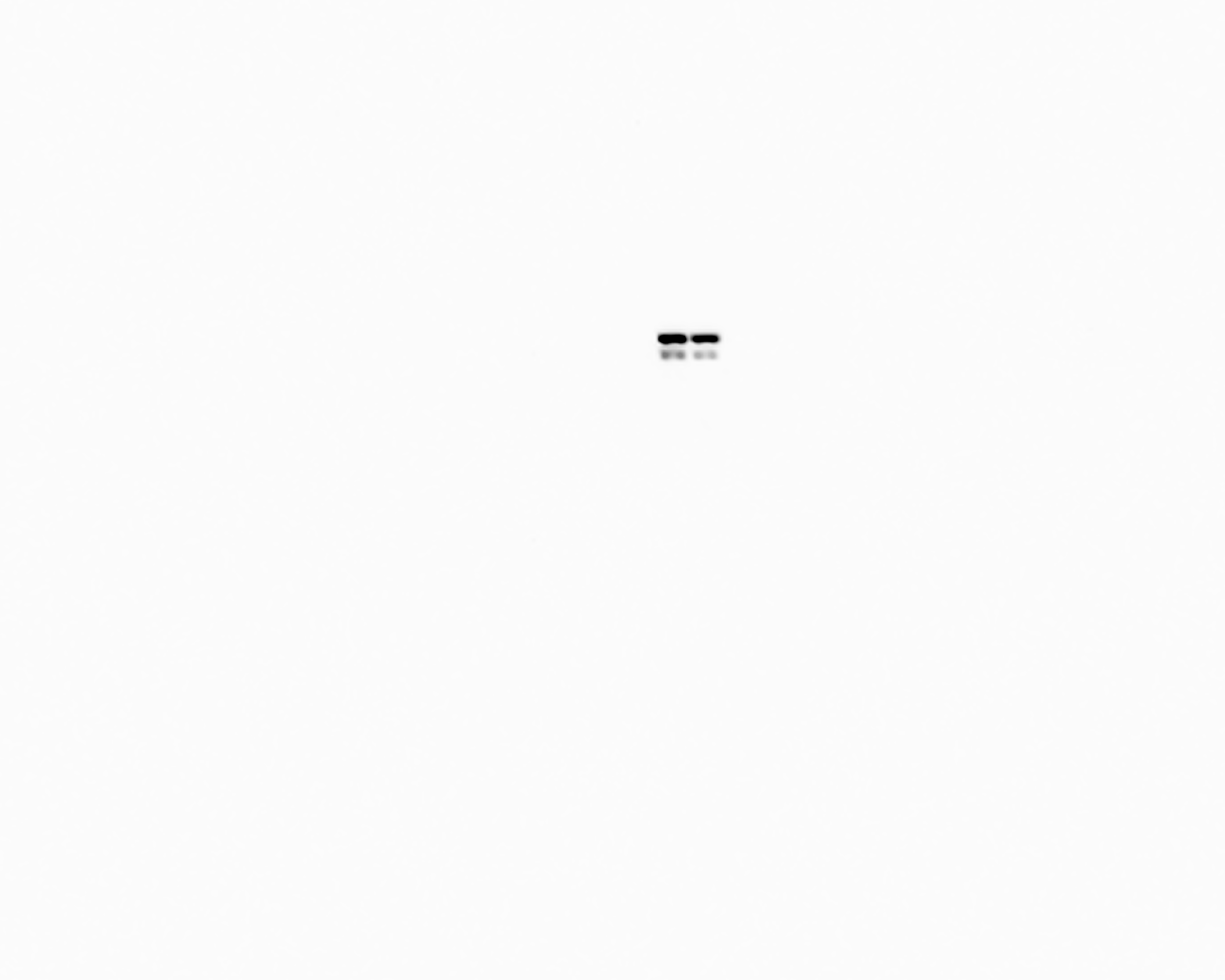

Supplement: Supplementary file 7 — Additional file 7. [file 12964_2024_1475_MOESM7_ESM.zip › Additional file 2/Figure 5H/TE-1/input oct4.tif]

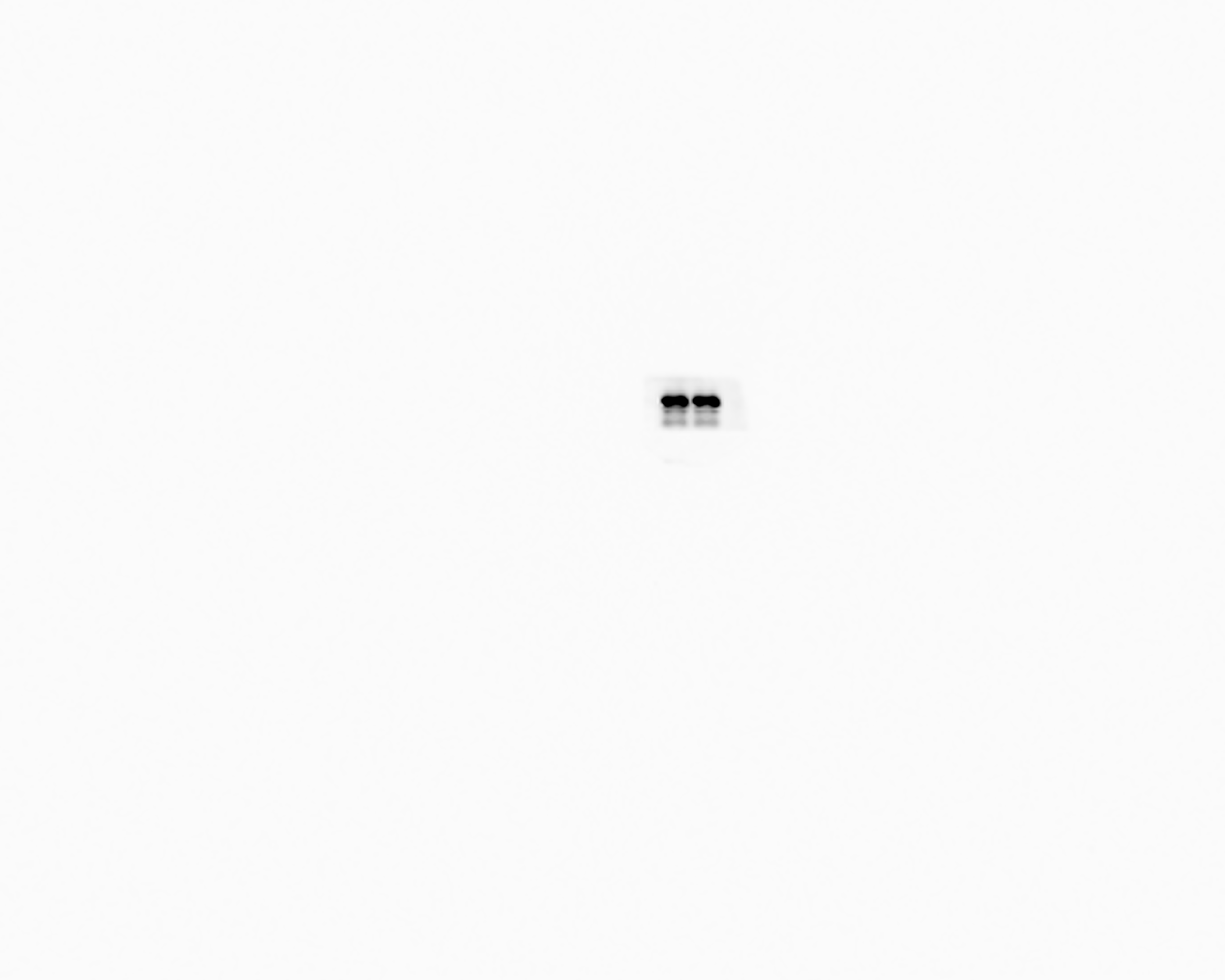

Supplement: Supplementary file 7 — Additional file 7. [file 12964_2024_1475_MOESM7_ESM.zip › Additional file 2/Figure 5H/TE-1/input wwp2.tif]

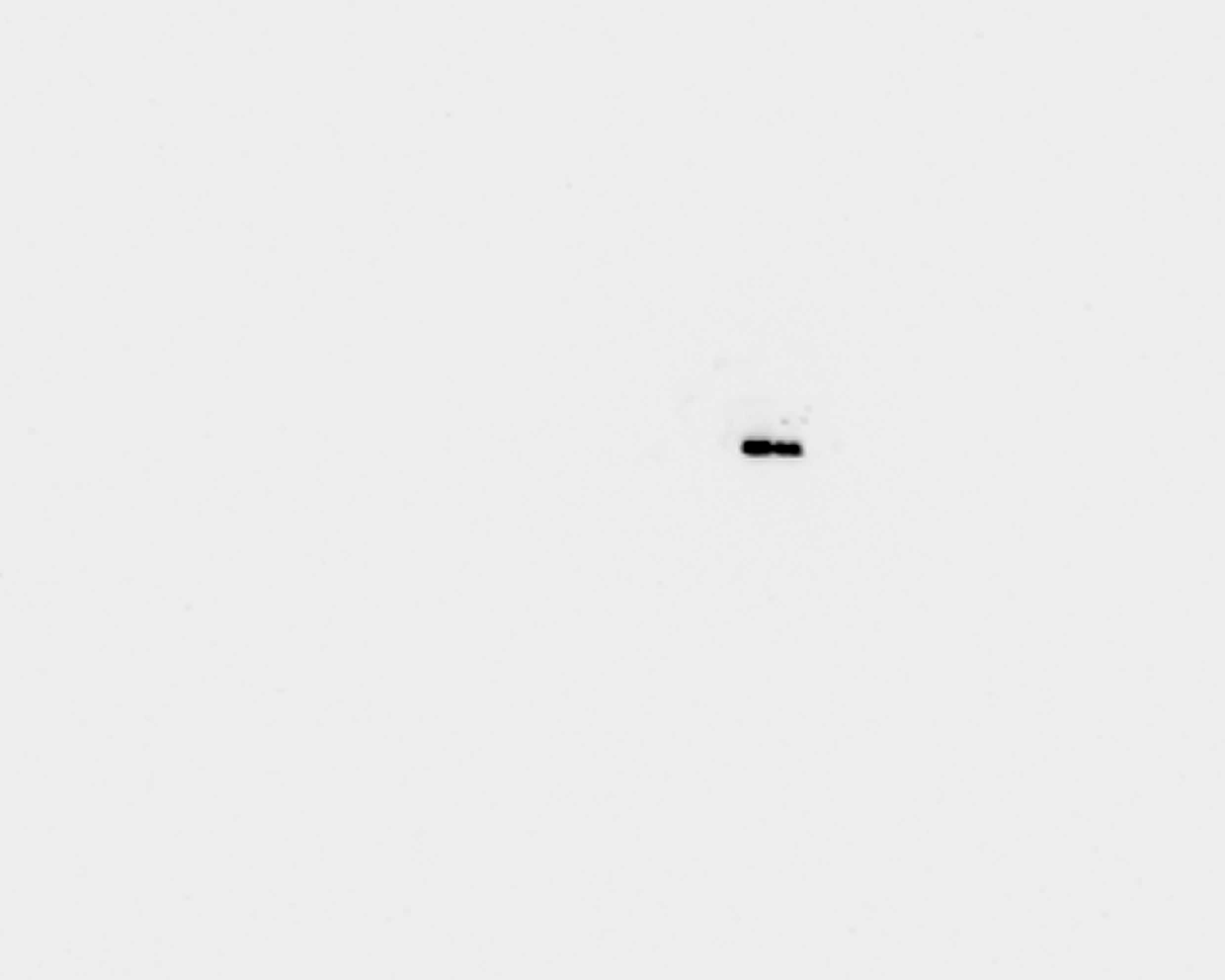

Supplement: Supplementary file 7 — Additional file 7. [file 12964_2024_1475_MOESM7_ESM.zip › Additional file 2/Figure 5H/TE-1/ip OCT4.tif]

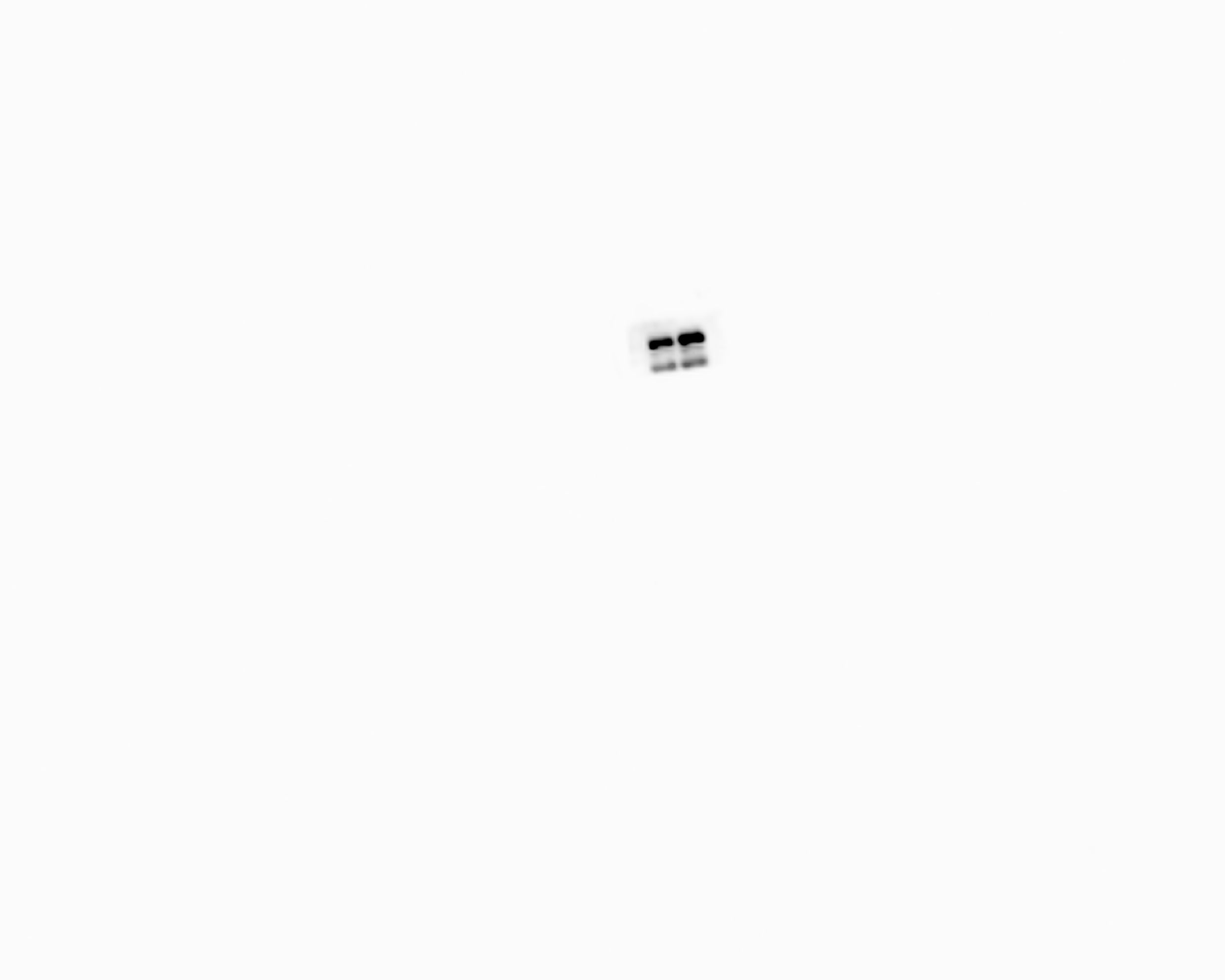

Supplement: Supplementary file 7 — Additional file 7. [file 12964_2024_1475_MOESM7_ESM.zip › Additional file 2/Figure 5H/TE-1/ip wwp2.tif]

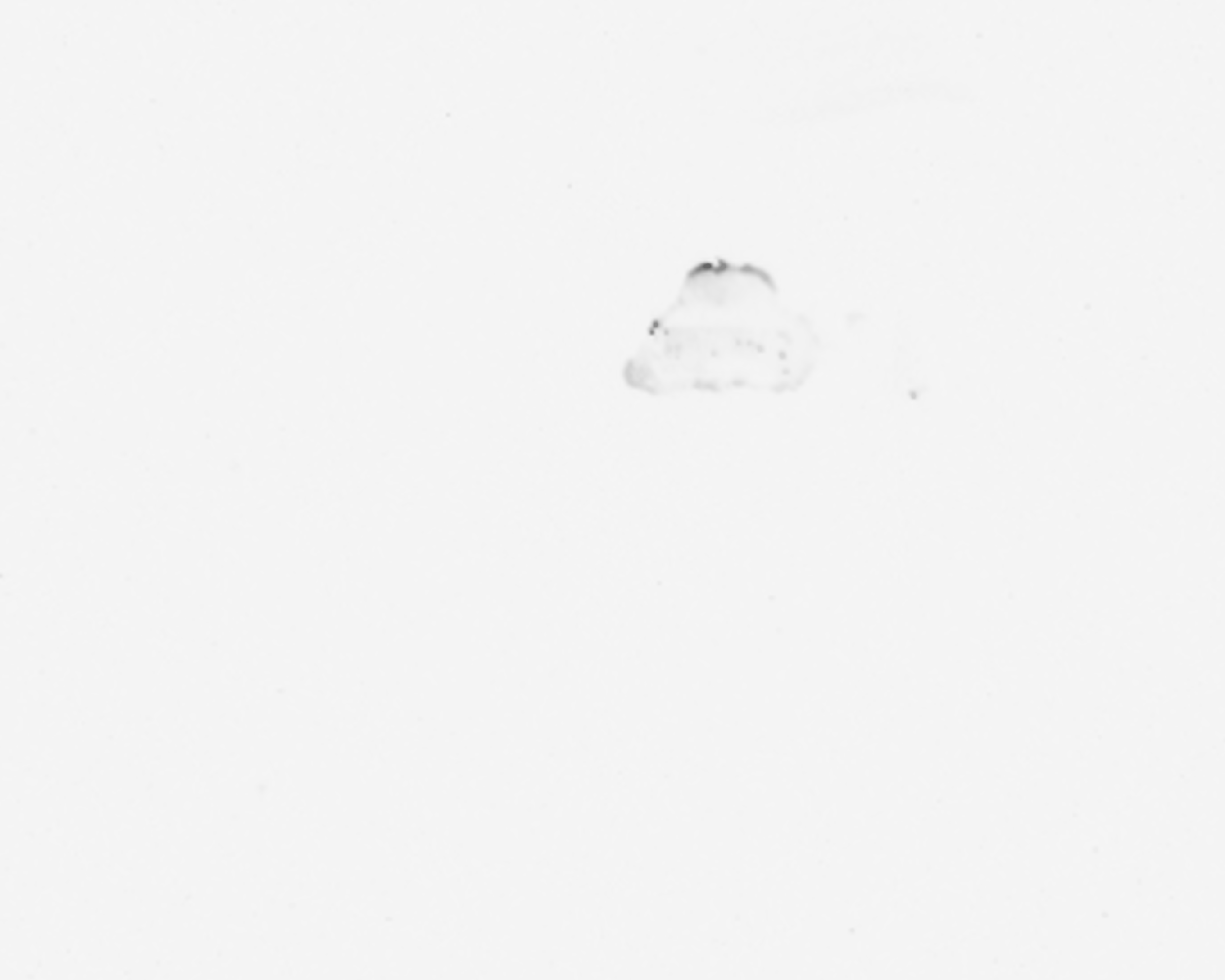

Supplement: Supplementary file 7 — Additional file 7. [file 12964_2024_1475_MOESM7_ESM.zip › Additional file 2/Figure 5I/KYSE-150/IgG oct4.tif]

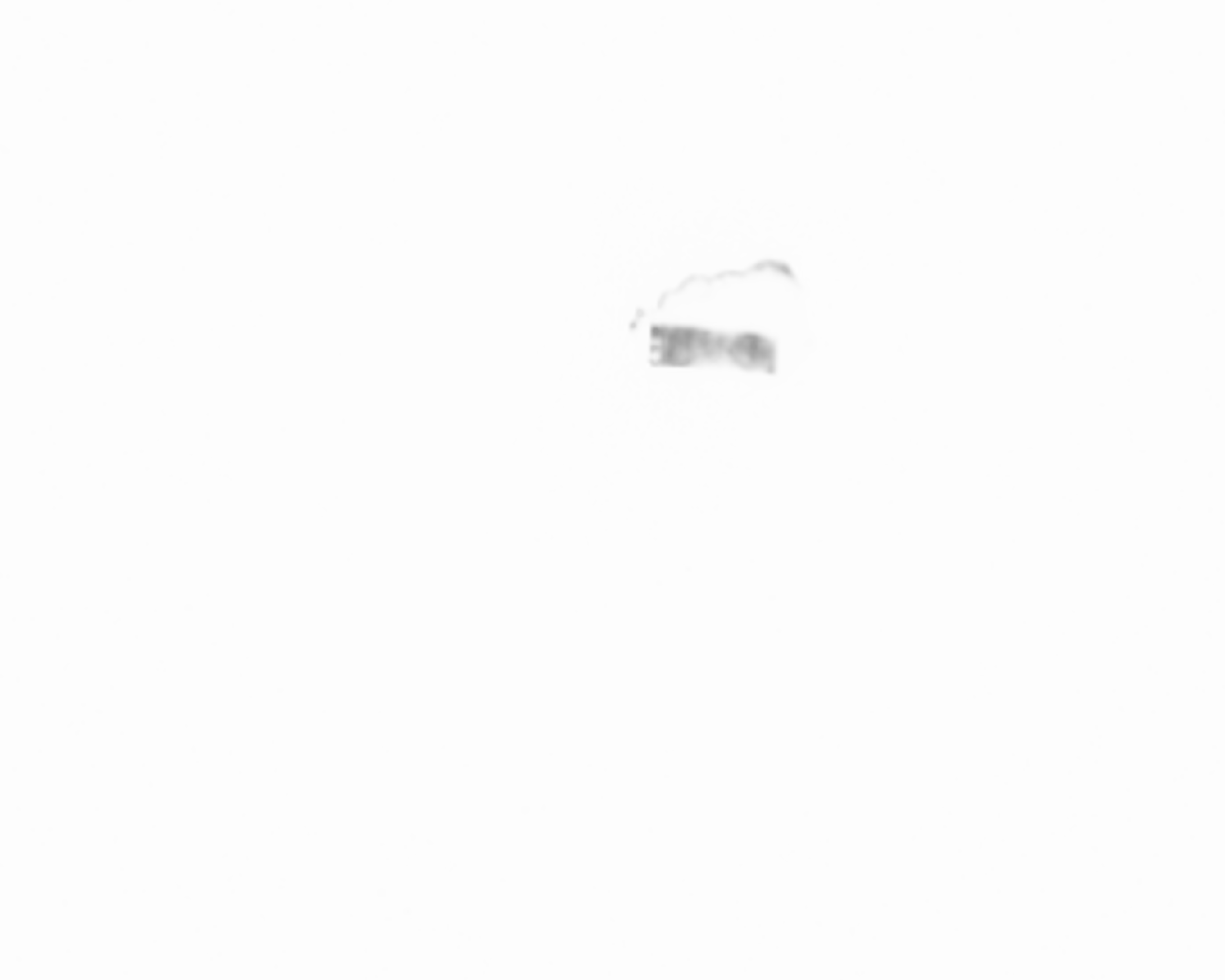

Supplement: Supplementary file 7 — Additional file 7. [file 12964_2024_1475_MOESM7_ESM.zip › Additional file 2/Figure 5I/KYSE-150/IgG wwp2.tif]

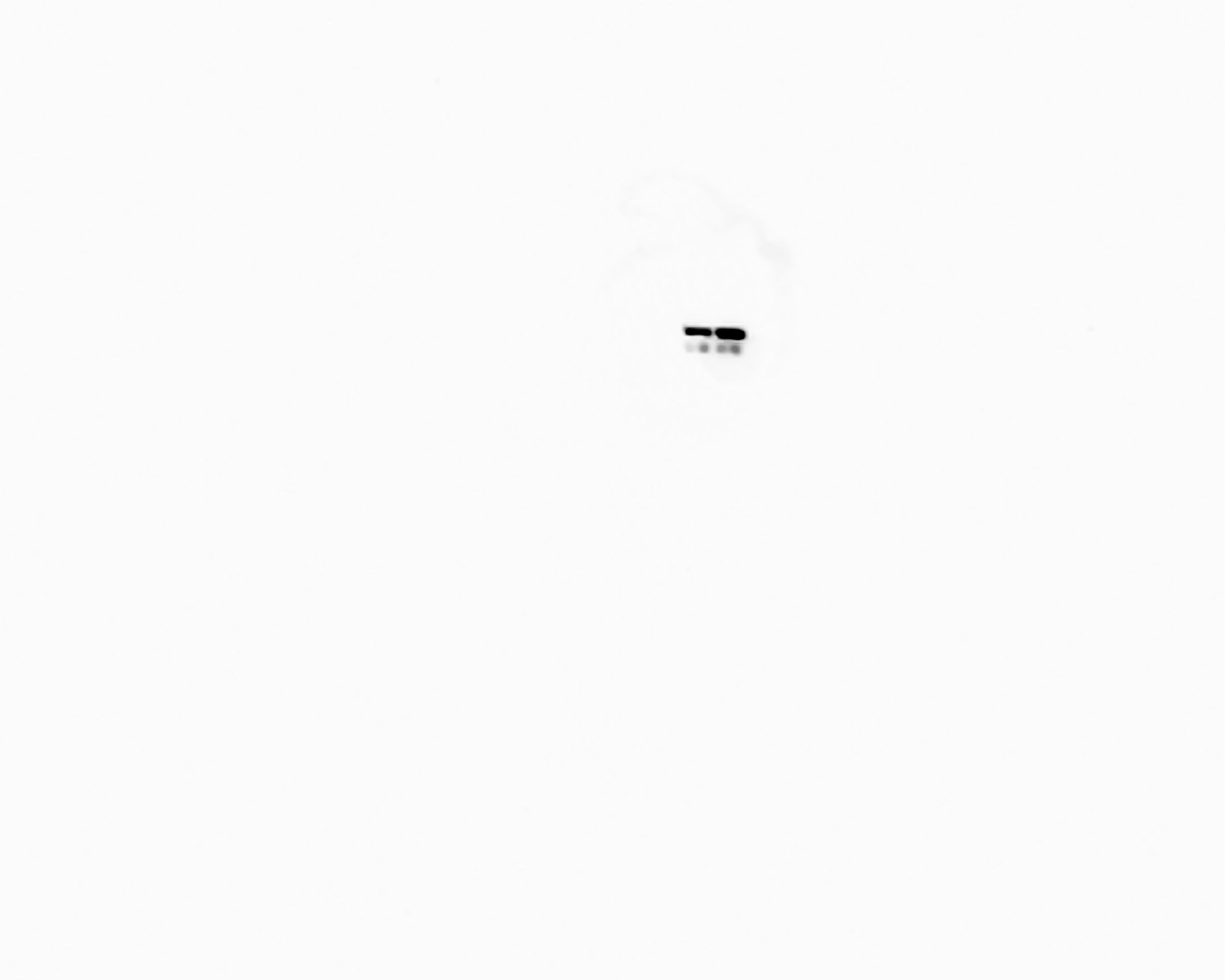

Supplement: Supplementary file 7 — Additional file 7. [file 12964_2024_1475_MOESM7_ESM.zip › Additional file 2/Figure 5I/KYSE-150/input oct4.tif]

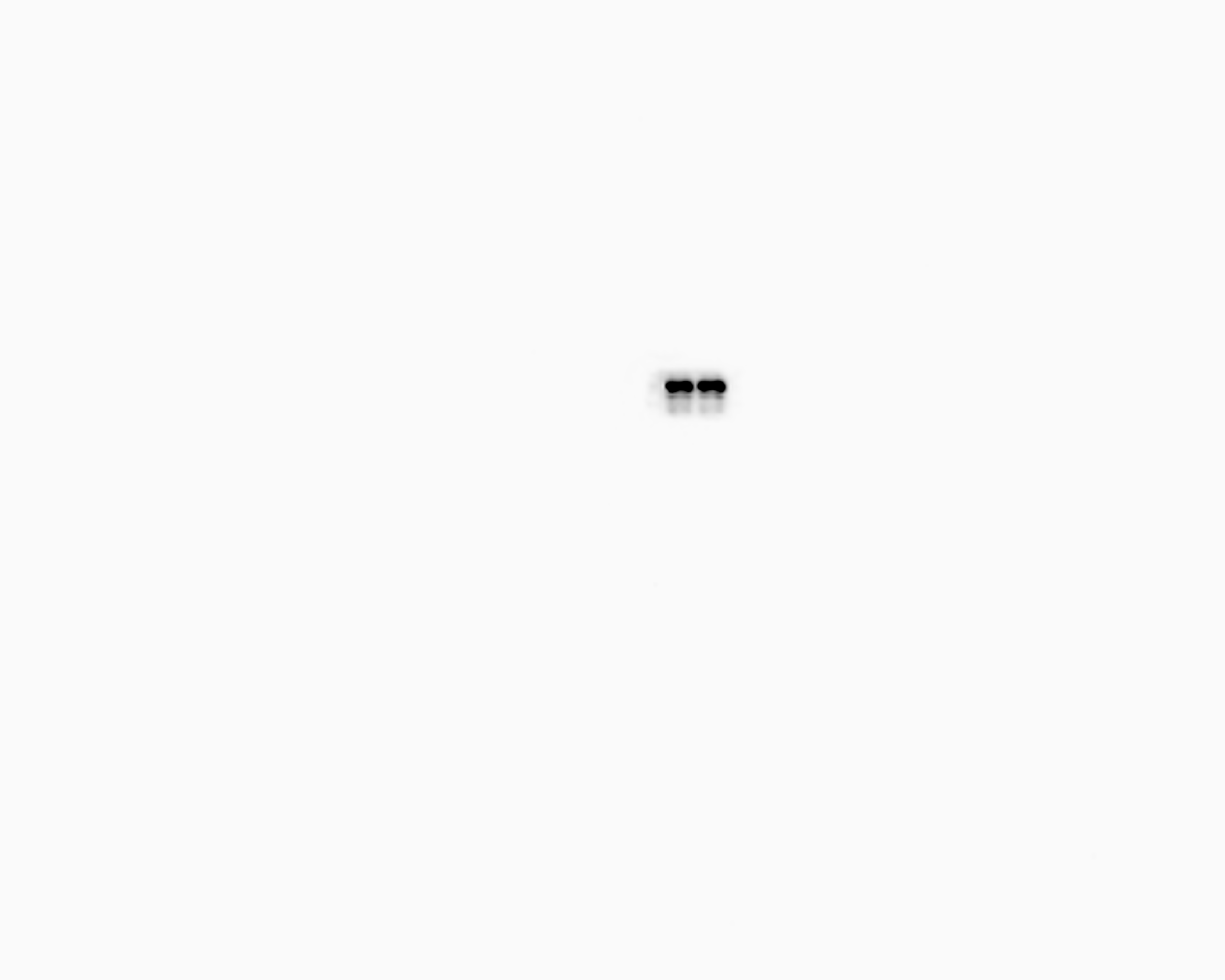

Supplement: Supplementary file 7 — Additional file 7. [file 12964_2024_1475_MOESM7_ESM.zip › Additional file 2/Figure 5I/KYSE-150/input wwp2.tif]

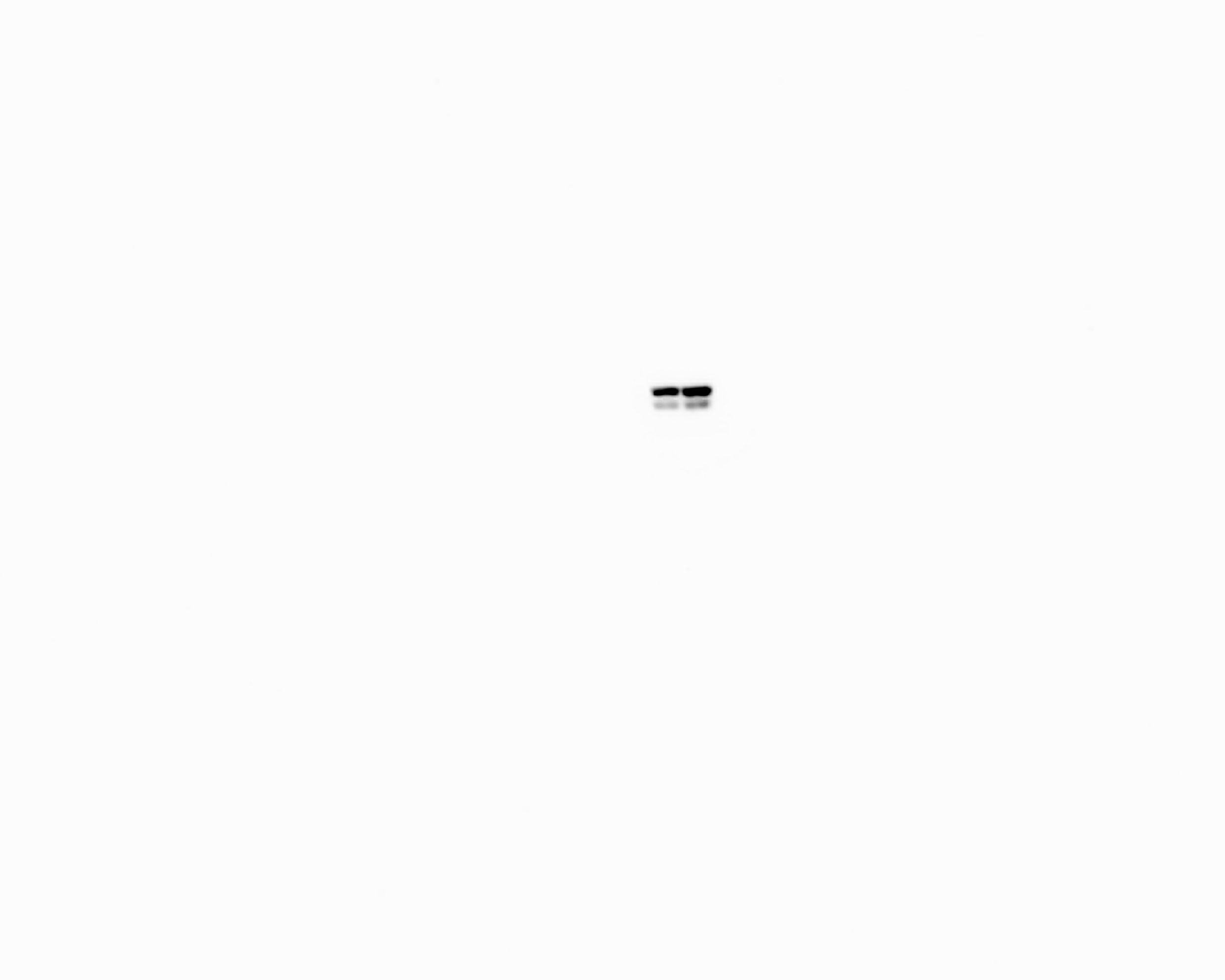

Supplement: Supplementary file 7 — Additional file 7. [file 12964_2024_1475_MOESM7_ESM.zip › Additional file 2/Figure 5I/KYSE-150/ip oct4.tif]

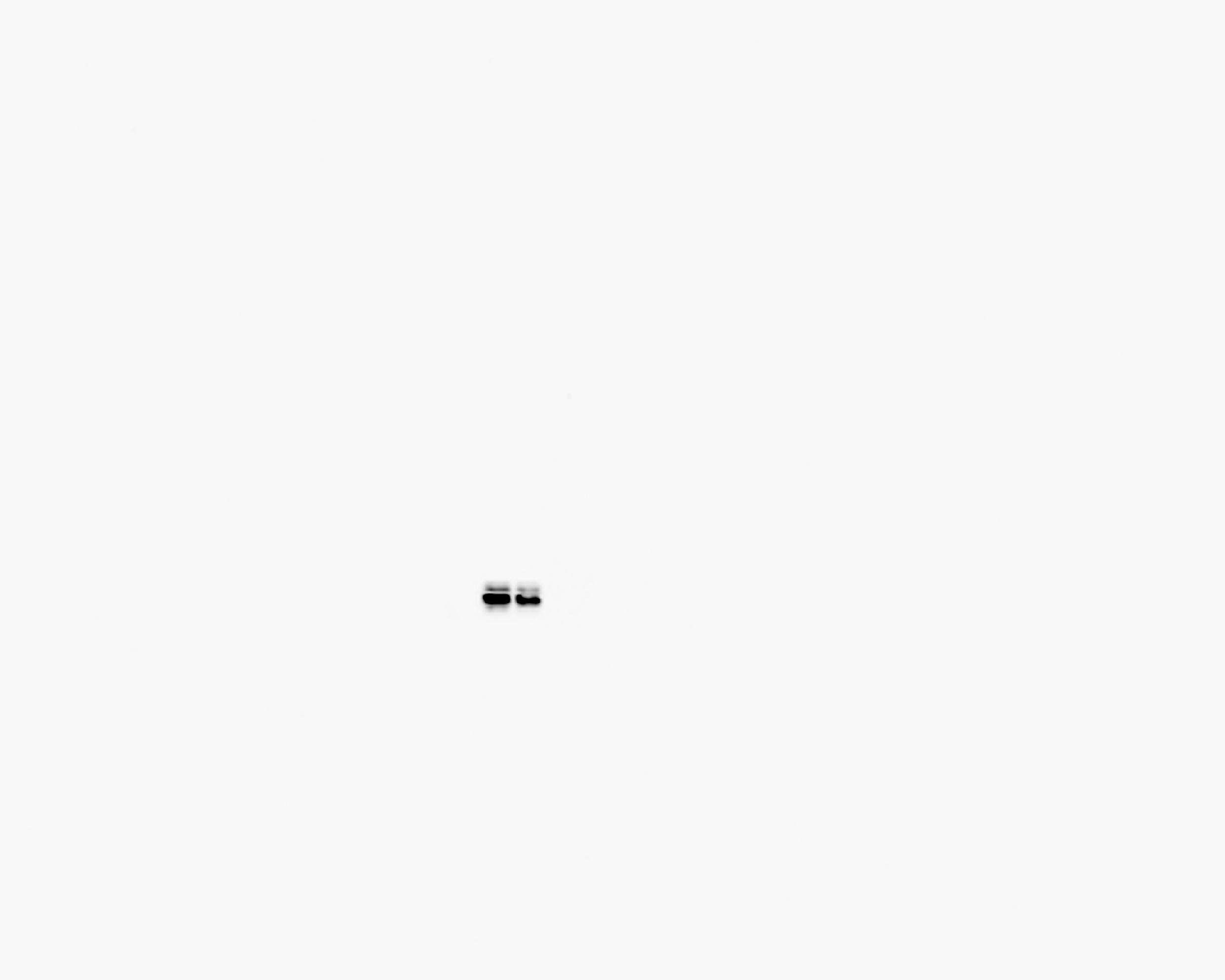

Supplement: Supplementary file 7 — Additional file 7. [file 12964_2024_1475_MOESM7_ESM.zip › Additional file 2/Figure 5I/KYSE-150/ip wwp2.tif]

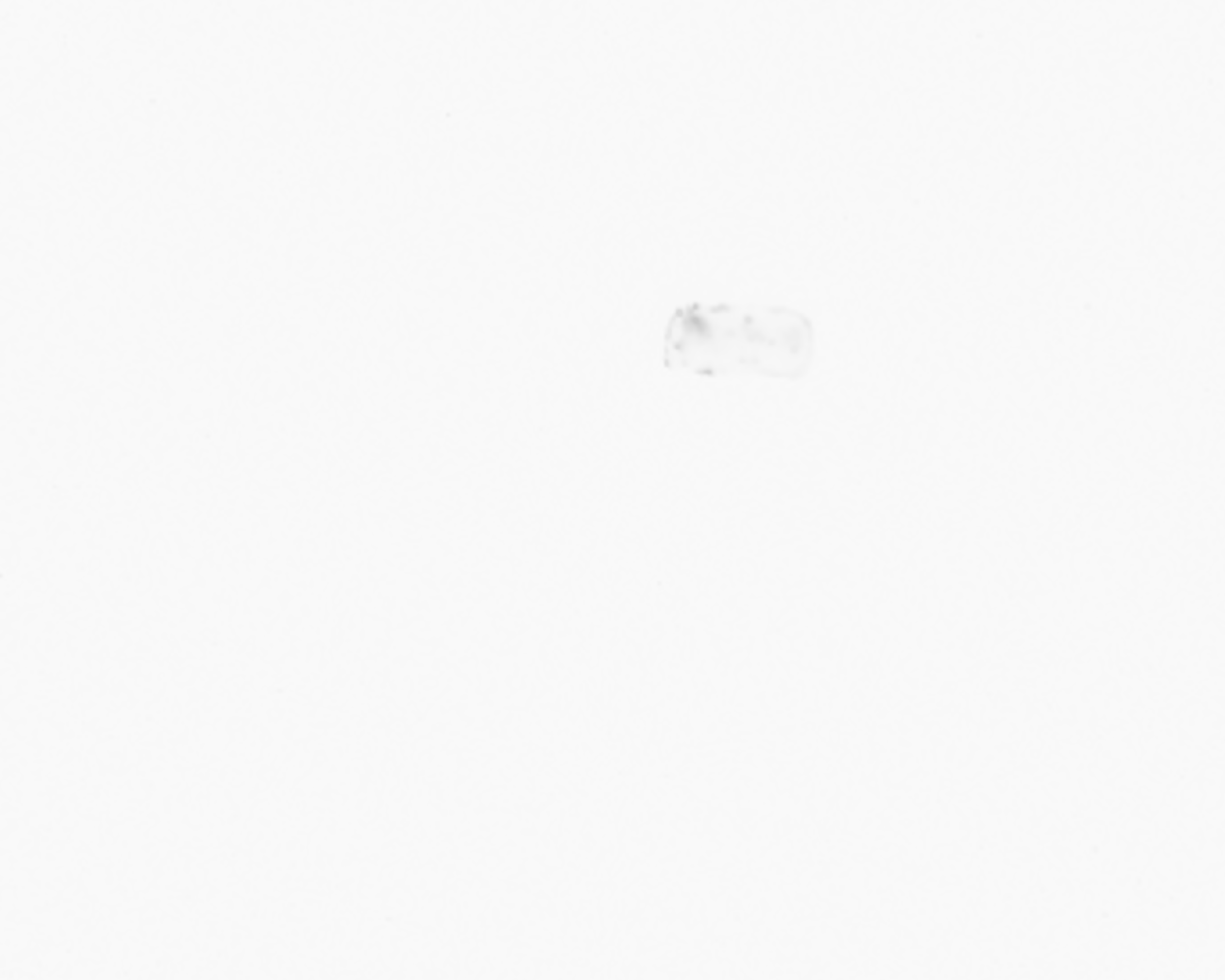

Supplement: Supplementary file 7 — Additional file 7. [file 12964_2024_1475_MOESM7_ESM.zip › Additional file 2/Figure 5I/KYSE-30/IgG oct4.tif]

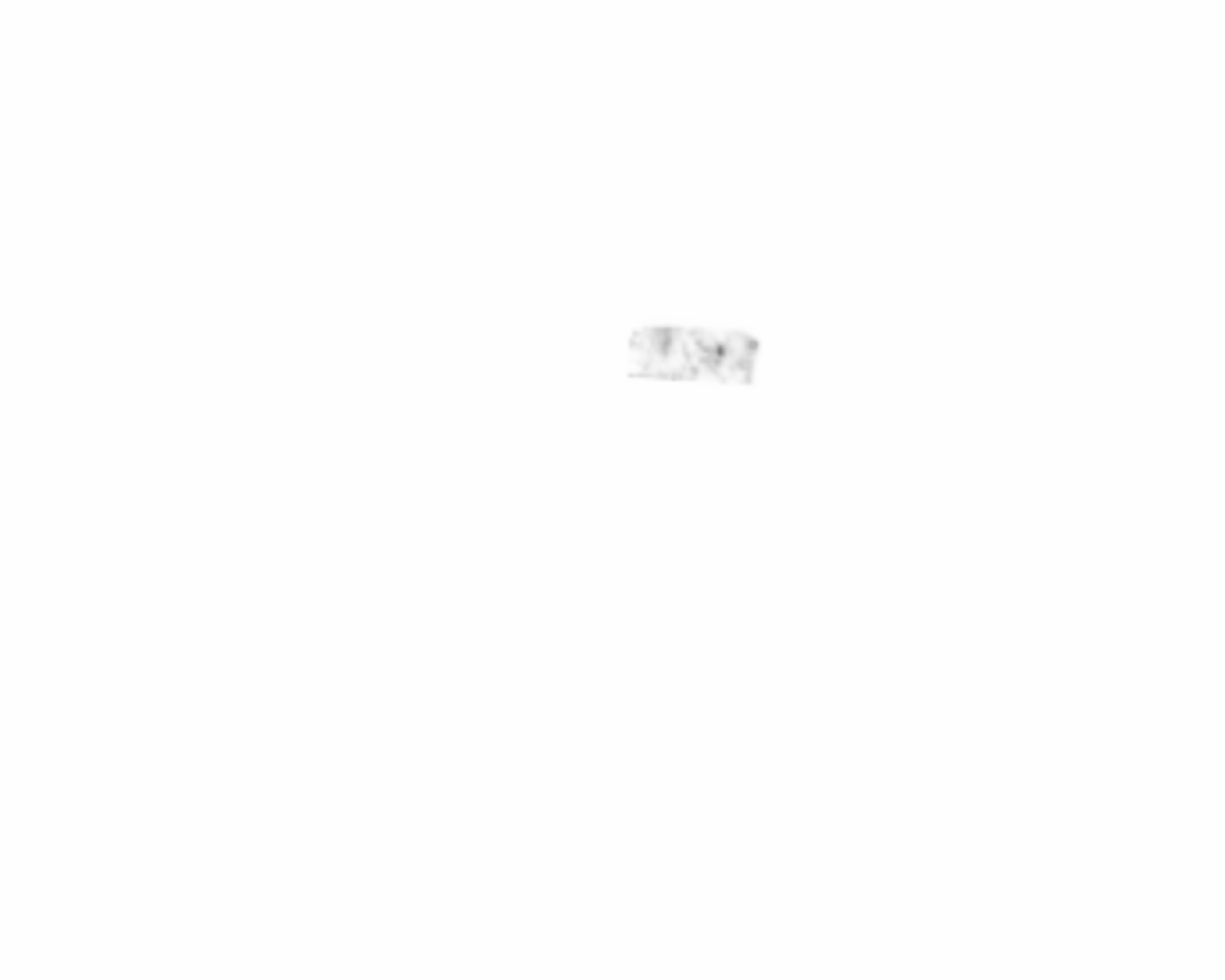

Supplement: Supplementary file 7 — Additional file 7. [file 12964_2024_1475_MOESM7_ESM.zip › Additional file 2/Figure 5I/KYSE-30/IgG wwp2.tif]

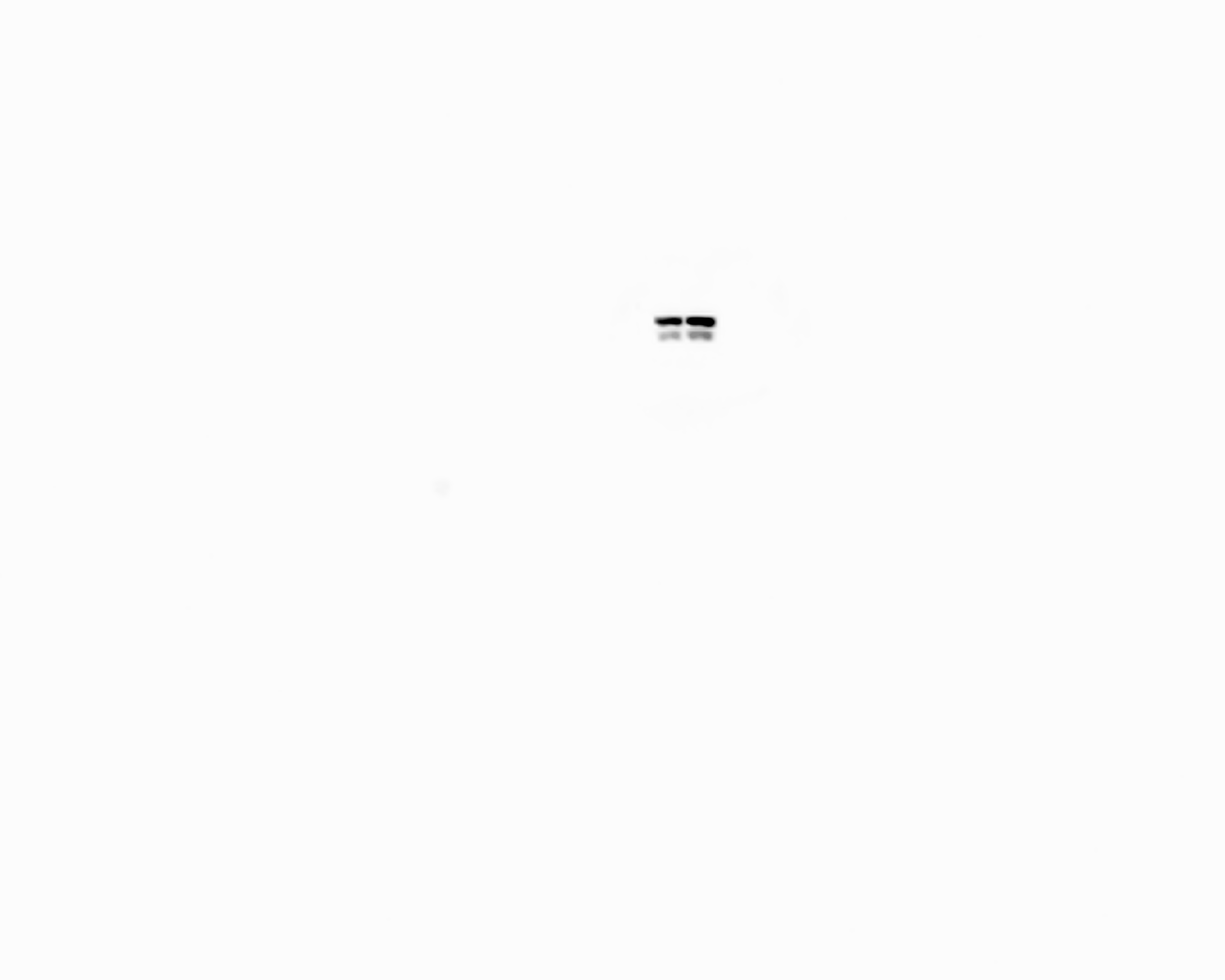

Supplement: Supplementary file 7 — Additional file 7. [file 12964_2024_1475_MOESM7_ESM.zip › Additional file 2/Figure 5I/KYSE-30/input oct4.tif]

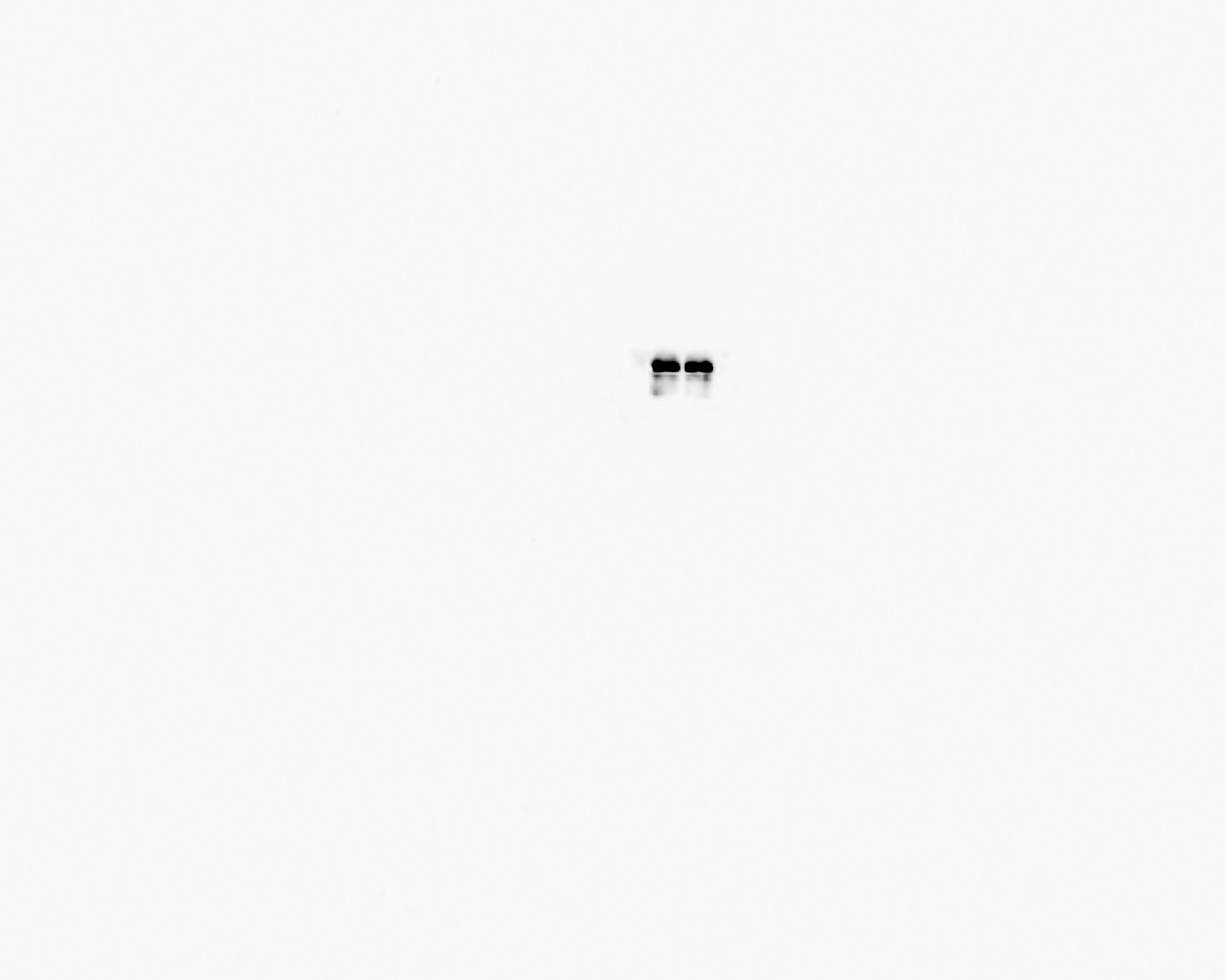

Supplement: Supplementary file 7 — Additional file 7. [file 12964_2024_1475_MOESM7_ESM.zip › Additional file 2/Figure 5I/KYSE-30/input wwp2.tif]

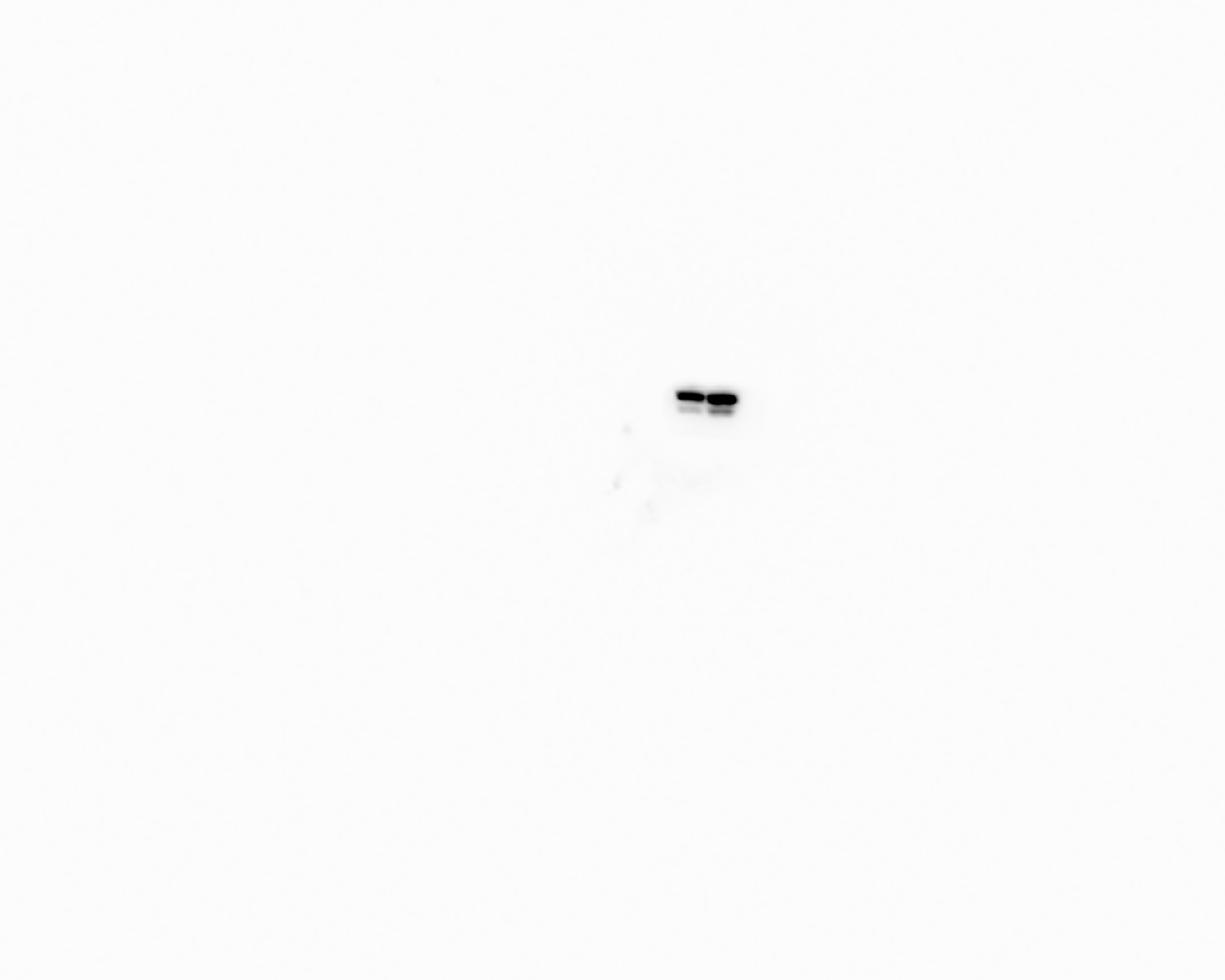

Supplement: Supplementary file 7 — Additional file 7. [file 12964_2024_1475_MOESM7_ESM.zip › Additional file 2/Figure 5I/KYSE-30/ip oct4.tif]

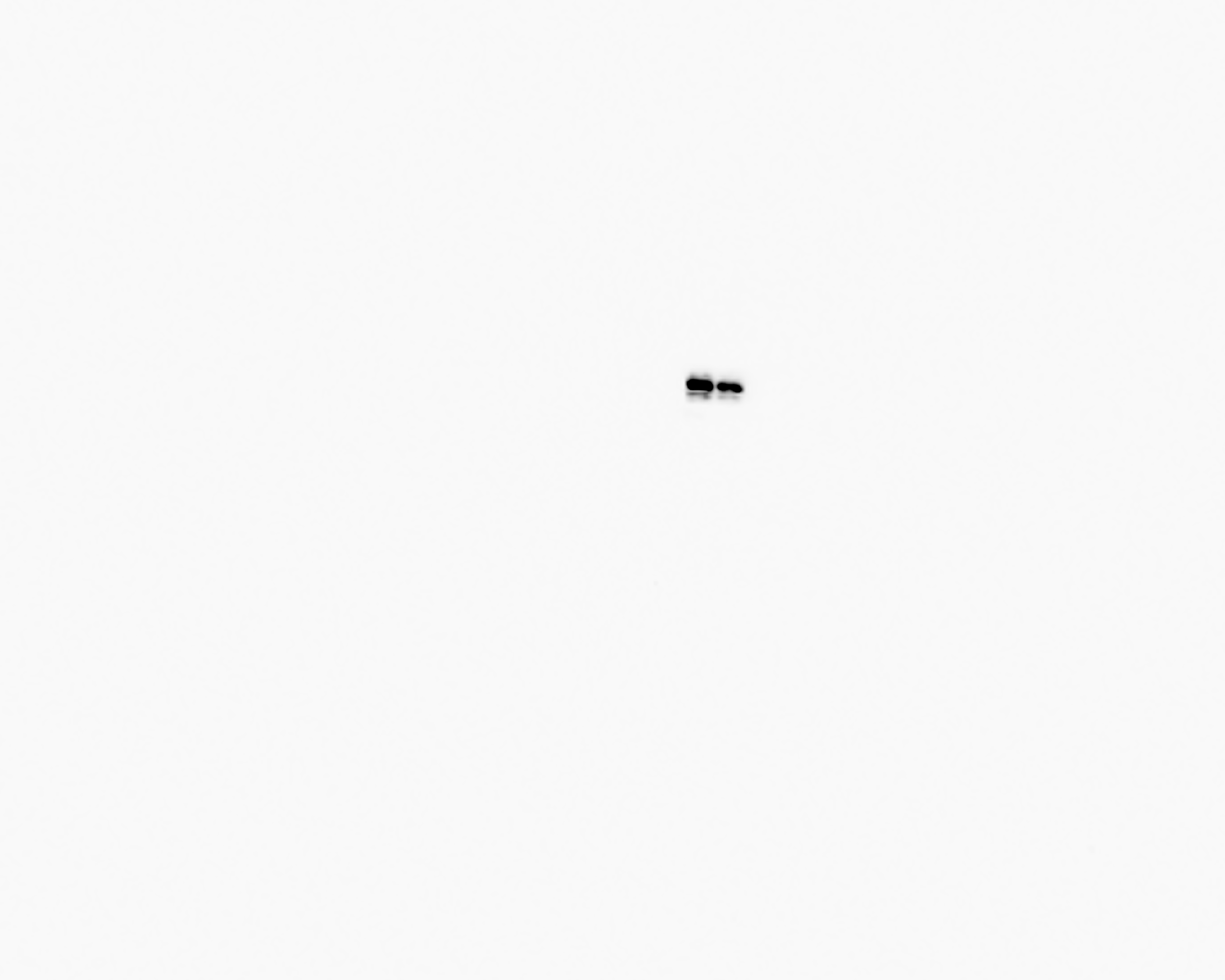

Supplement: Supplementary file 7 — Additional file 7. [file 12964_2024_1475_MOESM7_ESM.zip › Additional file 2/Figure 5I/KYSE-30/ip wwp2.tif]

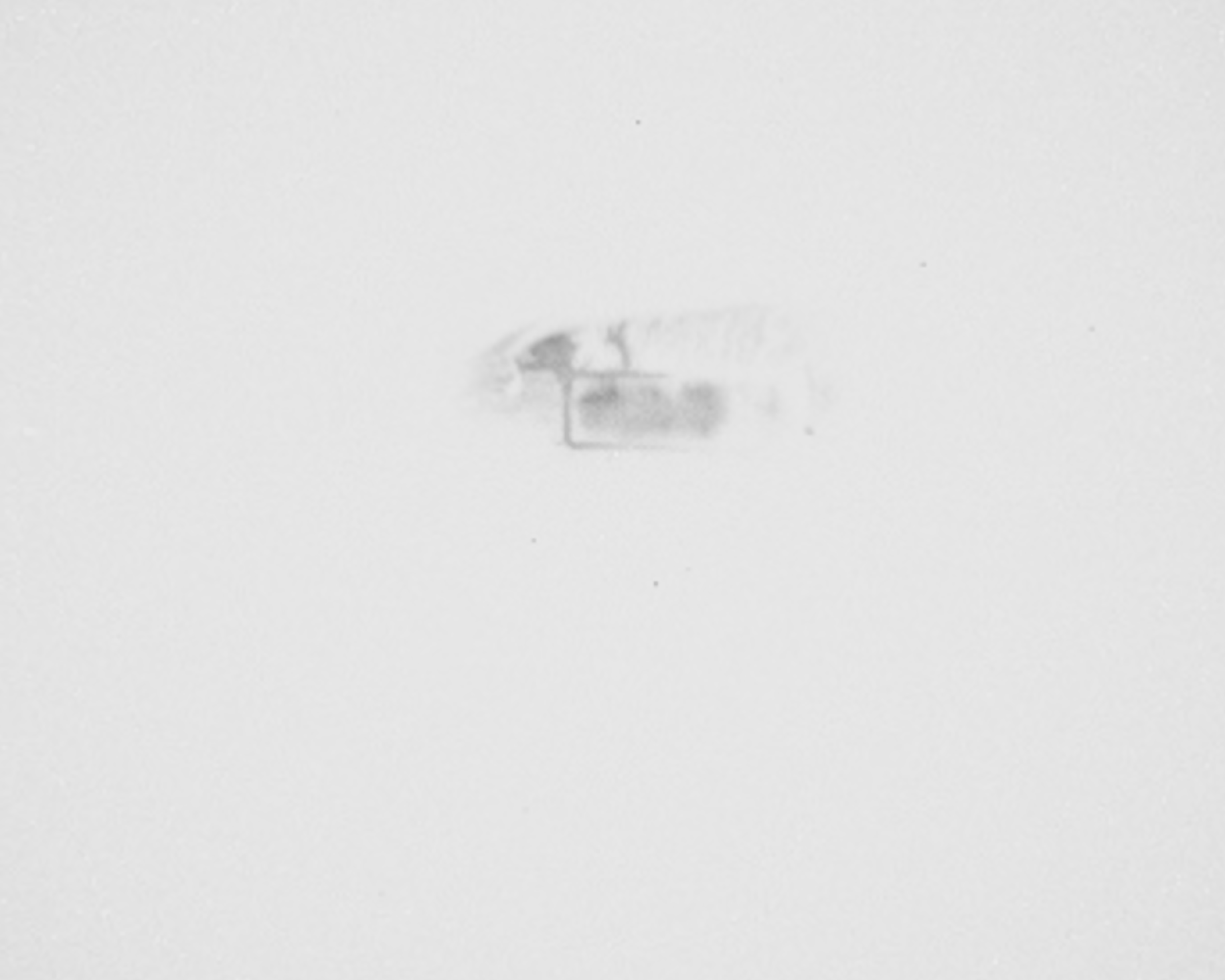

Supplement: Supplementary file 7 — Additional file 7. [file 12964_2024_1475_MOESM7_ESM.zip › Additional file 2/Figure 5J/KYSE-150/IgG oct4.tif]

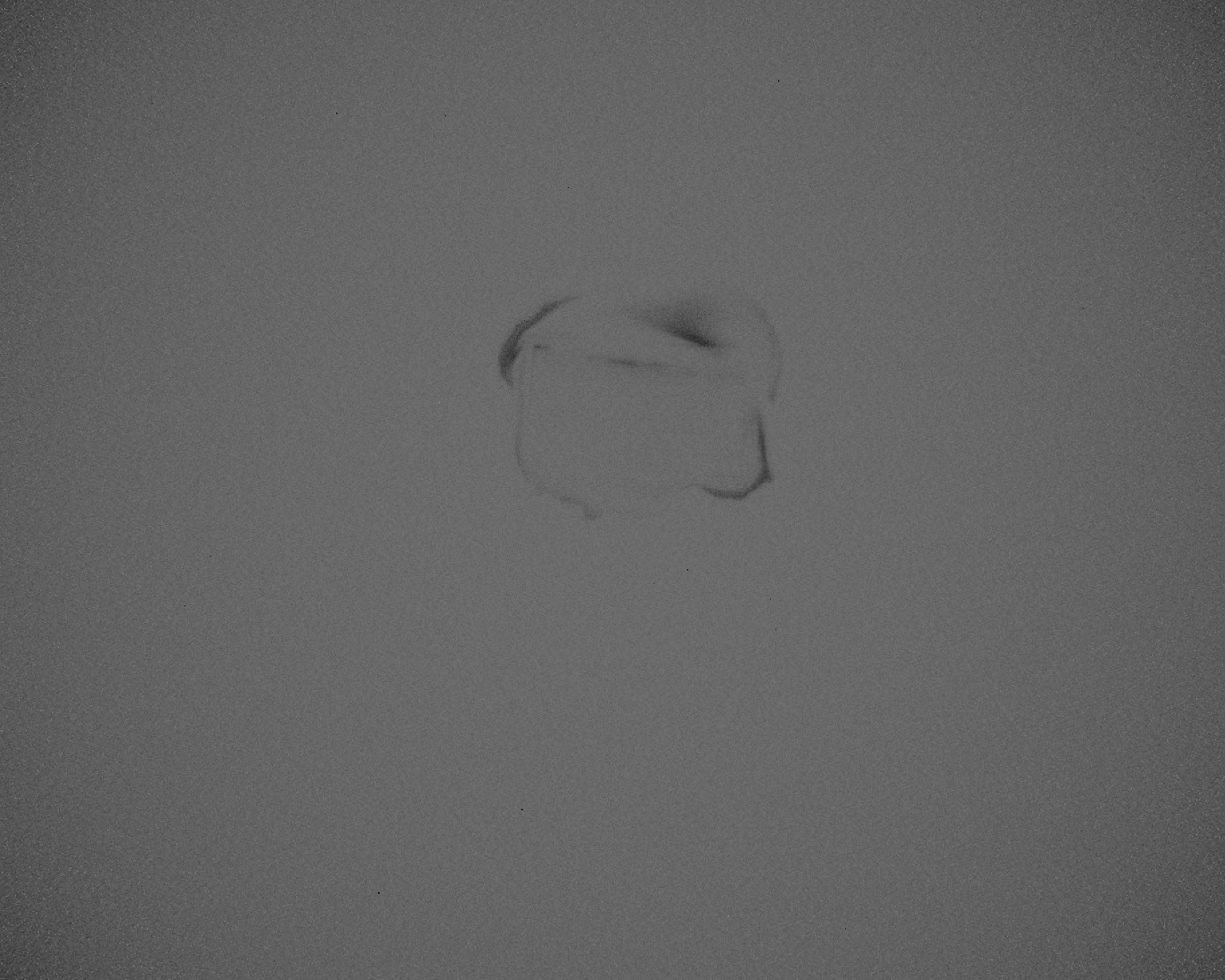

Supplement: Supplementary file 7 — Additional file 7. [file 12964_2024_1475_MOESM7_ESM.zip › Additional file 2/Figure 5J/KYSE-150/IgG ubiquitin.tif]

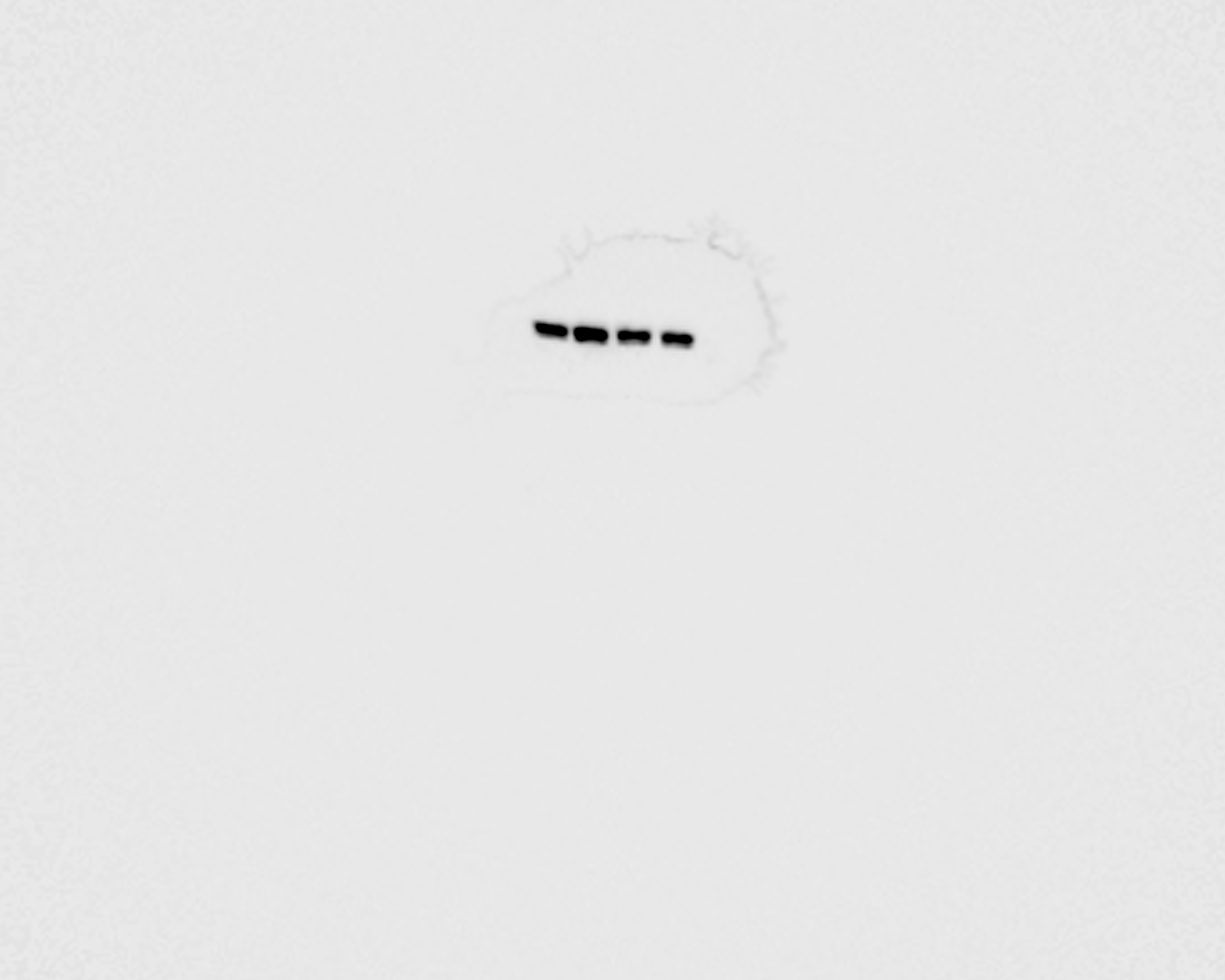

Supplement: Supplementary file 7 — Additional file 7. [file 12964_2024_1475_MOESM7_ESM.zip › Additional file 2/Figure 5J/KYSE-150/input oct4.tif]

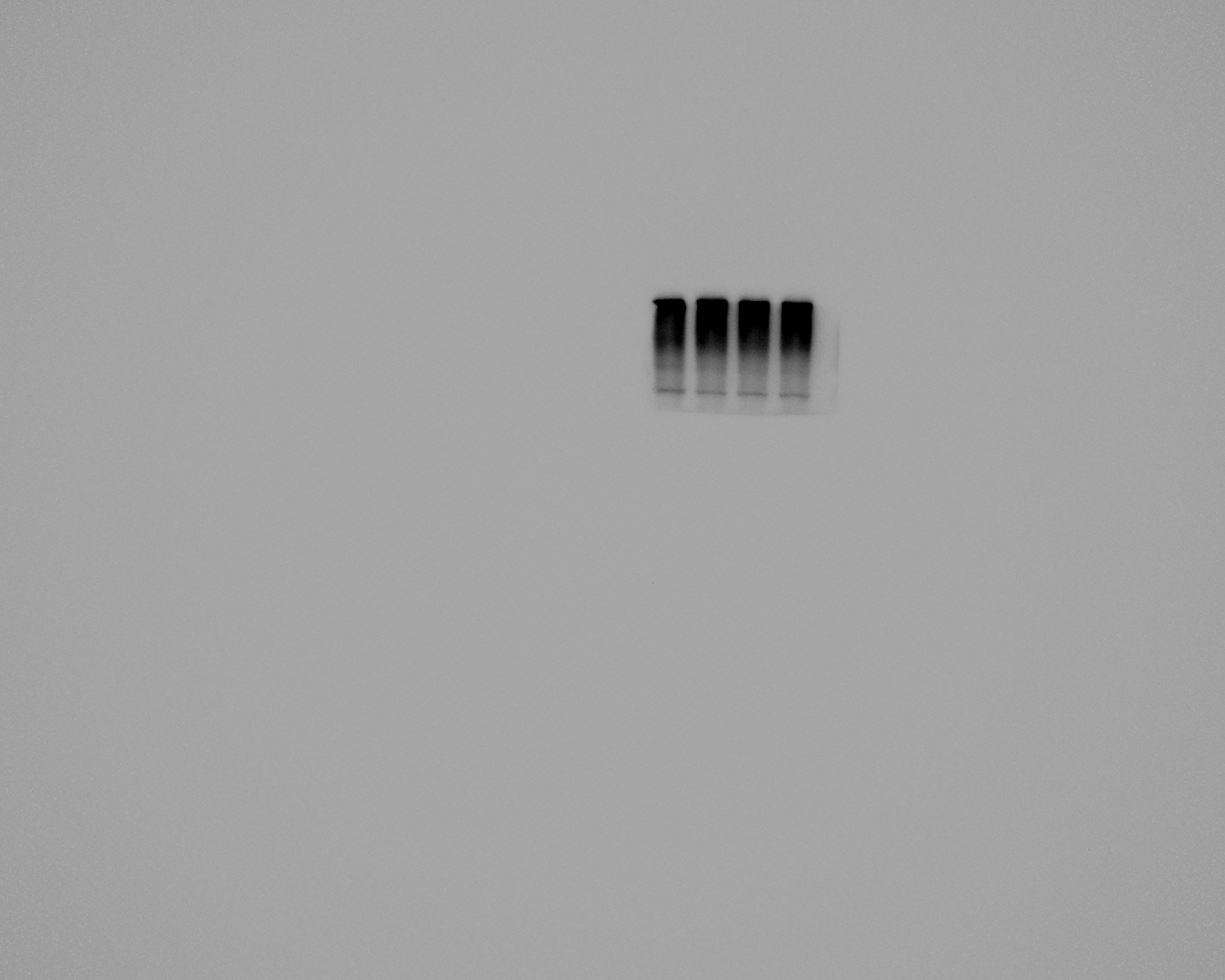

Supplement: Supplementary file 7 — Additional file 7. [file 12964_2024_1475_MOESM7_ESM.zip › Additional file 2/Figure 5J/KYSE-150/input ubiquitin.tif]

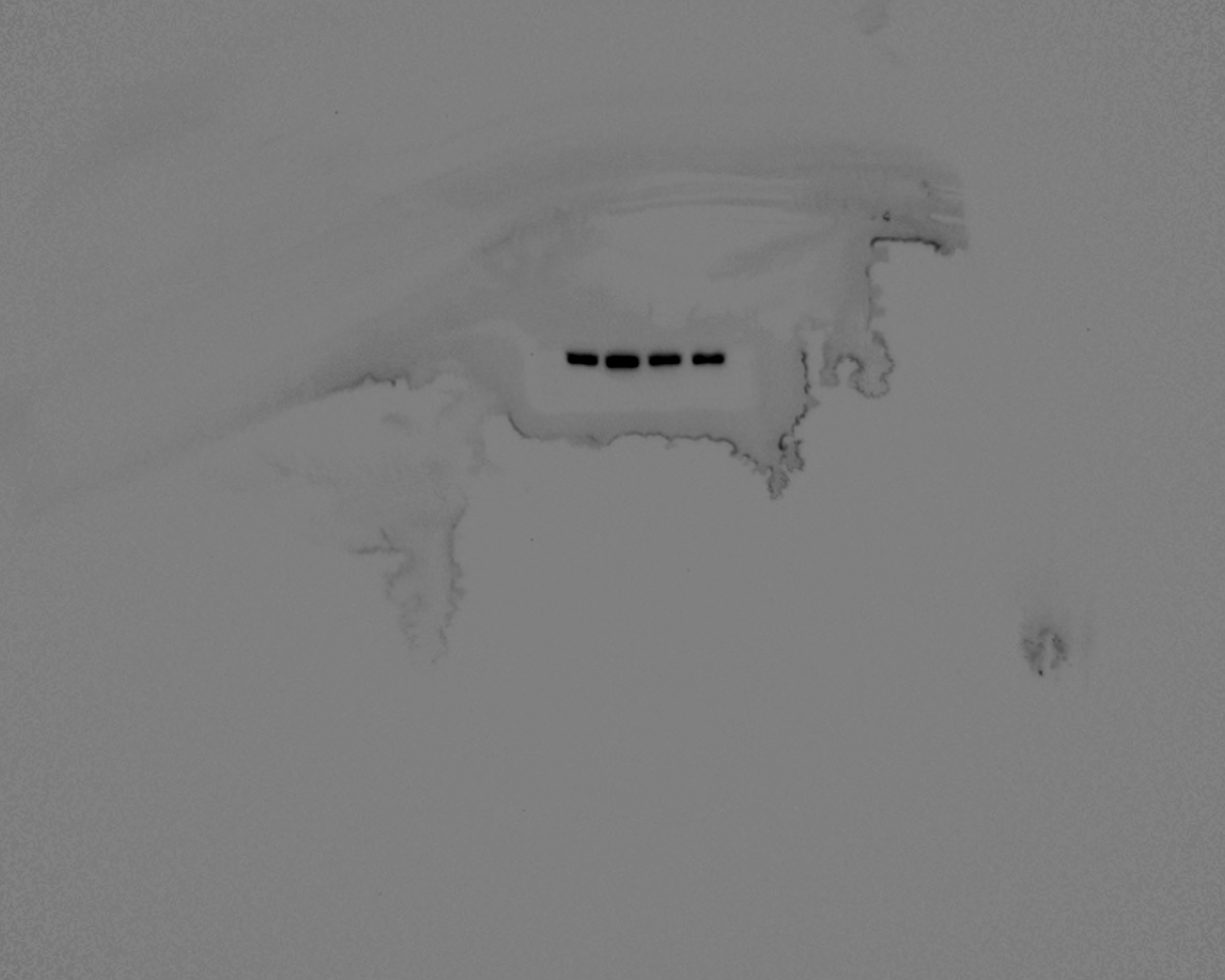

Supplement: Supplementary file 7 — Additional file 7. [file 12964_2024_1475_MOESM7_ESM.zip › Additional file 2/Figure 5J/KYSE-150/ip oct4.tif]

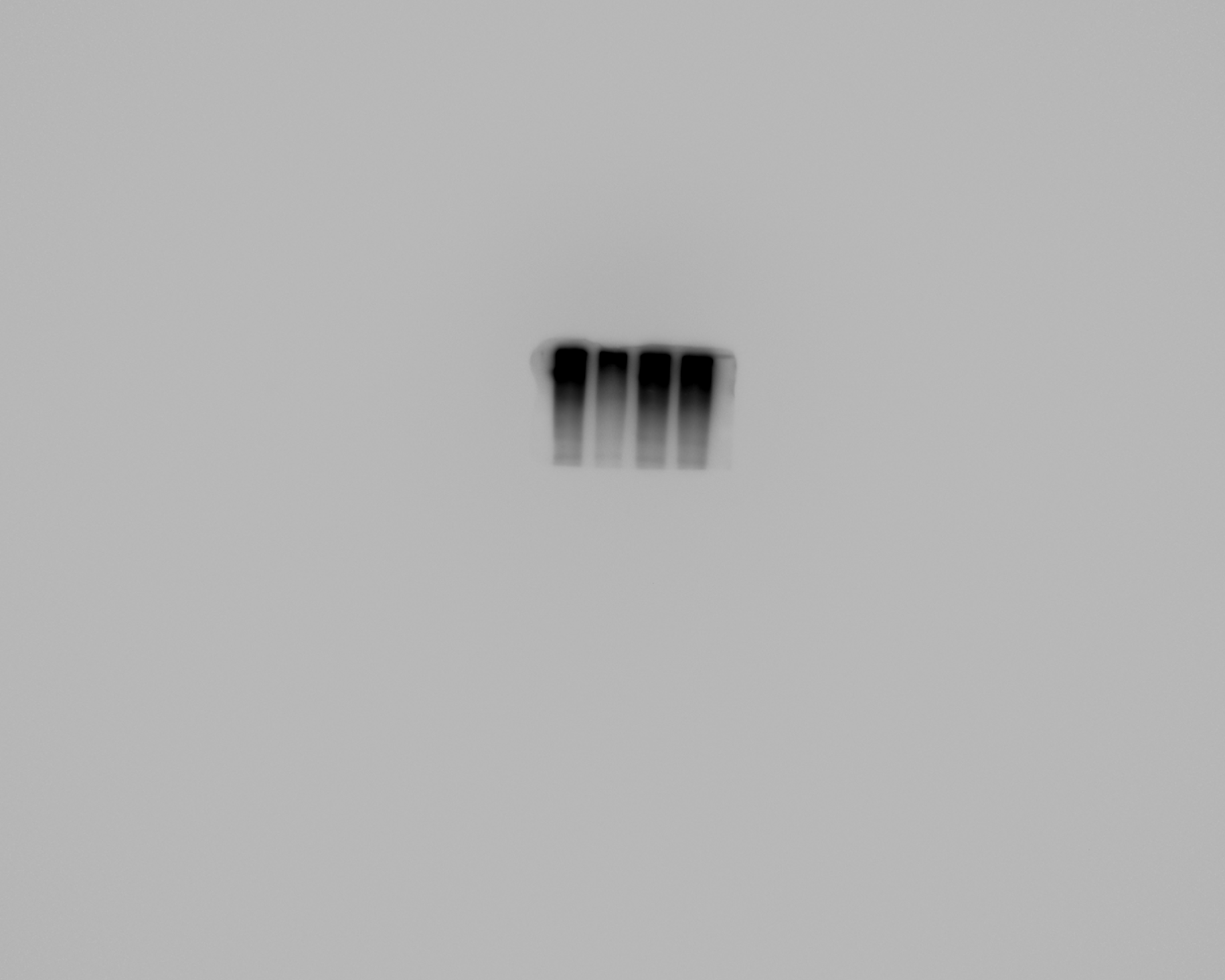

Supplement: Supplementary file 7 — Additional file 7. [file 12964_2024_1475_MOESM7_ESM.zip › Additional file 2/Figure 5J/KYSE-150/ip ubiquitin.tif]

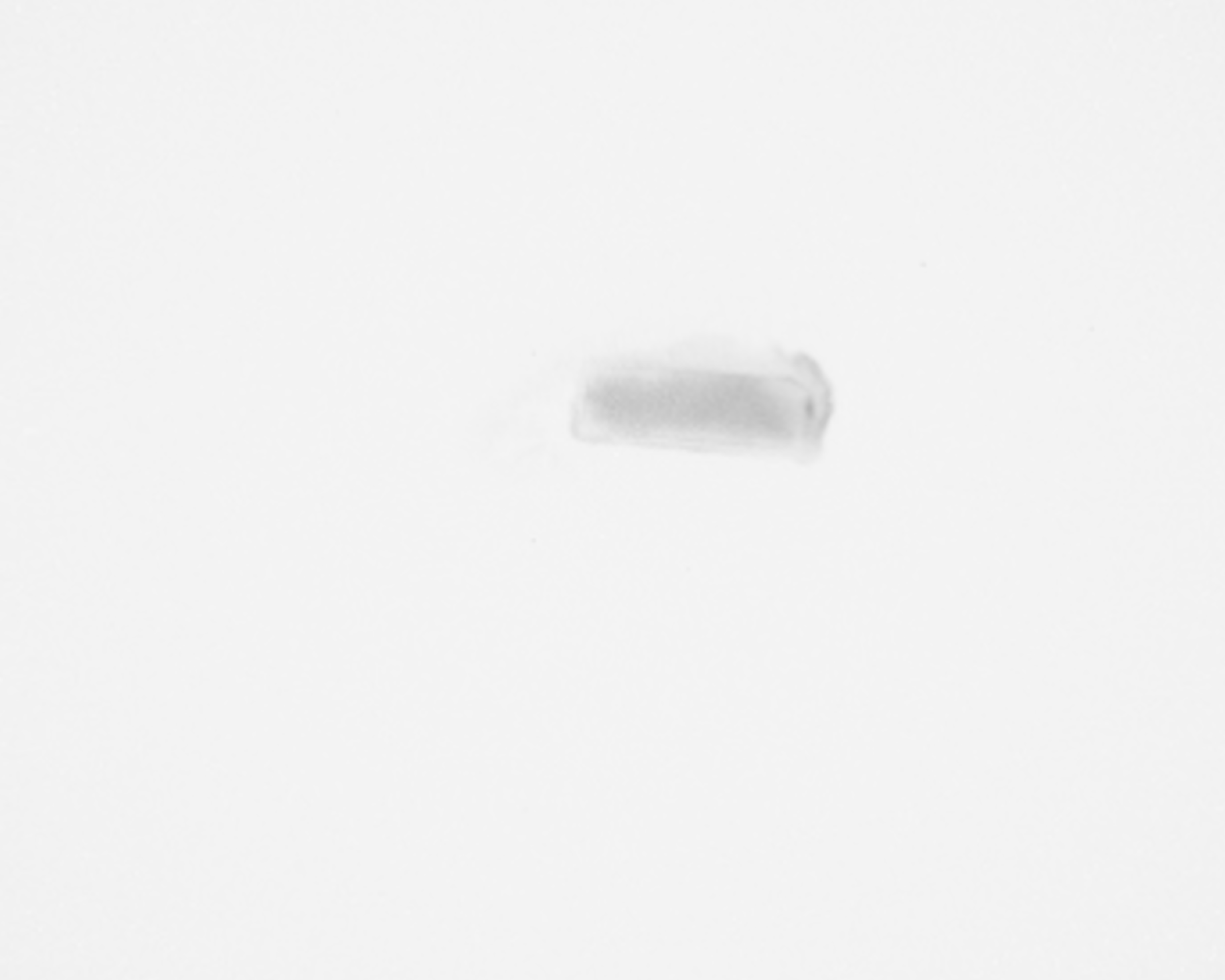

Supplement: Supplementary file 7 — Additional file 7. [file 12964_2024_1475_MOESM7_ESM.zip › Additional file 2/Figure 5J/KYSE-30/IgG oct4.tif]

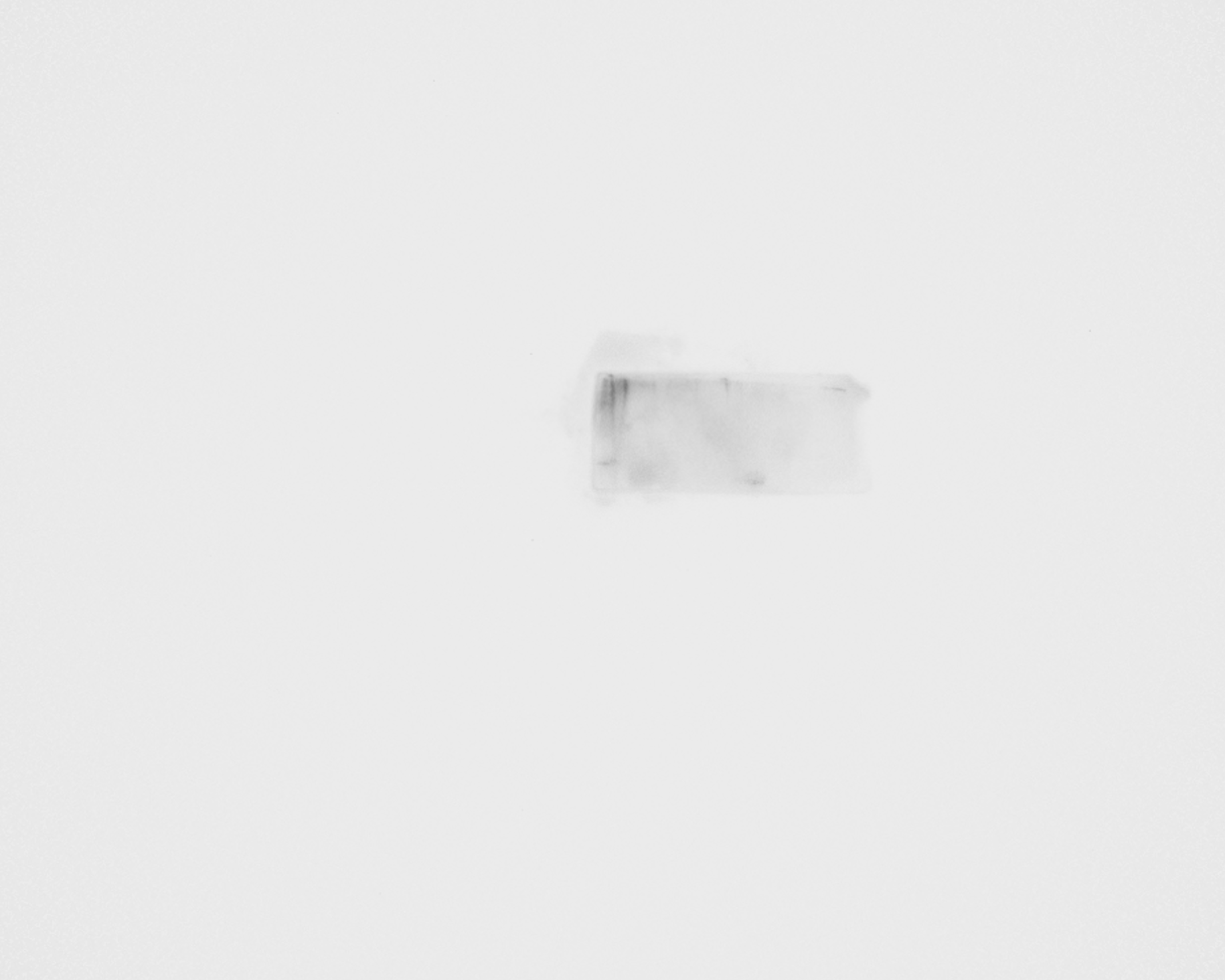

Supplement: Supplementary file 7 — Additional file 7. [file 12964_2024_1475_MOESM7_ESM.zip › Additional file 2/Figure 5J/KYSE-30/IgG ubiquitin.tif]

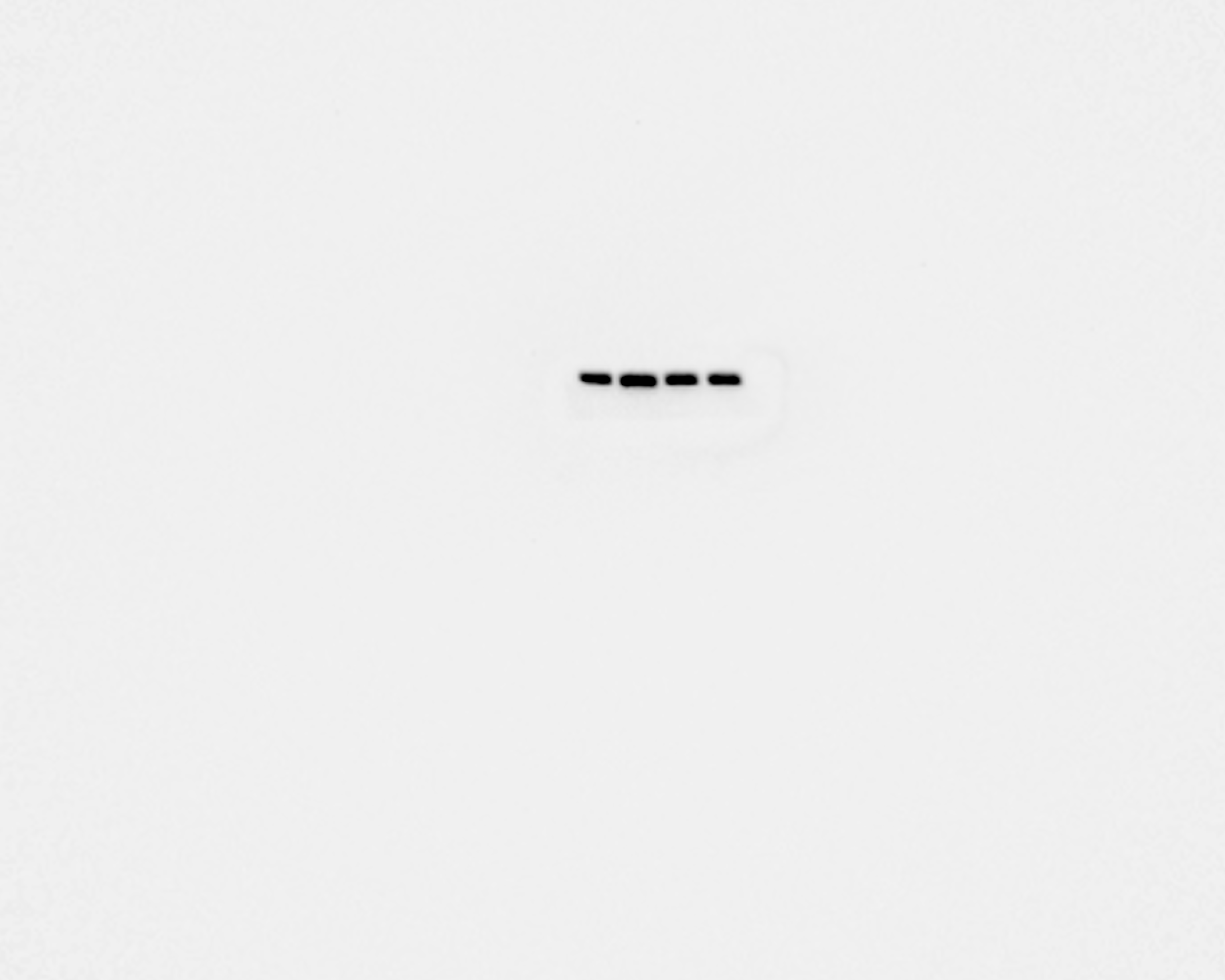

Supplement: Supplementary file 7 — Additional file 7. [file 12964_2024_1475_MOESM7_ESM.zip › Additional file 2/Figure 5J/KYSE-30/input oct4.tif]

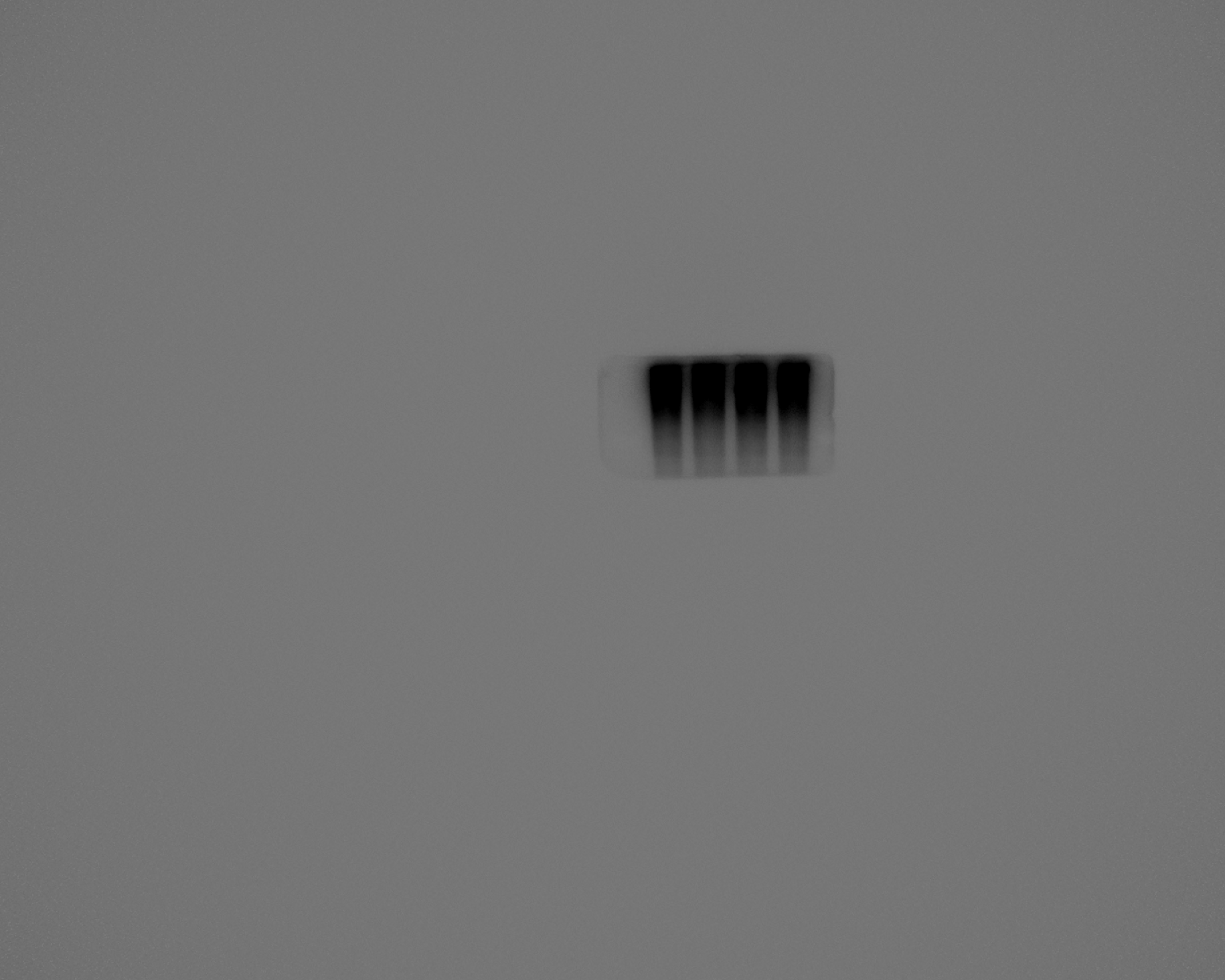

Supplement: Supplementary file 7 — Additional file 7. [file 12964_2024_1475_MOESM7_ESM.zip › Additional file 2/Figure 5J/KYSE-30/input ubiquitin.tif]

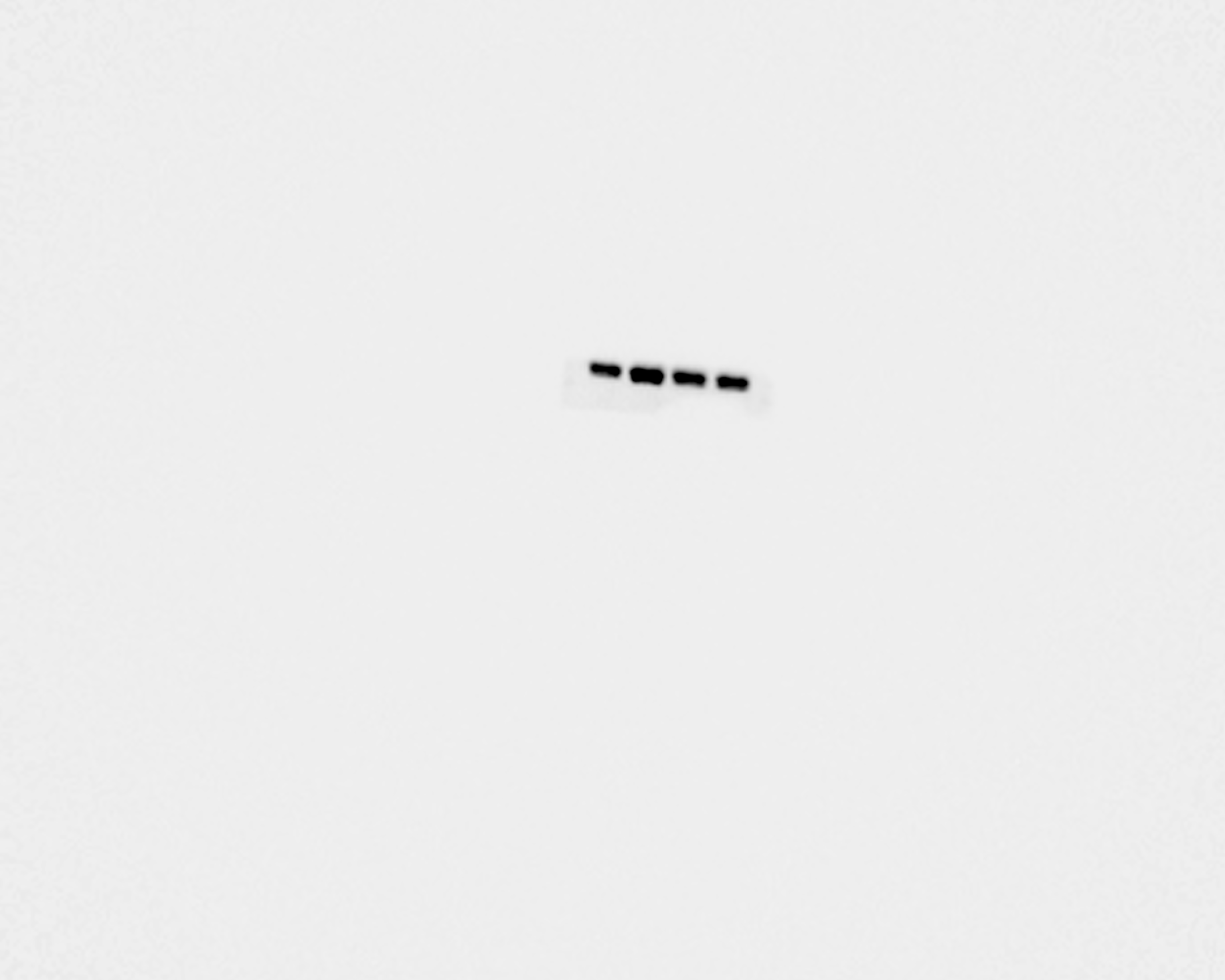

Supplement: Supplementary file 7 — Additional file 7. [file 12964_2024_1475_MOESM7_ESM.zip › Additional file 2/Figure 5J/KYSE-30/ip oct4.tif]

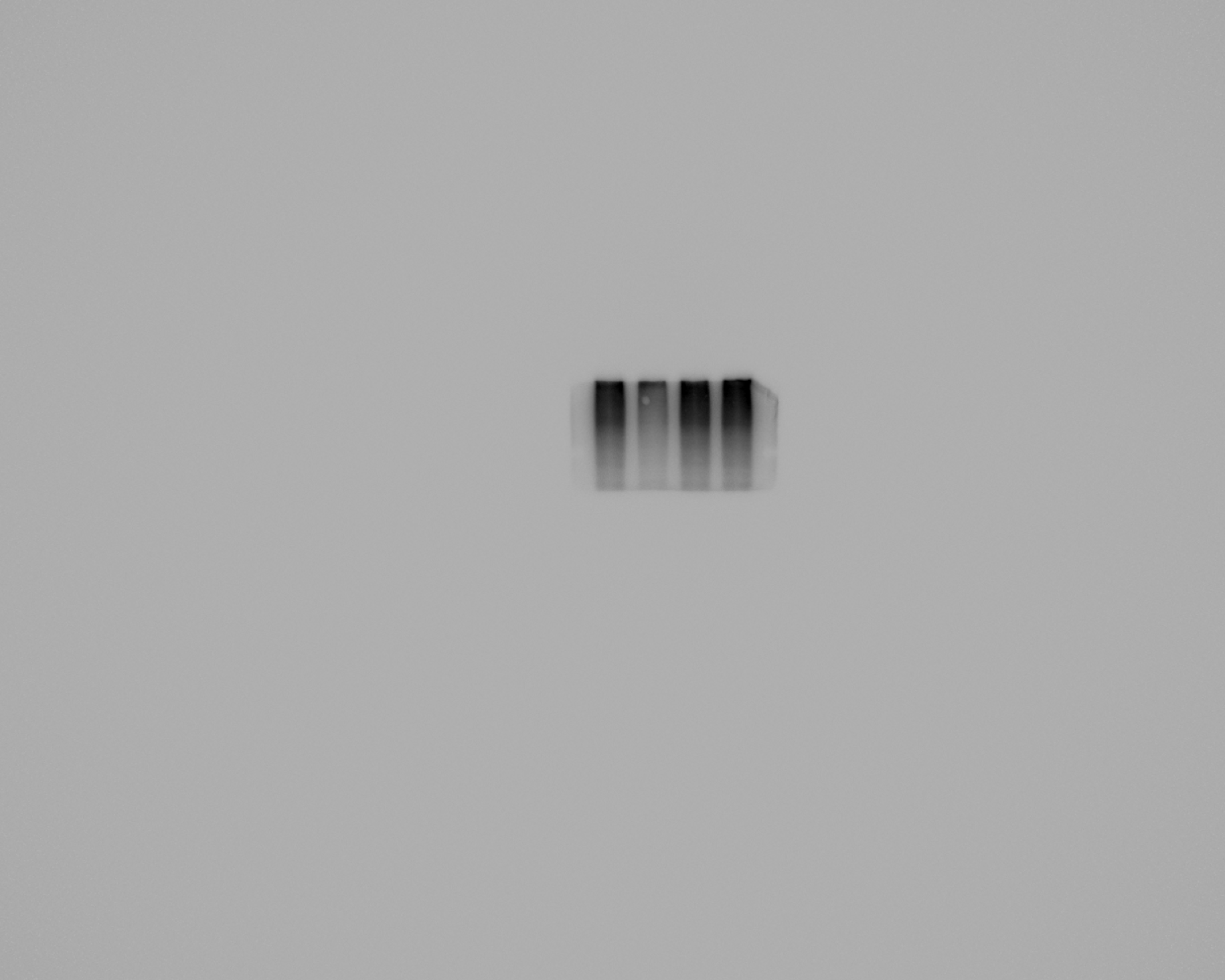

Supplement: Supplementary file 7 — Additional file 7. [file 12964_2024_1475_MOESM7_ESM.zip › Additional file 2/Figure 5J/KYSE-30/ip ubiquitin.tif]

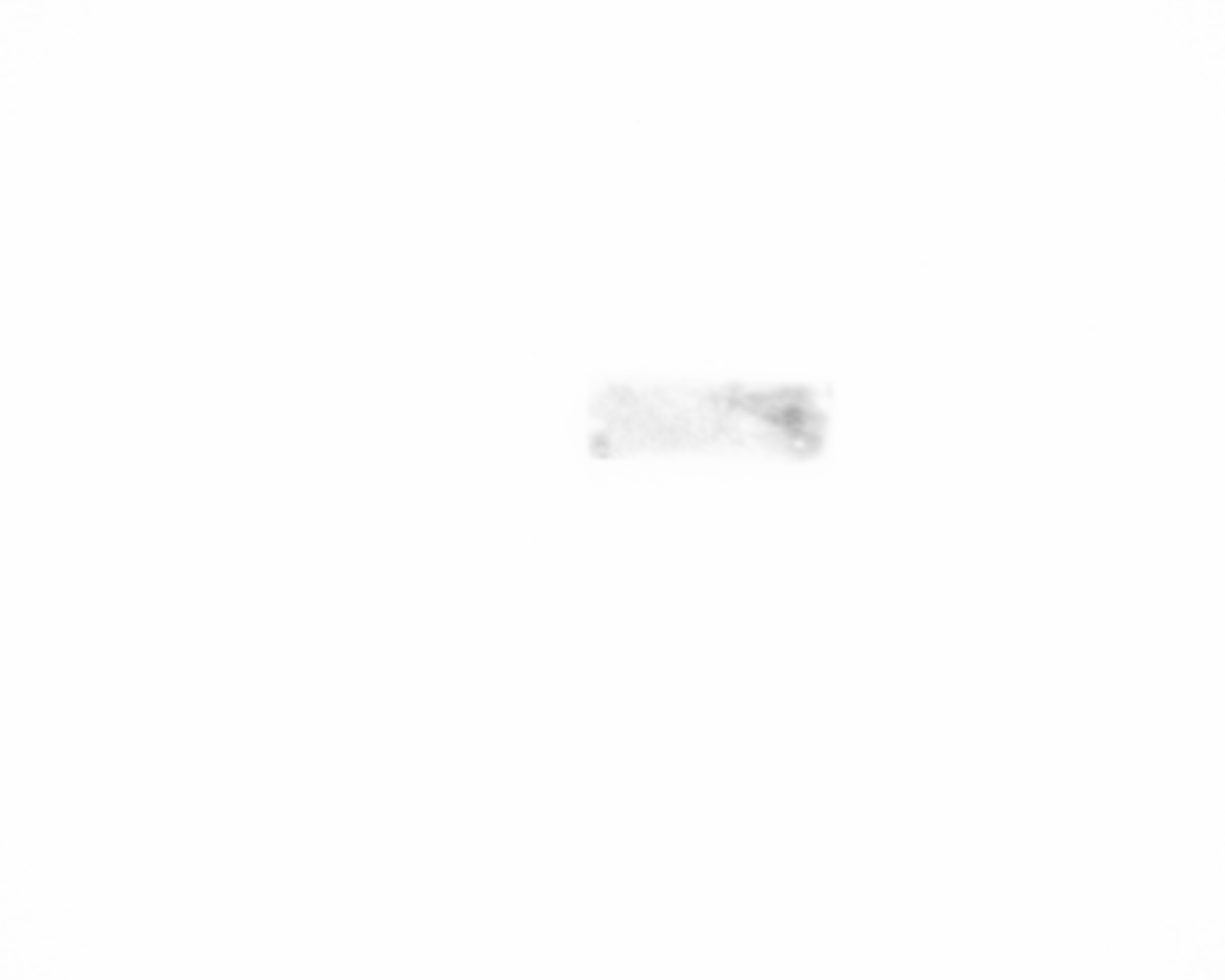

Supplement: Supplementary file 7 — Additional file 7. [file 12964_2024_1475_MOESM7_ESM.zip › Additional file 2/Figure 5K/KYSE-150/IgG oct4.tif]

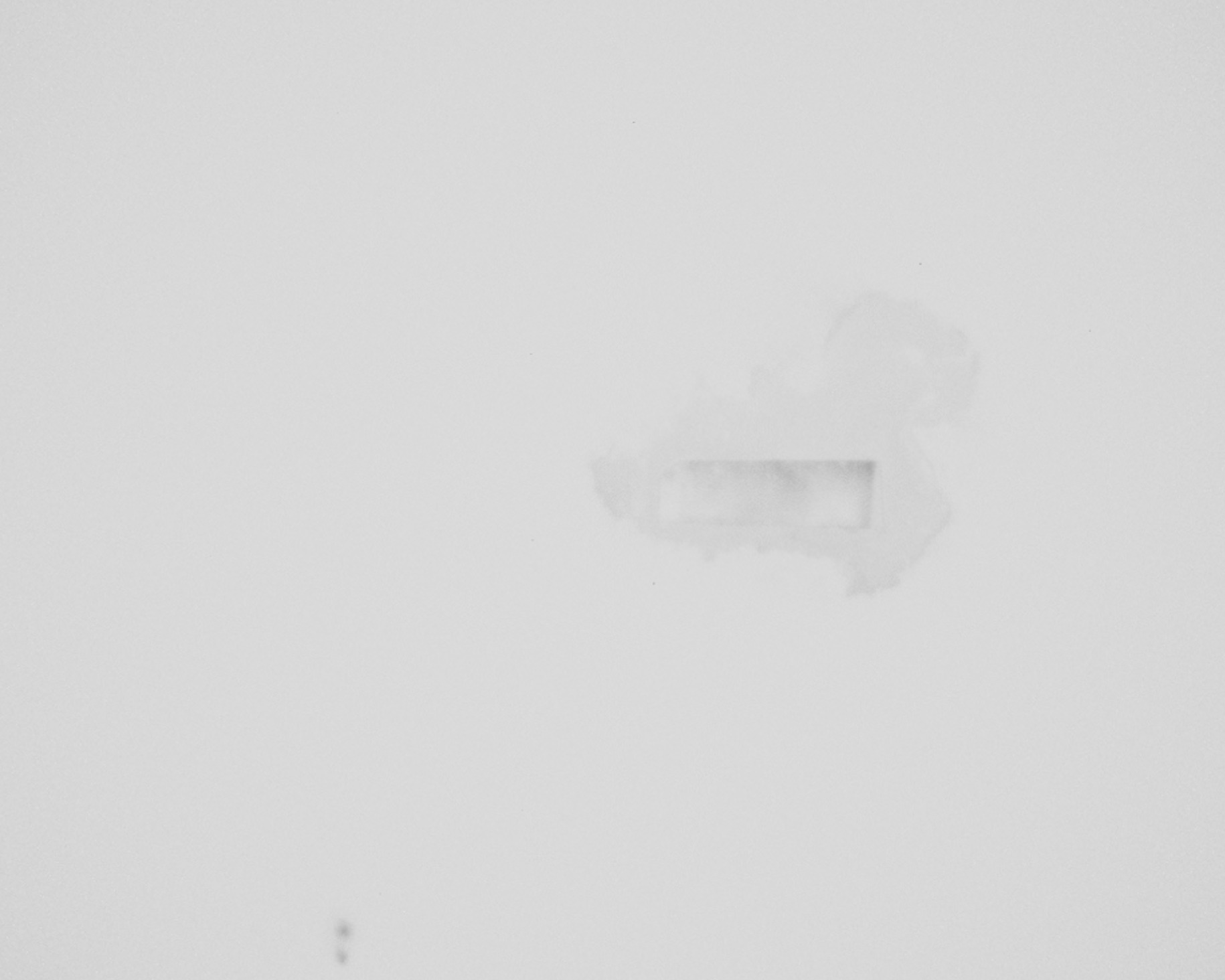

Supplement: Supplementary file 7 — Additional file 7. [file 12964_2024_1475_MOESM7_ESM.zip › Additional file 2/Figure 5K/KYSE-150/IgG wwp2.tif]

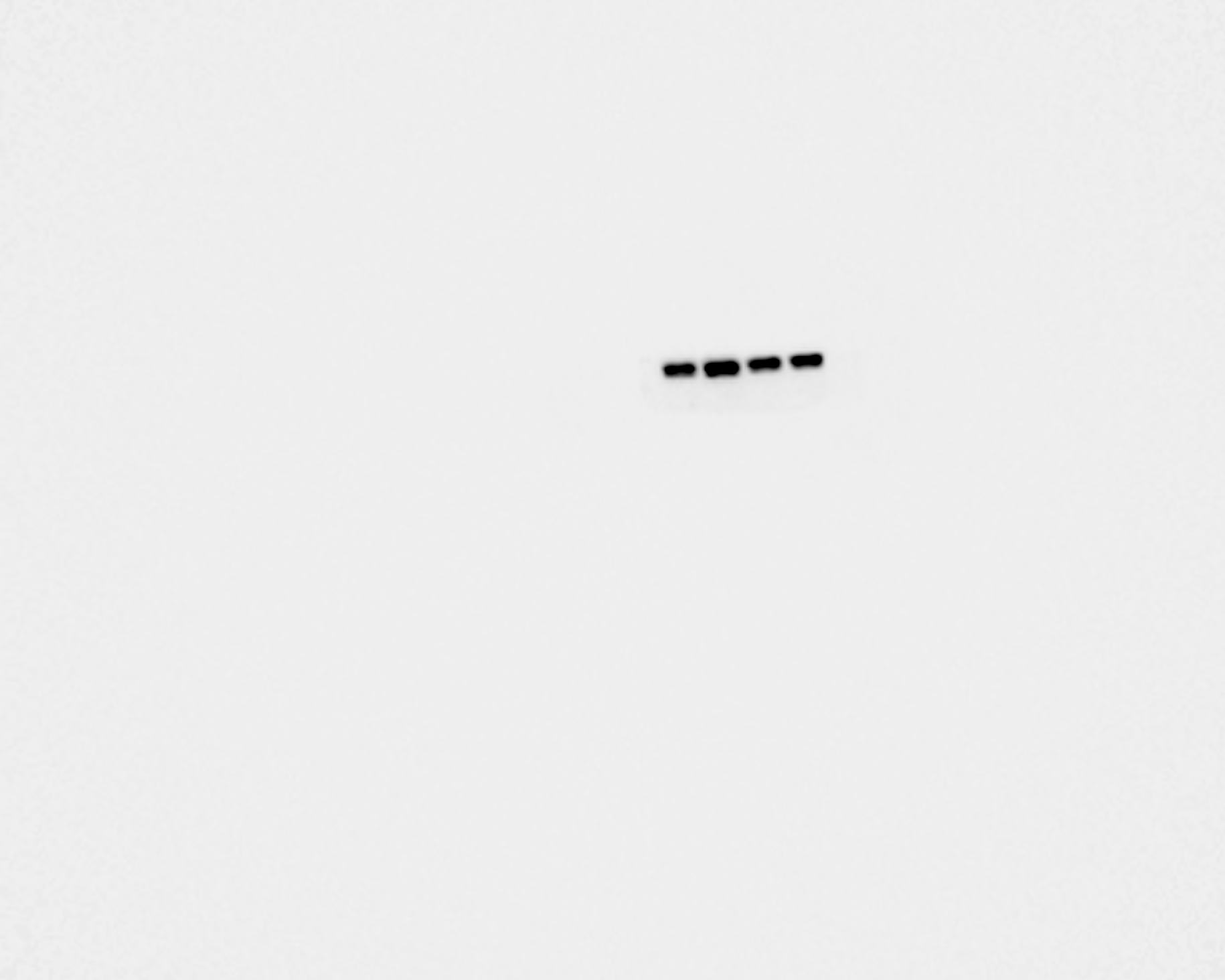

Supplement: Supplementary file 7 — Additional file 7. [file 12964_2024_1475_MOESM7_ESM.zip › Additional file 2/Figure 5K/KYSE-150/input oct4.tif]

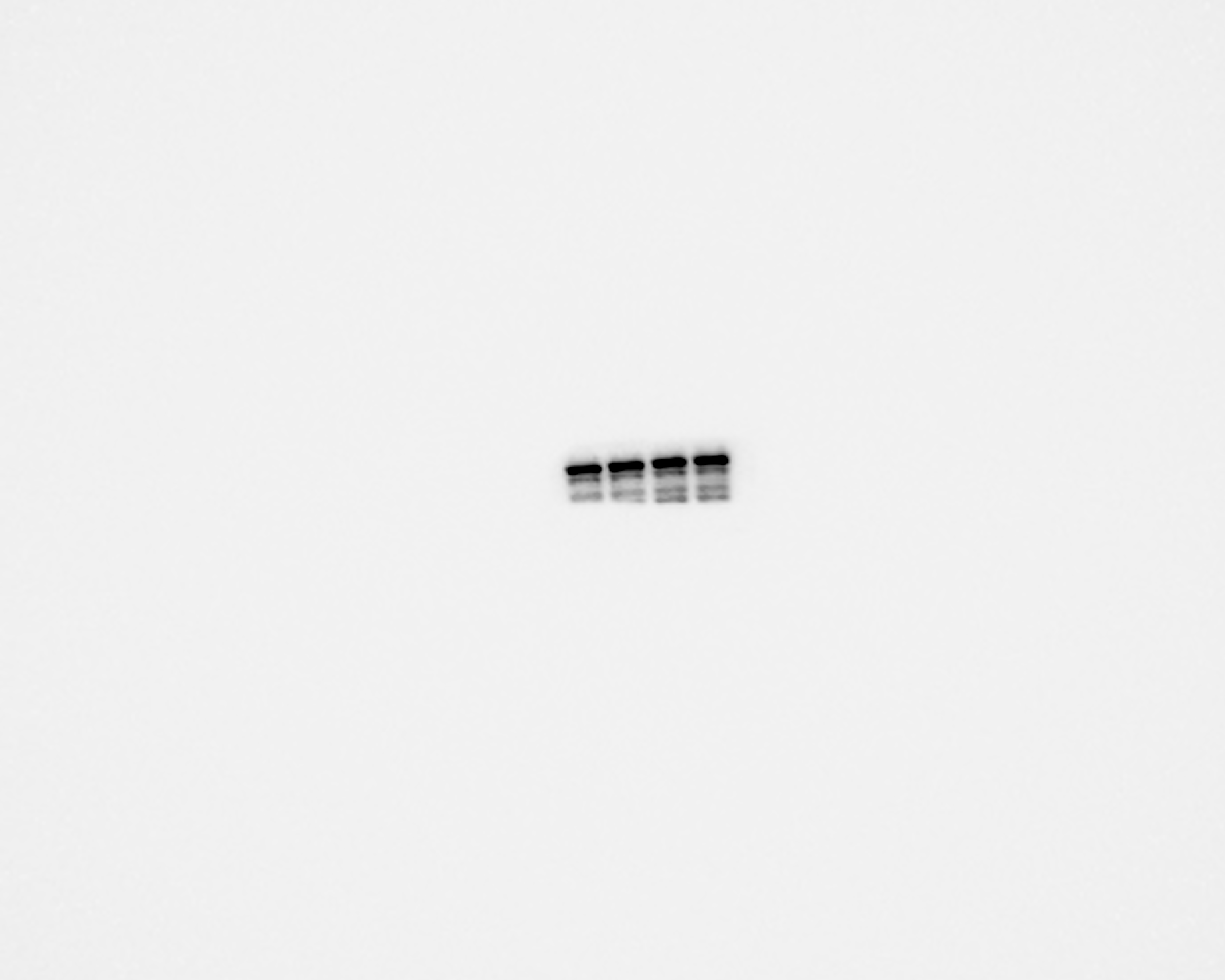

Supplement: Supplementary file 7 — Additional file 7. [file 12964_2024_1475_MOESM7_ESM.zip › Additional file 2/Figure 5K/KYSE-150/input wwp2.tif]

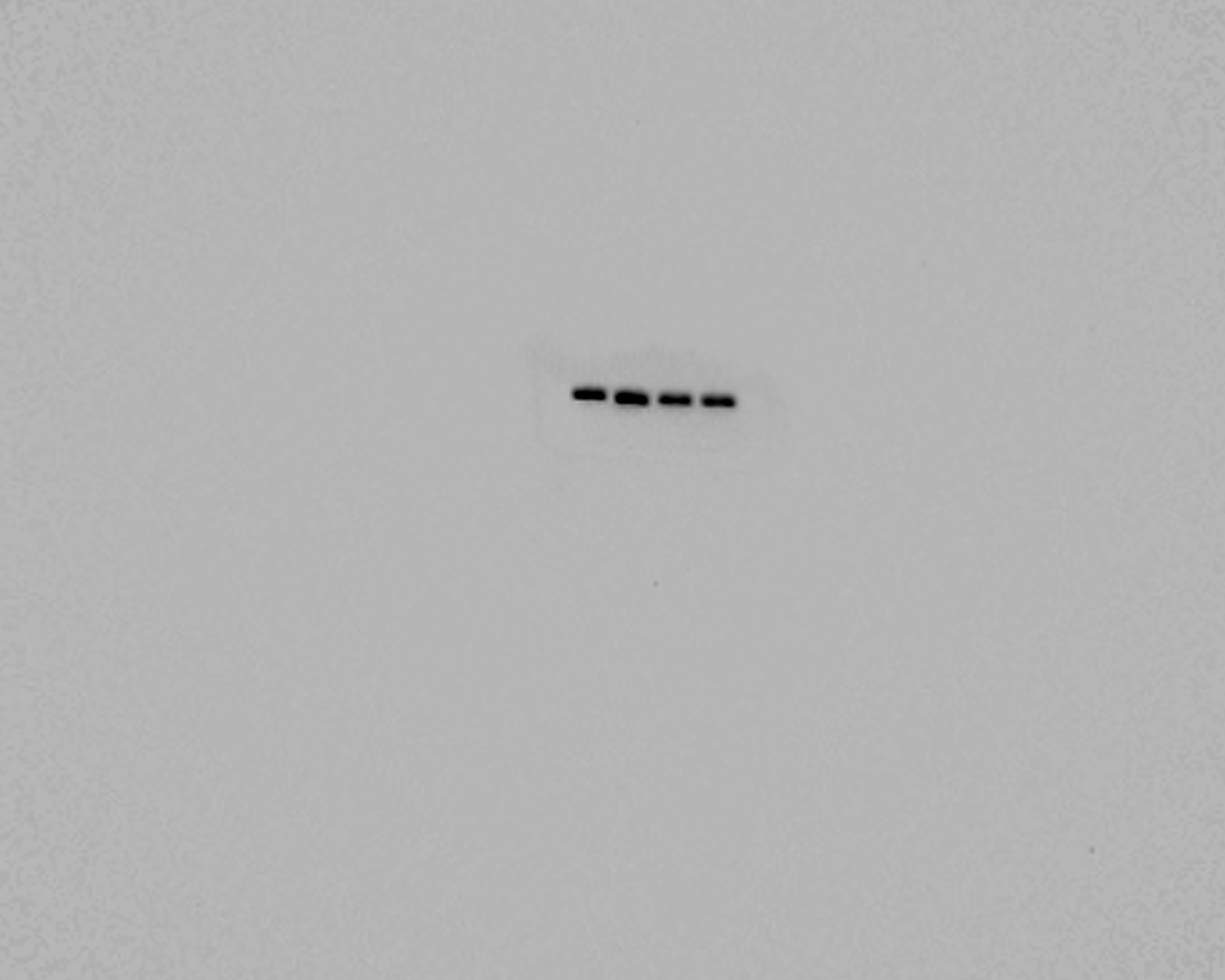

Supplement: Supplementary file 7 — Additional file 7. [file 12964_2024_1475_MOESM7_ESM.zip › Additional file 2/Figure 5K/KYSE-150/ip oct4.tif]

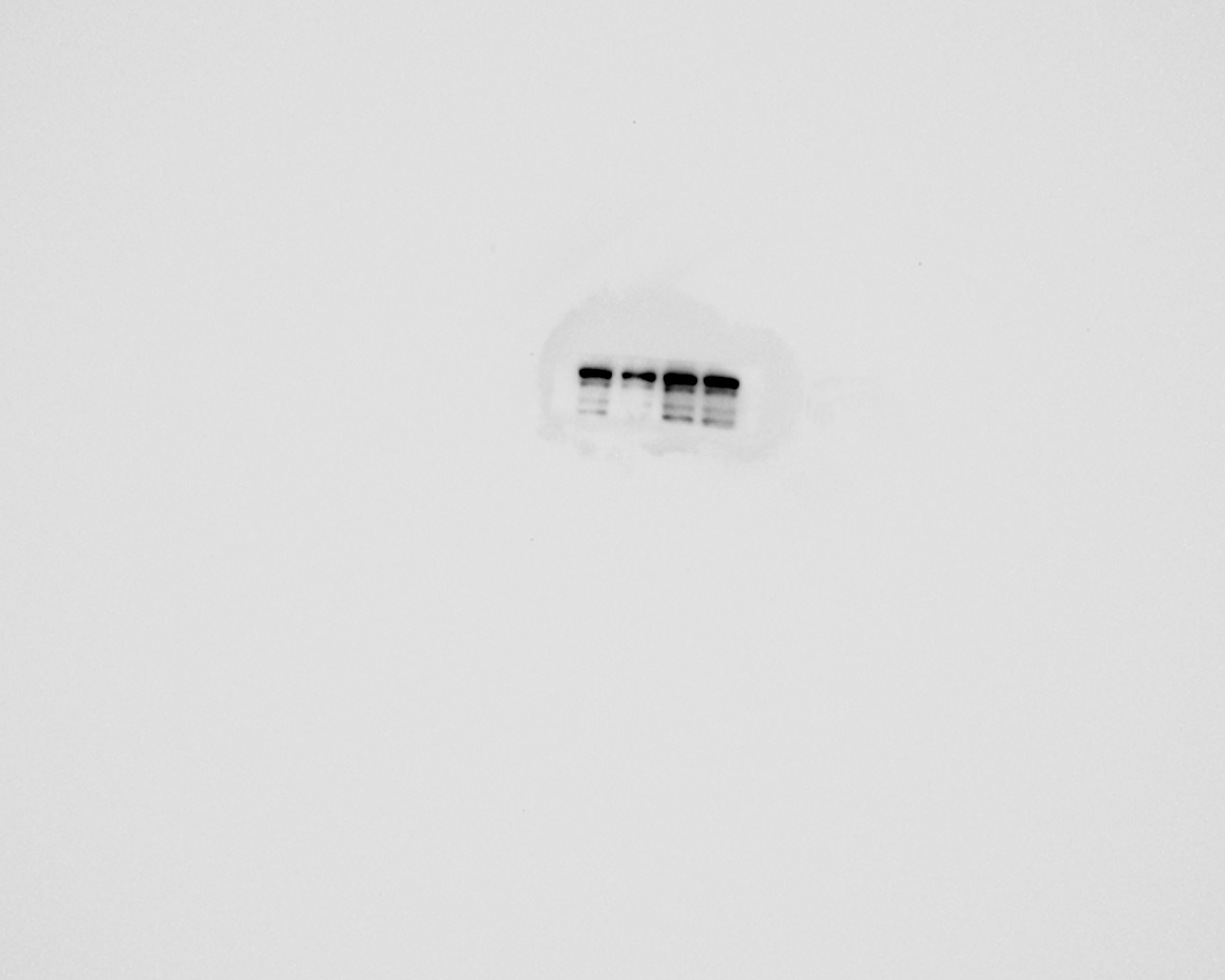

Supplement: Supplementary file 7 — Additional file 7. [file 12964_2024_1475_MOESM7_ESM.zip › Additional file 2/Figure 5K/KYSE-150/ip wwp2.tif]

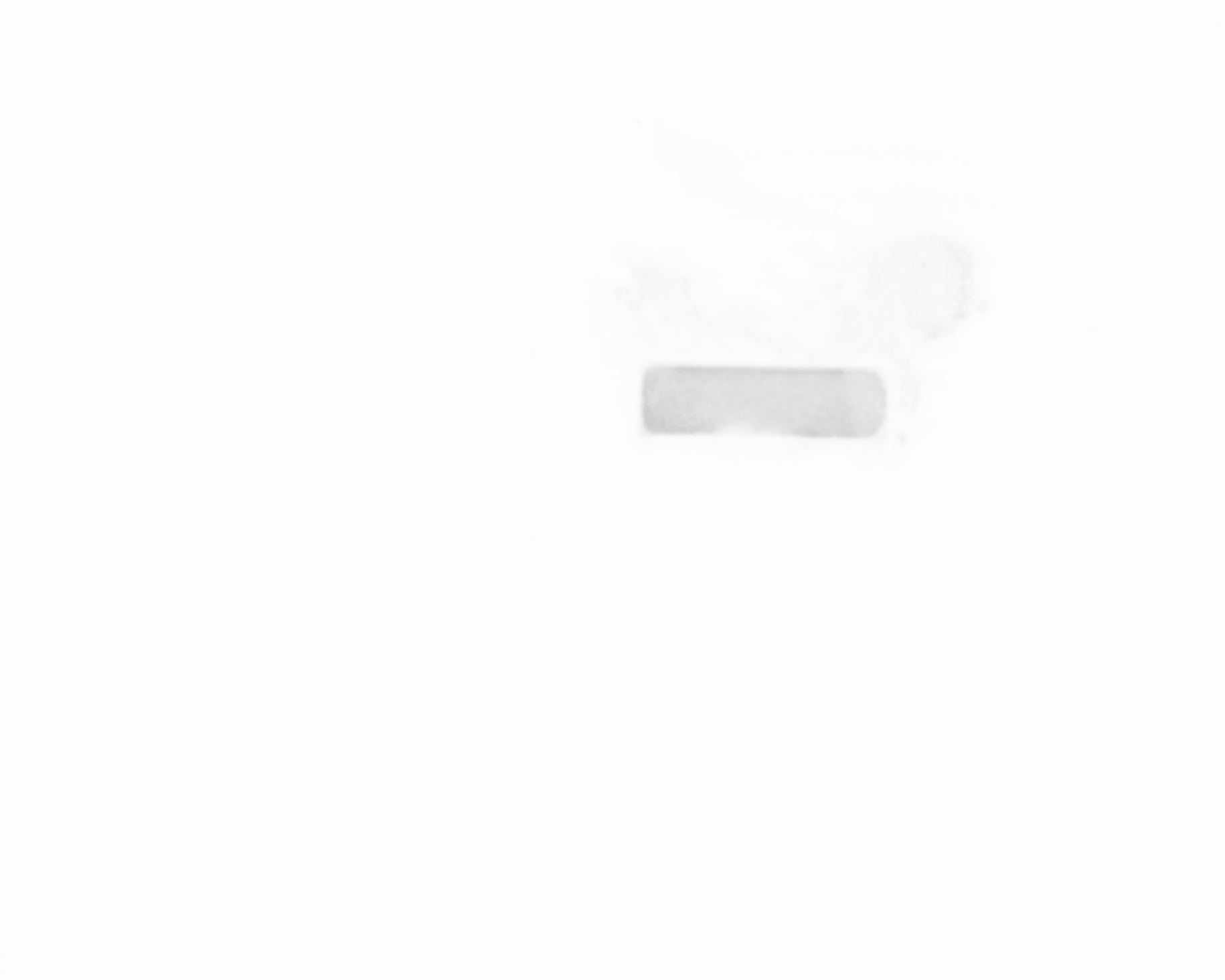

Supplement: Supplementary file 7 — Additional file 7. [file 12964_2024_1475_MOESM7_ESM.zip › Additional file 2/Figure 5K/KYSE-30/IgG oct4.tif]

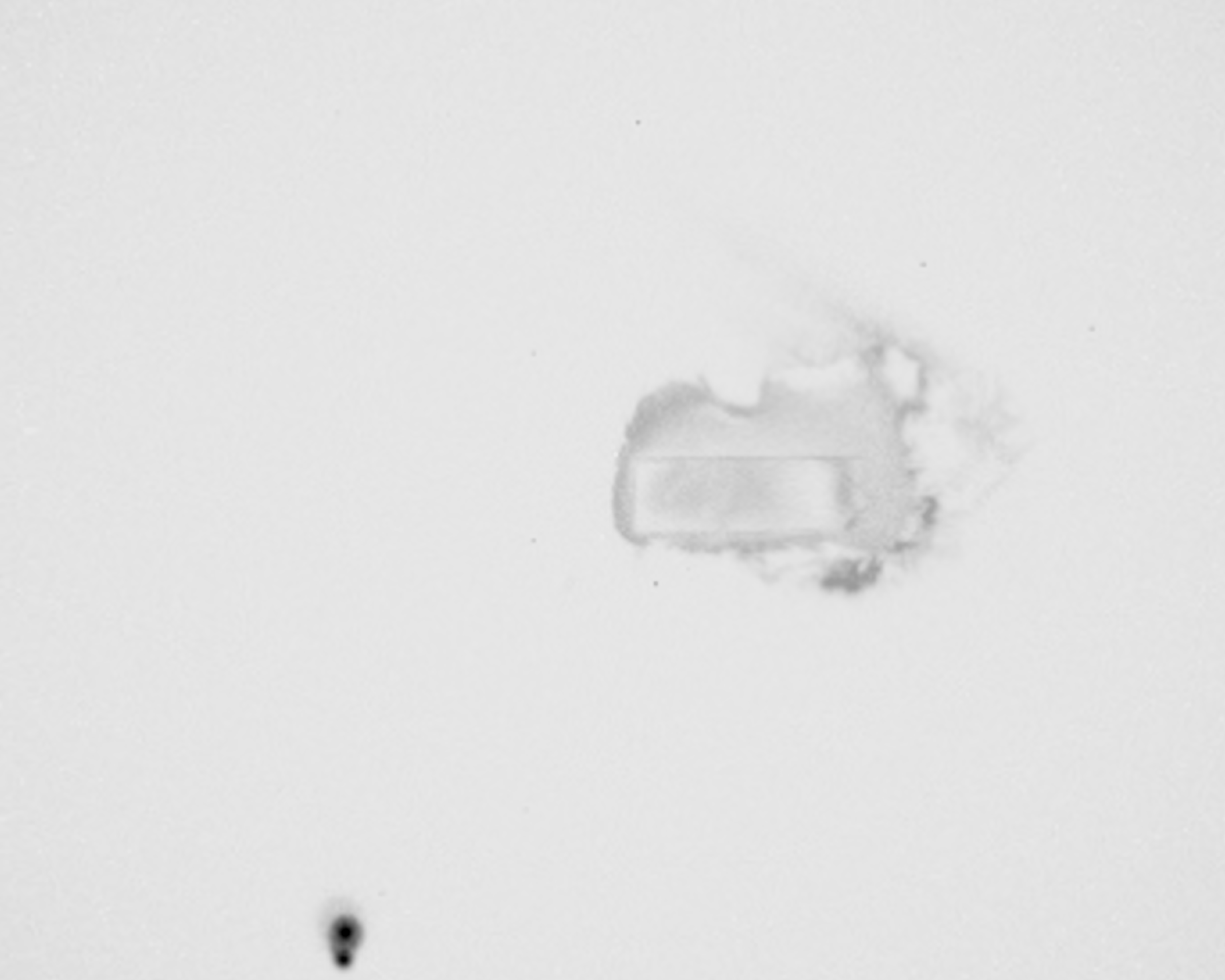

Supplement: Supplementary file 7 — Additional file 7. [file 12964_2024_1475_MOESM7_ESM.zip › Additional file 2/Figure 5K/KYSE-30/IgG wwp2.tif]

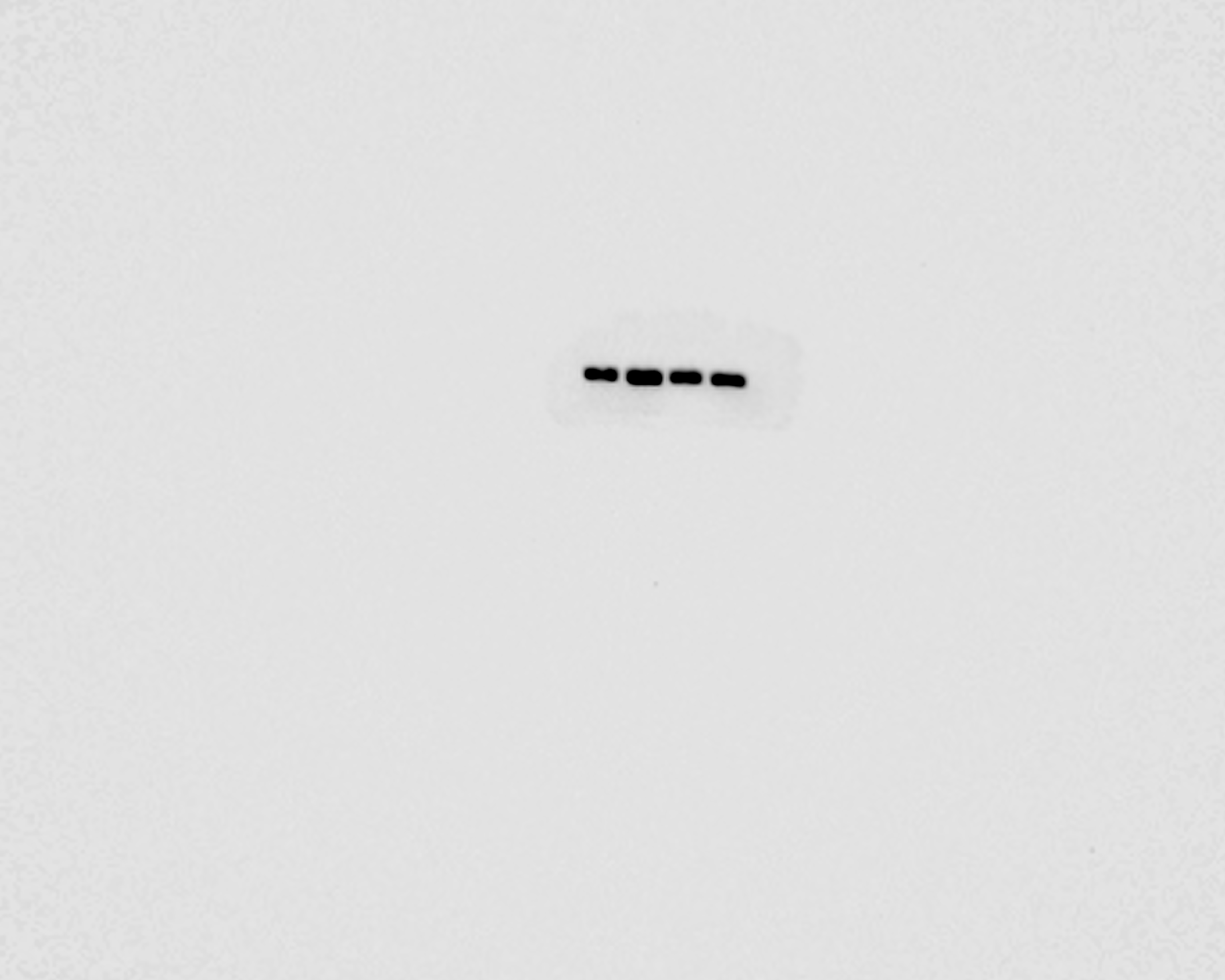

Supplement: Supplementary file 7 — Additional file 7. [file 12964_2024_1475_MOESM7_ESM.zip › Additional file 2/Figure 5K/KYSE-30/input oct4.tif]

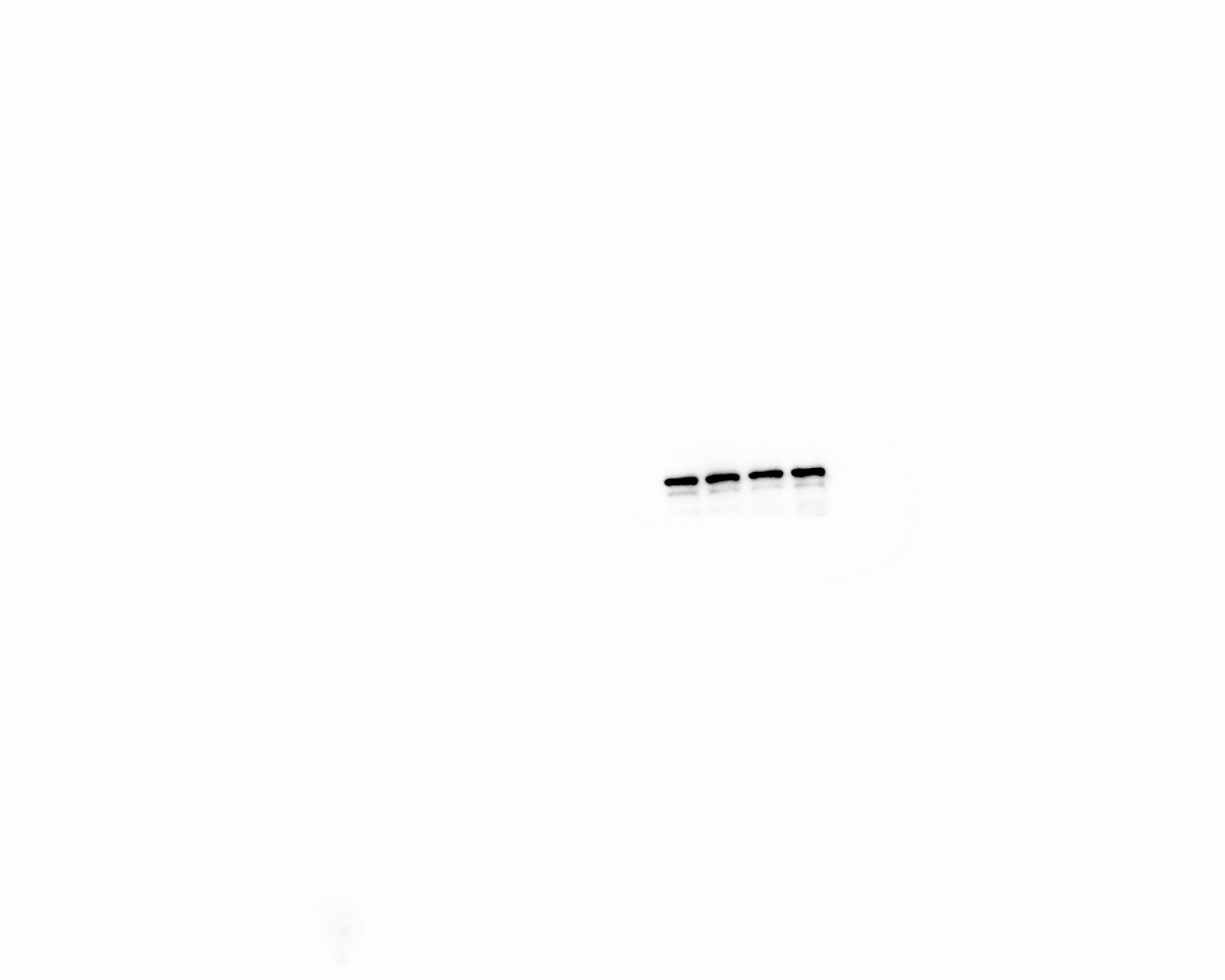

Supplement: Supplementary file 7 — Additional file 7. [file 12964_2024_1475_MOESM7_ESM.zip › Additional file 2/Figure 5K/KYSE-30/input wwp2.tif]

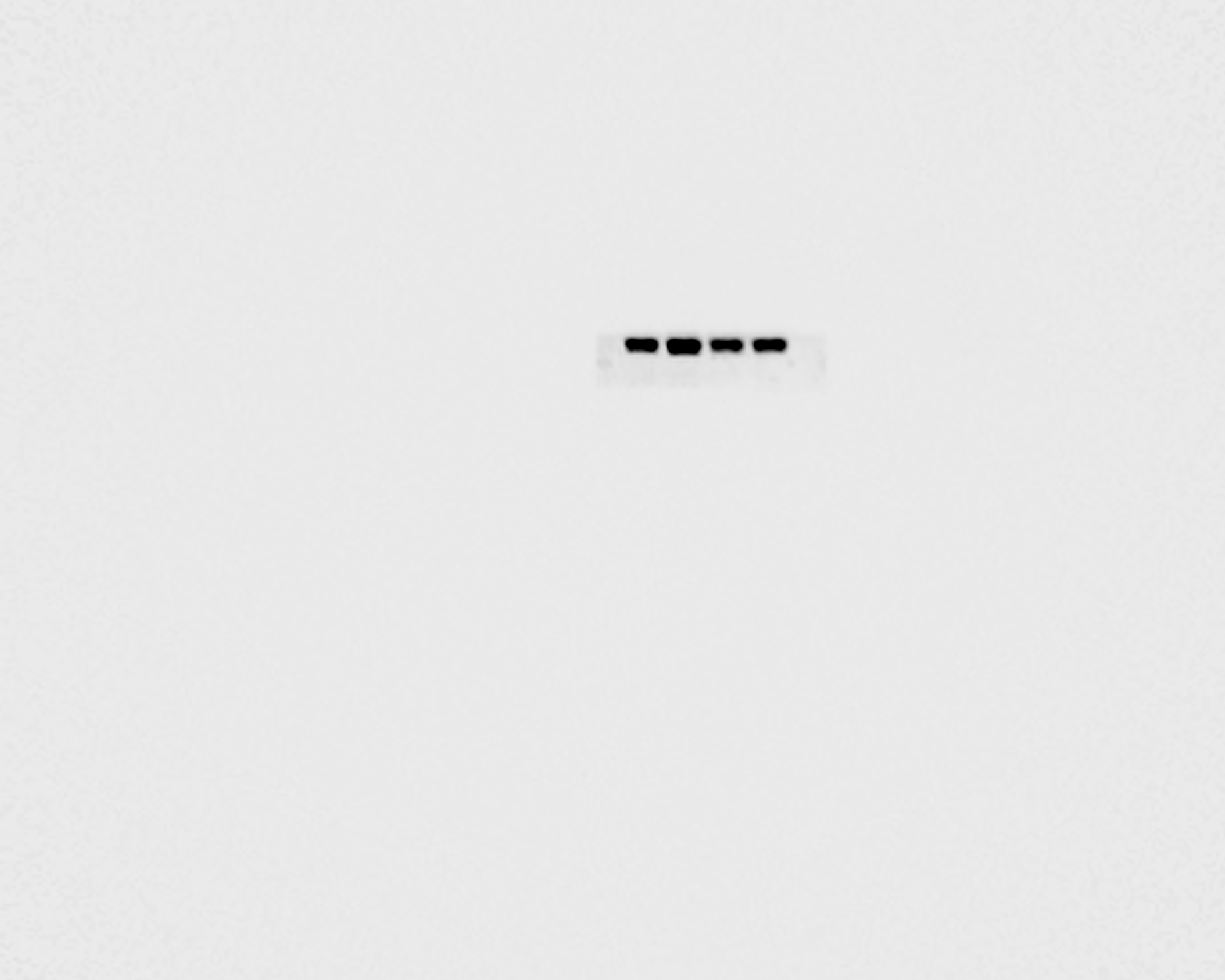

Supplement: Supplementary file 7 — Additional file 7. [file 12964_2024_1475_MOESM7_ESM.zip › Additional file 2/Figure 5K/KYSE-30/ip oct4.tif]

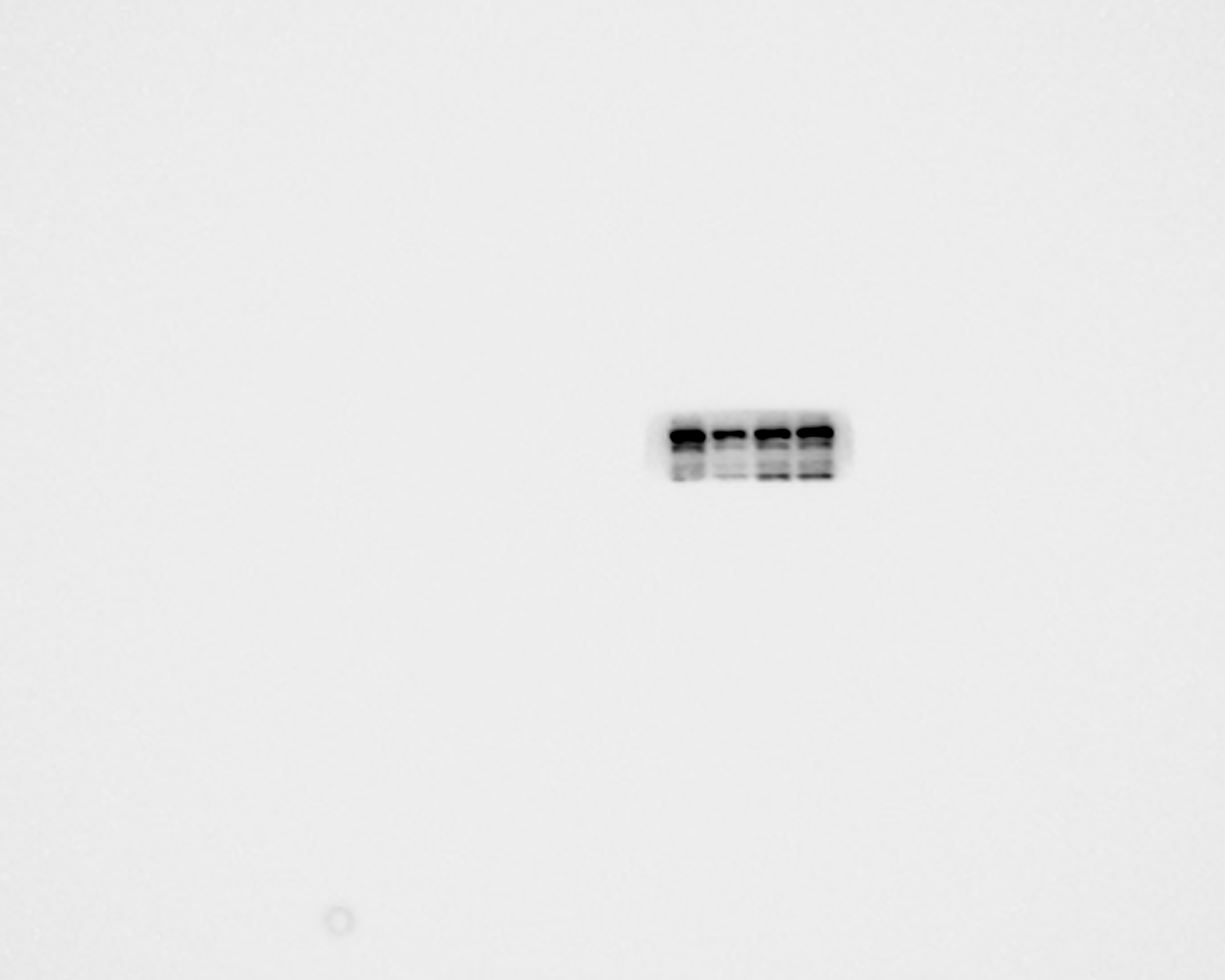

Supplement: Supplementary file 7 — Additional file 7. [file 12964_2024_1475_MOESM7_ESM.zip › Additional file 2/Figure 5K/KYSE-30/ip wwp2.tif]

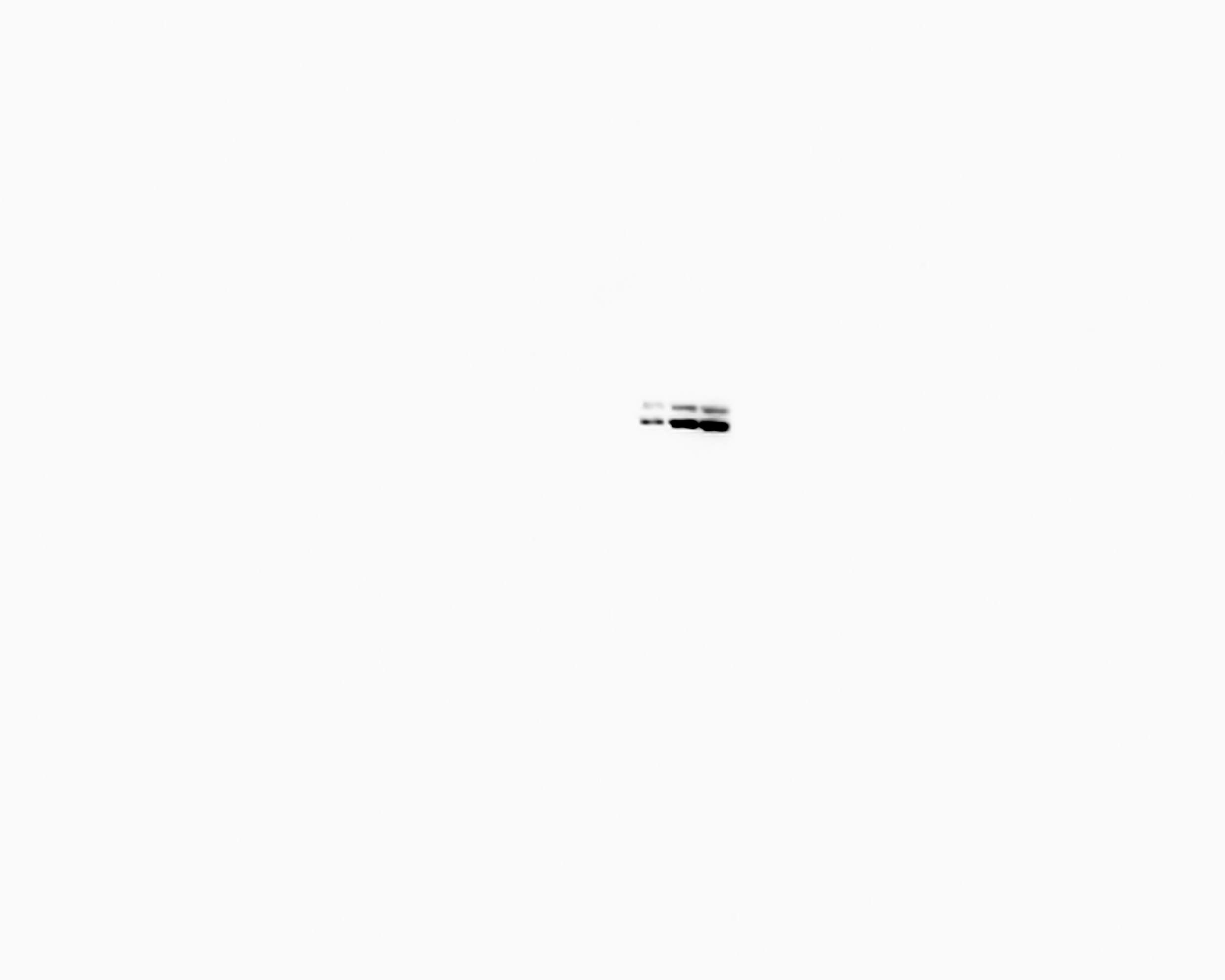

Supplement: Supplementary file 7 — Additional file 7. [file 12964_2024_1475_MOESM7_ESM.zip › Additional file 2/Figure 5L/KYSE-150/OCT4.tif]
