# Supplementary figures and images for: A positive feedback between PDIA3P1 and OCT4 promotes the cancer stem cell properties of esophageal squamous cell carcinoma (part 1 of 2)
Source: Cell Commun Signal. 2024 Jan 22;22:60. doi: 10.1186/s12964-024-01475-3 (PMC10801955; doi:10.1186/s12964-024-01475-3)

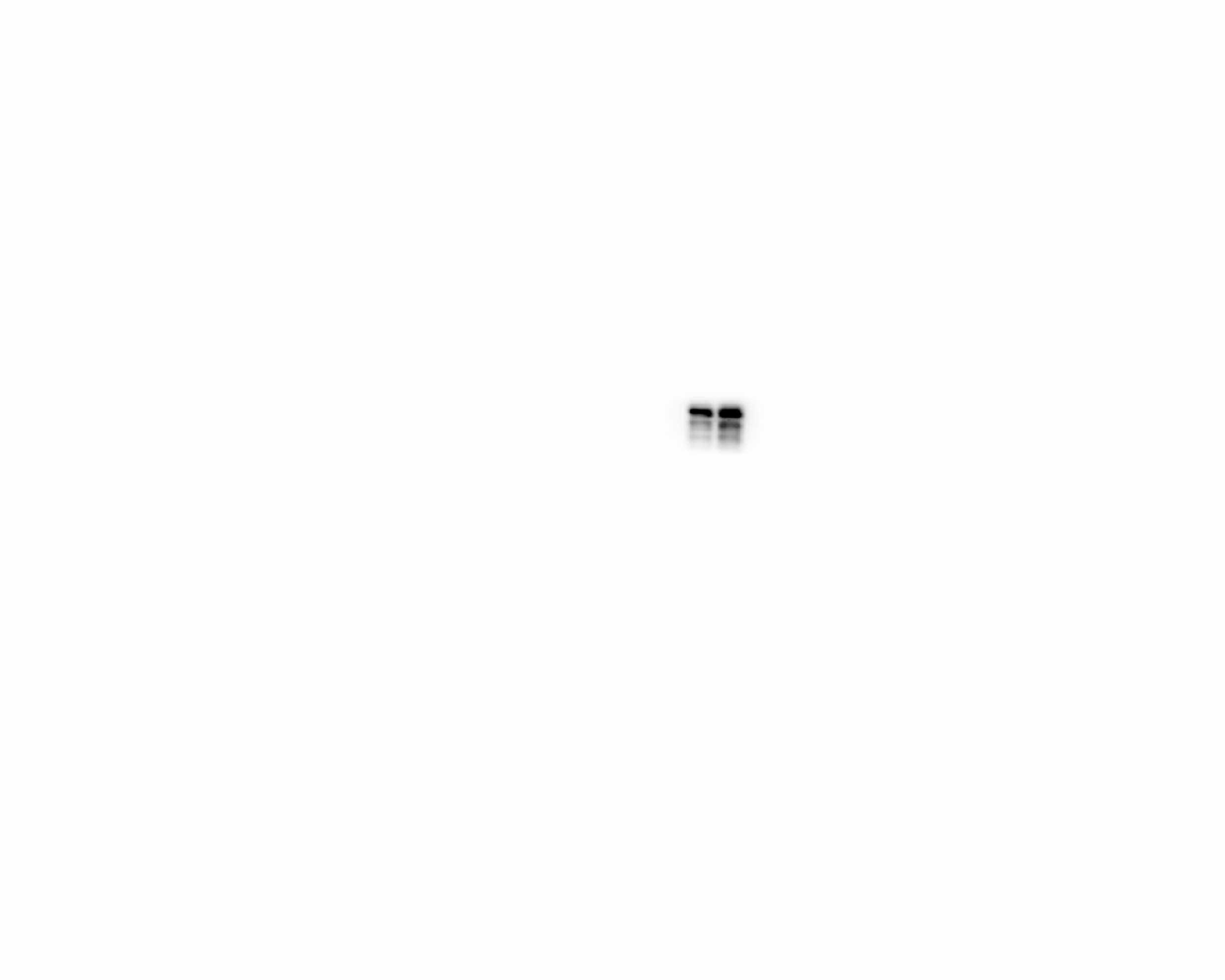

Supplement: Supplementary file 7 — Additional file 7. [file 12964_2024_1475_MOESM7_ESM.zip › Additional file 2/Figure 1N/Eca-109/E-cardherin.tif]

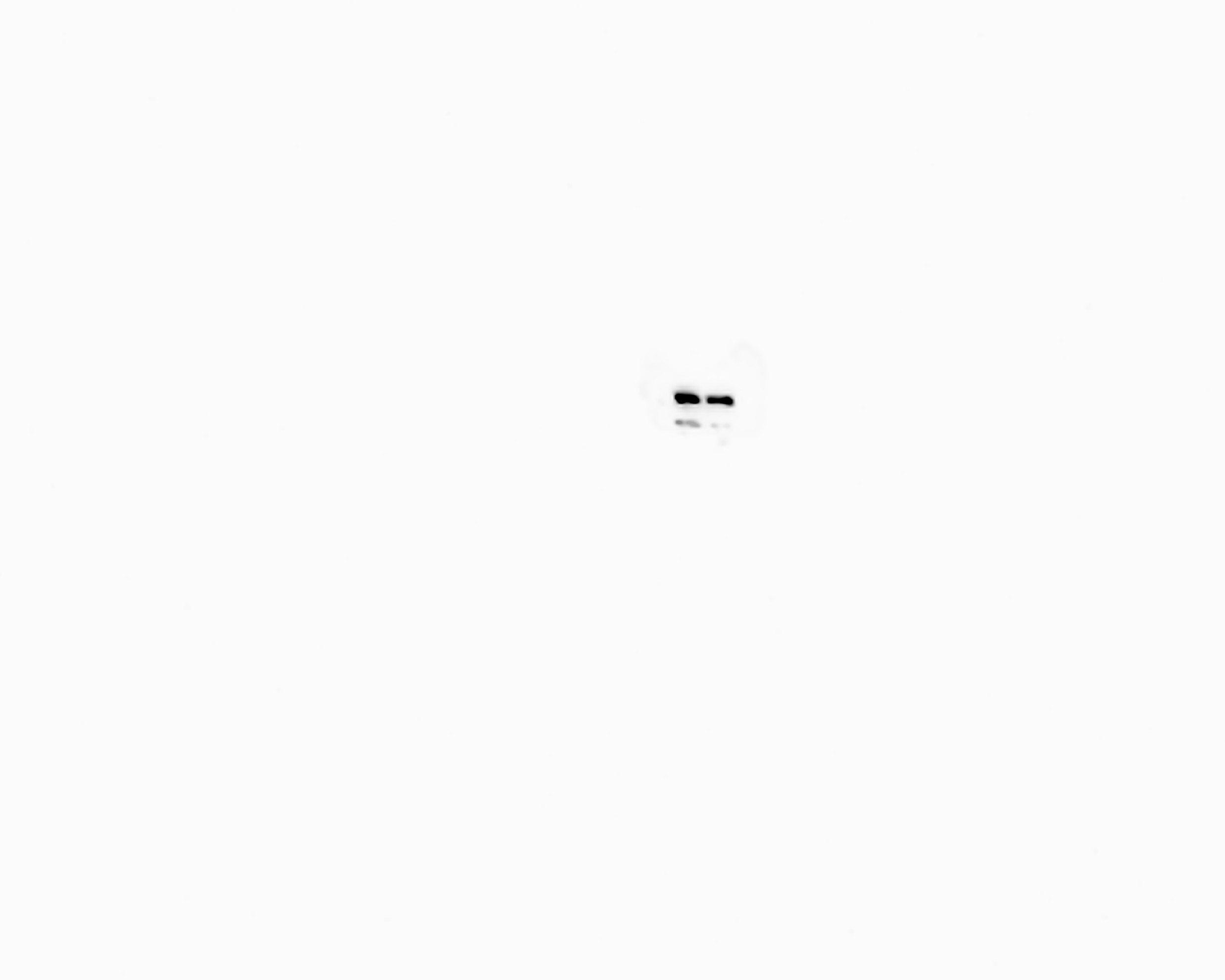

Supplement: Supplementary file 7 — Additional file 7. [file 12964_2024_1475_MOESM7_ESM.zip › Additional file 2/Figure 1N/Eca-109/N-cadherin.tif]

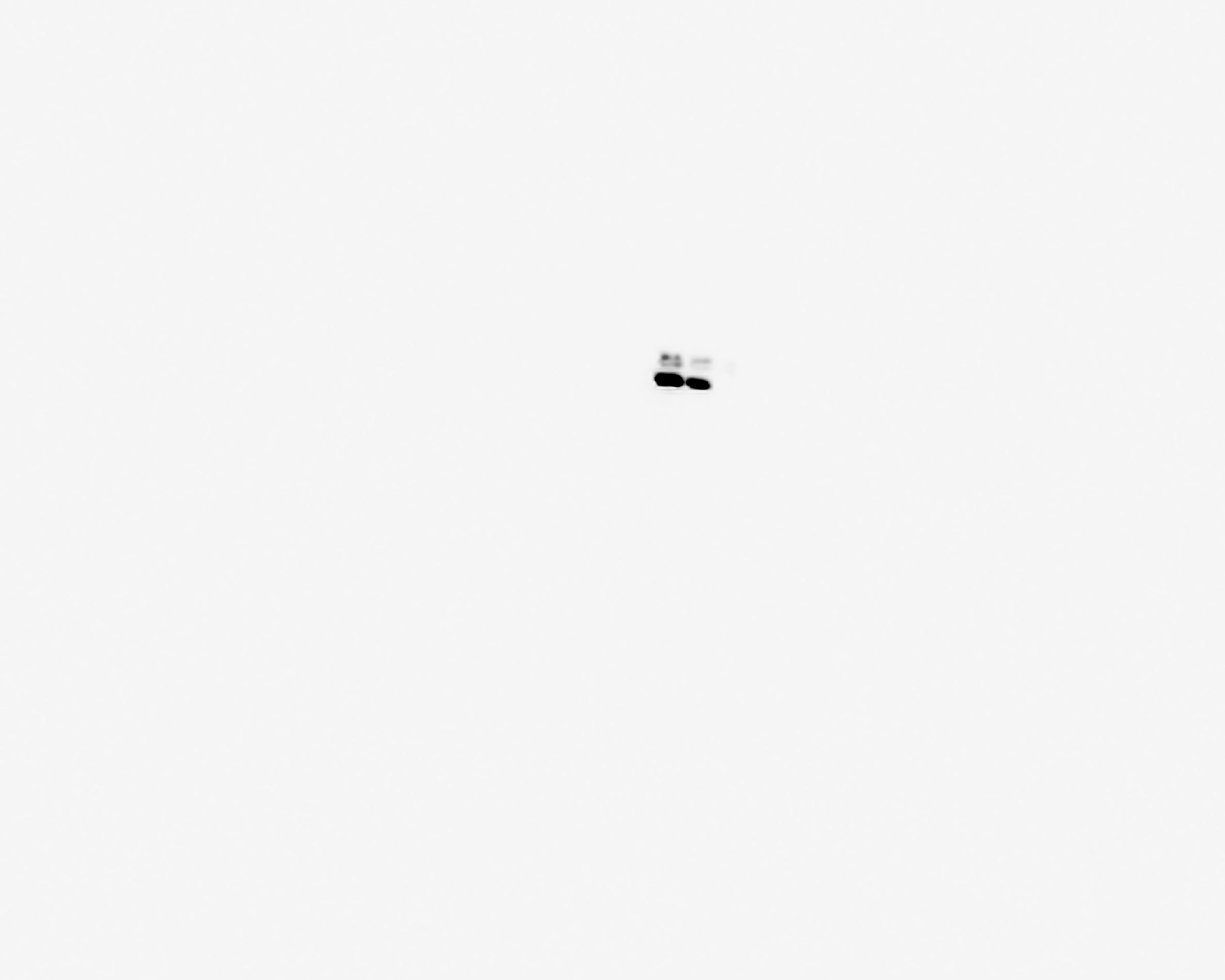

Supplement: Supplementary file 7 — Additional file 7. [file 12964_2024_1475_MOESM7_ESM.zip › Additional file 2/Figure 1N/Eca-109/snail.tif]

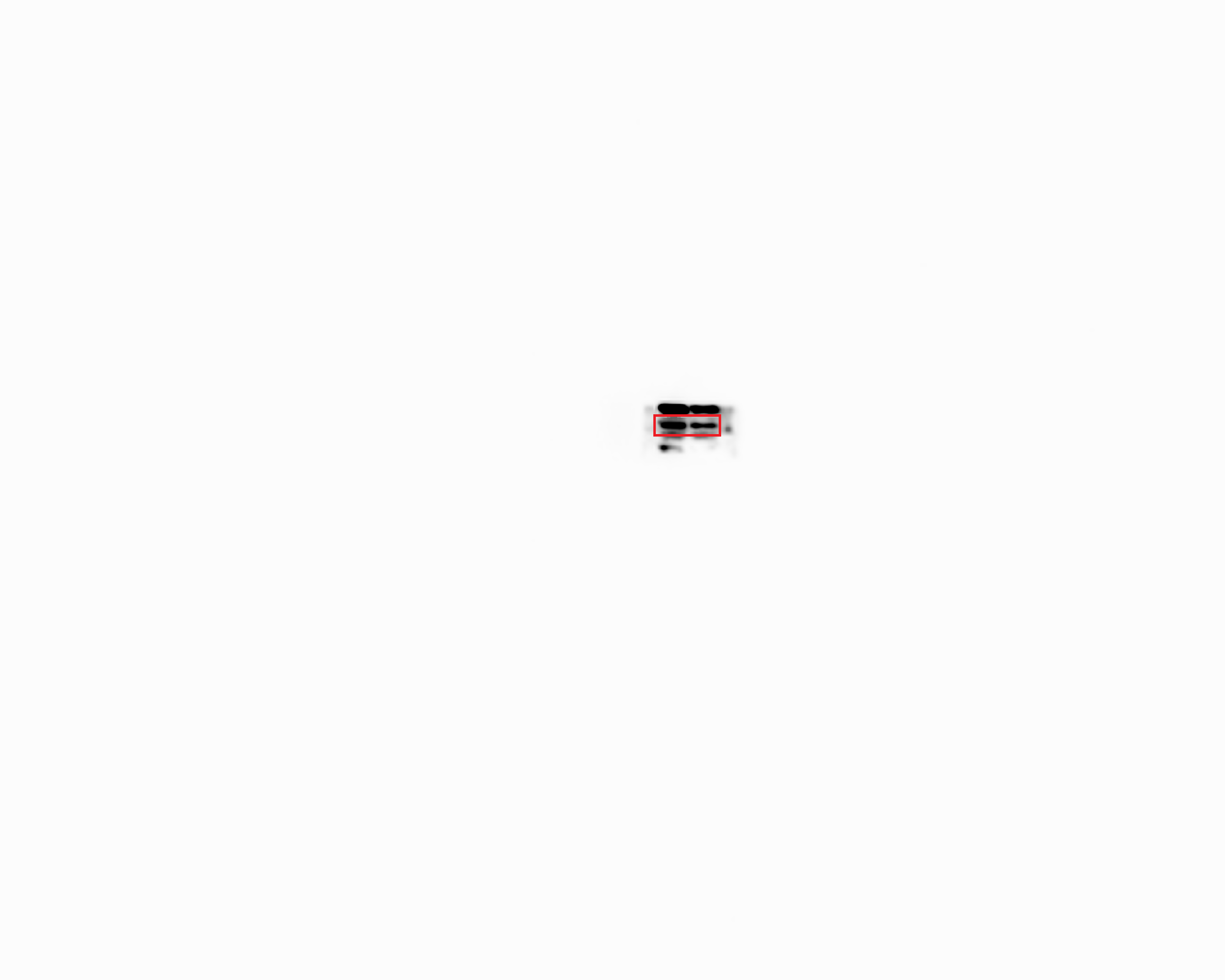

Supplement: Supplementary file 7 — Additional file 7. [file 12964_2024_1475_MOESM7_ESM.zip › Additional file 2/Figure 1N/Eca-109/Vimentin.tif]

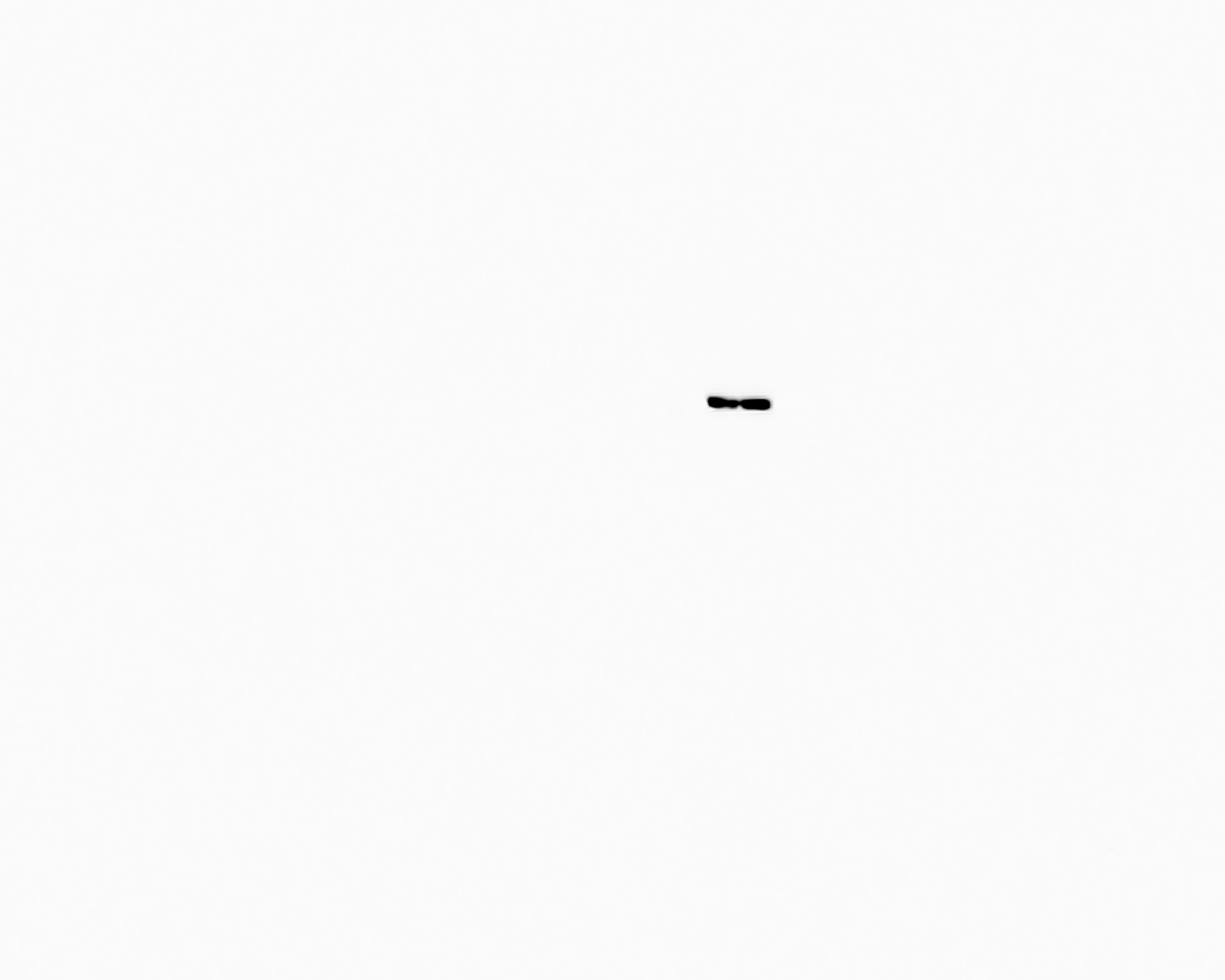

Supplement: Supplementary file 7 — Additional file 7. [file 12964_2024_1475_MOESM7_ESM.zip › Additional file 2/Figure 1N/Eca-109/a┬-actin.tif]

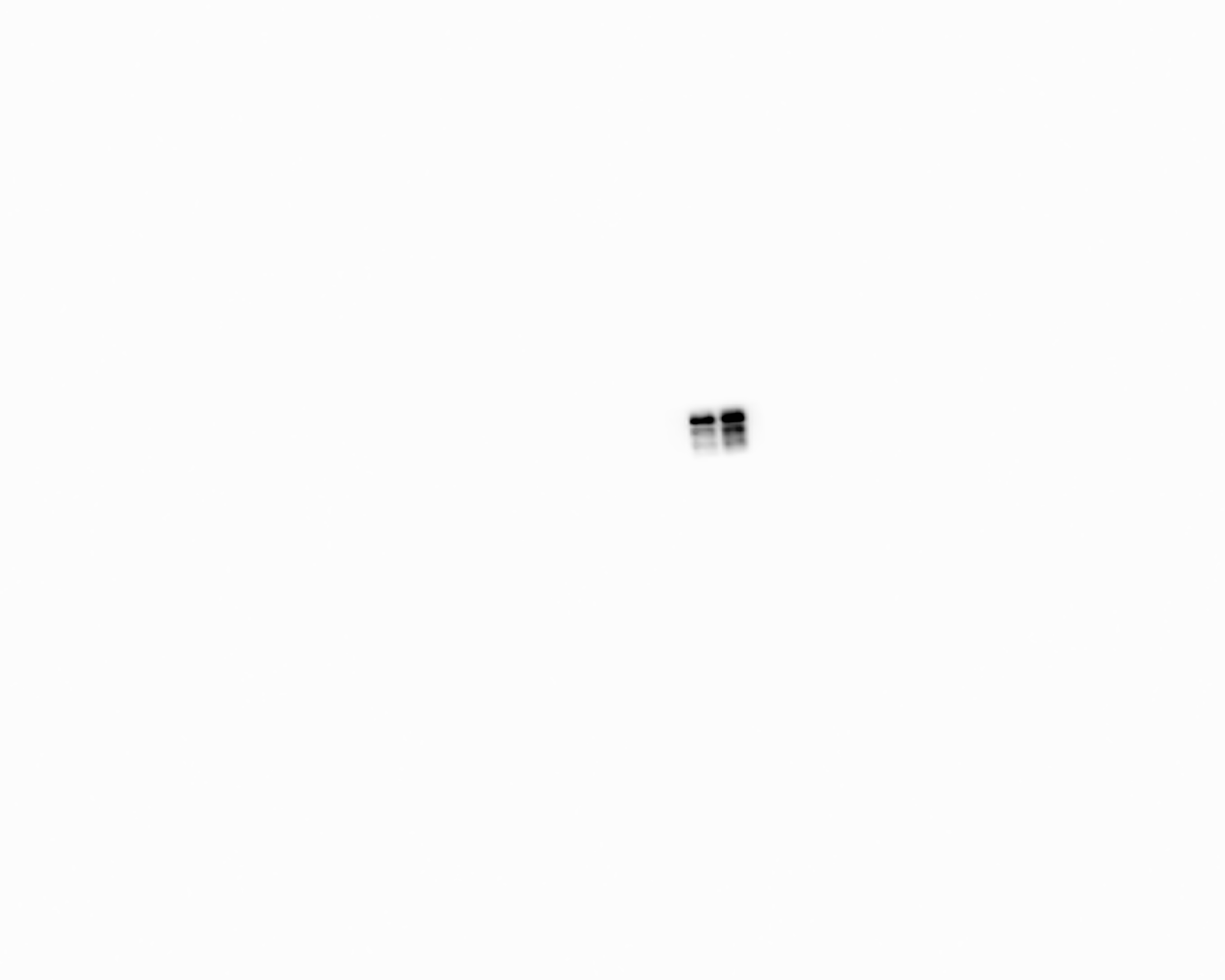

Supplement: Supplementary file 7 — Additional file 7. [file 12964_2024_1475_MOESM7_ESM.zip › Additional file 2/Figure 1N/TE-1/E-cadherin.tif]

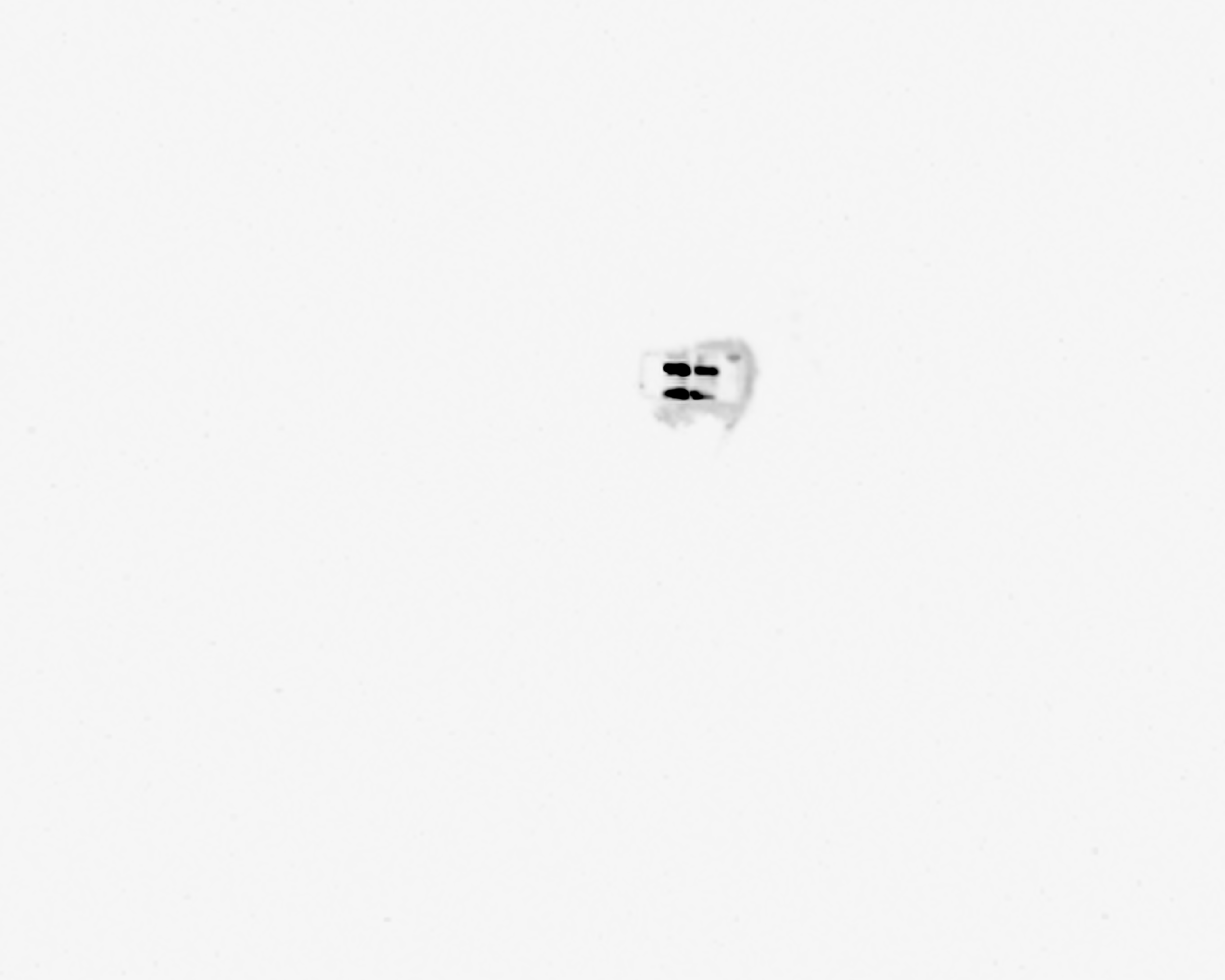

Supplement: Supplementary file 7 — Additional file 7. [file 12964_2024_1475_MOESM7_ESM.zip › Additional file 2/Figure 1N/TE-1/N-cadherin.tif]

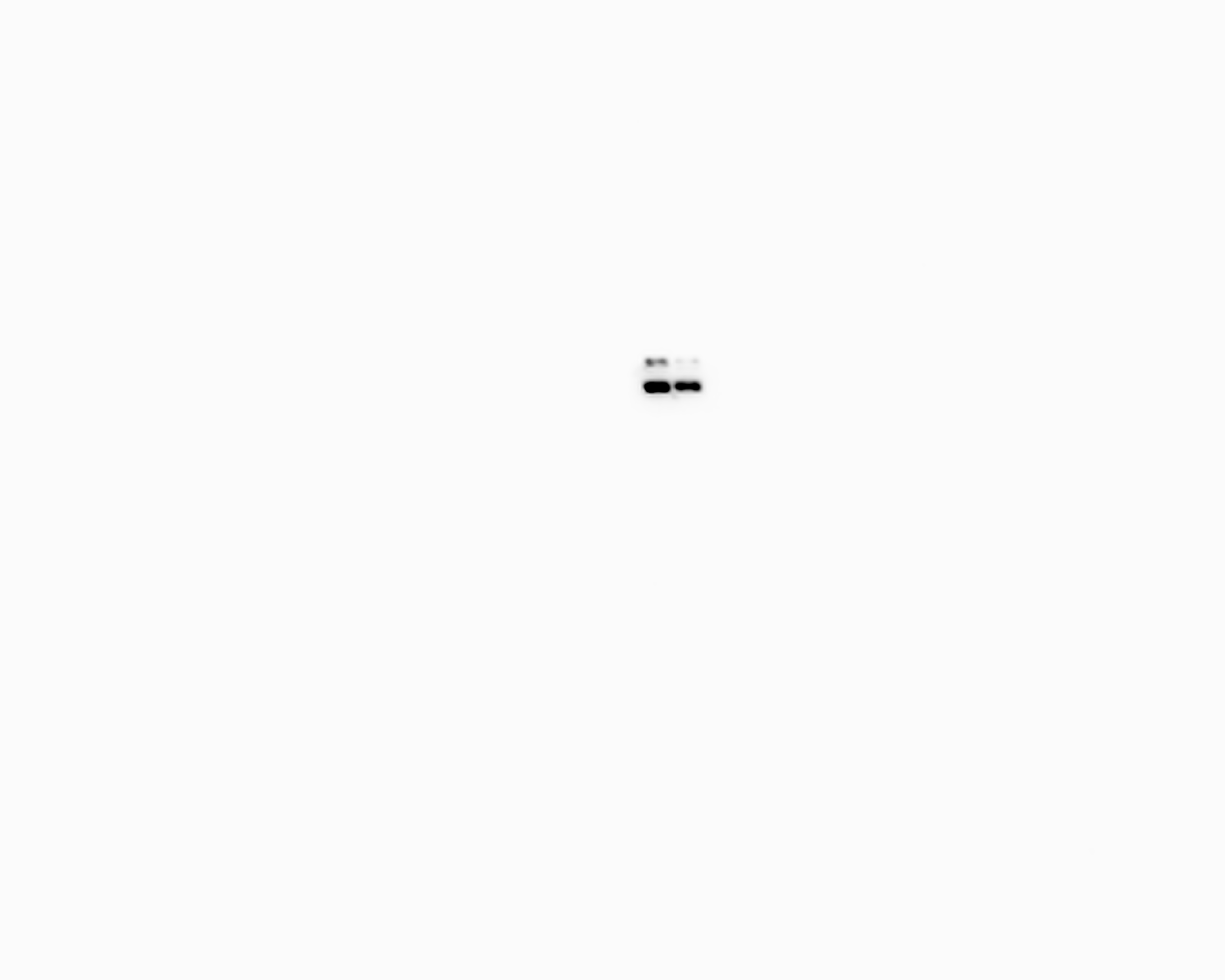

Supplement: Supplementary file 7 — Additional file 7. [file 12964_2024_1475_MOESM7_ESM.zip › Additional file 2/Figure 1N/TE-1/snail.tif]

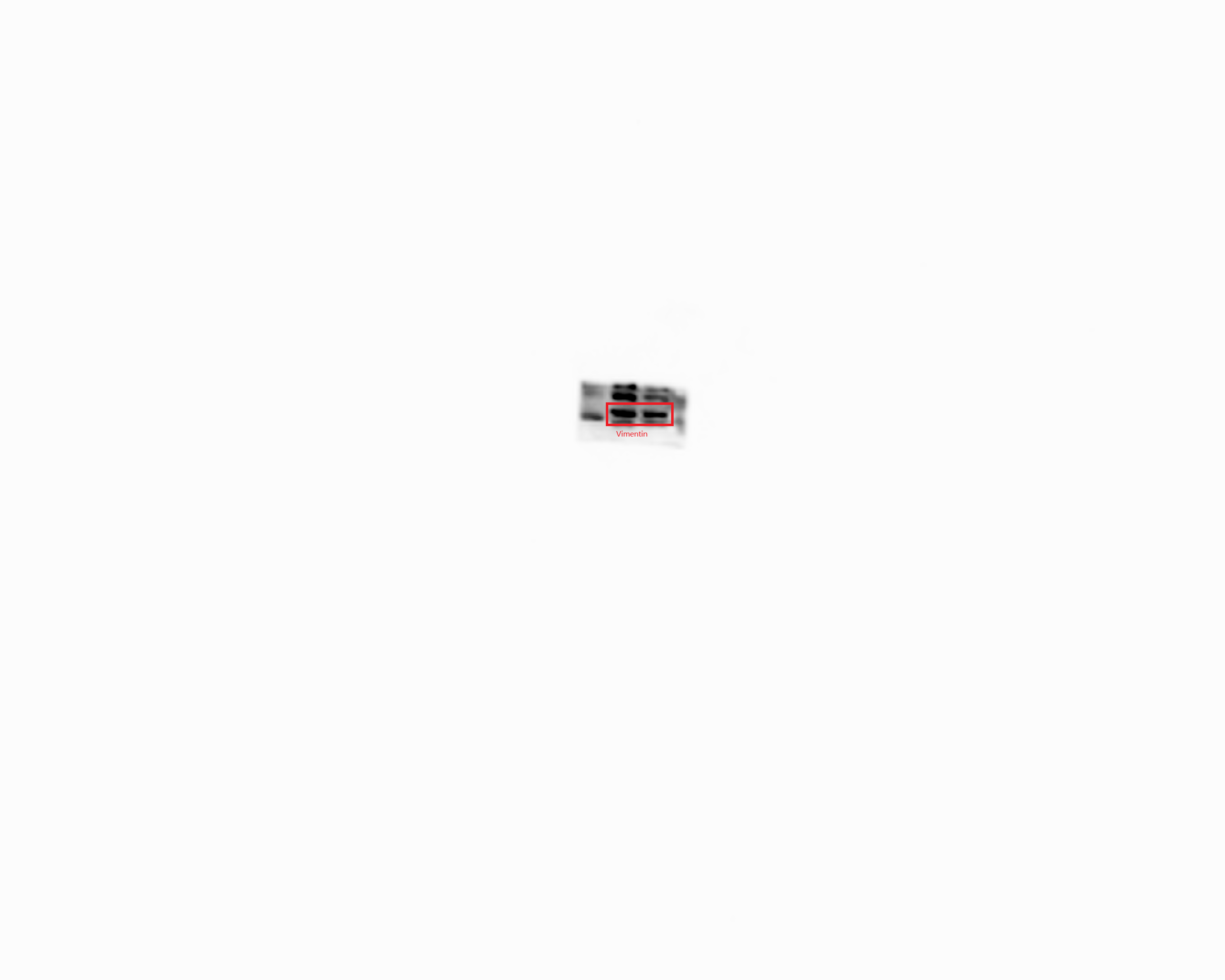

Supplement: Supplementary file 7 — Additional file 7. [file 12964_2024_1475_MOESM7_ESM.zip › Additional file 2/Figure 1N/TE-1/Vimentin.tif]

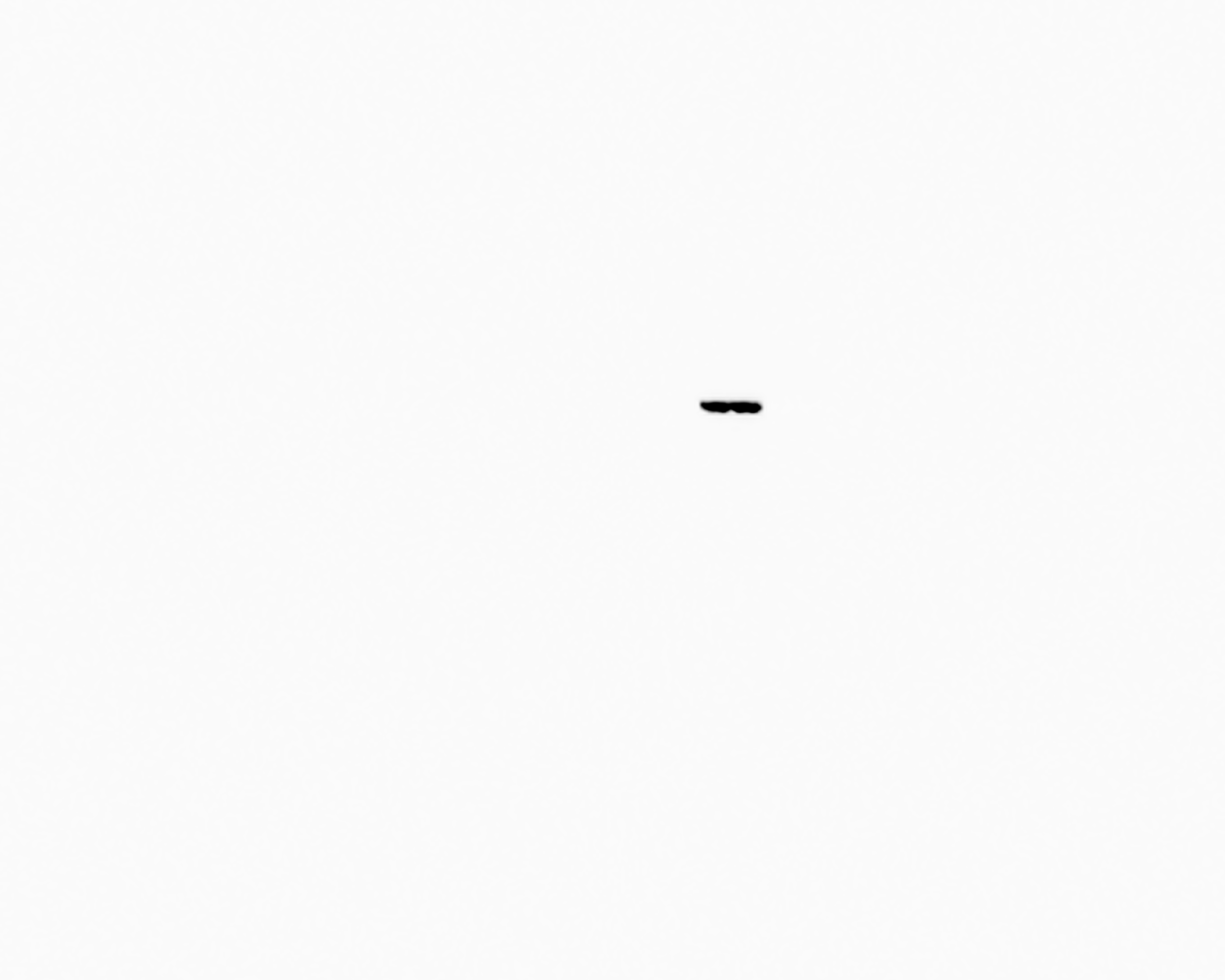

Supplement: Supplementary file 7 — Additional file 7. [file 12964_2024_1475_MOESM7_ESM.zip › Additional file 2/Figure 1N/TE-1/a┬-actin.tif]

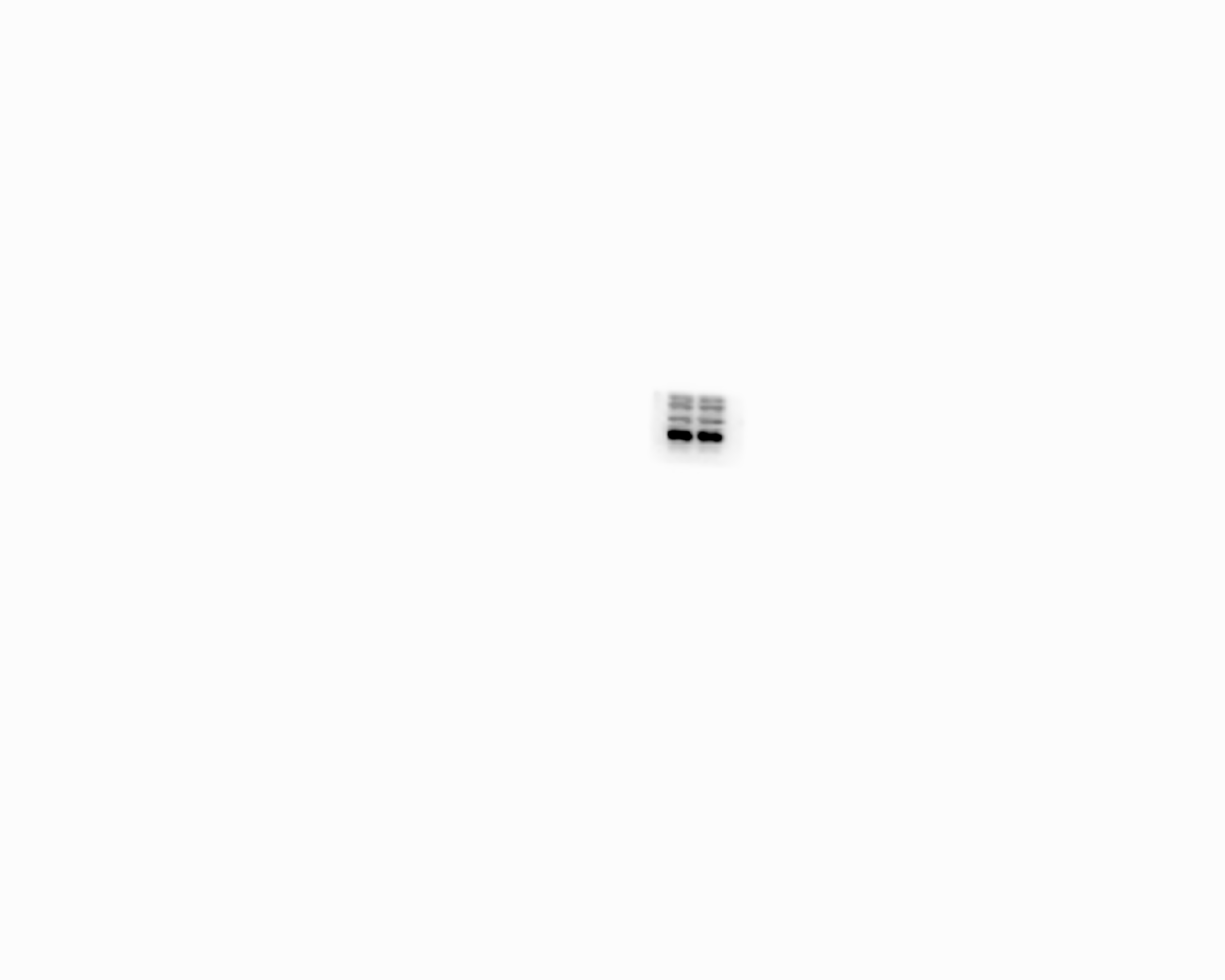

Supplement: Supplementary file 7 — Additional file 7. [file 12964_2024_1475_MOESM7_ESM.zip › Additional file 2/Figure 2I/Eca-109/c-myc.tif]

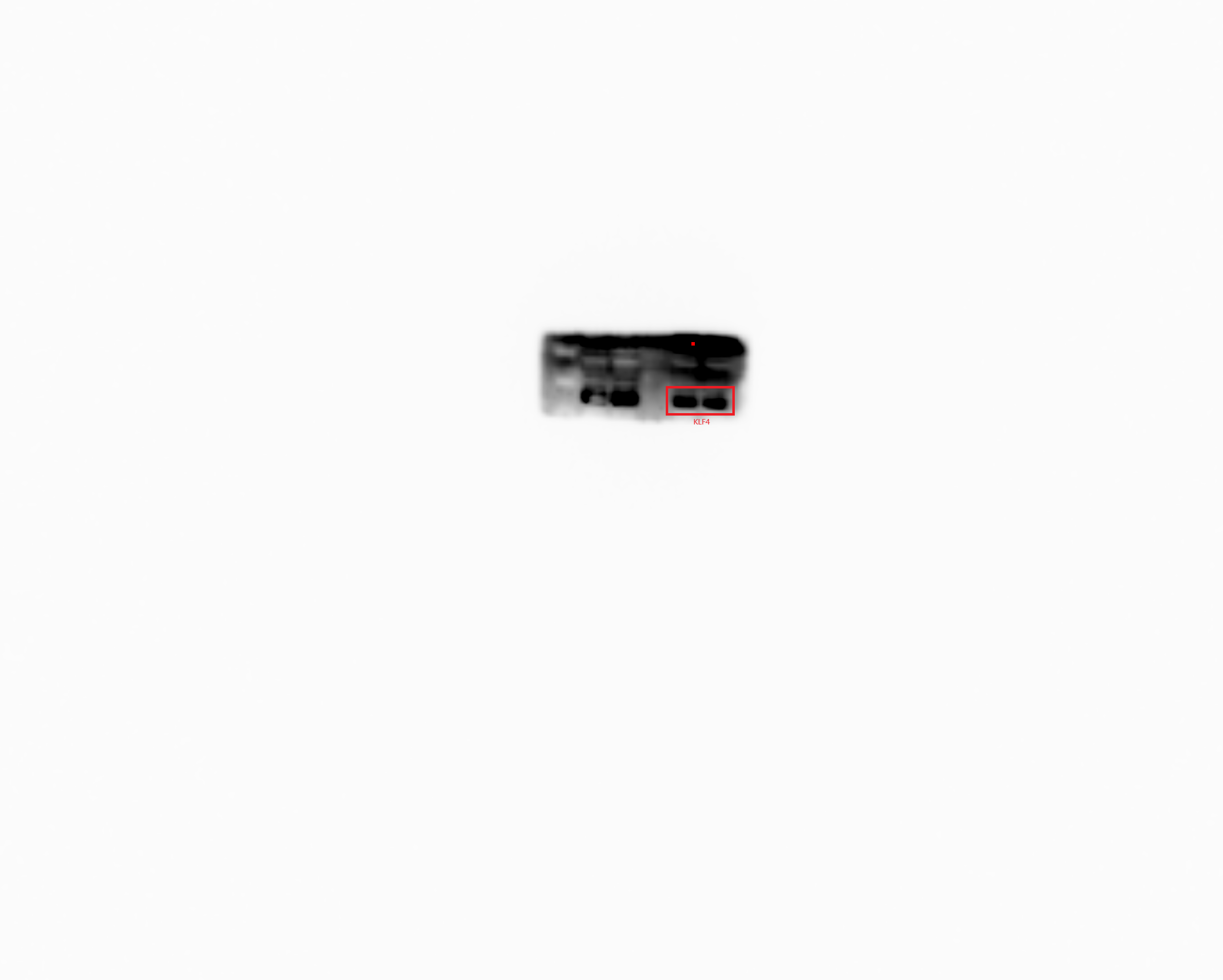

Supplement: Supplementary file 7 — Additional file 7. [file 12964_2024_1475_MOESM7_ESM.zip › Additional file 2/Figure 2I/Eca-109/klf4.tif]

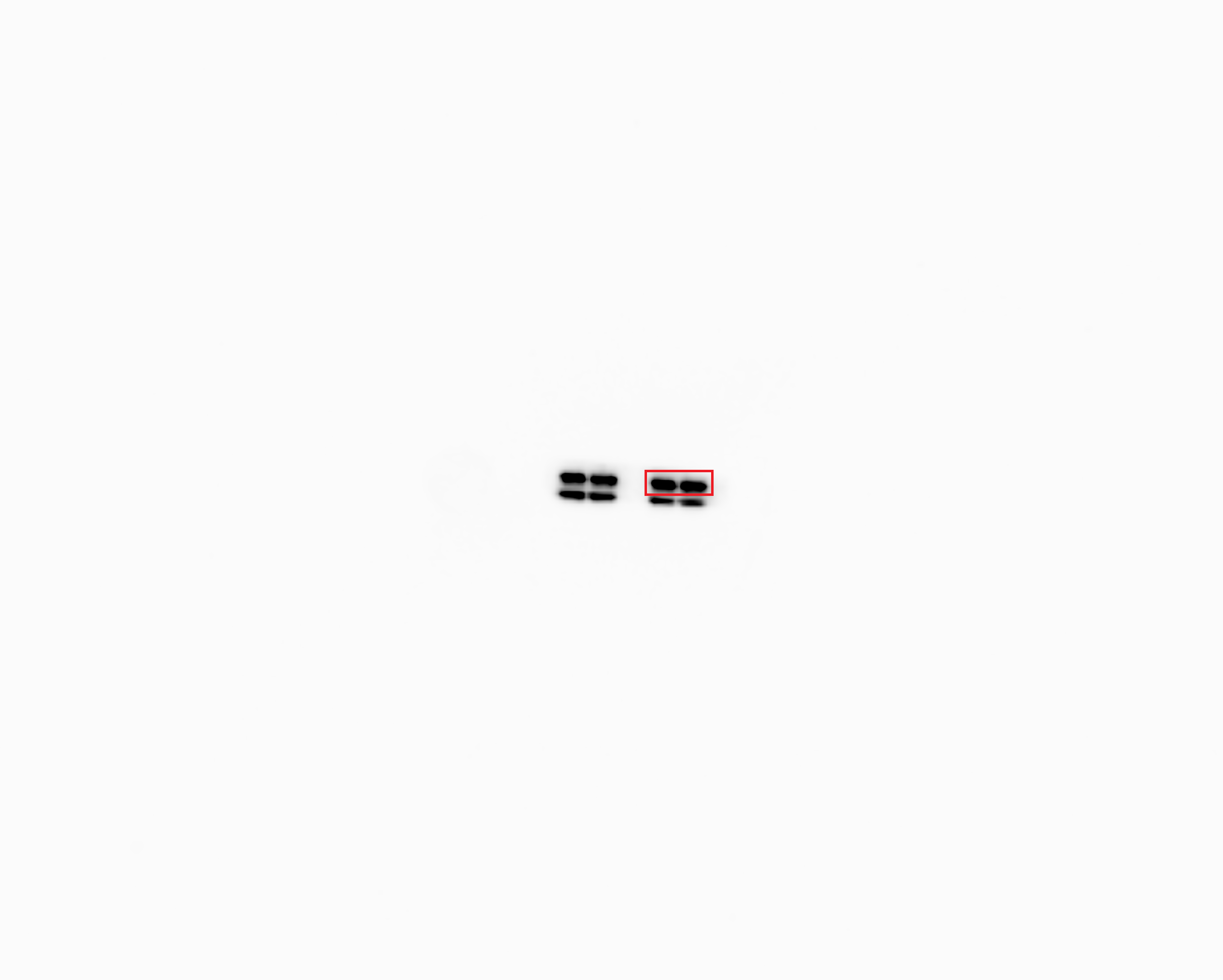

Supplement: Supplementary file 7 — Additional file 7. [file 12964_2024_1475_MOESM7_ESM.zip › Additional file 2/Figure 2I/Eca-109/nanog.tif]

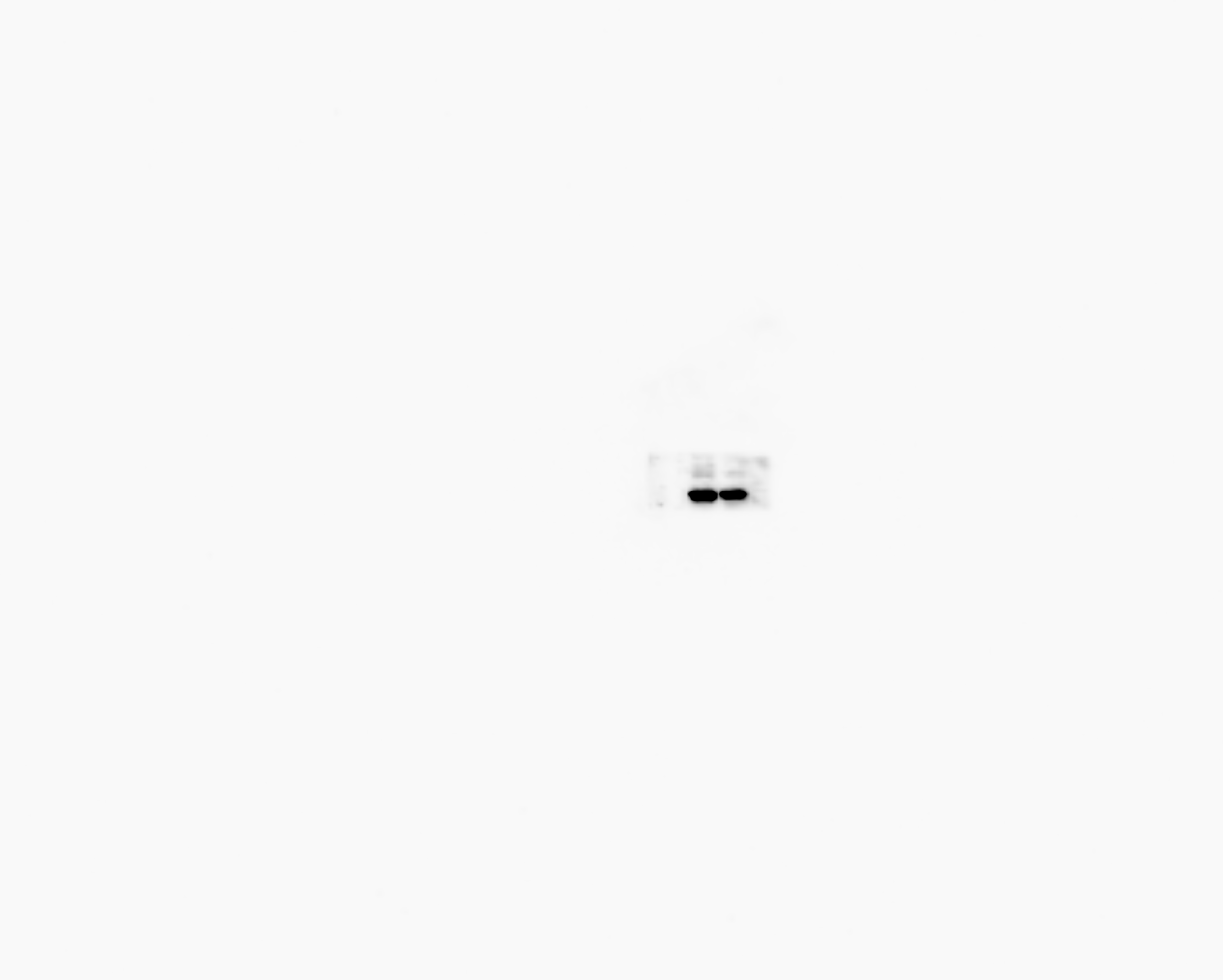

Supplement: Supplementary file 7 — Additional file 7. [file 12964_2024_1475_MOESM7_ESM.zip › Additional file 2/Figure 2I/Eca-109/oct4.tif]

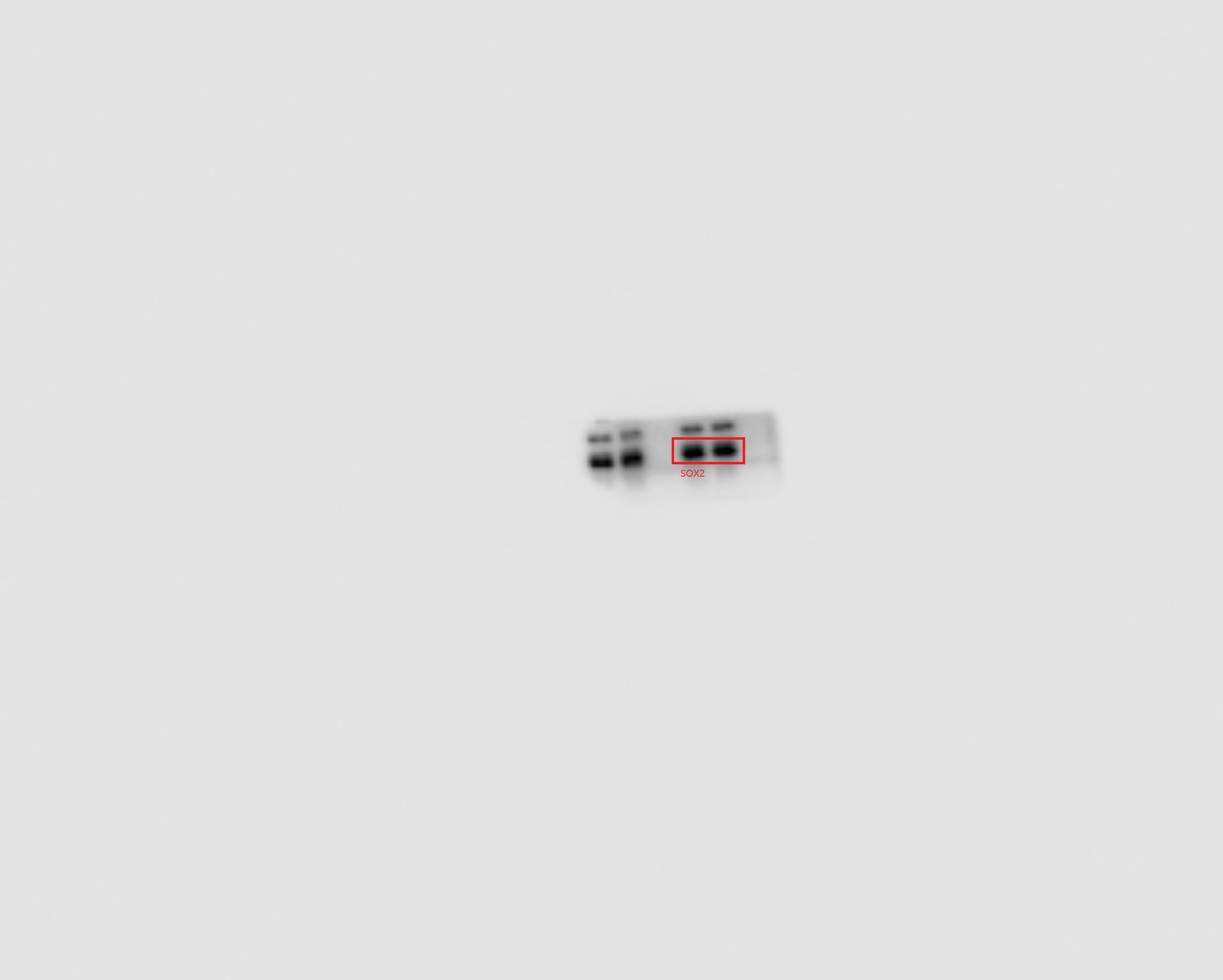

Supplement: Supplementary file 7 — Additional file 7. [file 12964_2024_1475_MOESM7_ESM.zip › Additional file 2/Figure 2I/Eca-109/sox2.tif]

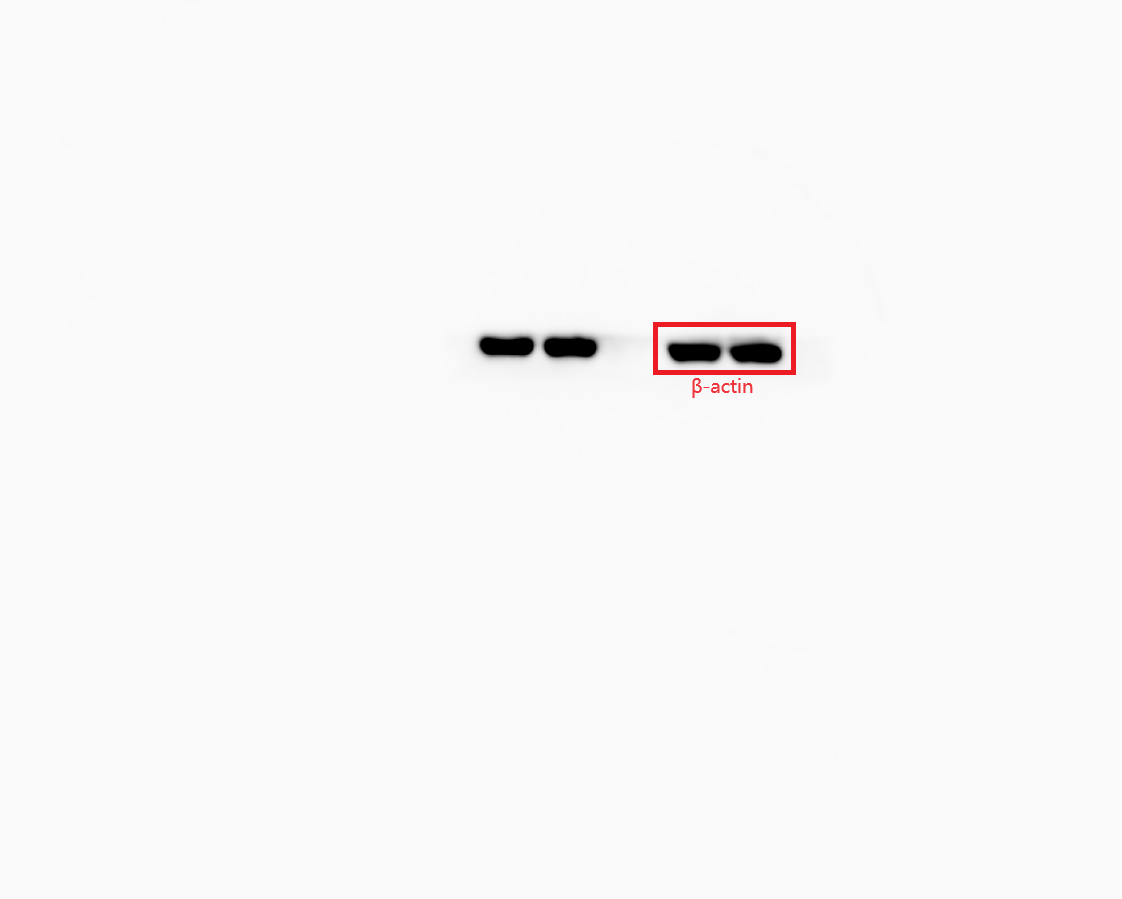

Supplement: Supplementary file 7 — Additional file 7. [file 12964_2024_1475_MOESM7_ESM.zip › Additional file 2/Figure 2I/Eca-109/a┬-actin.tif]

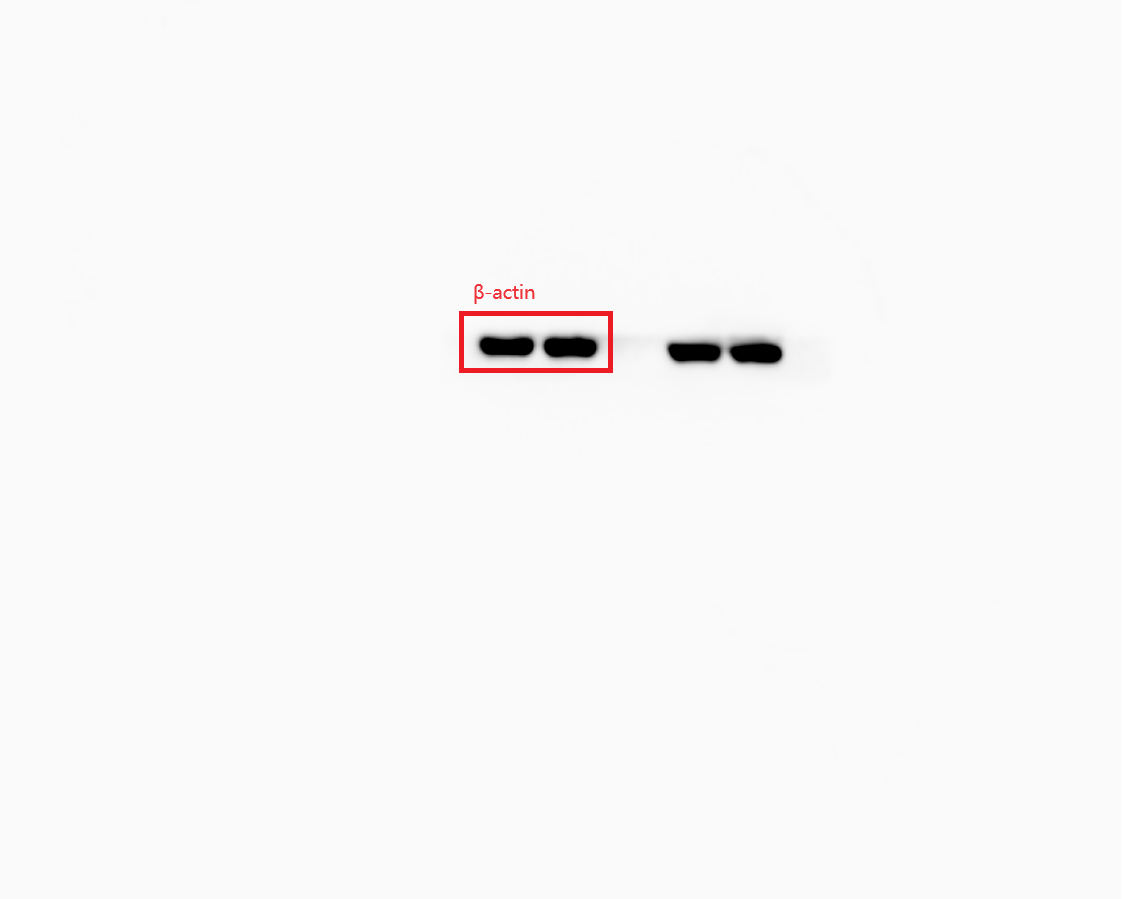

Supplement: Supplementary file 7 — Additional file 7. [file 12964_2024_1475_MOESM7_ESM.zip › Additional file 2/Figure 2I/TE-1/actin te-1.tif]

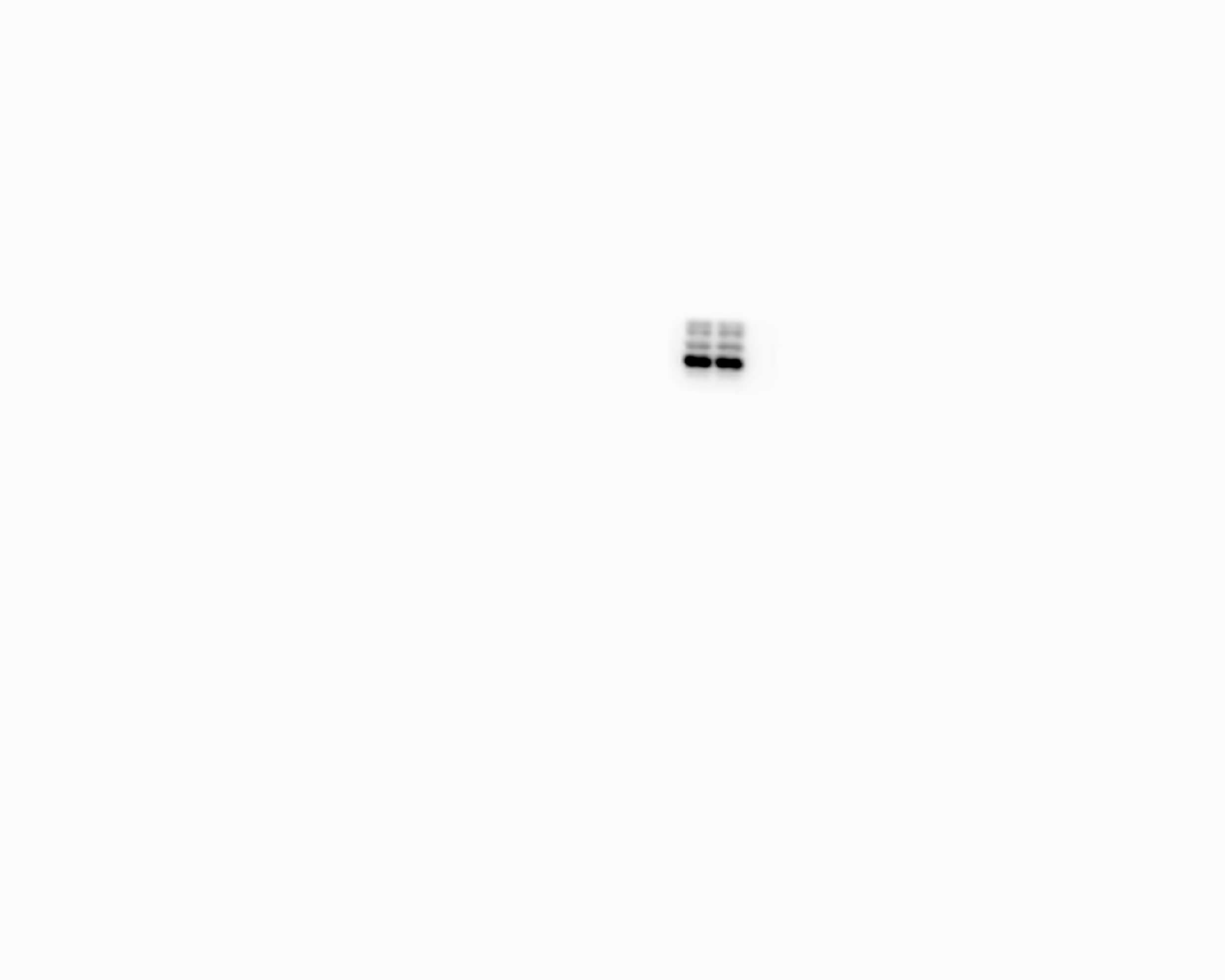

Supplement: Supplementary file 7 — Additional file 7. [file 12964_2024_1475_MOESM7_ESM.zip › Additional file 2/Figure 2I/TE-1/c-myc.tif]

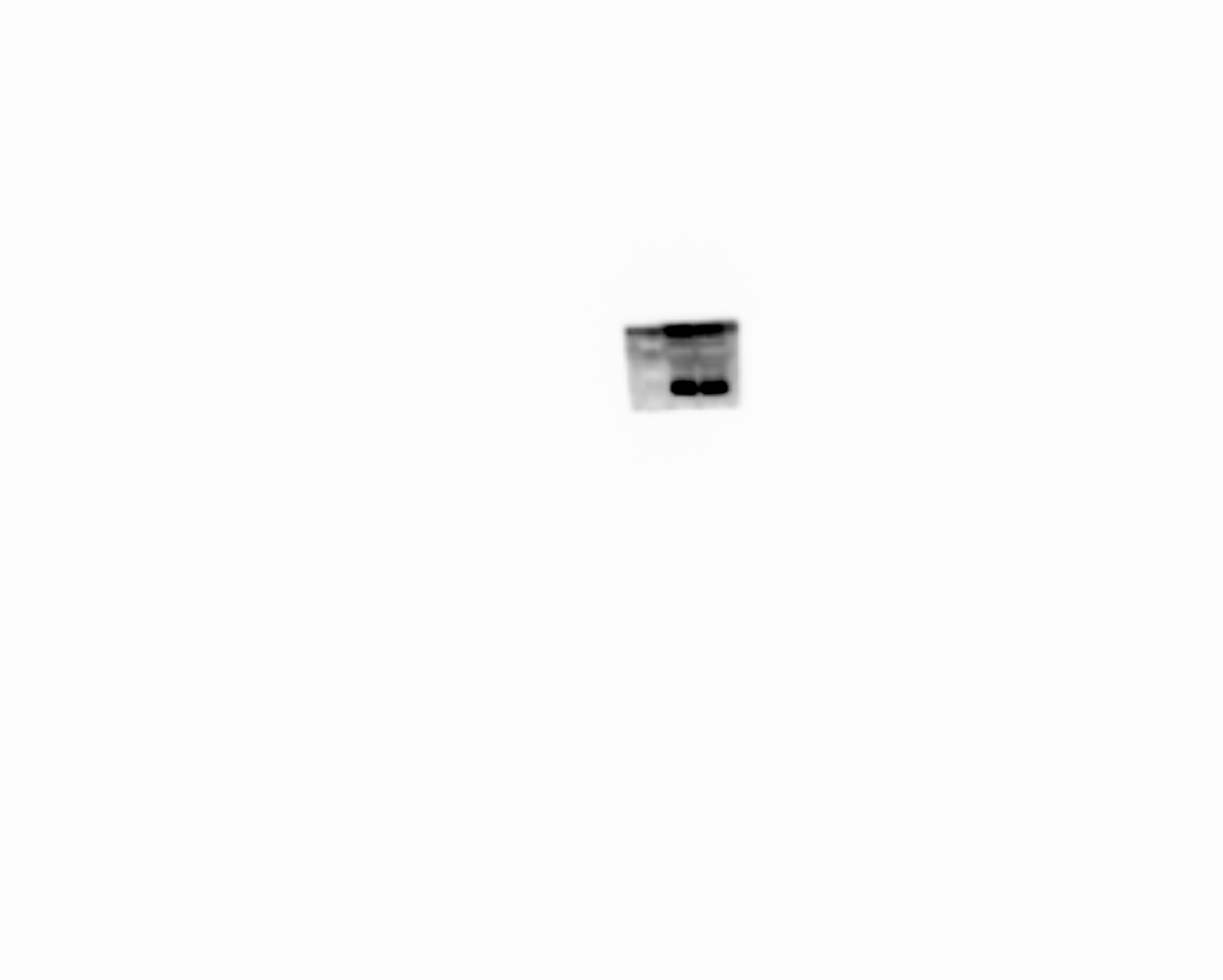

Supplement: Supplementary file 7 — Additional file 7. [file 12964_2024_1475_MOESM7_ESM.zip › Additional file 2/Figure 2I/TE-1/klf4.tif]

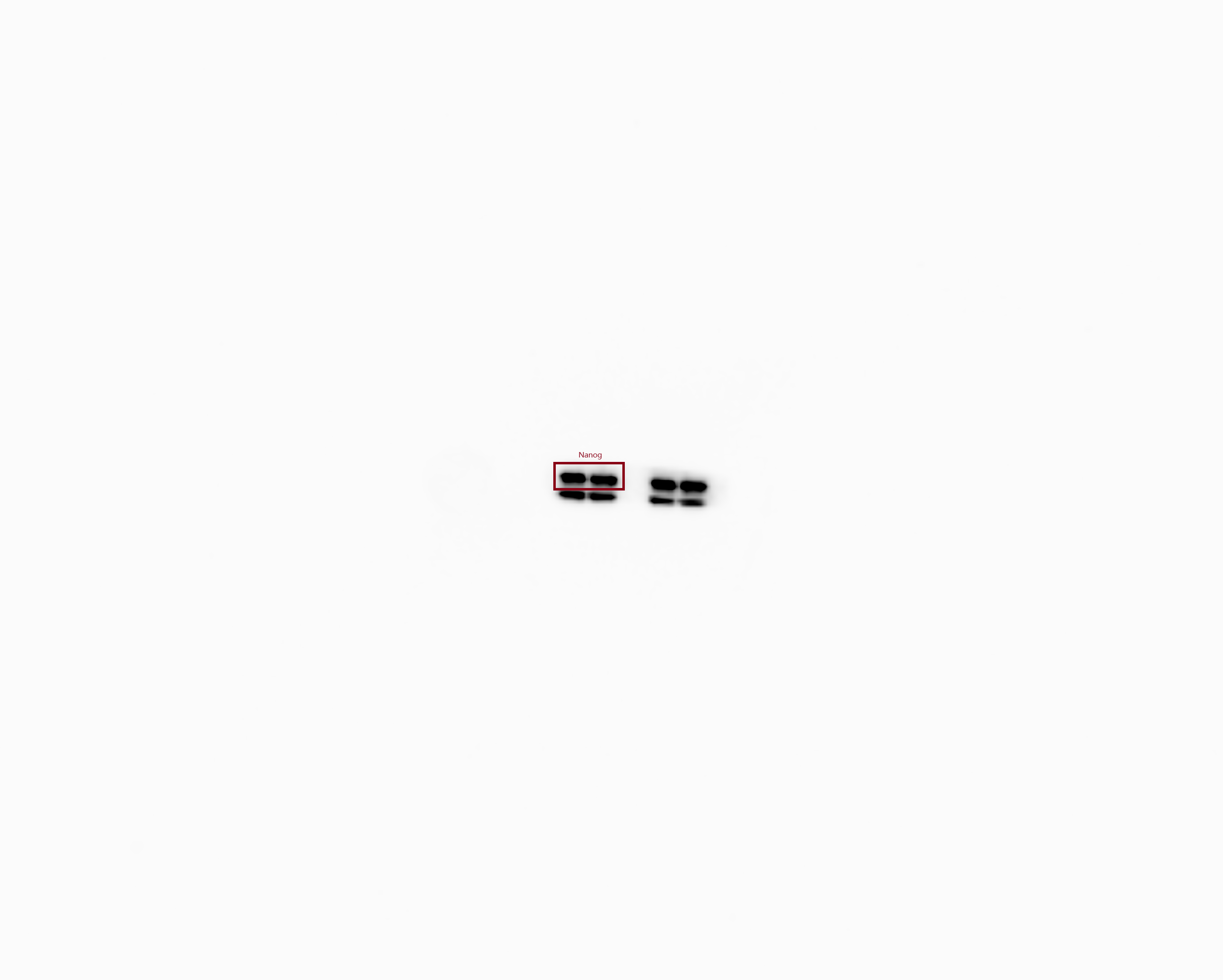

Supplement: Supplementary file 7 — Additional file 7. [file 12964_2024_1475_MOESM7_ESM.zip › Additional file 2/Figure 2I/TE-1/nanog-TE-1.tif]

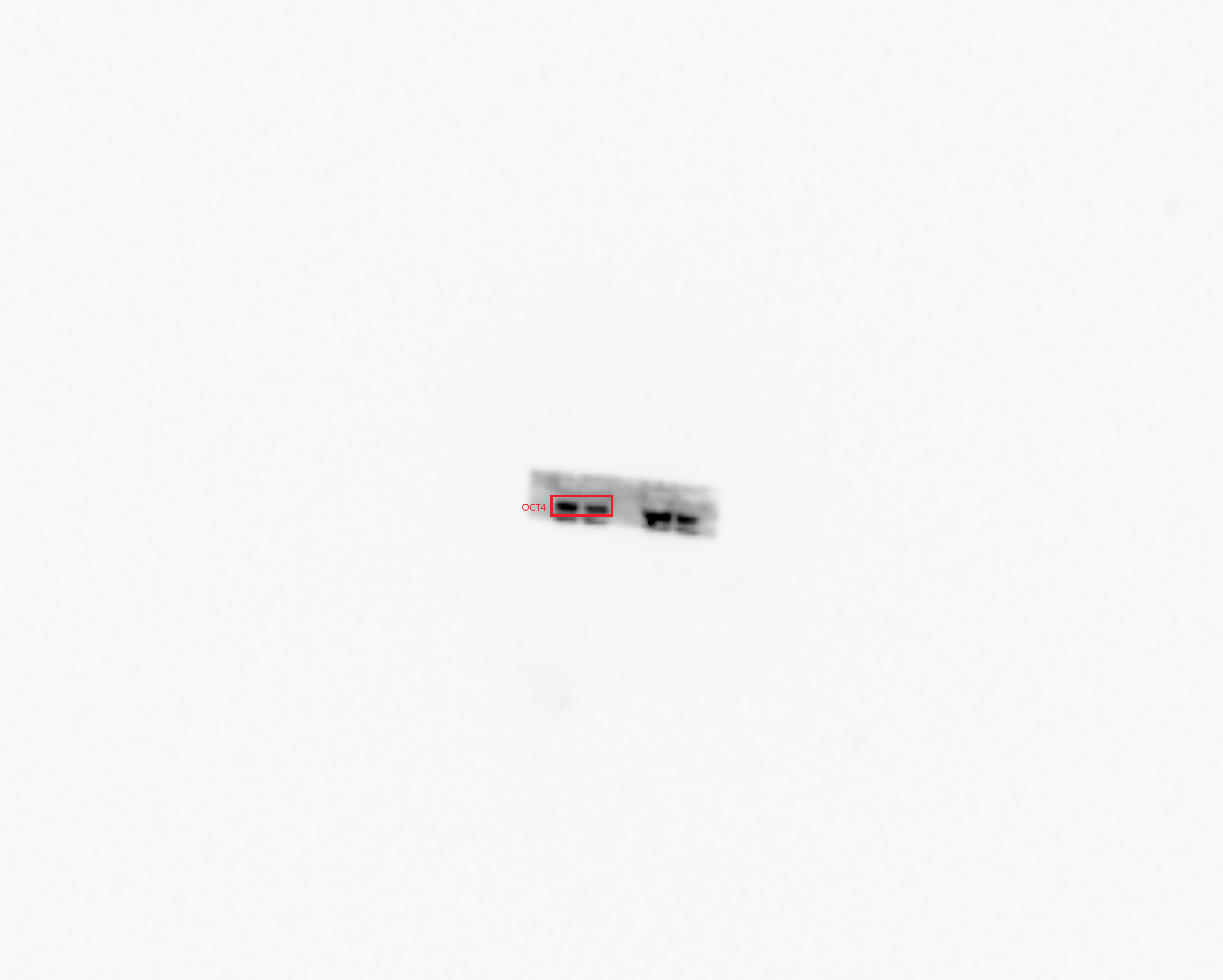

Supplement: Supplementary file 7 — Additional file 7. [file 12964_2024_1475_MOESM7_ESM.zip › Additional file 2/Figure 2I/TE-1/oct4.tif]

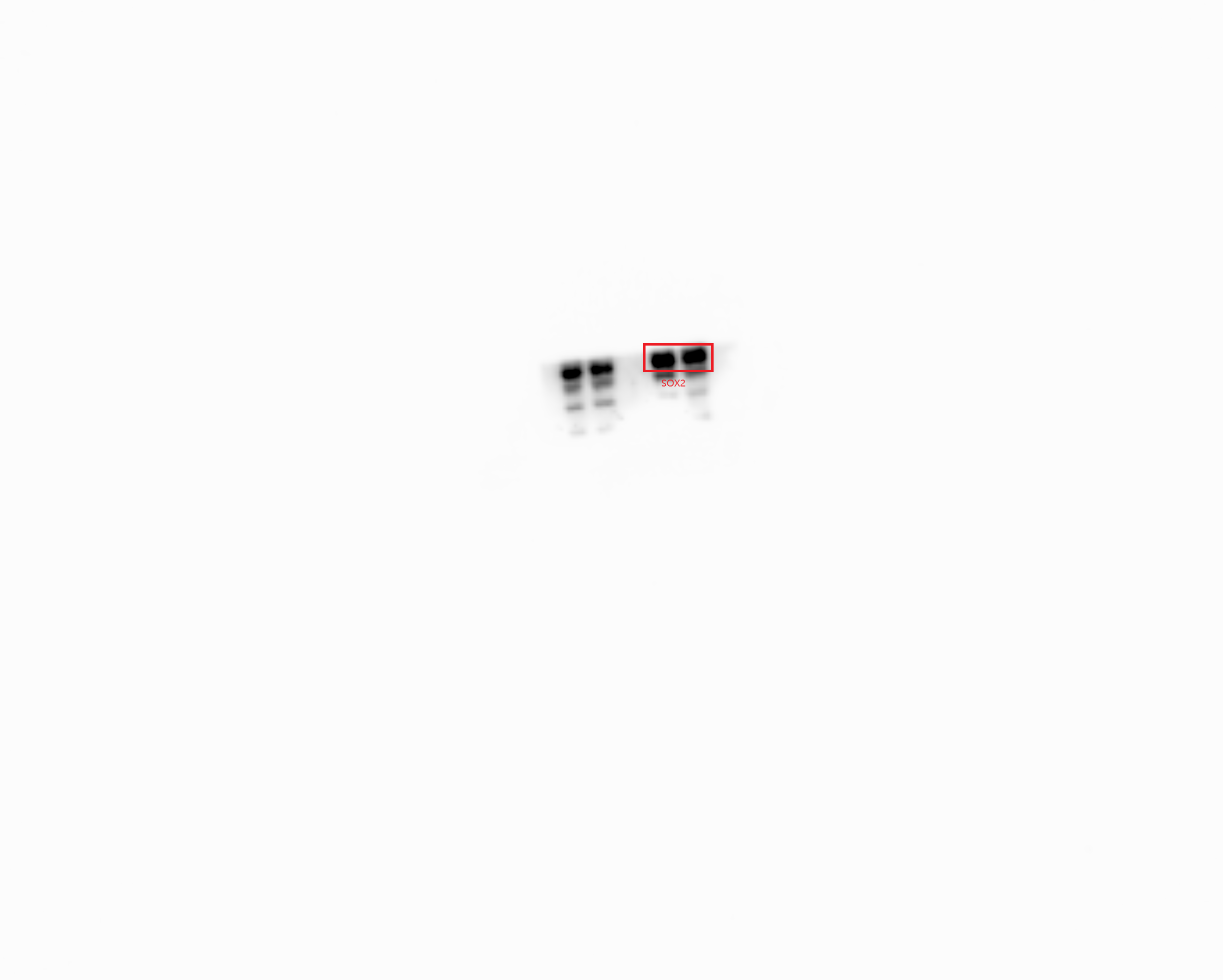

Supplement: Supplementary file 7 — Additional file 7. [file 12964_2024_1475_MOESM7_ESM.zip › Additional file 2/Figure 2I/TE-1/sox2.tif]

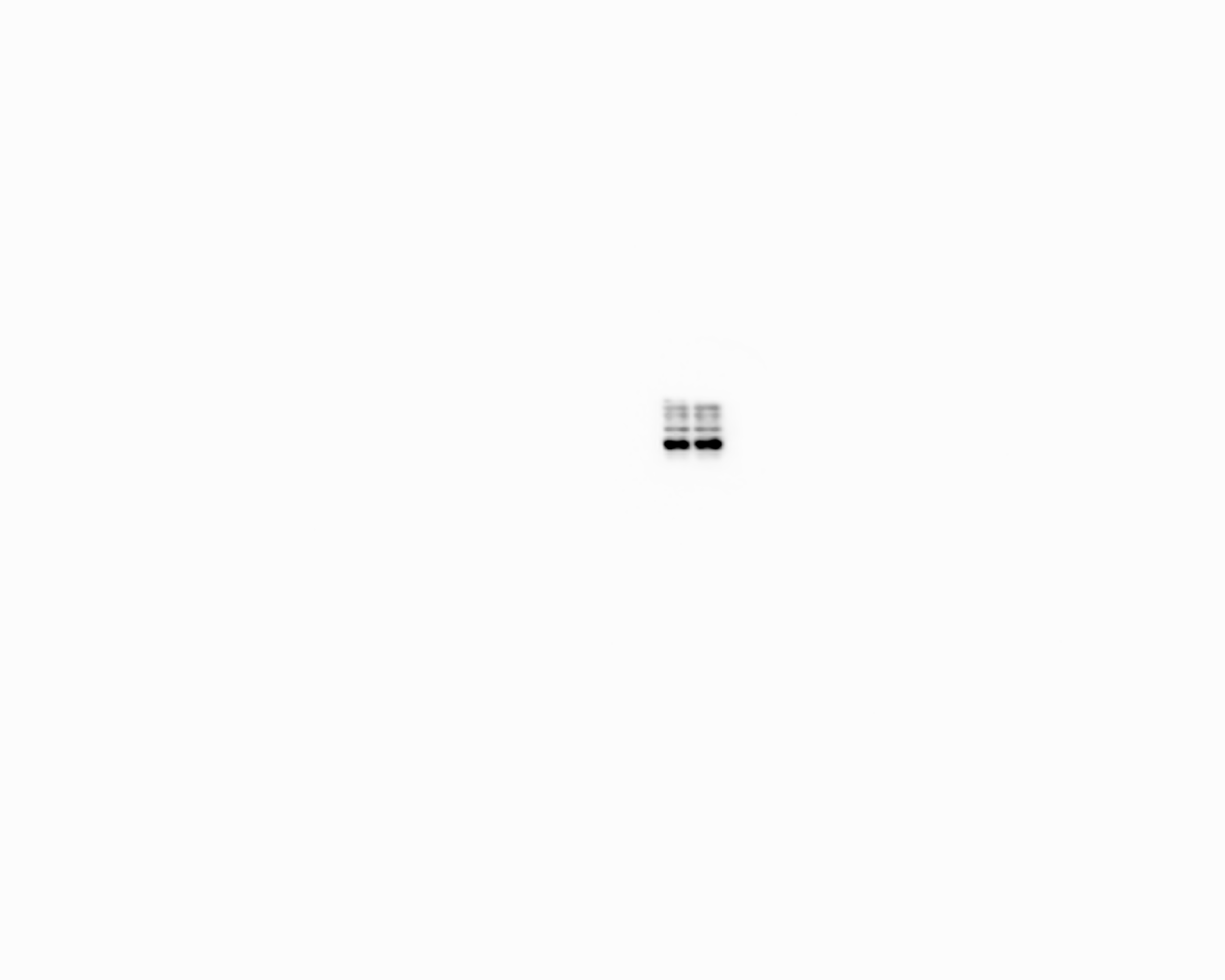

Supplement: Supplementary file 7 — Additional file 7. [file 12964_2024_1475_MOESM7_ESM.zip › Additional file 2/Figure 2J/KYSE-150/c-myc.tif]

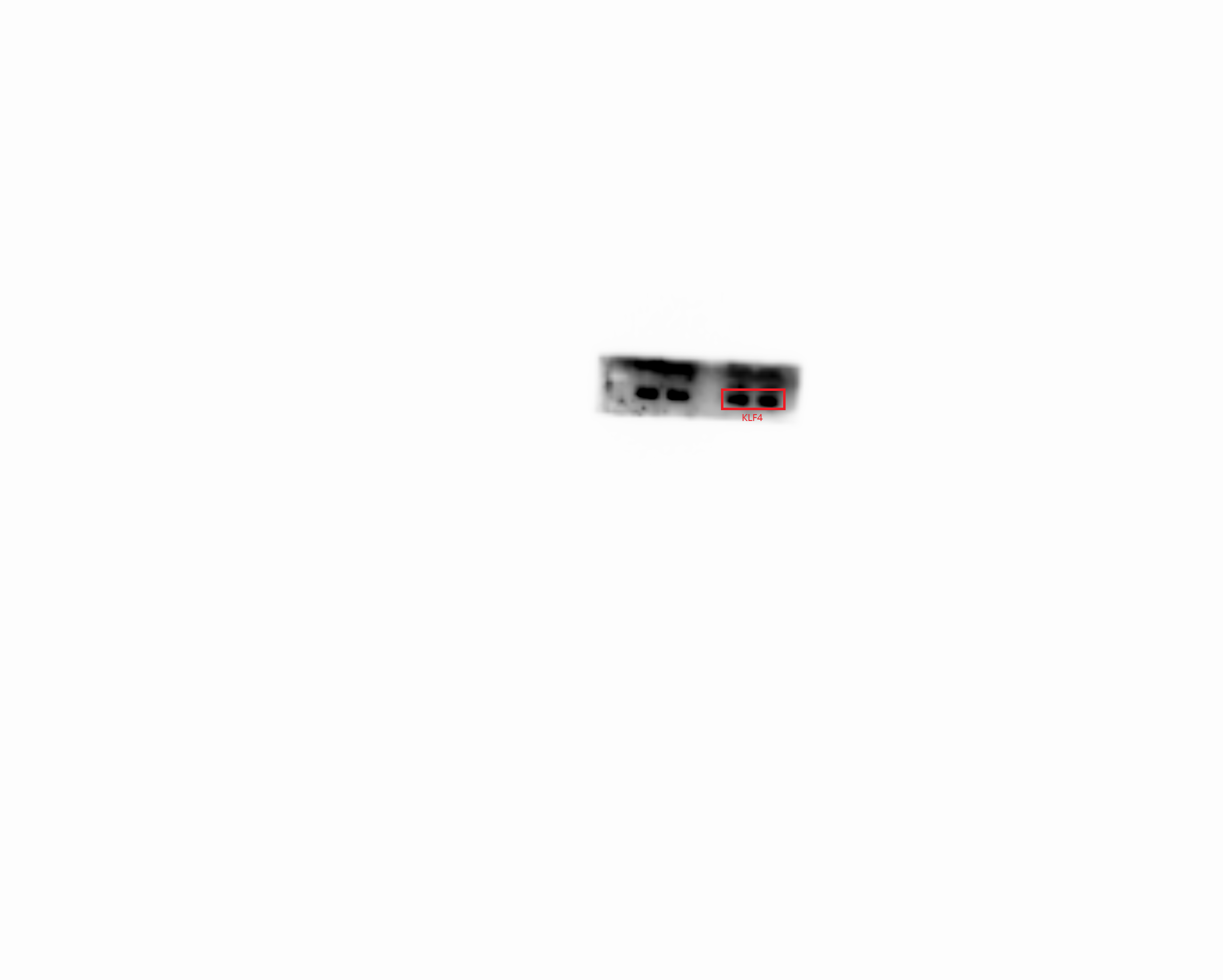

Supplement: Supplementary file 7 — Additional file 7. [file 12964_2024_1475_MOESM7_ESM.zip › Additional file 2/Figure 2J/KYSE-150/klf4.tif]

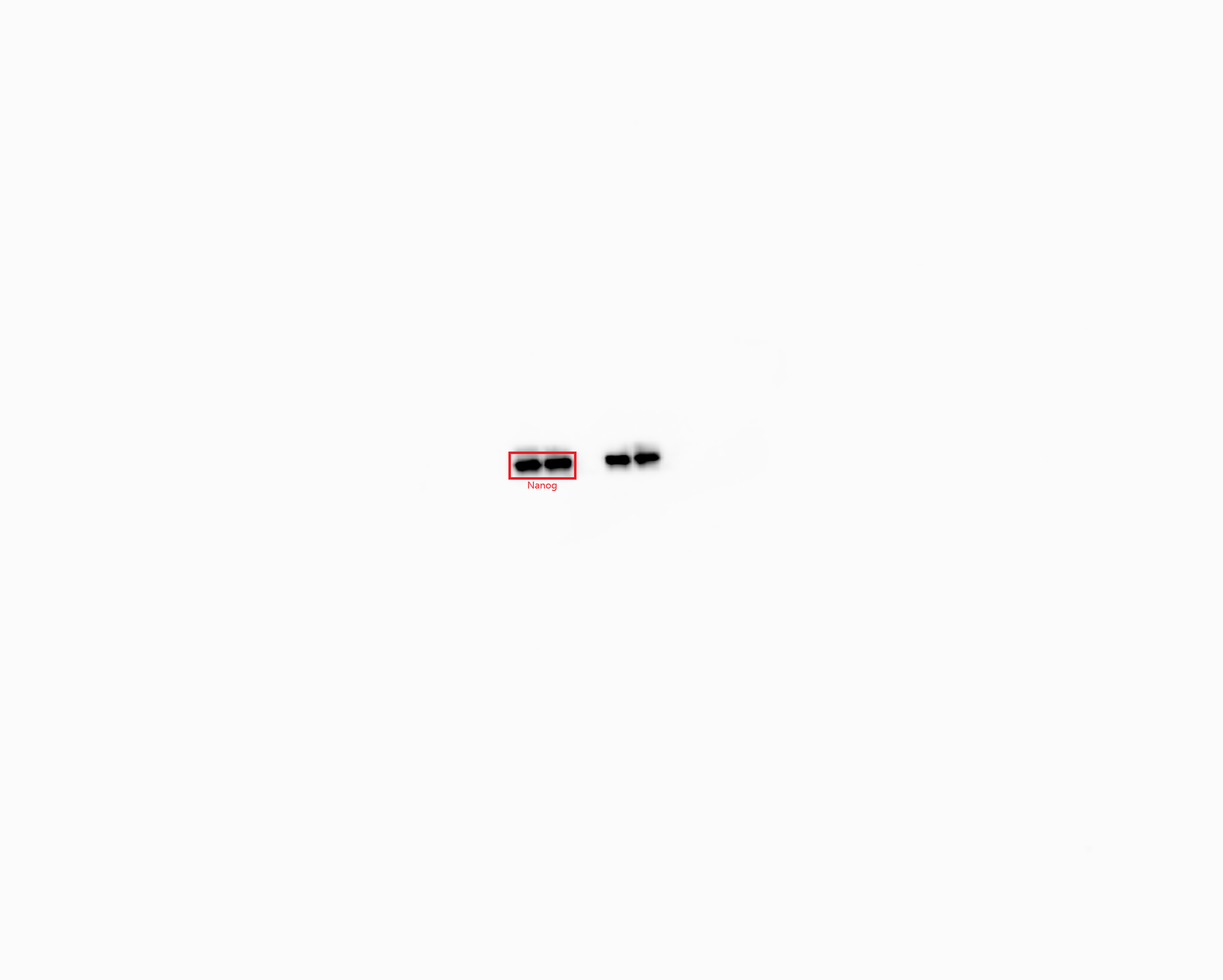

Supplement: Supplementary file 7 — Additional file 7. [file 12964_2024_1475_MOESM7_ESM.zip › Additional file 2/Figure 2J/KYSE-150/nanog.tif]

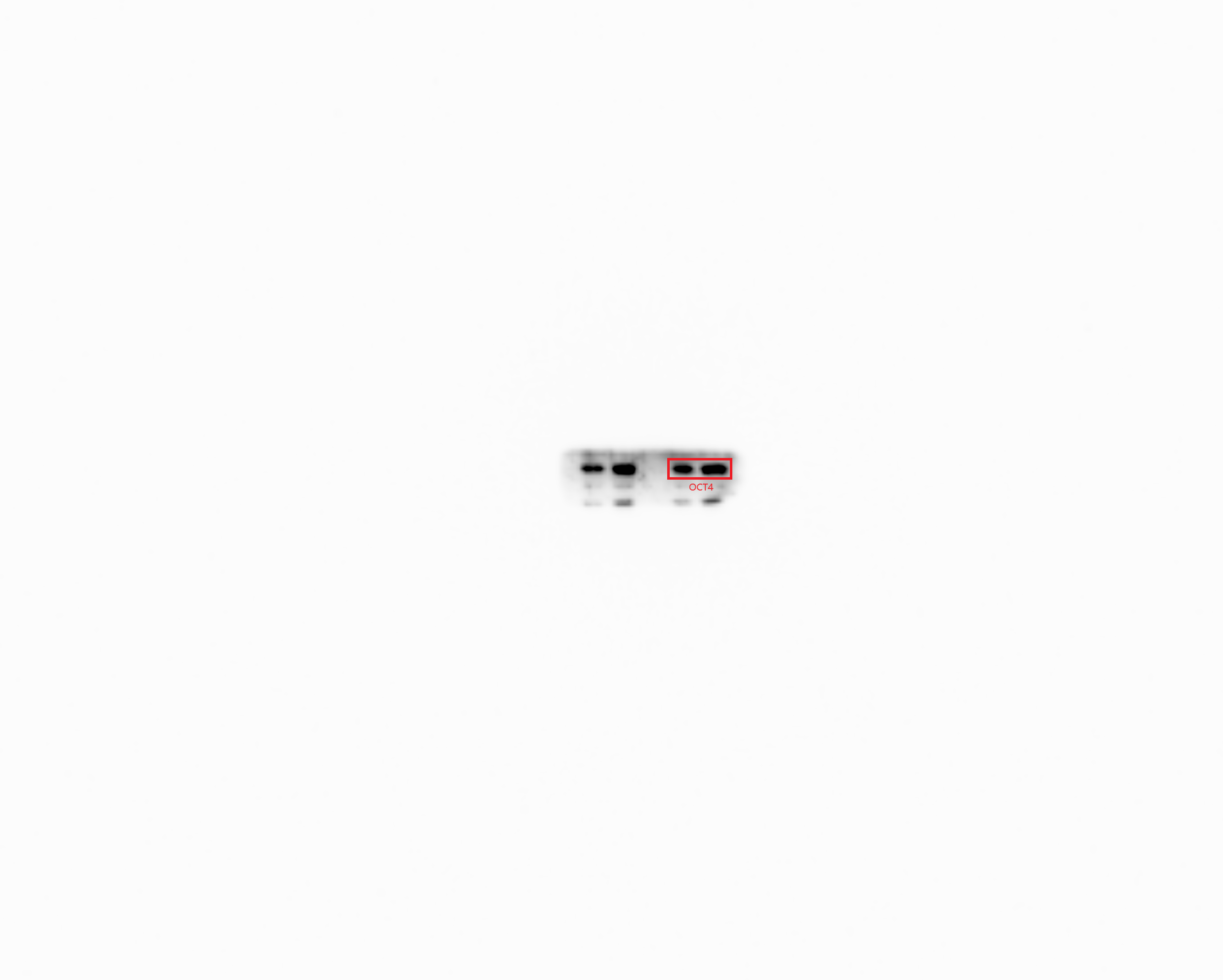

Supplement: Supplementary file 7 — Additional file 7. [file 12964_2024_1475_MOESM7_ESM.zip › Additional file 2/Figure 2J/KYSE-150/oct4.tif]

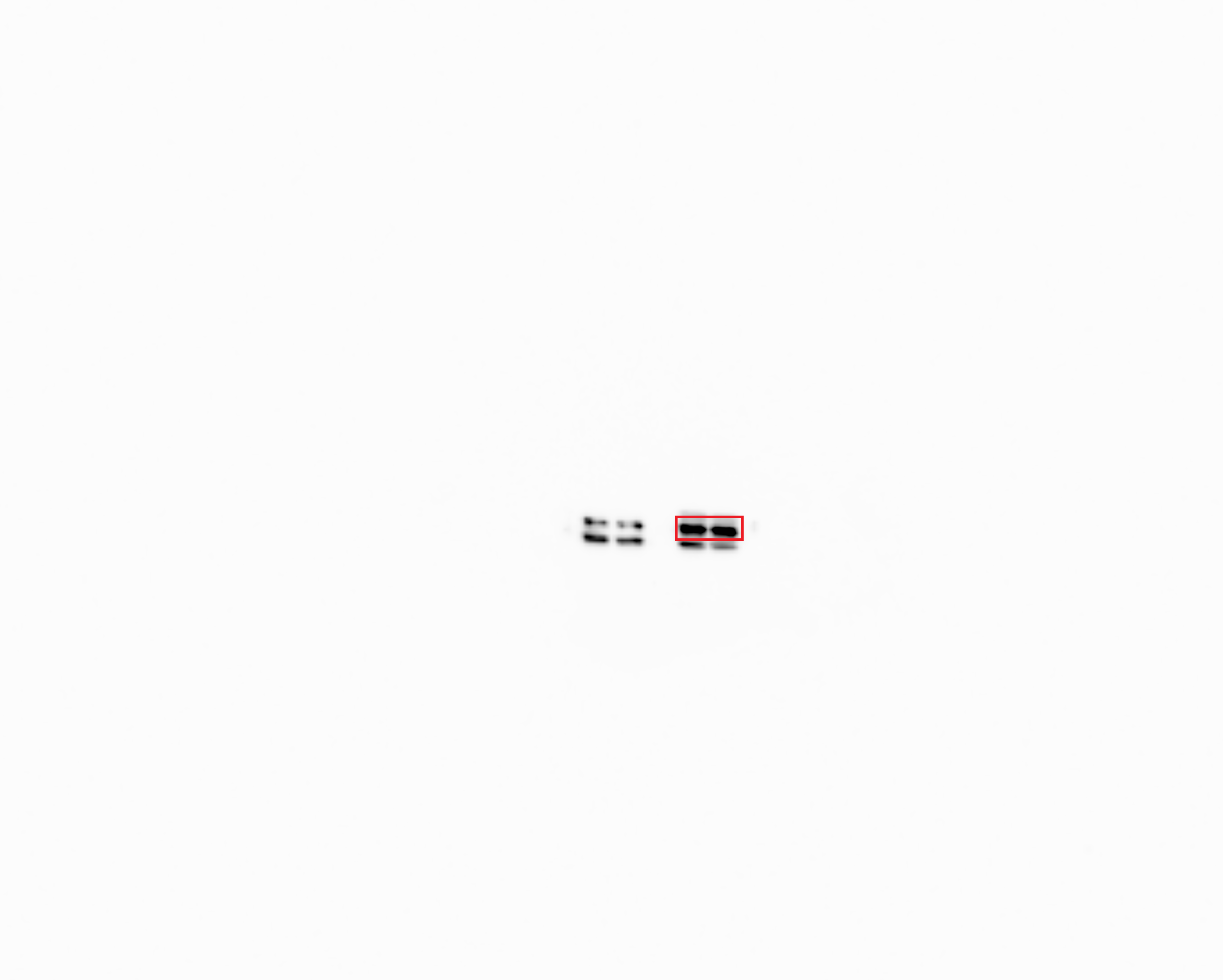

Supplement: Supplementary file 7 — Additional file 7. [file 12964_2024_1475_MOESM7_ESM.zip › Additional file 2/Figure 2J/KYSE-150/SOX2.tif]

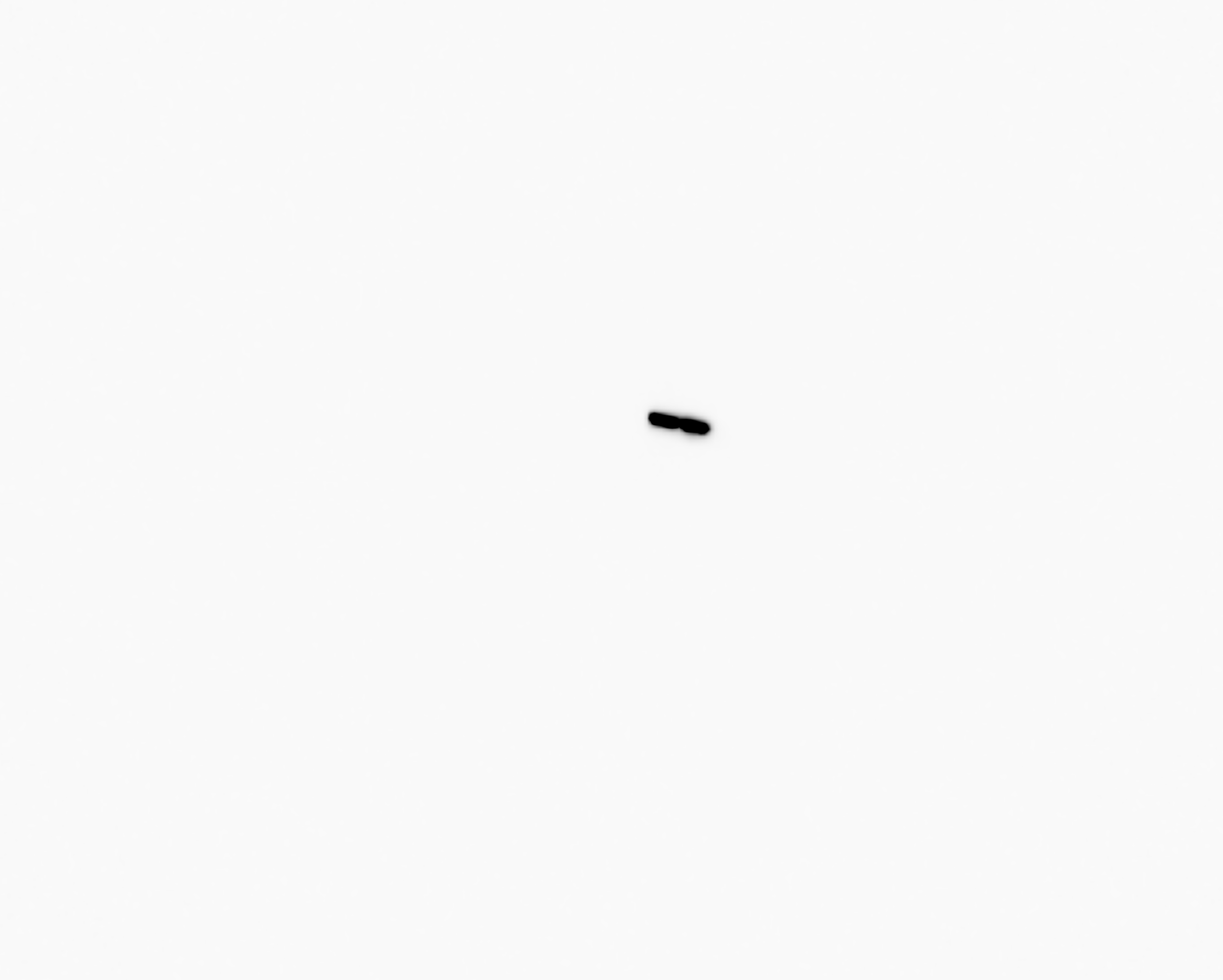

Supplement: Supplementary file 7 — Additional file 7. [file 12964_2024_1475_MOESM7_ESM.zip › Additional file 2/Figure 2J/KYSE-150/a┬-actin.tif]

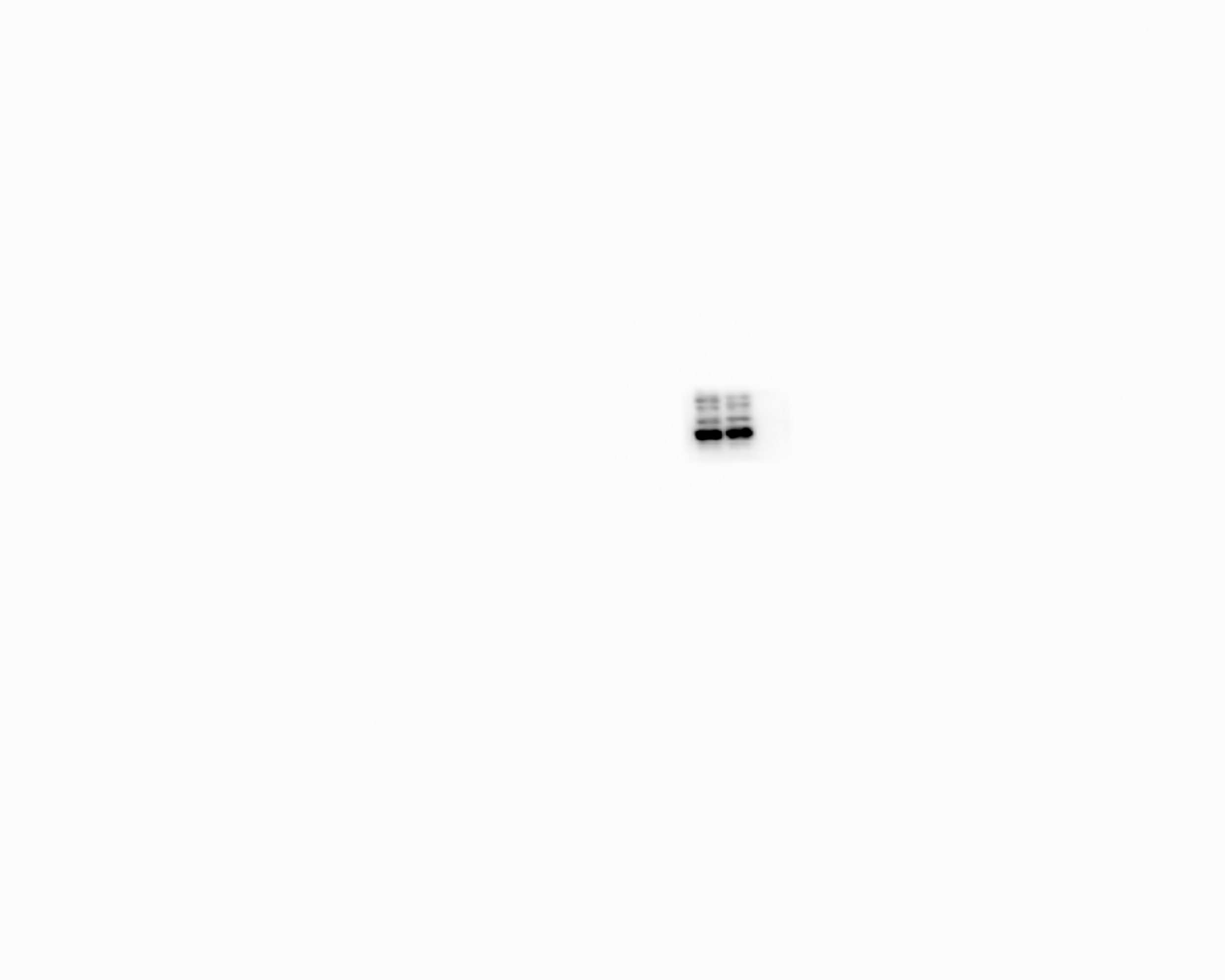

Supplement: Supplementary file 7 — Additional file 7. [file 12964_2024_1475_MOESM7_ESM.zip › Additional file 2/Figure 2J/KYSE-30/c-myc.tif]

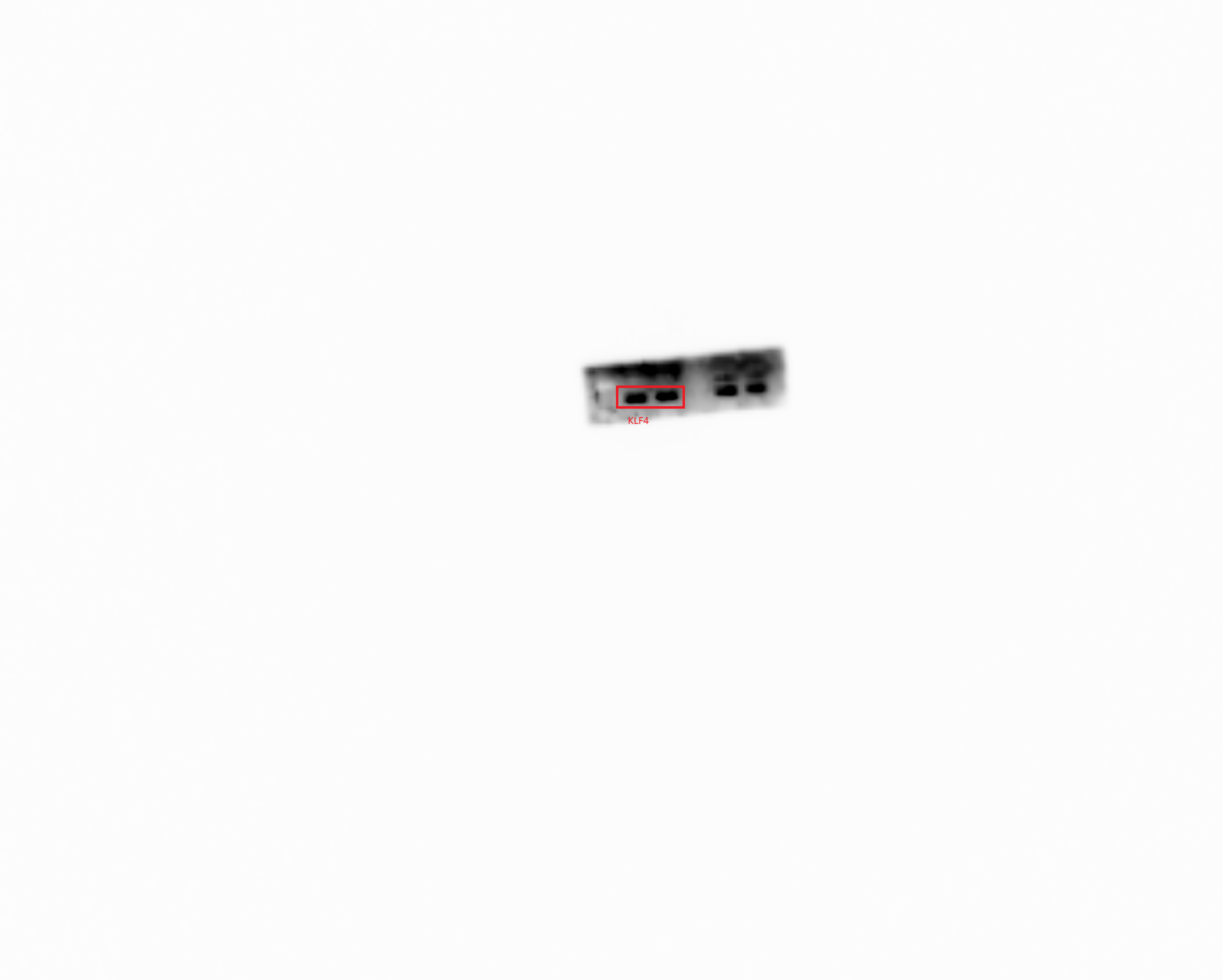

Supplement: Supplementary file 7 — Additional file 7. [file 12964_2024_1475_MOESM7_ESM.zip › Additional file 2/Figure 2J/KYSE-30/klf4.tif]

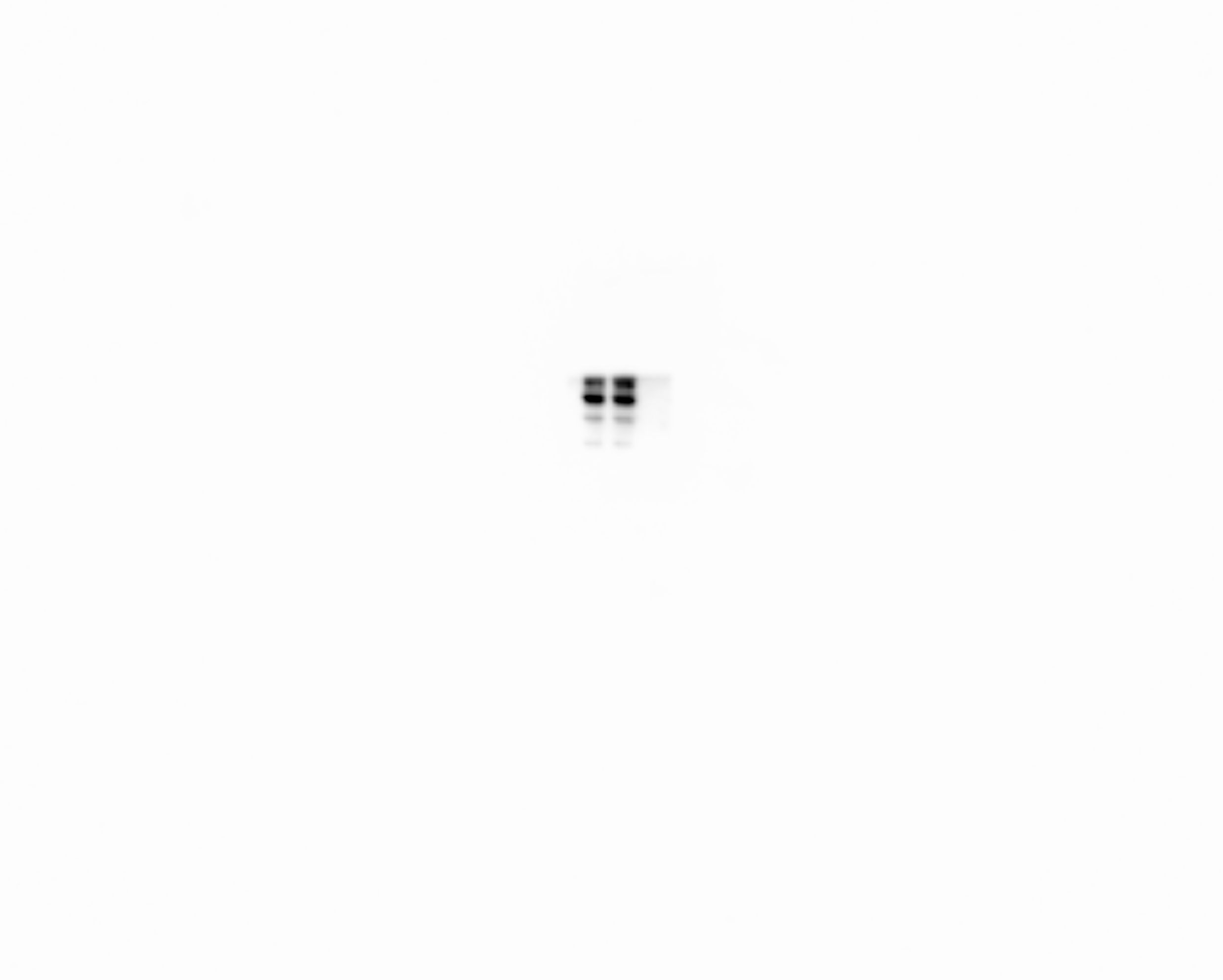

Supplement: Supplementary file 7 — Additional file 7. [file 12964_2024_1475_MOESM7_ESM.zip › Additional file 2/Figure 2J/KYSE-30/nanog.tif]

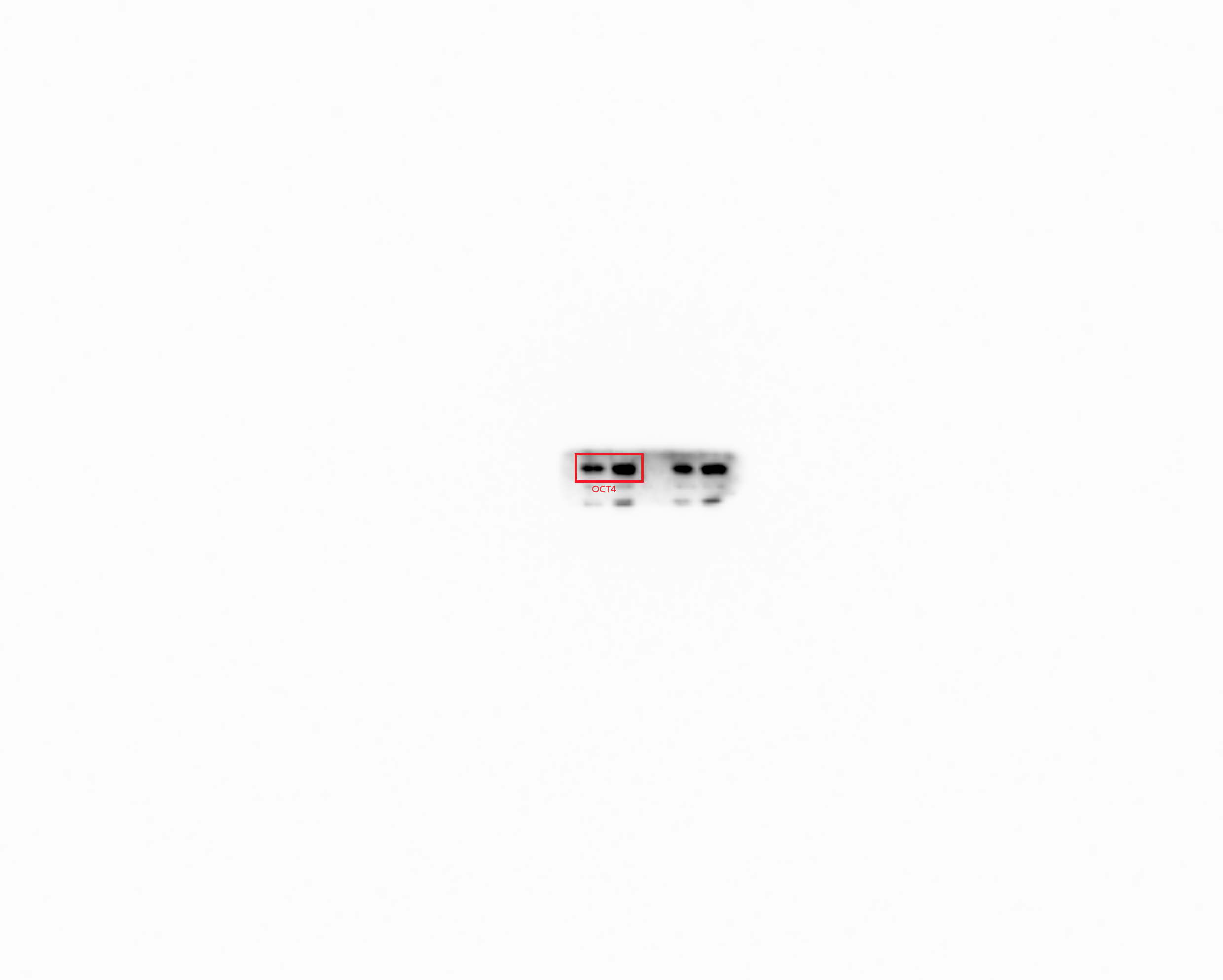

Supplement: Supplementary file 7 — Additional file 7. [file 12964_2024_1475_MOESM7_ESM.zip › Additional file 2/Figure 2J/KYSE-30/oct4.tif]

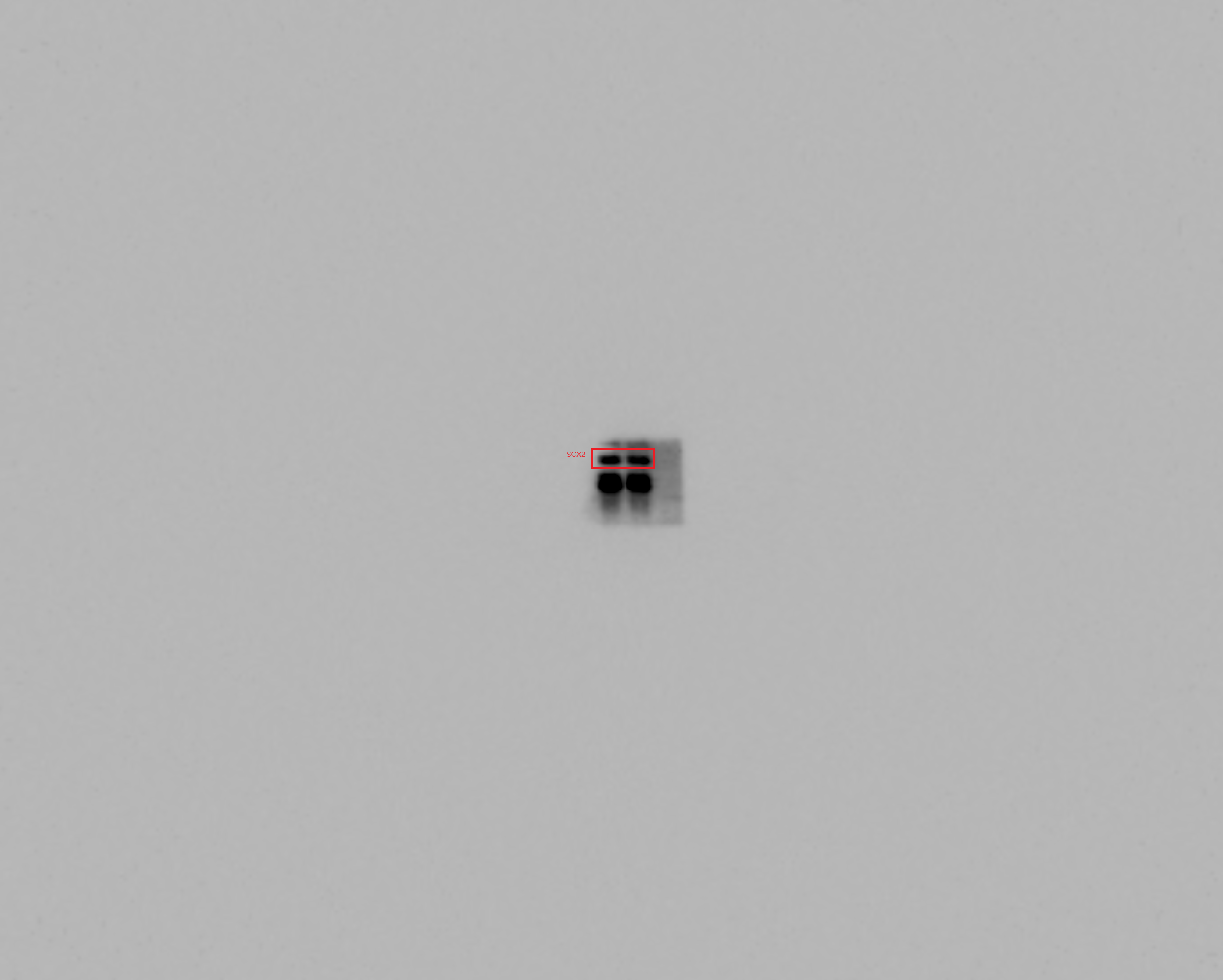

Supplement: Supplementary file 7 — Additional file 7. [file 12964_2024_1475_MOESM7_ESM.zip › Additional file 2/Figure 2J/KYSE-30/sox2.tif]

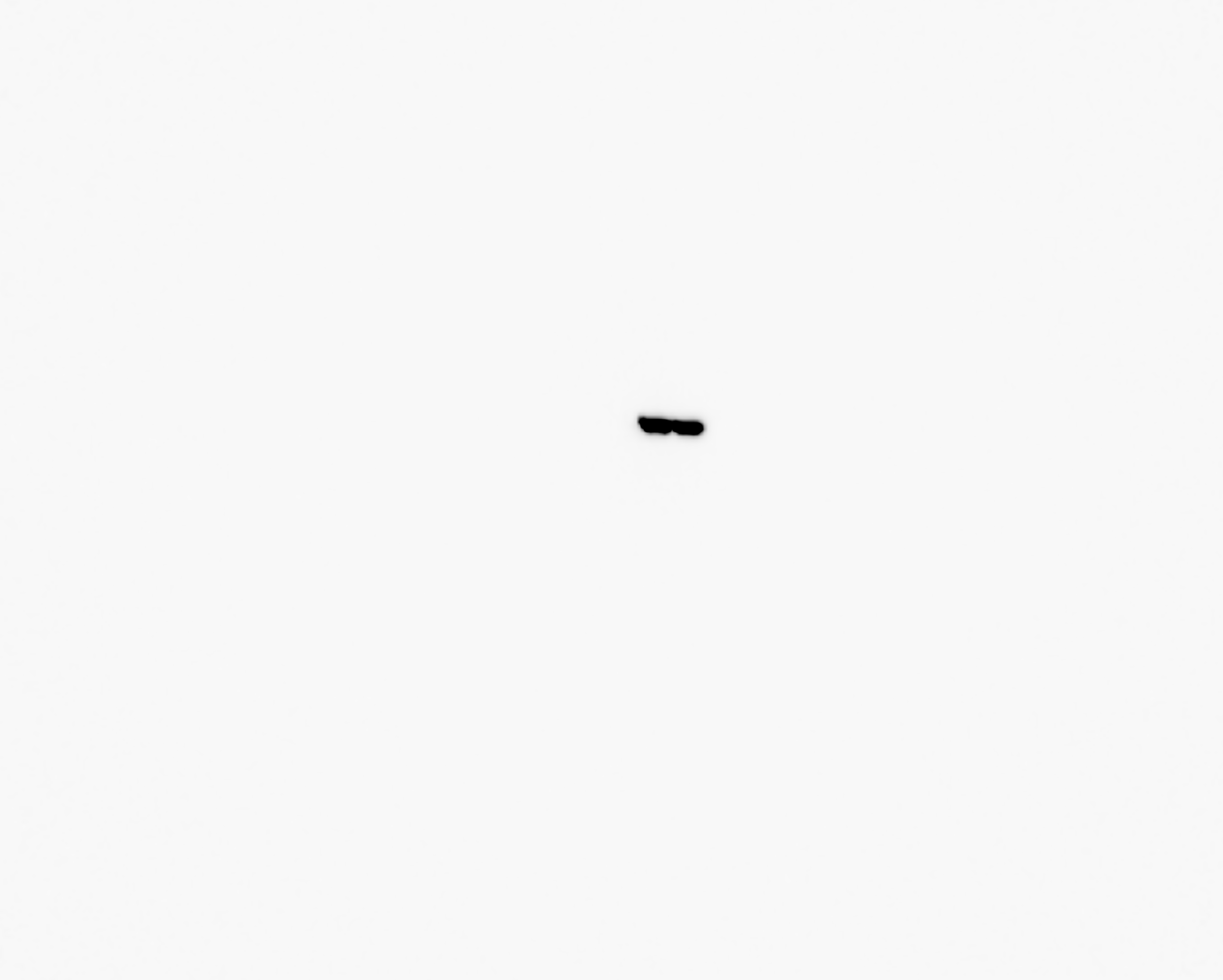

Supplement: Supplementary file 7 — Additional file 7. [file 12964_2024_1475_MOESM7_ESM.zip › Additional file 2/Figure 2J/KYSE-30/a┬-actin.tif]

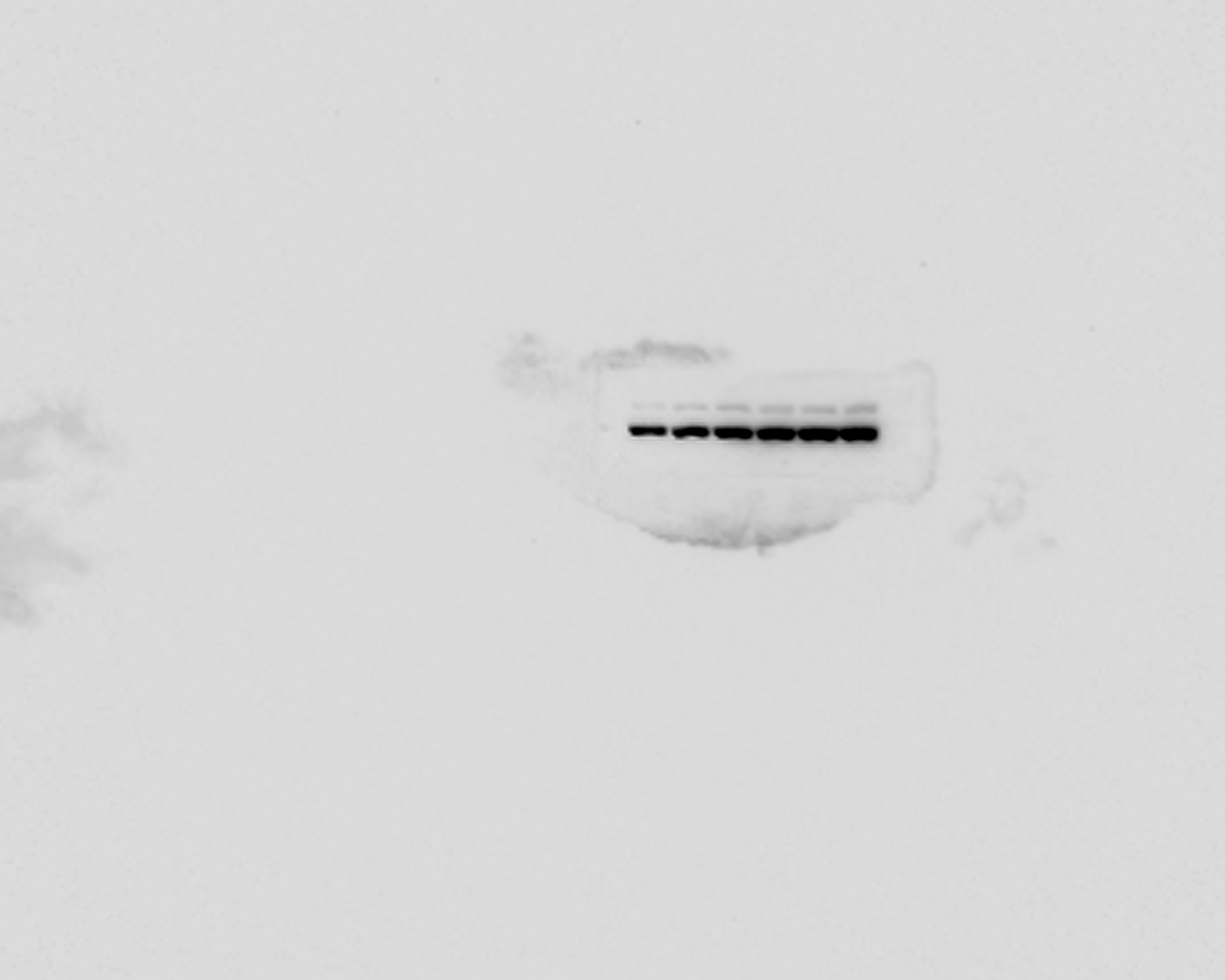

Supplement: Supplementary file 7 — Additional file 7. [file 12964_2024_1475_MOESM7_ESM.zip › Additional file 2/Figure 2M/oct4.tif]

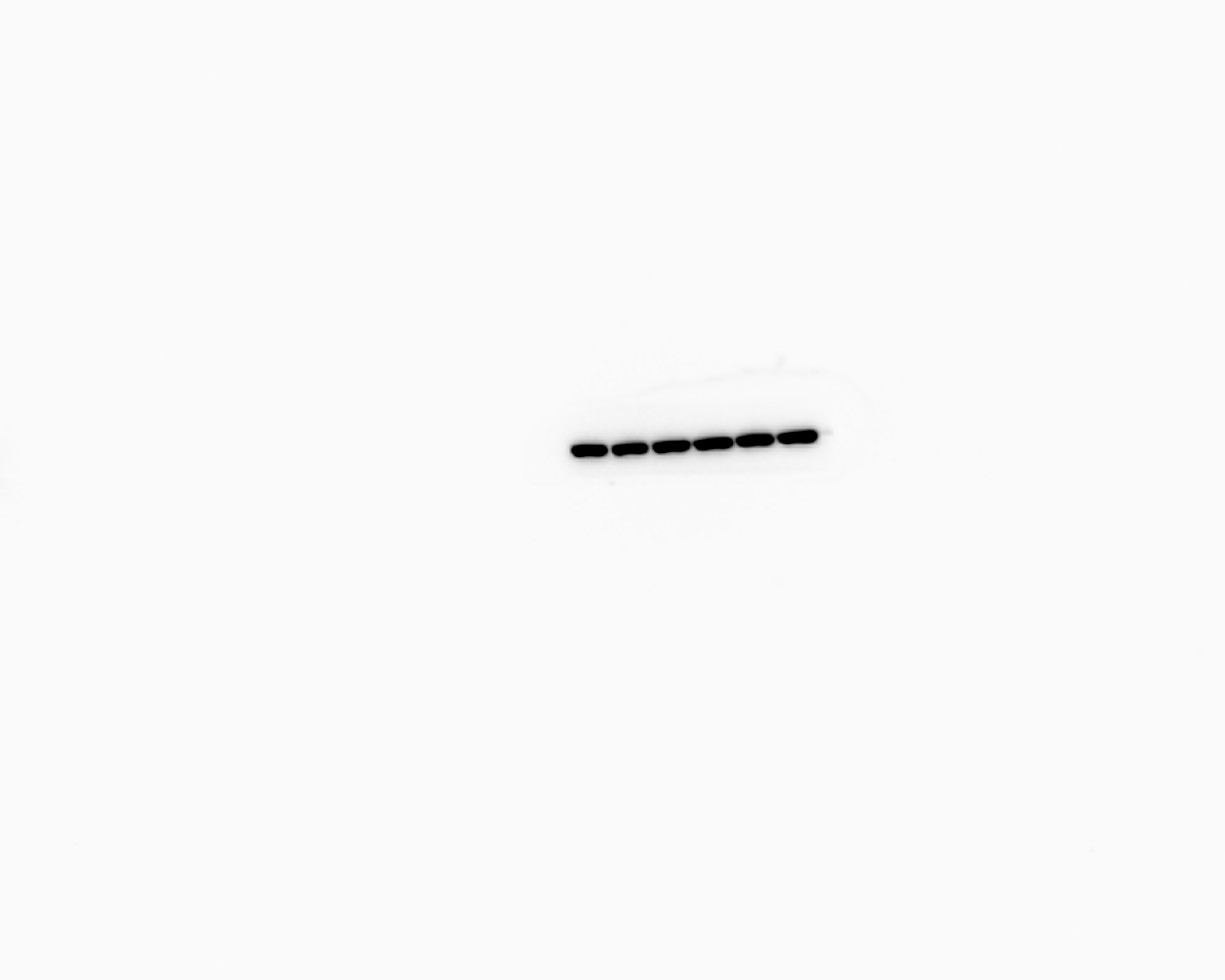

Supplement: Supplementary file 7 — Additional file 7. [file 12964_2024_1475_MOESM7_ESM.zip › Additional file 2/Figure 2M/a┬-actin.tif]

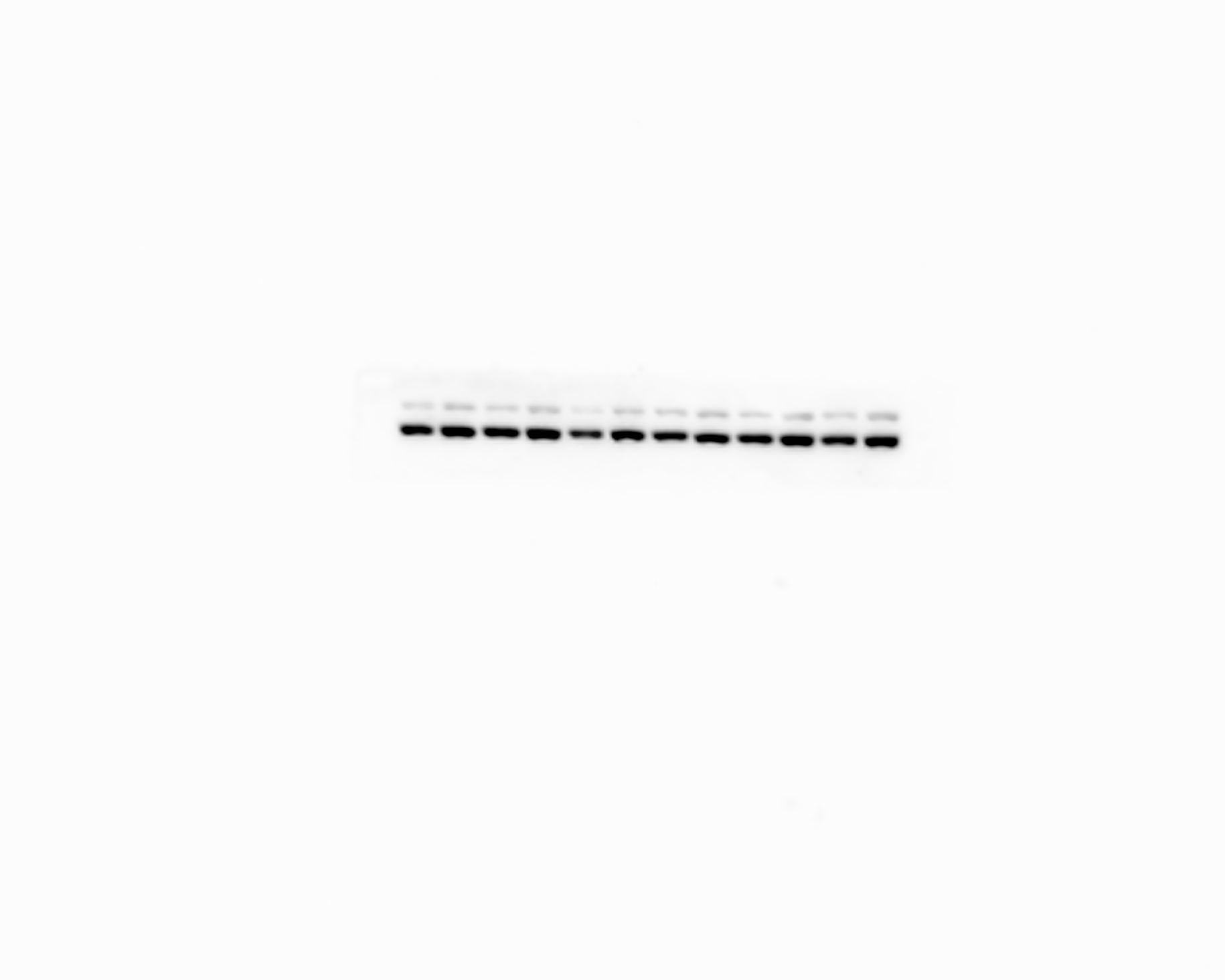

Supplement: Supplementary file 7 — Additional file 7. [file 12964_2024_1475_MOESM7_ESM.zip › Additional file 2/Figure 2N/oct4.tif]

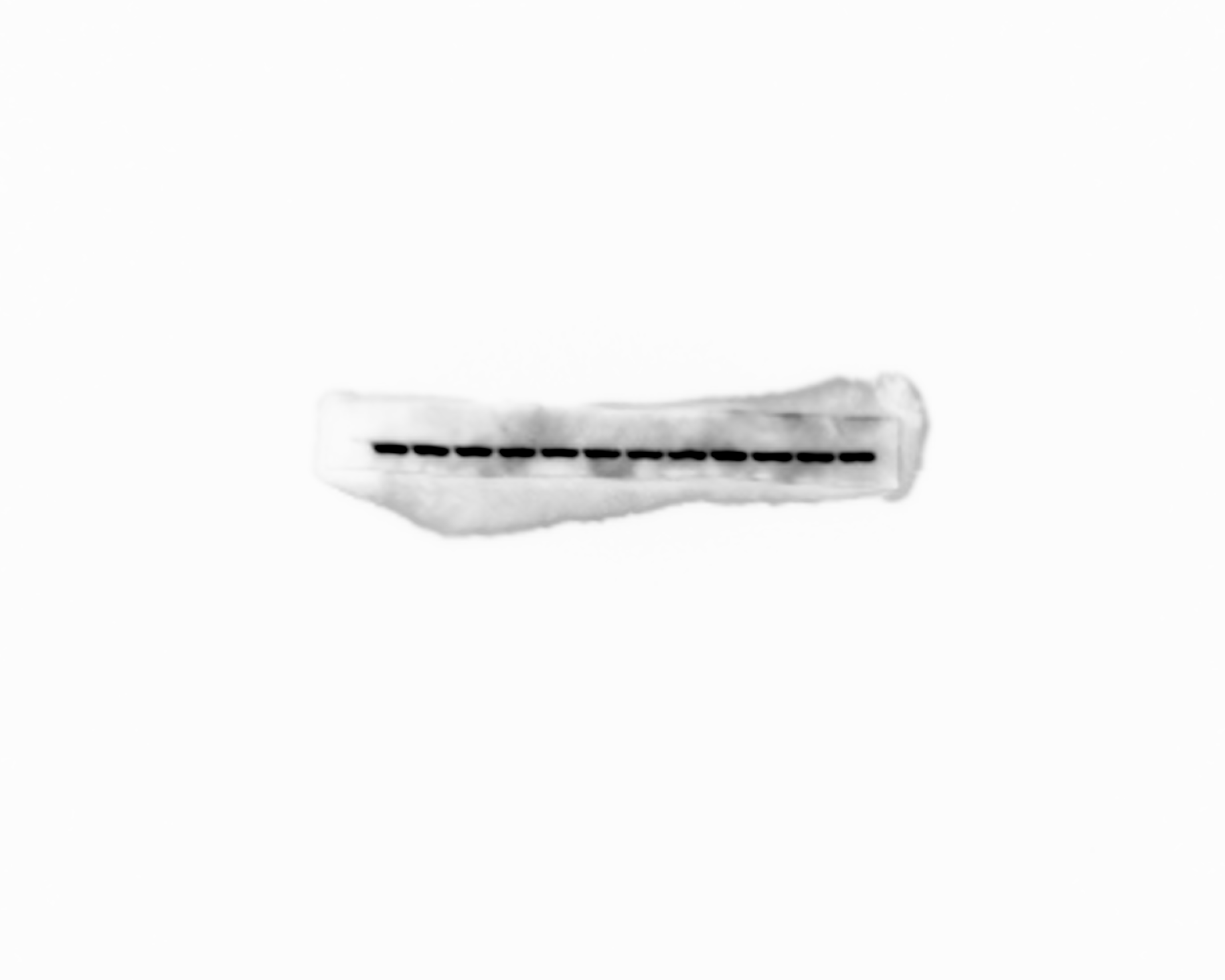

Supplement: Supplementary file 7 — Additional file 7. [file 12964_2024_1475_MOESM7_ESM.zip › Additional file 2/Figure 2N/a┬-actin.tif]

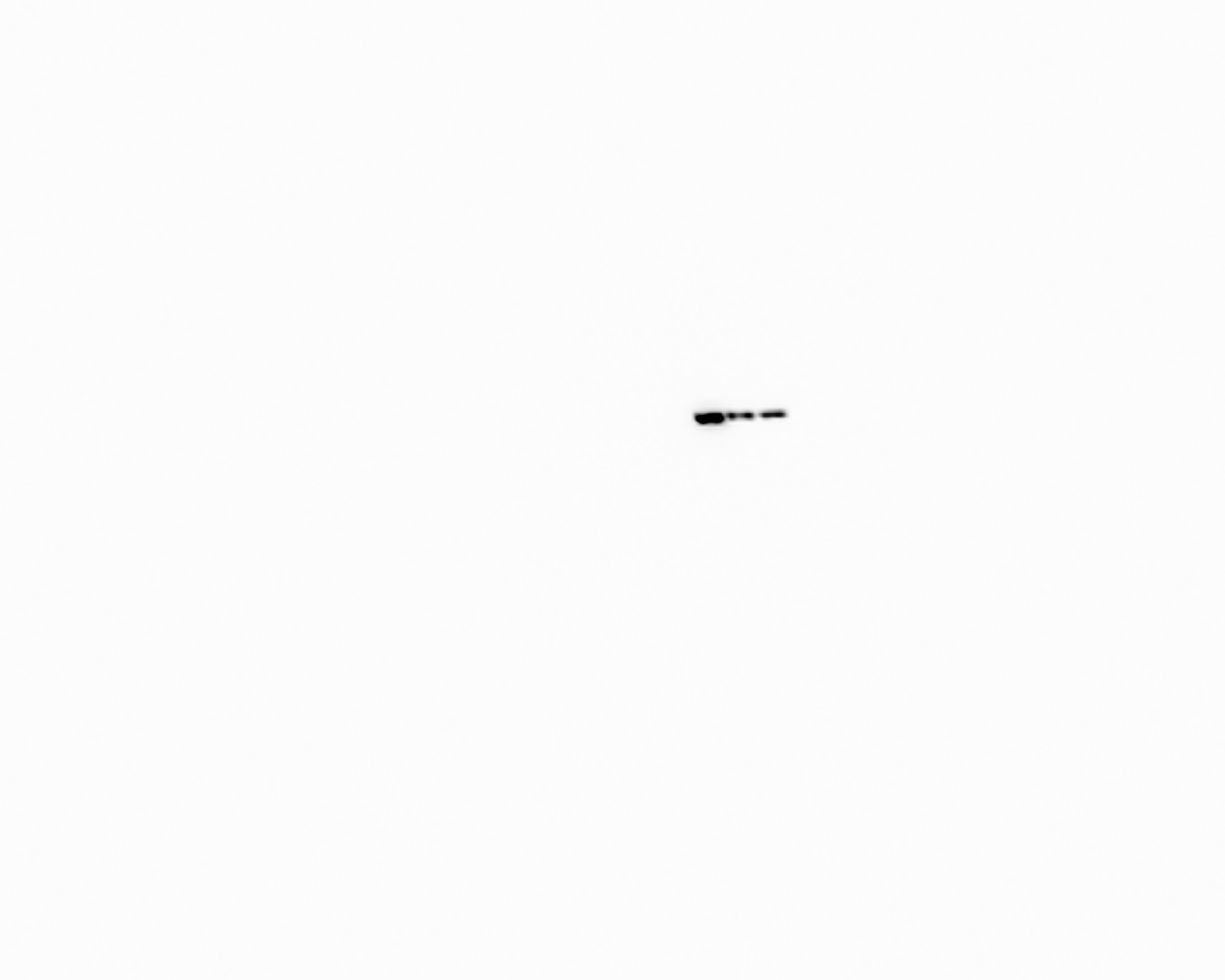

Supplement: Supplementary file 7 — Additional file 7. [file 12964_2024_1475_MOESM7_ESM.zip › Additional file 2/Figure 2O/Eca-109/oct4.tif]

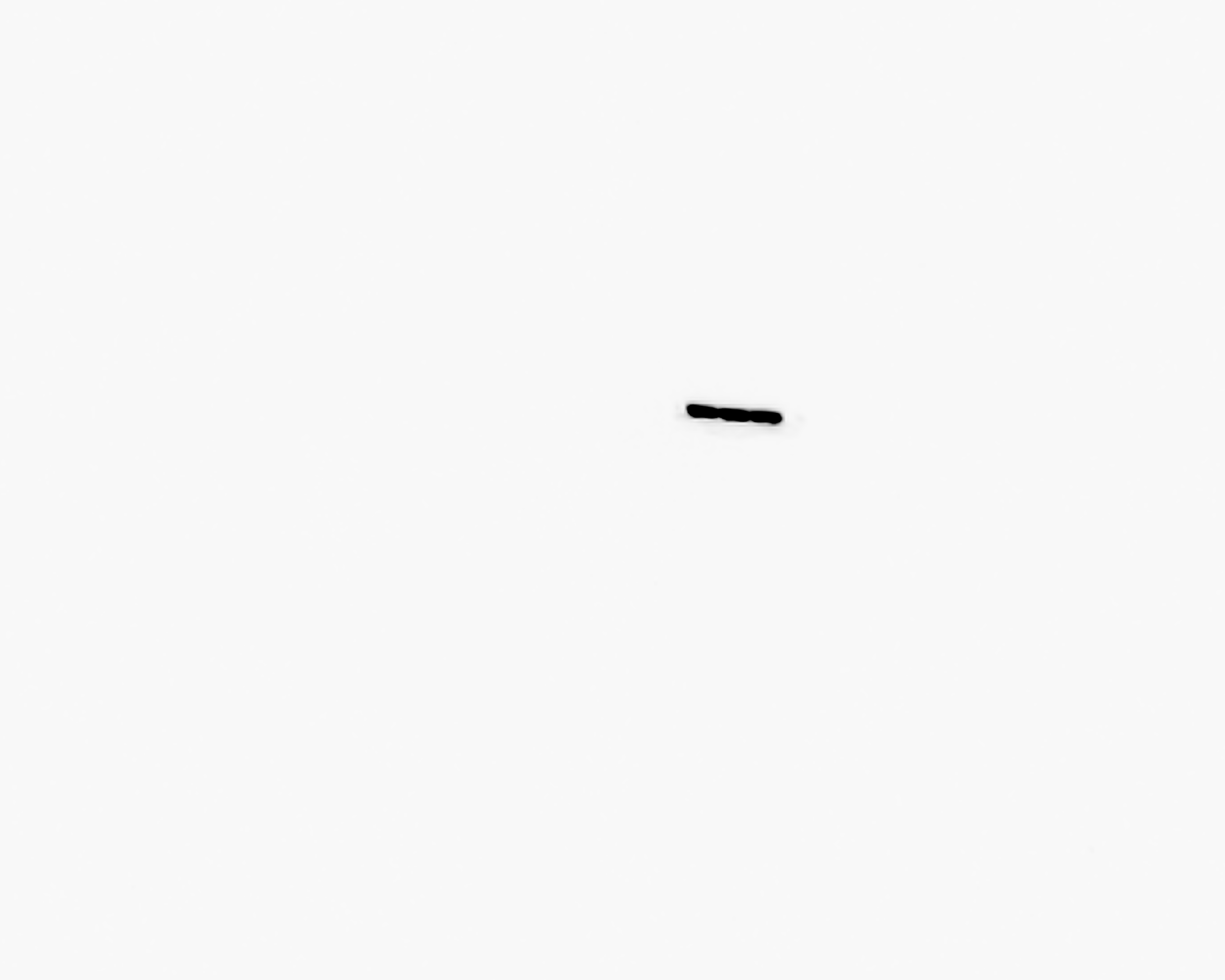

Supplement: Supplementary file 7 — Additional file 7. [file 12964_2024_1475_MOESM7_ESM.zip › Additional file 2/Figure 2O/Eca-109/a┬-actin.tif]

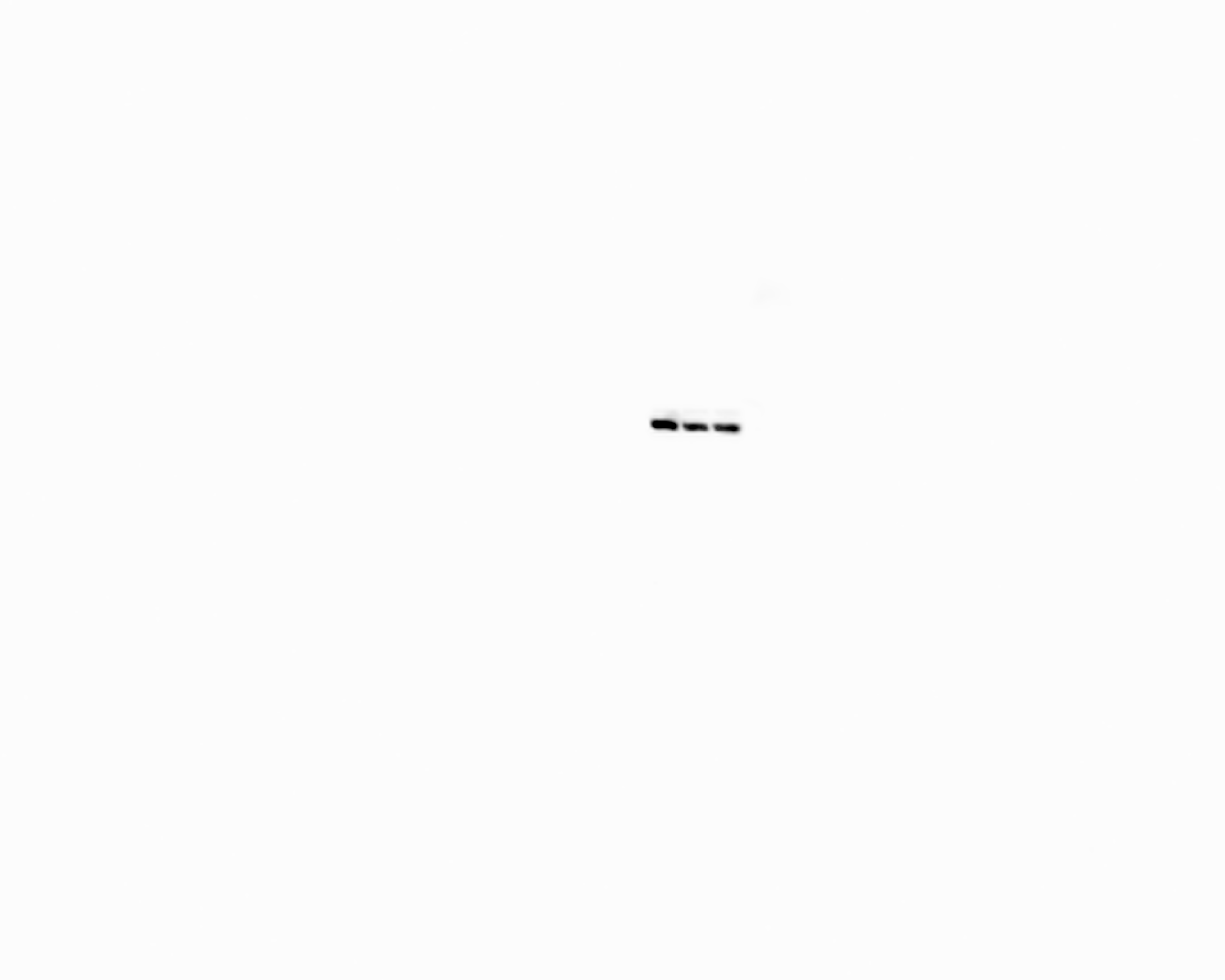

Supplement: Supplementary file 7 — Additional file 7. [file 12964_2024_1475_MOESM7_ESM.zip › Additional file 2/Figure 2O/KYSE-150/oct4.tif]

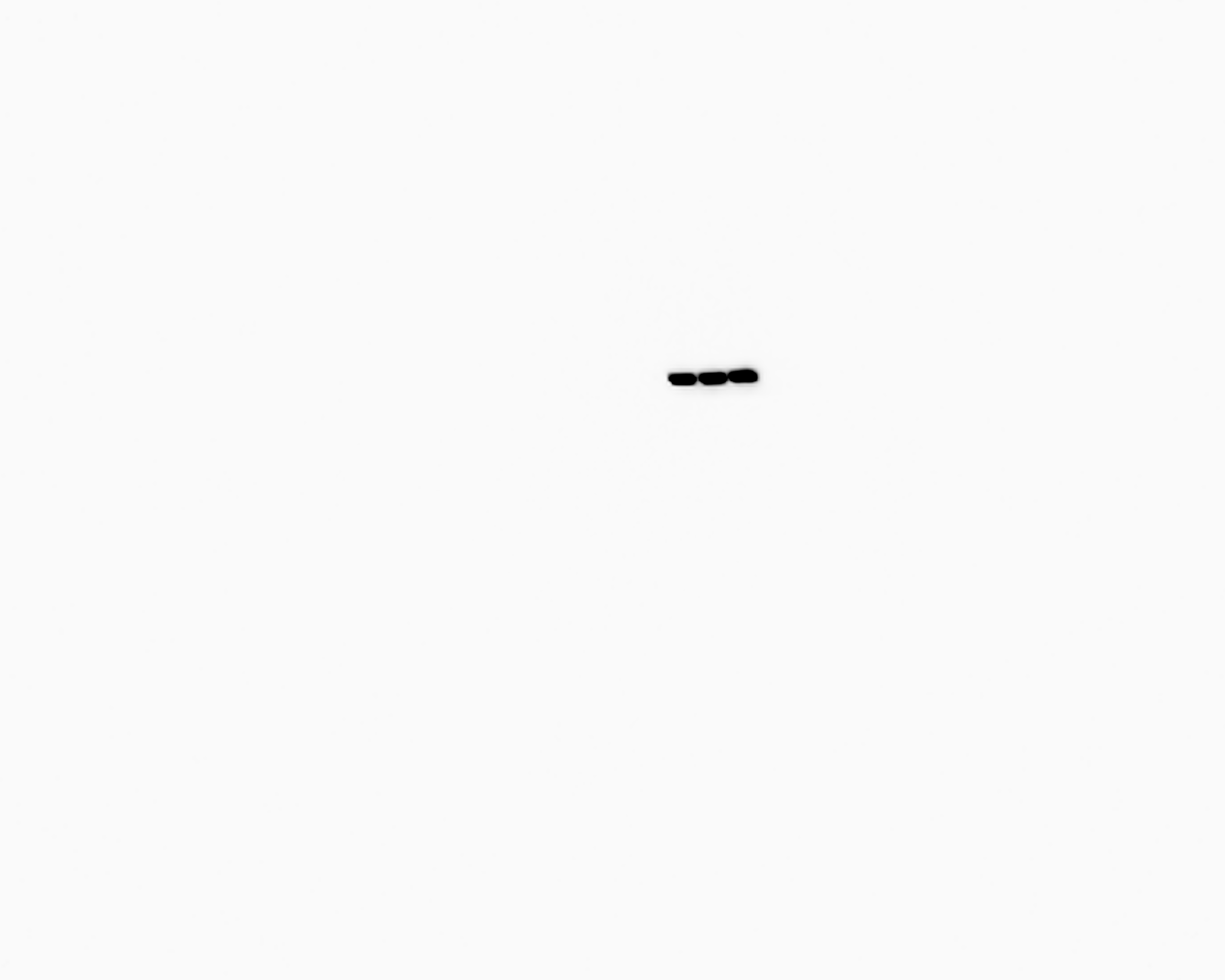

Supplement: Supplementary file 7 — Additional file 7. [file 12964_2024_1475_MOESM7_ESM.zip › Additional file 2/Figure 2O/KYSE-150/a┬-actin.tif]

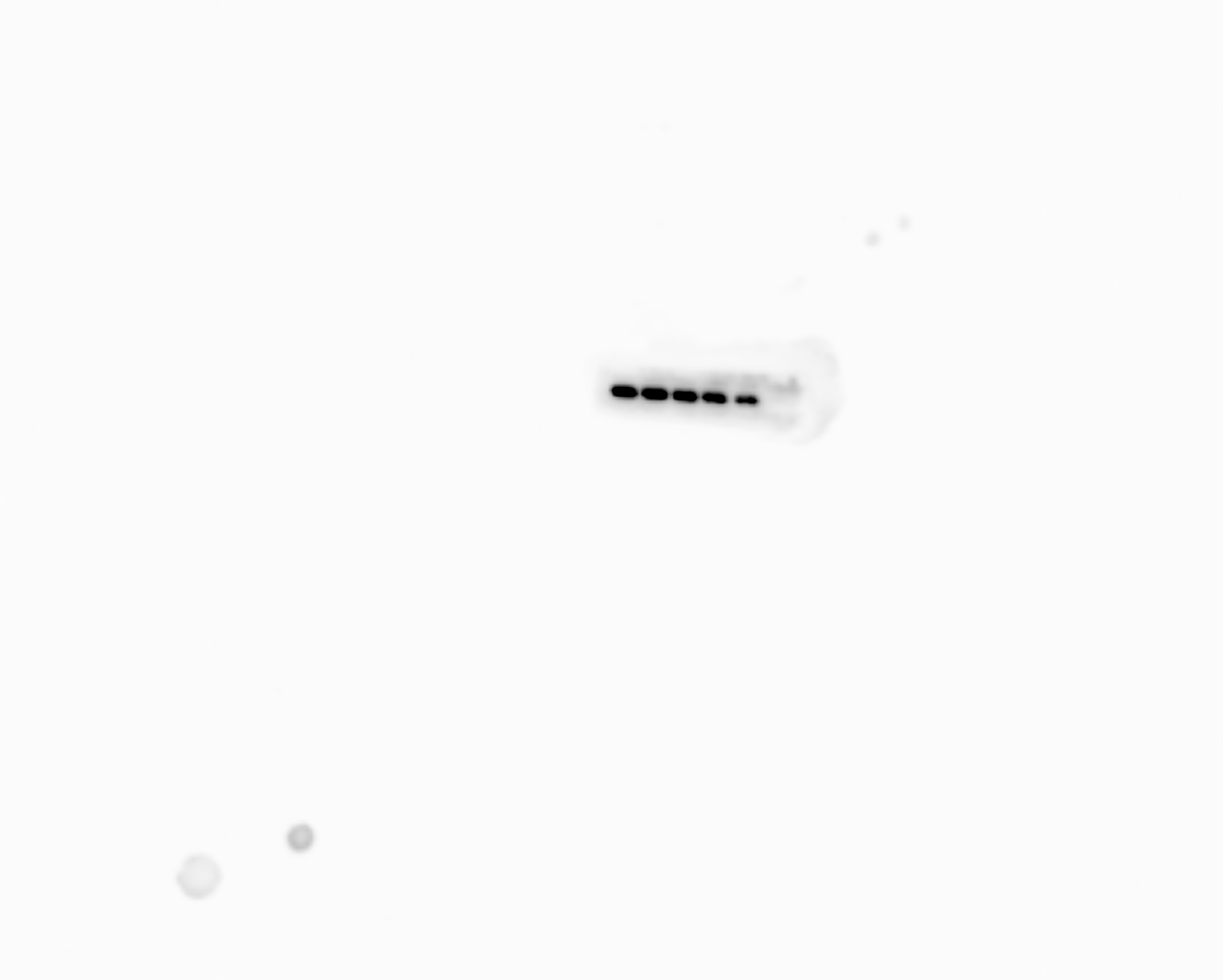

Supplement: Supplementary file 7 — Additional file 7. [file 12964_2024_1475_MOESM7_ESM.zip › Additional file 2/Figure 3C/Eca-109/nc oct4.tif]

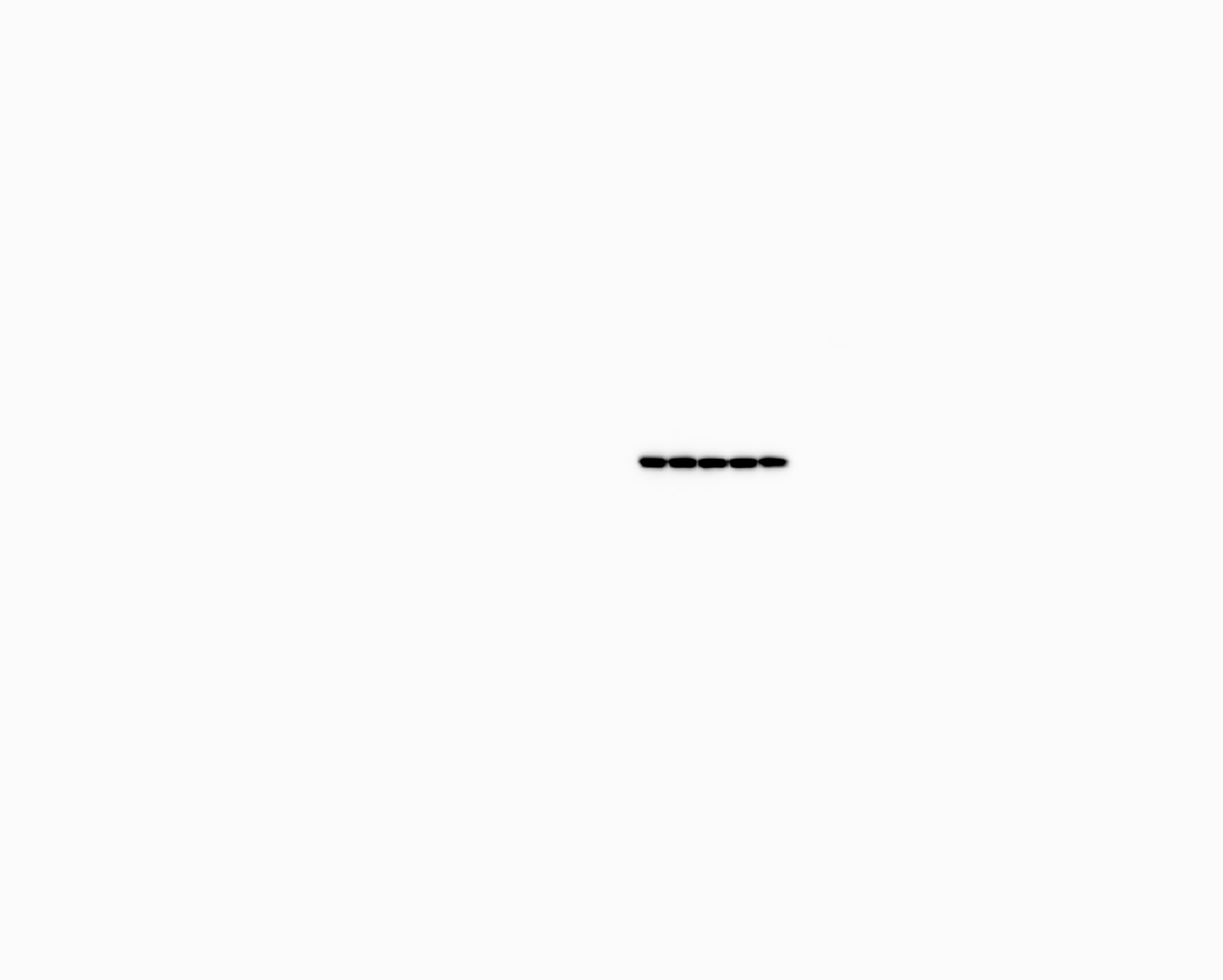

Supplement: Supplementary file 7 — Additional file 7. [file 12964_2024_1475_MOESM7_ESM.zip › Additional file 2/Figure 3C/Eca-109/nc a┬-actin.tif]

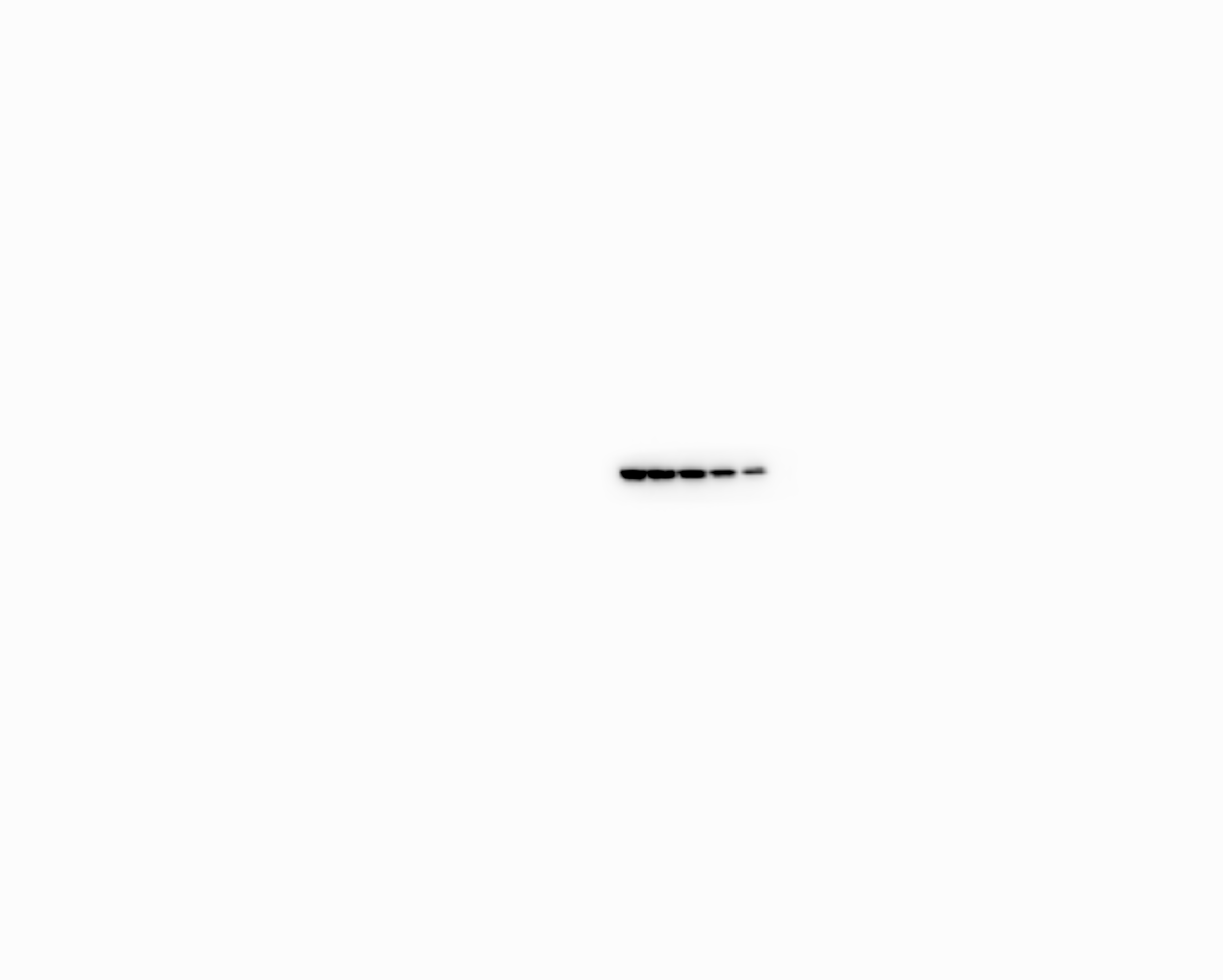

Supplement: Supplementary file 7 — Additional file 7. [file 12964_2024_1475_MOESM7_ESM.zip › Additional file 2/Figure 3C/Eca-109/siPDIA3P1 oct4.tif]

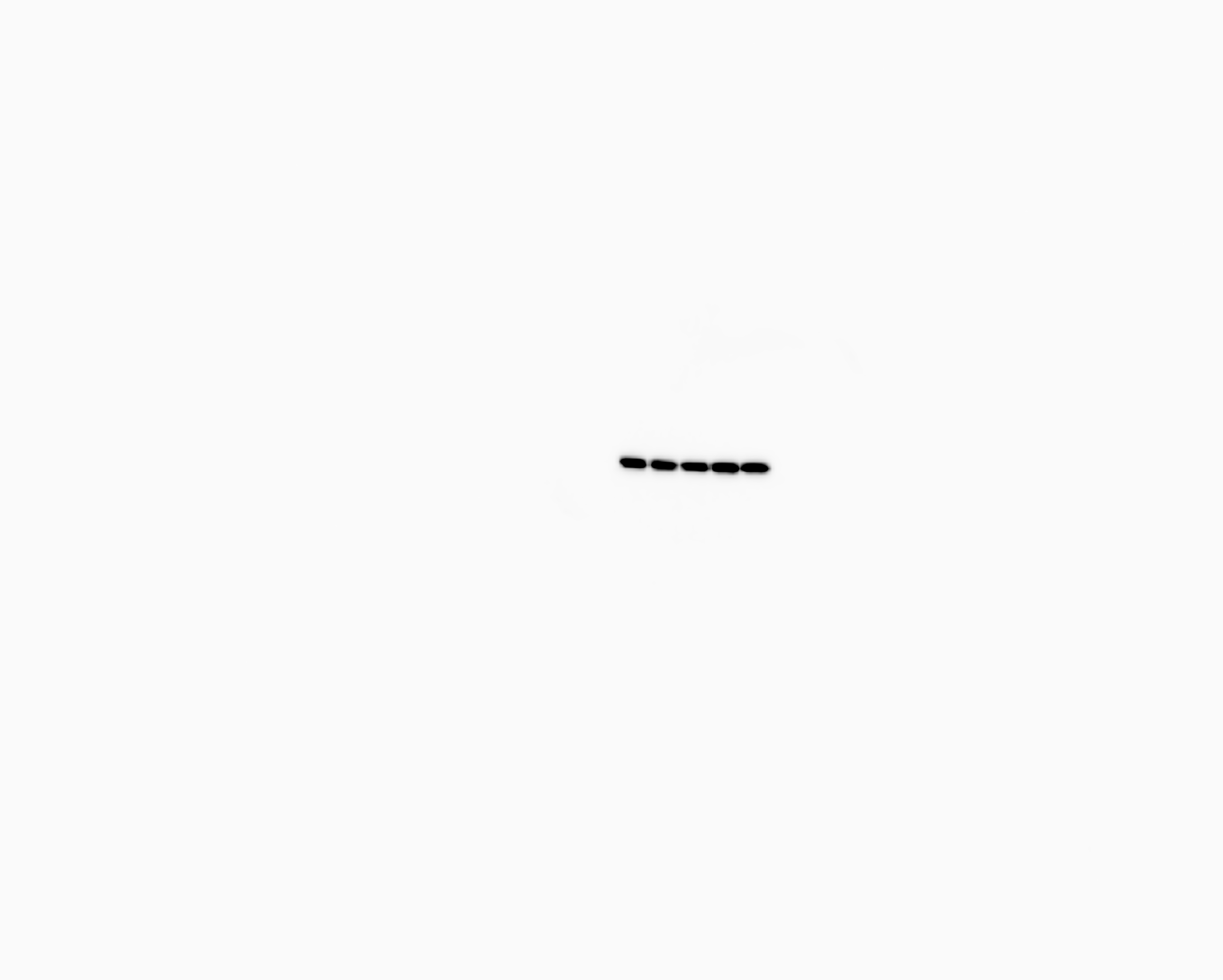

Supplement: Supplementary file 7 — Additional file 7. [file 12964_2024_1475_MOESM7_ESM.zip › Additional file 2/Figure 3C/Eca-109/siPDIA3P1 a┬-actin.tif]

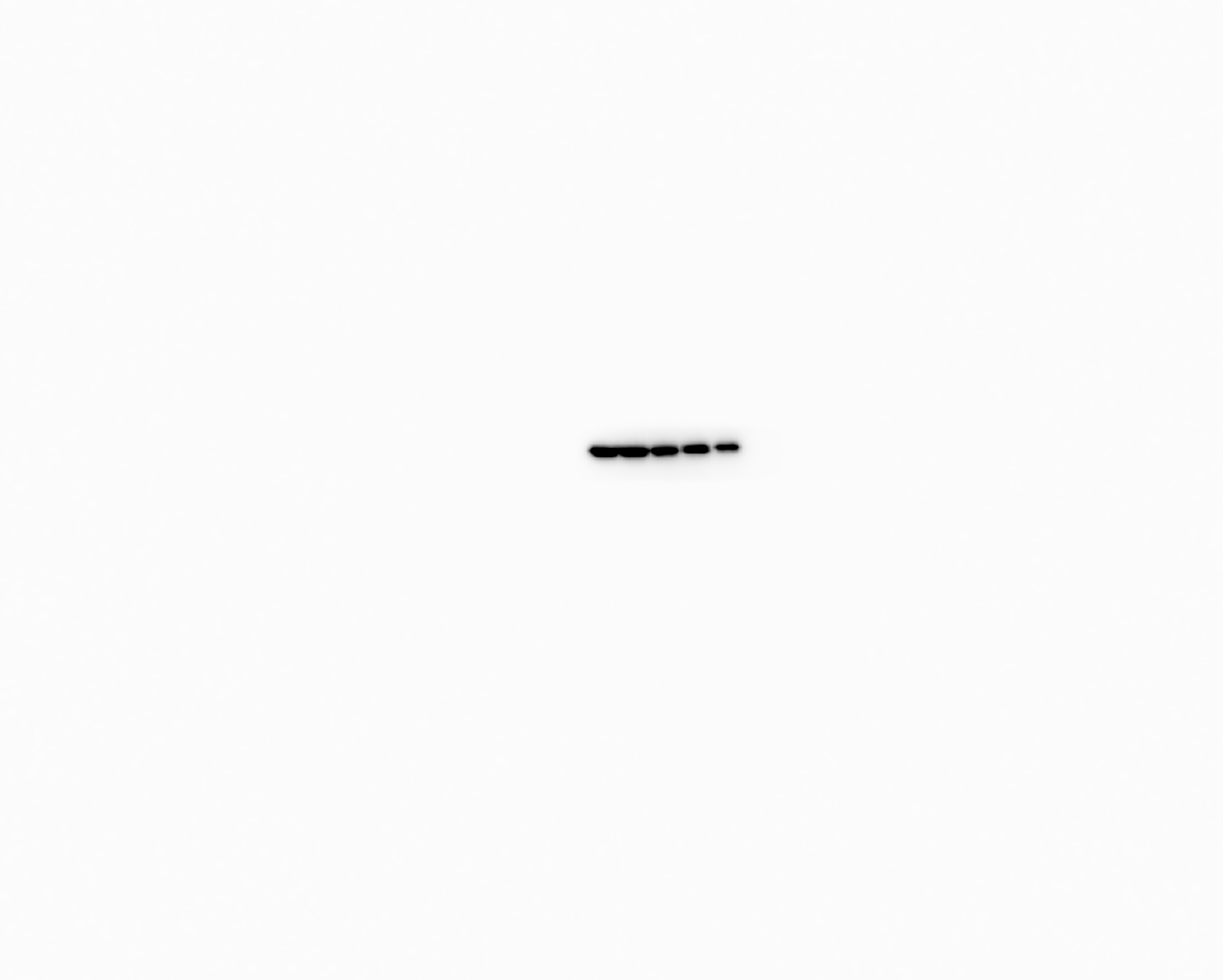

Supplement: Supplementary file 7 — Additional file 7. [file 12964_2024_1475_MOESM7_ESM.zip › Additional file 2/Figure 3C/TE-1/nc oct4.tif]

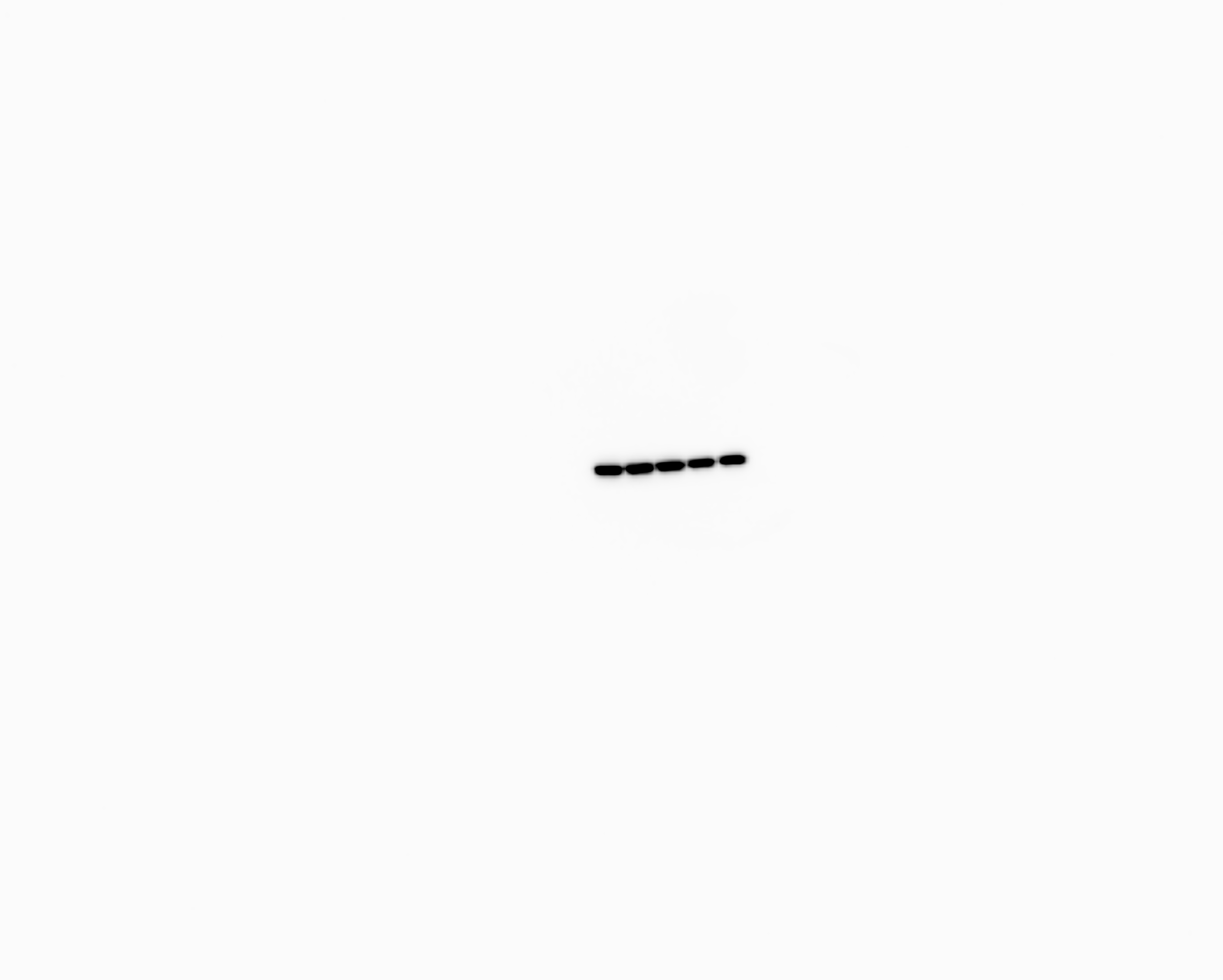

Supplement: Supplementary file 7 — Additional file 7. [file 12964_2024_1475_MOESM7_ESM.zip › Additional file 2/Figure 3C/TE-1/nc a┬-actin.tif]

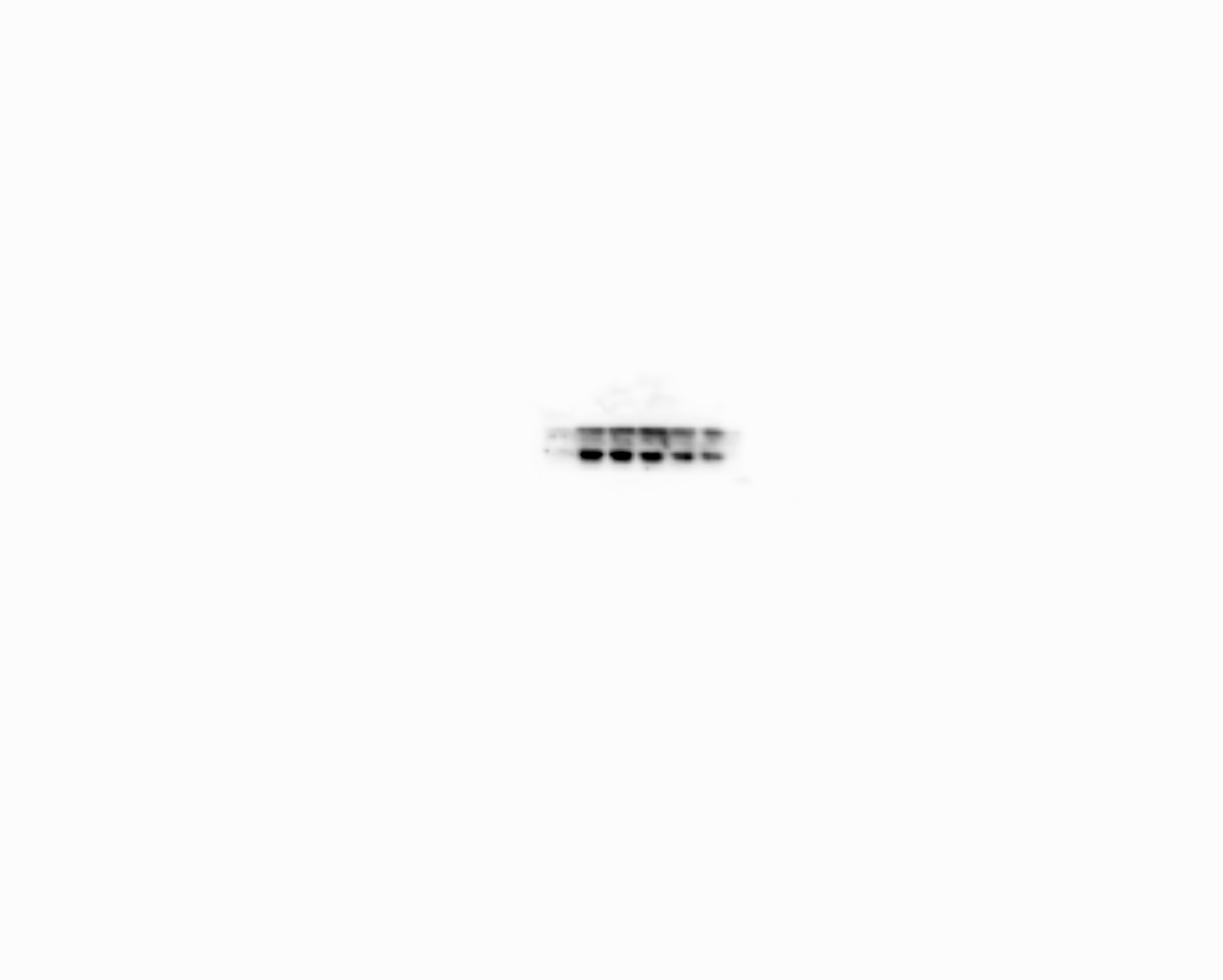

Supplement: Supplementary file 7 — Additional file 7. [file 12964_2024_1475_MOESM7_ESM.zip › Additional file 2/Figure 3C/TE-1/siPDIA3P1 oct4.tif]

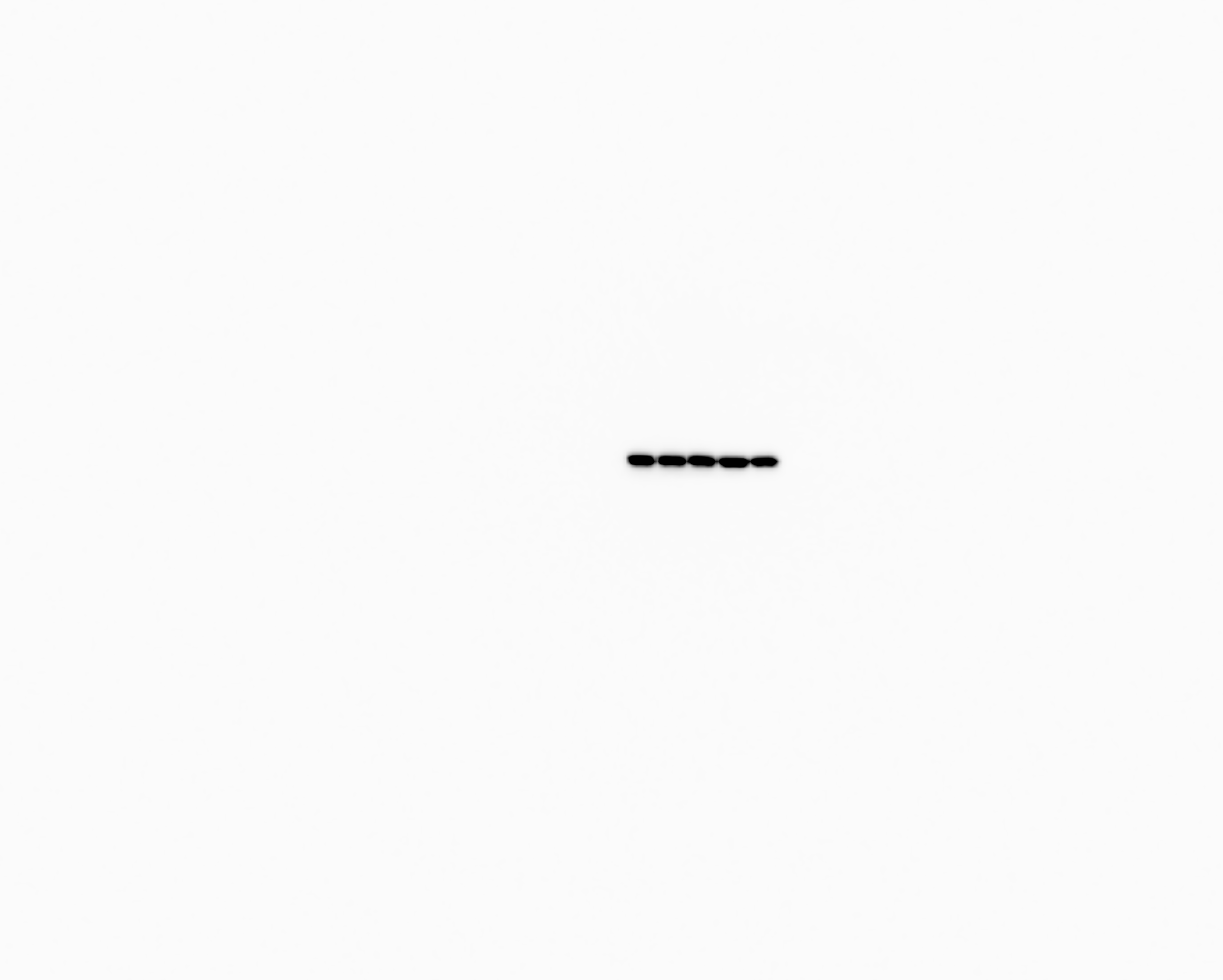

Supplement: Supplementary file 7 — Additional file 7. [file 12964_2024_1475_MOESM7_ESM.zip › Additional file 2/Figure 3C/TE-1/siPDIA3P1 a┬-actin.tif]

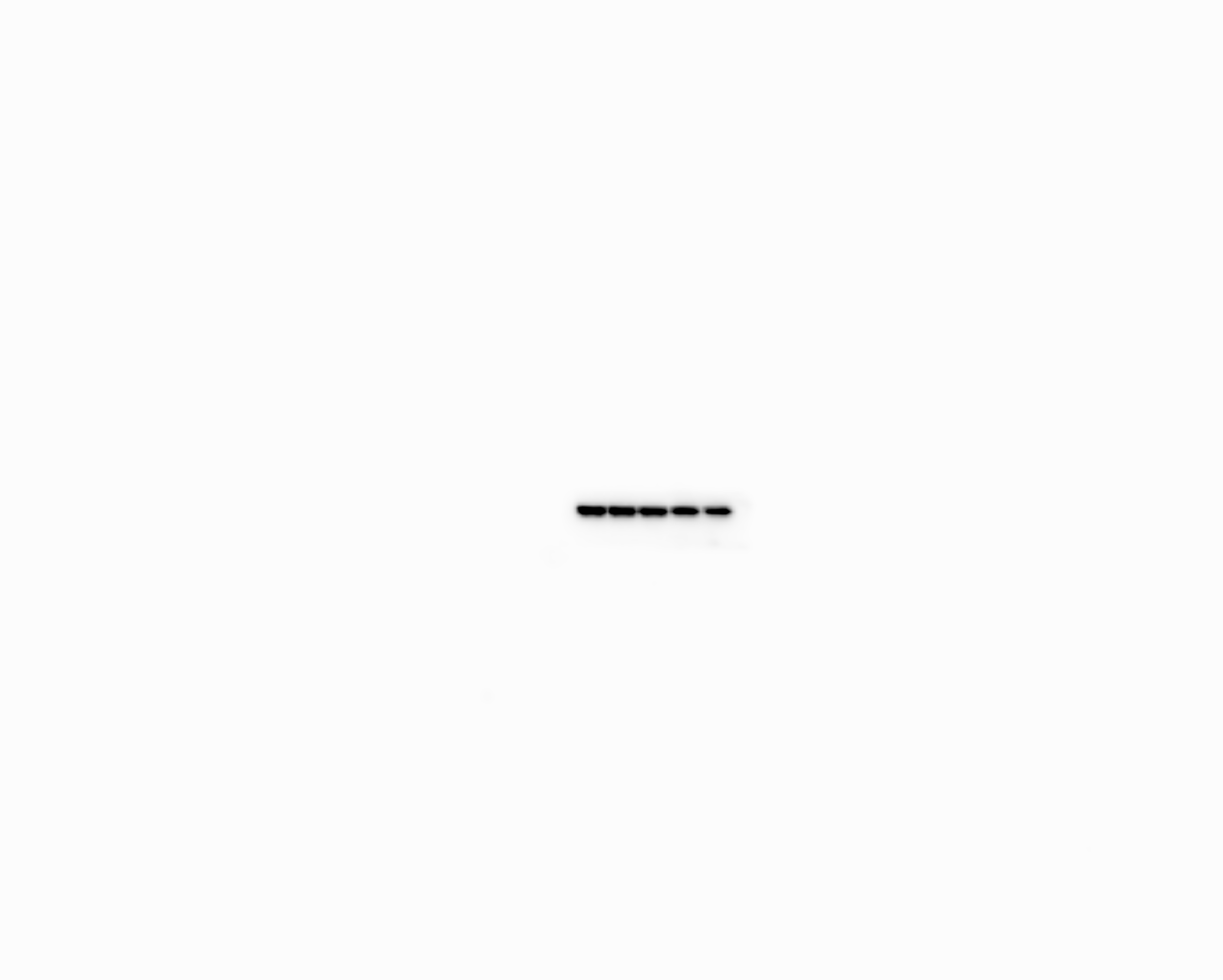

Supplement: Supplementary file 7 — Additional file 7. [file 12964_2024_1475_MOESM7_ESM.zip › Additional file 2/Figure 3D/KYSE-150/oePDIA3P1 oct4.tif]

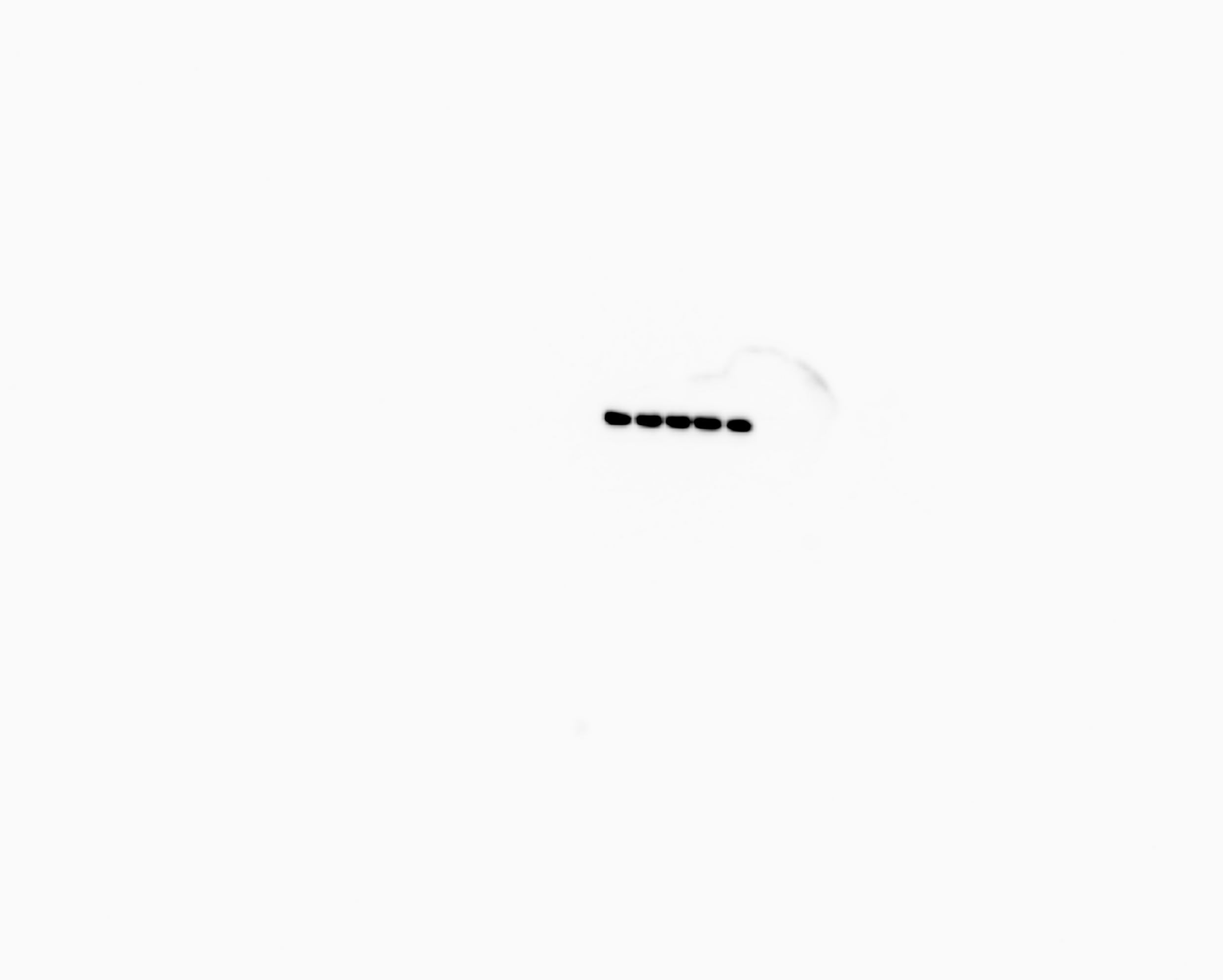

Supplement: Supplementary file 7 — Additional file 7. [file 12964_2024_1475_MOESM7_ESM.zip › Additional file 2/Figure 3D/KYSE-150/oePDIA3P1 a┬-actin.tif]

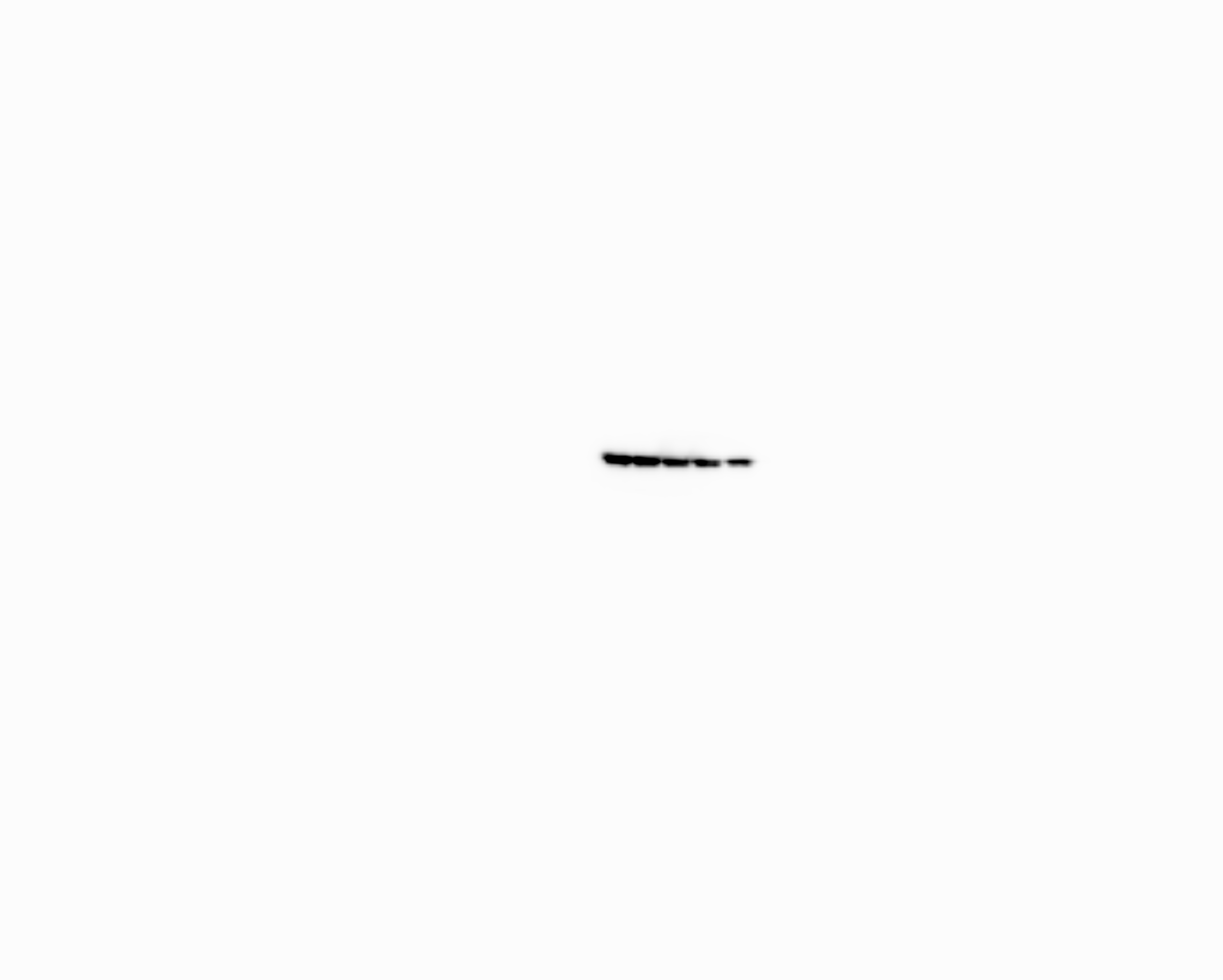

Supplement: Supplementary file 7 — Additional file 7. [file 12964_2024_1475_MOESM7_ESM.zip › Additional file 2/Figure 3D/KYSE-150/pc oct4.tif]

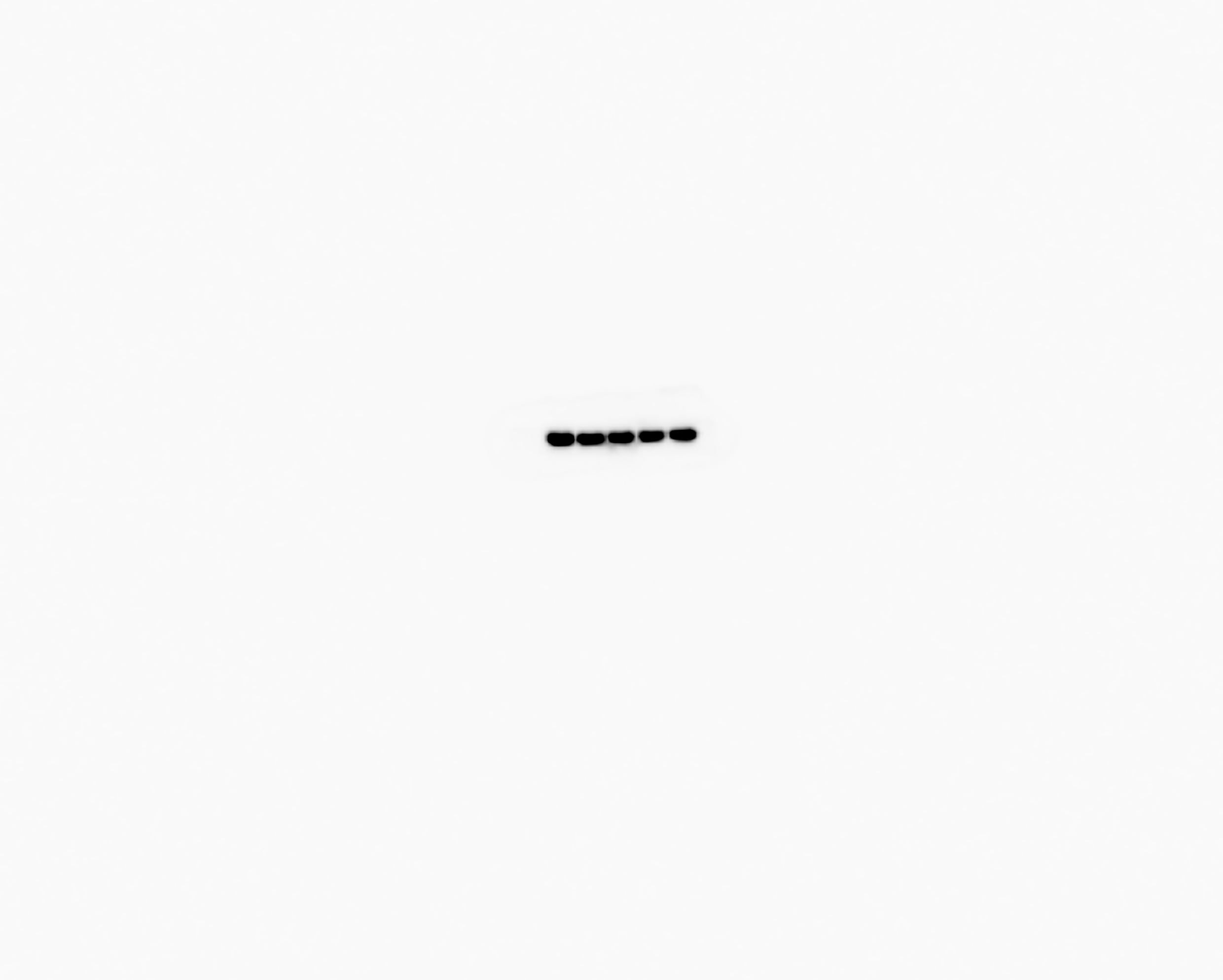

Supplement: Supplementary file 7 — Additional file 7. [file 12964_2024_1475_MOESM7_ESM.zip › Additional file 2/Figure 3D/KYSE-150/pc a┬-actin.tif]

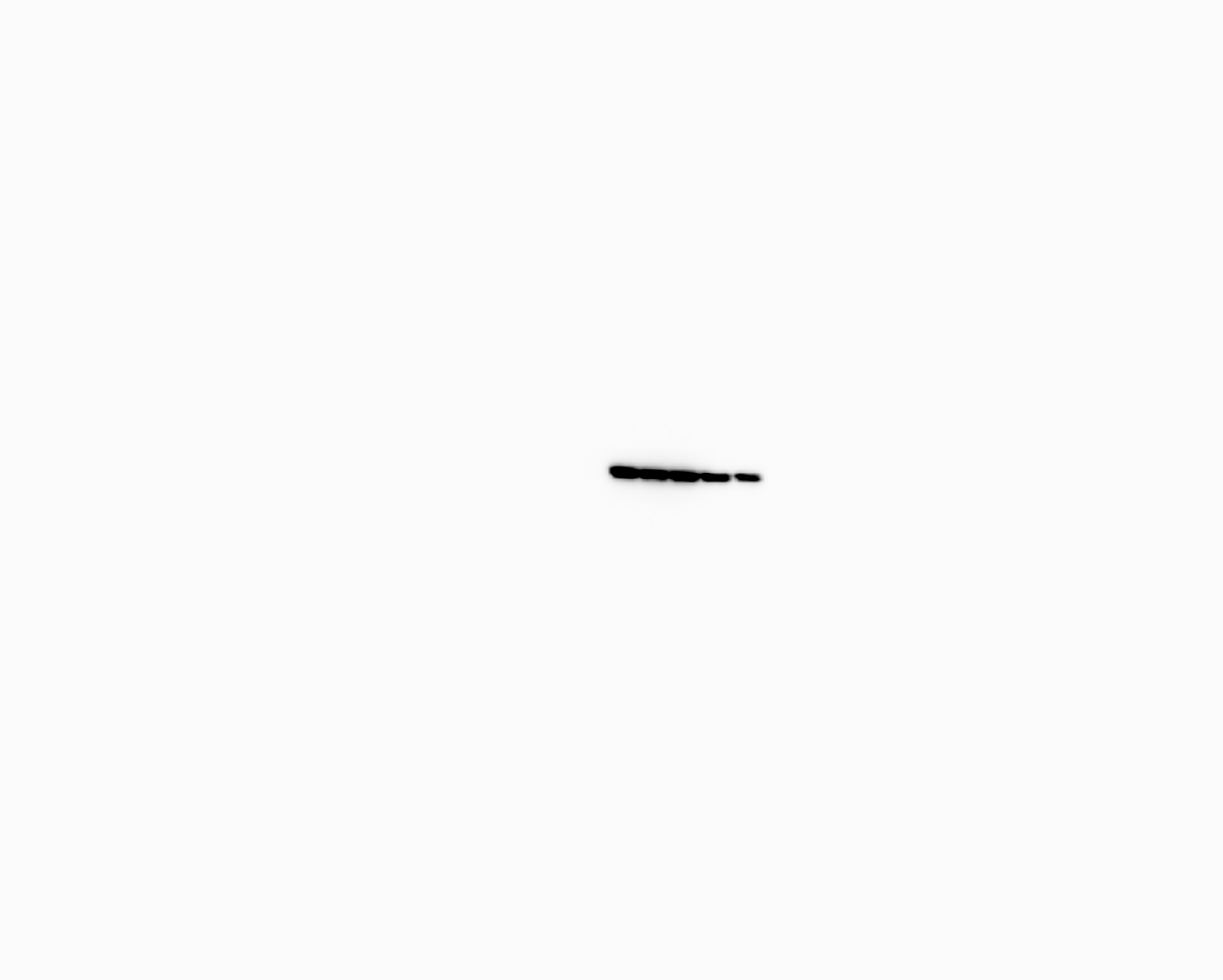

Supplement: Supplementary file 7 — Additional file 7. [file 12964_2024_1475_MOESM7_ESM.zip › Additional file 2/Figure 3D/KYSE-30/oePDIA3P1 oct4.tif]

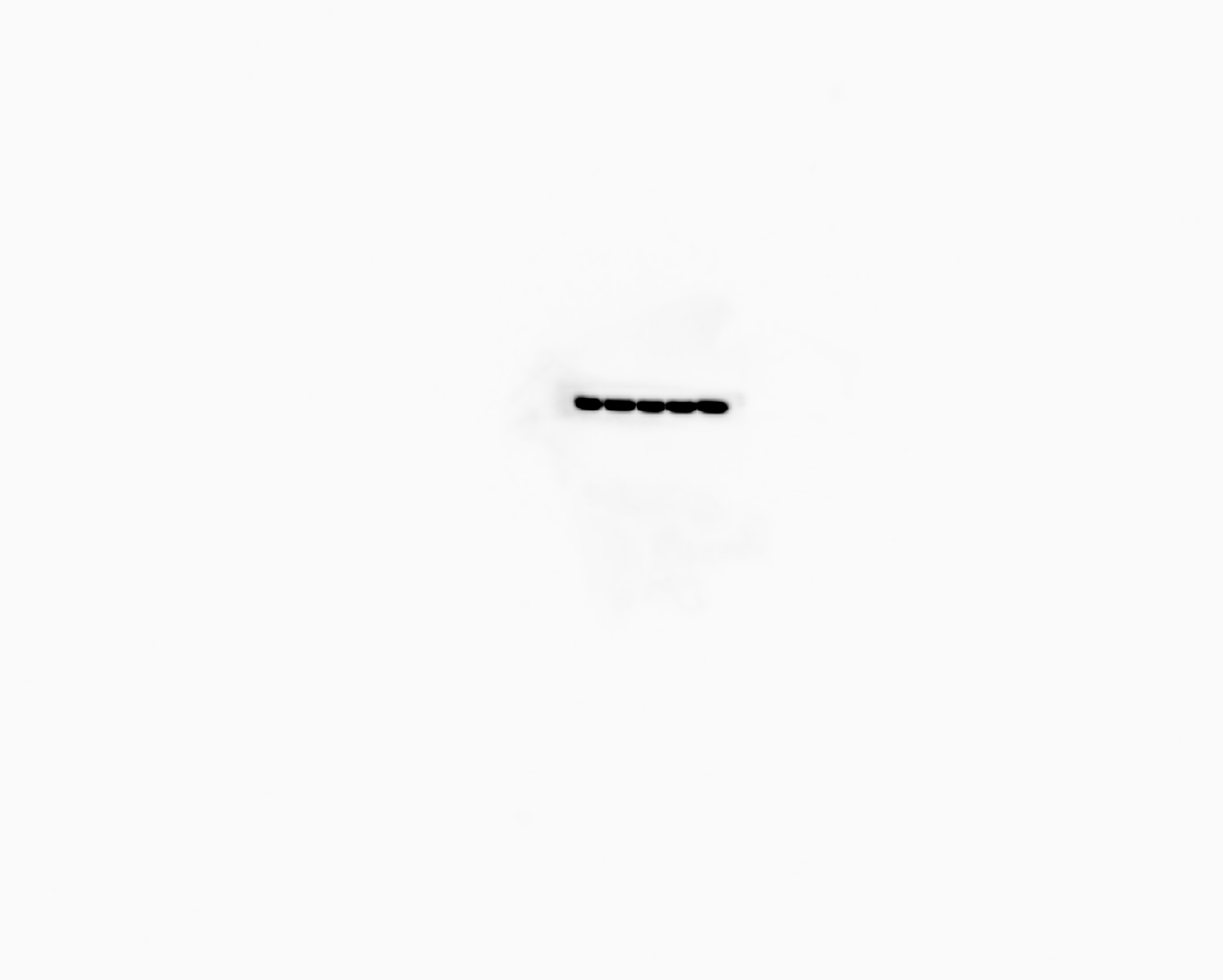

Supplement: Supplementary file 7 — Additional file 7. [file 12964_2024_1475_MOESM7_ESM.zip › Additional file 2/Figure 3D/KYSE-30/oePDIA3P1 a┬-actin.tif]

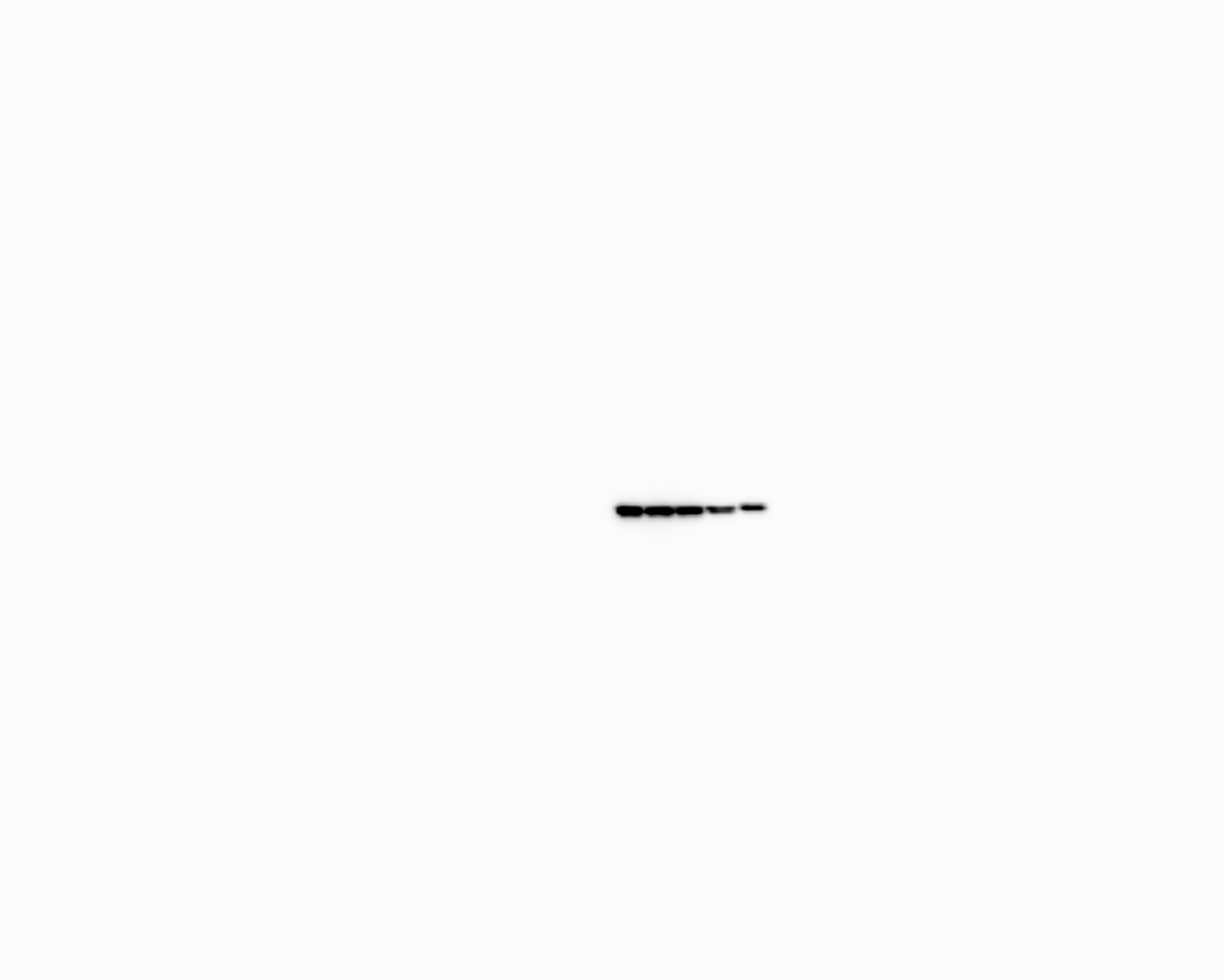

Supplement: Supplementary file 7 — Additional file 7. [file 12964_2024_1475_MOESM7_ESM.zip › Additional file 2/Figure 3D/KYSE-30/pc oct4.tif]

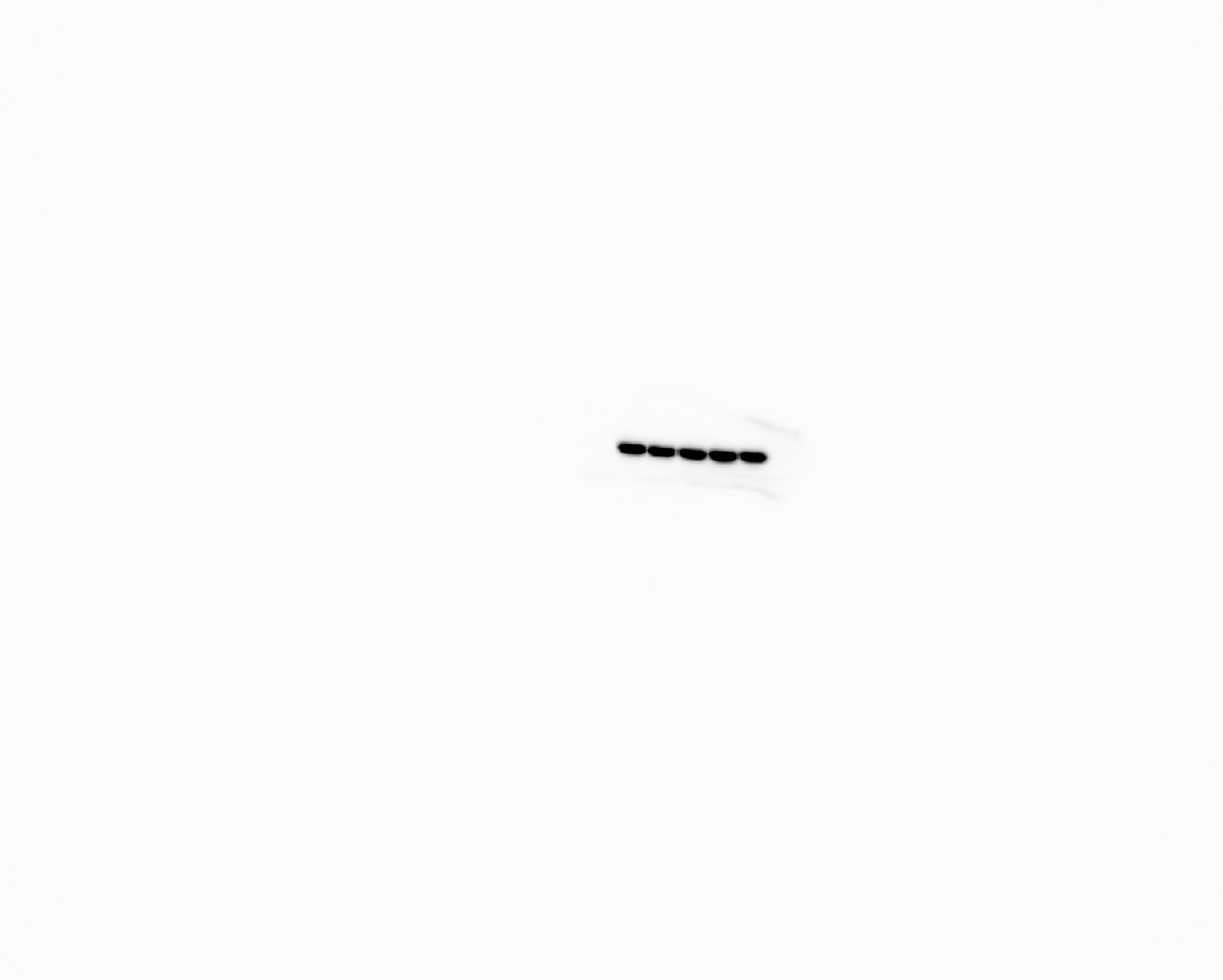

Supplement: Supplementary file 7 — Additional file 7. [file 12964_2024_1475_MOESM7_ESM.zip › Additional file 2/Figure 3D/KYSE-30/pc a┬-actin.tif]

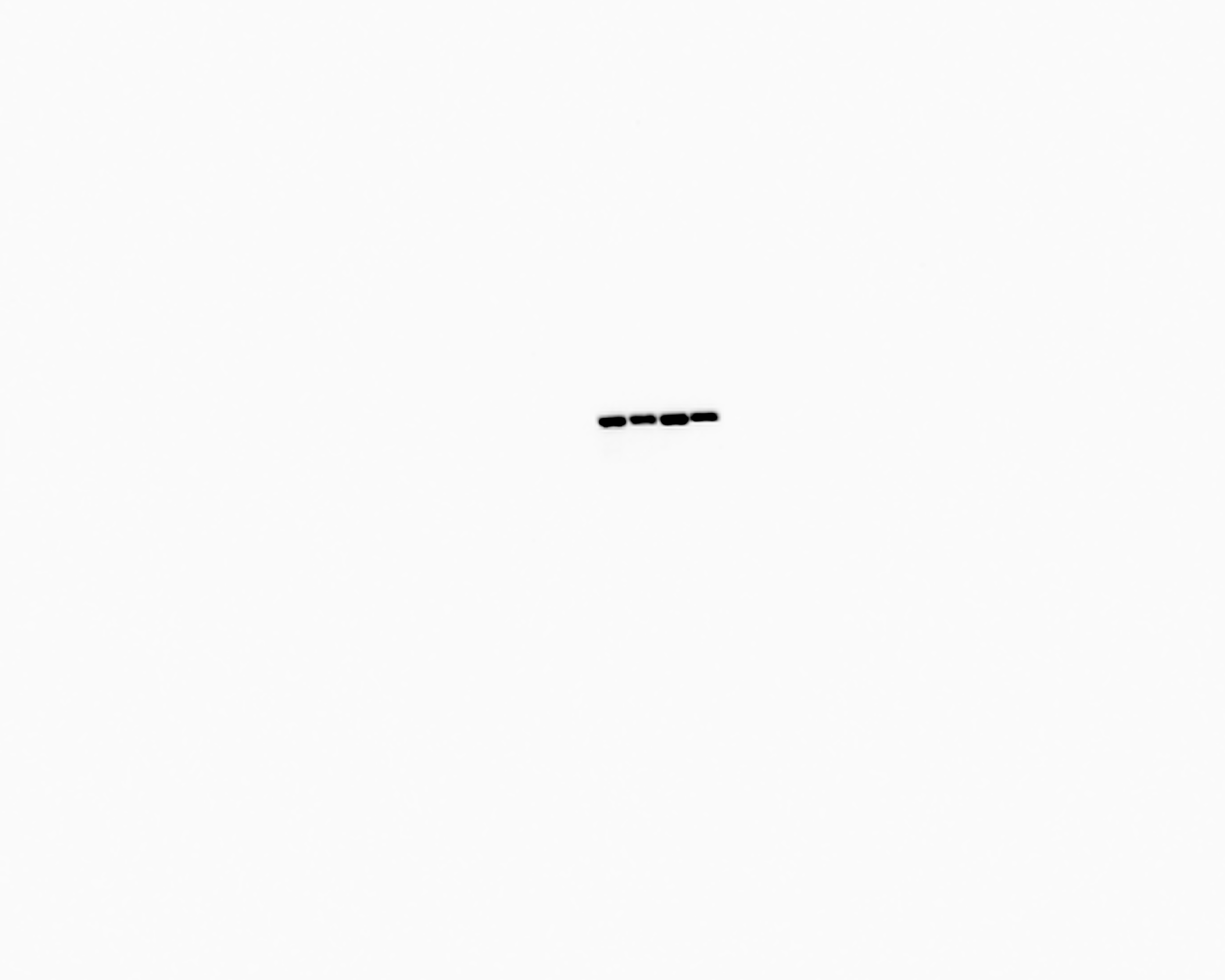

Supplement: Supplementary file 7 — Additional file 7. [file 12964_2024_1475_MOESM7_ESM.zip › Additional file 2/Figure 3E/Eca-109/oct4.tif]

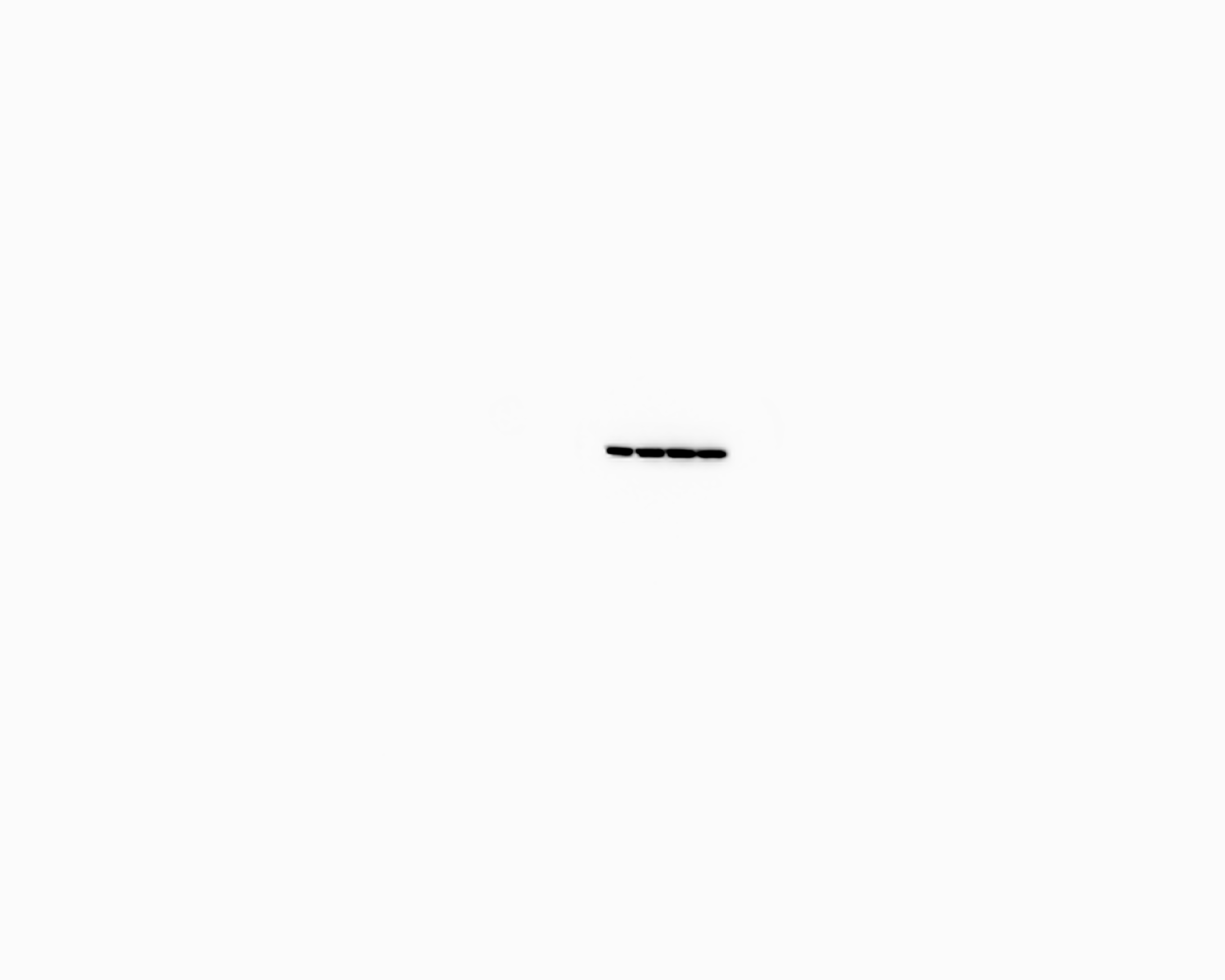

Supplement: Supplementary file 7 — Additional file 7. [file 12964_2024_1475_MOESM7_ESM.zip › Additional file 2/Figure 3E/Eca-109/a┬-actin.tif]

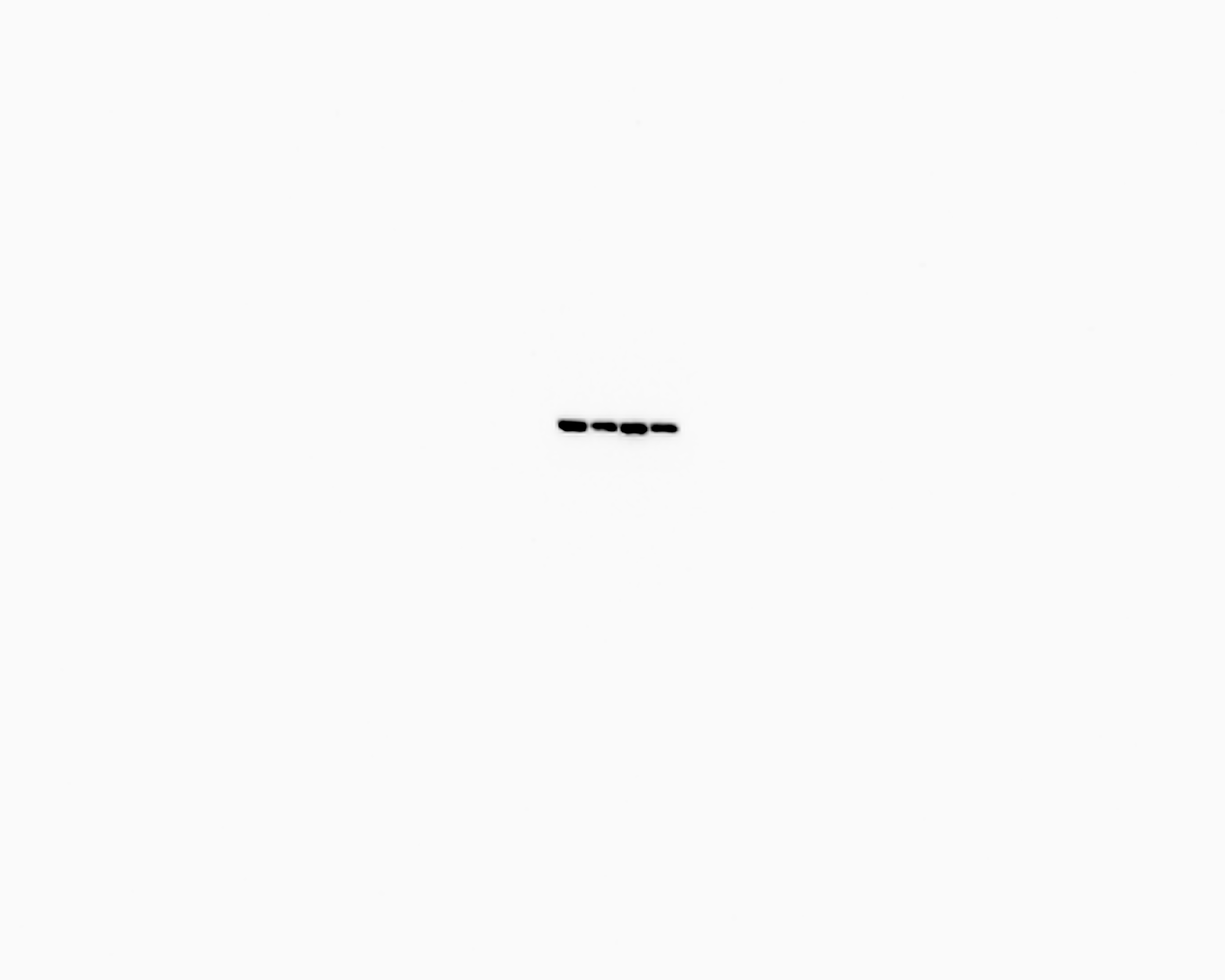

Supplement: Supplementary file 7 — Additional file 7. [file 12964_2024_1475_MOESM7_ESM.zip › Additional file 2/Figure 3E/TE-1/oct4.tif]

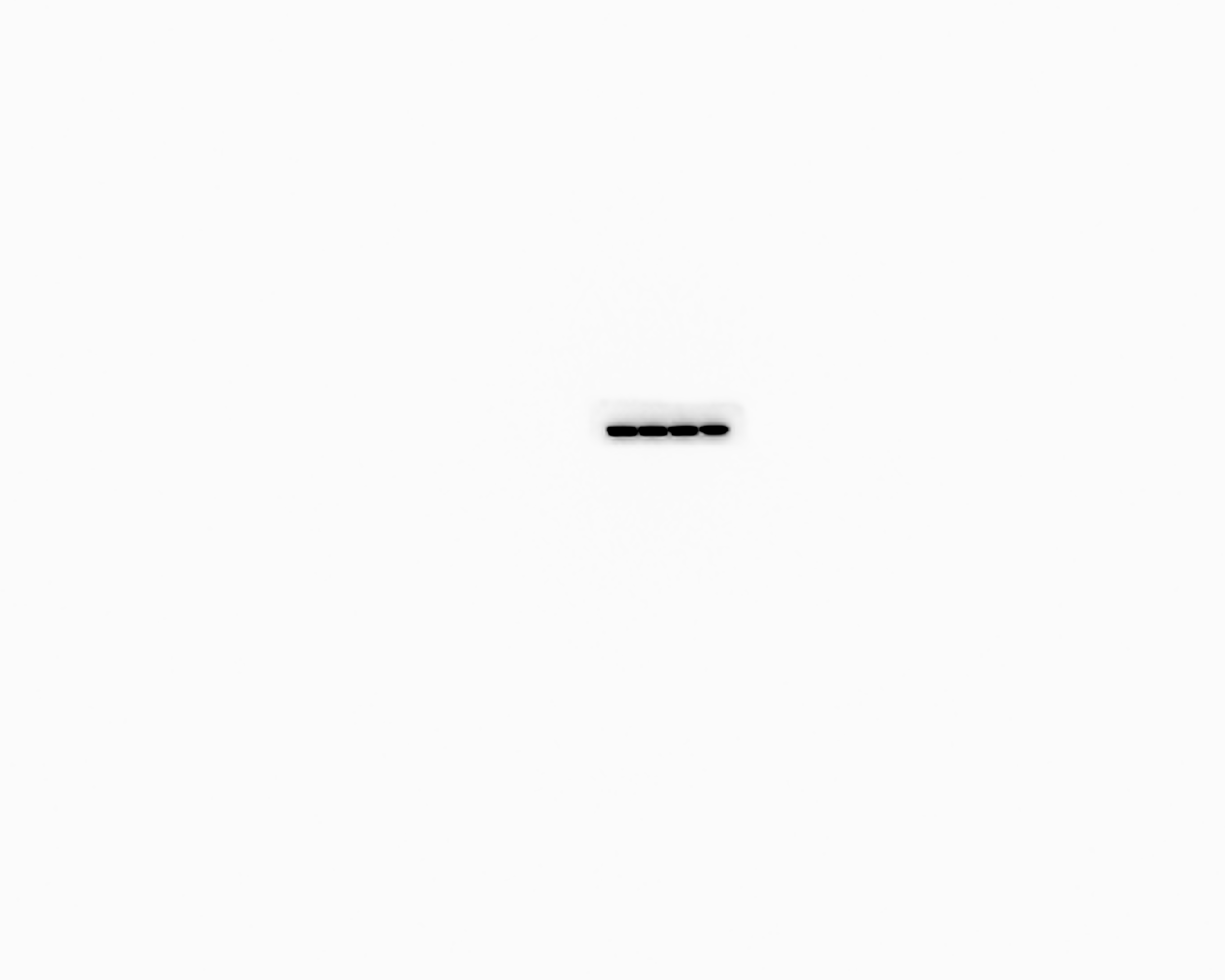

Supplement: Supplementary file 7 — Additional file 7. [file 12964_2024_1475_MOESM7_ESM.zip › Additional file 2/Figure 3E/TE-1/a┬-actin.tif]

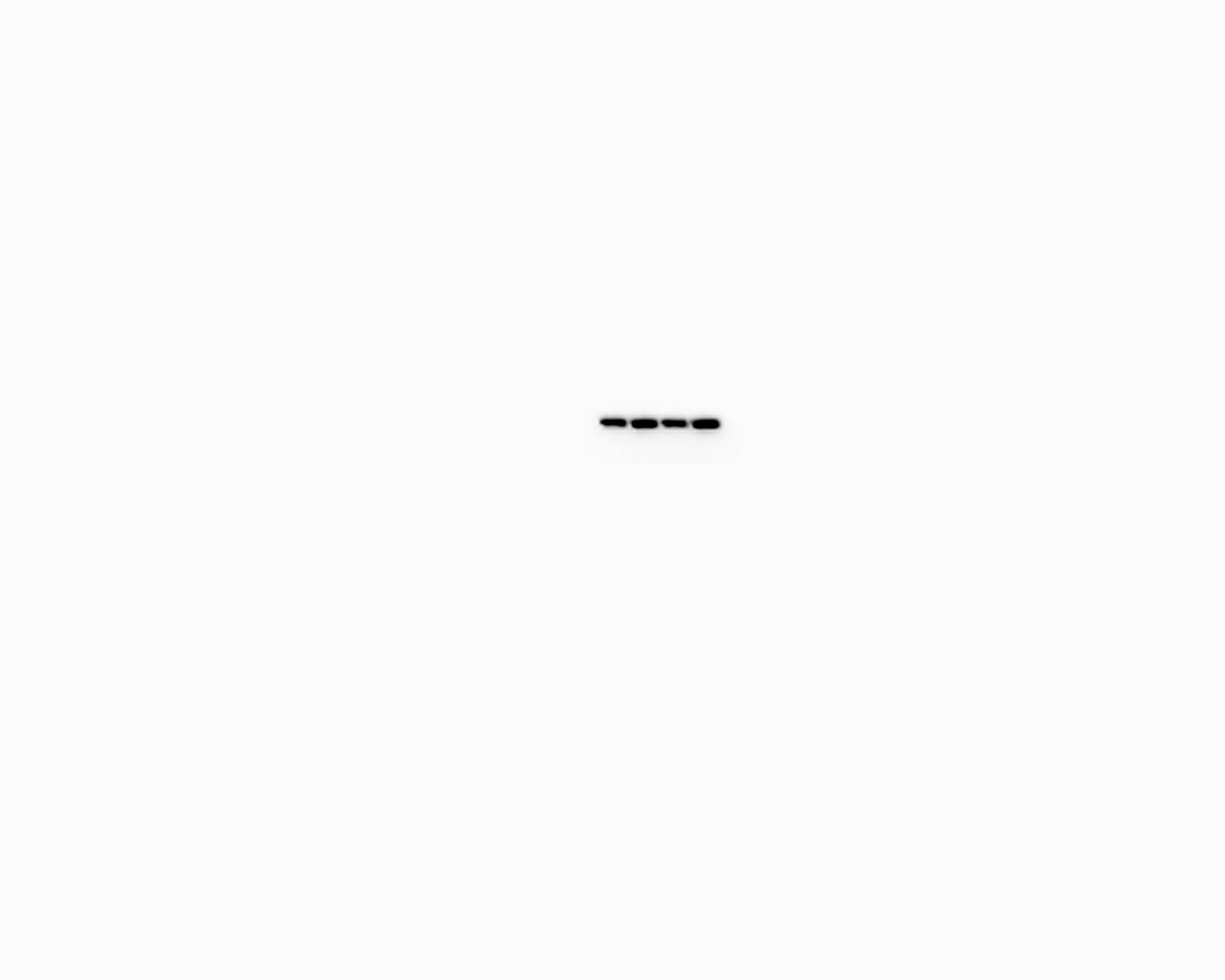

Supplement: Supplementary file 7 — Additional file 7. [file 12964_2024_1475_MOESM7_ESM.zip › Additional file 2/Figure 3F/KYSE-150/oct4.tif]

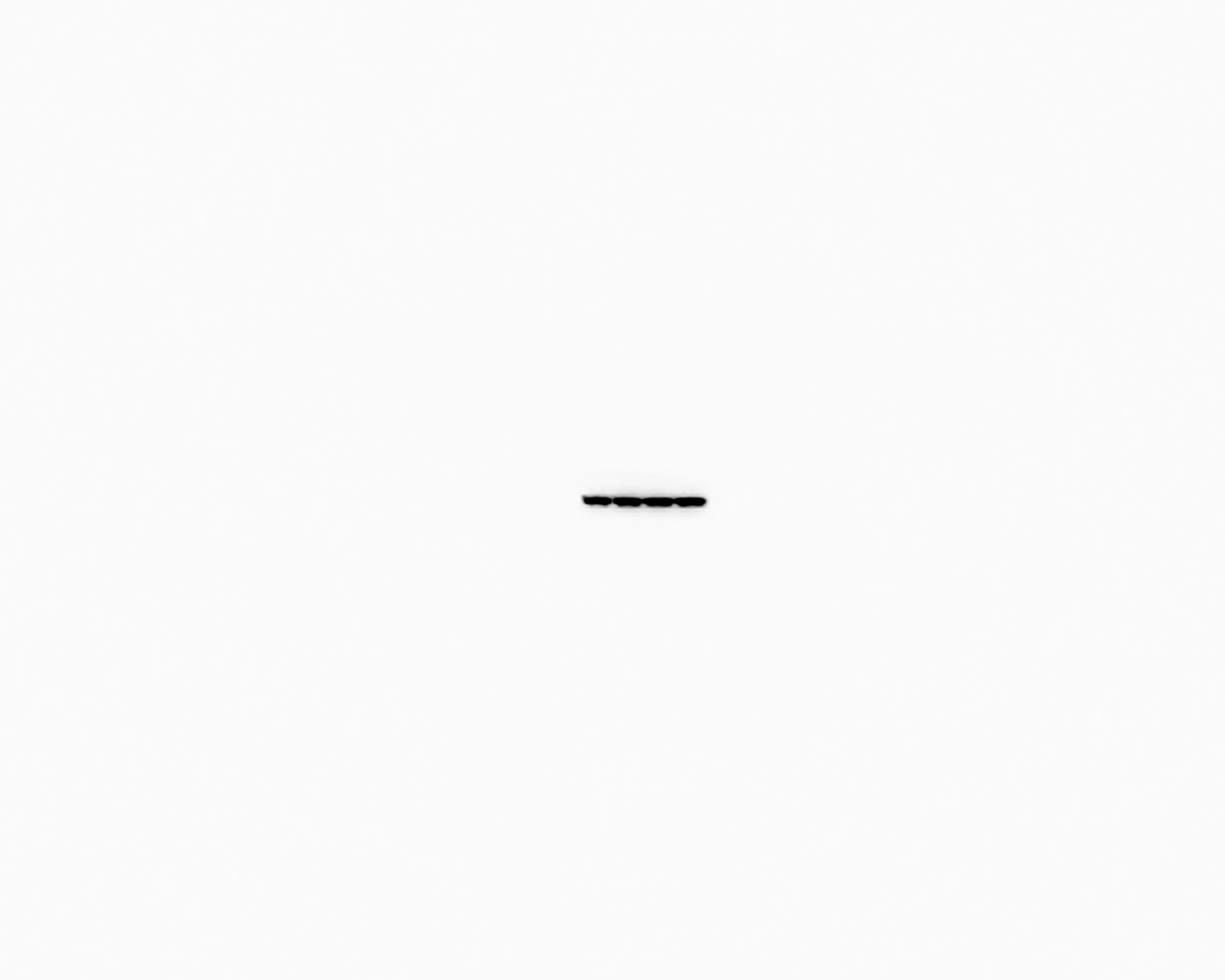

Supplement: Supplementary file 7 — Additional file 7. [file 12964_2024_1475_MOESM7_ESM.zip › Additional file 2/Figure 3F/KYSE-150/a┬-actin.tif]

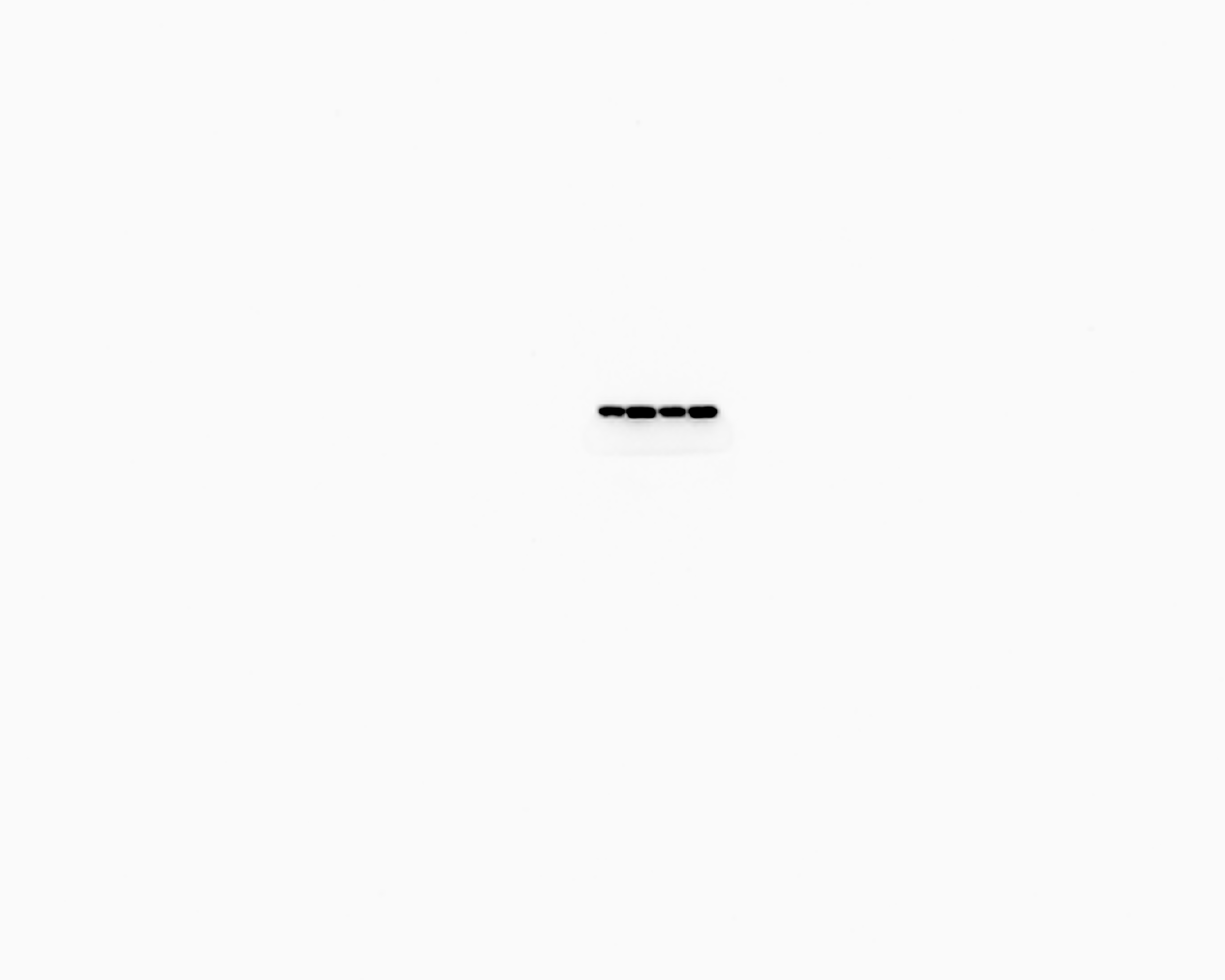

Supplement: Supplementary file 7 — Additional file 7. [file 12964_2024_1475_MOESM7_ESM.zip › Additional file 2/Figure 3F/KYSE-30/oct4.tif]

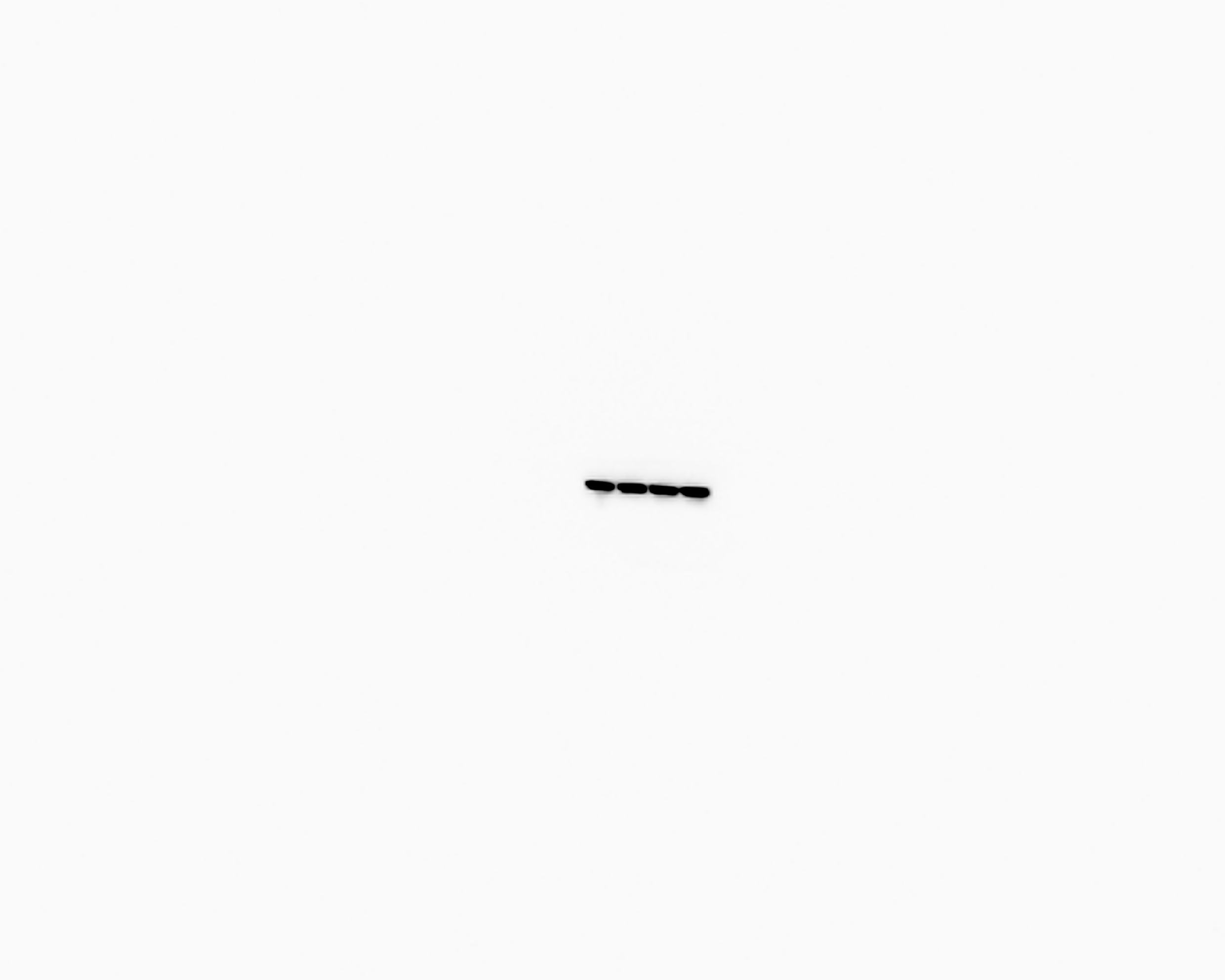

Supplement: Supplementary file 7 — Additional file 7. [file 12964_2024_1475_MOESM7_ESM.zip › Additional file 2/Figure 3F/KYSE-30/a┬-actin.tif]

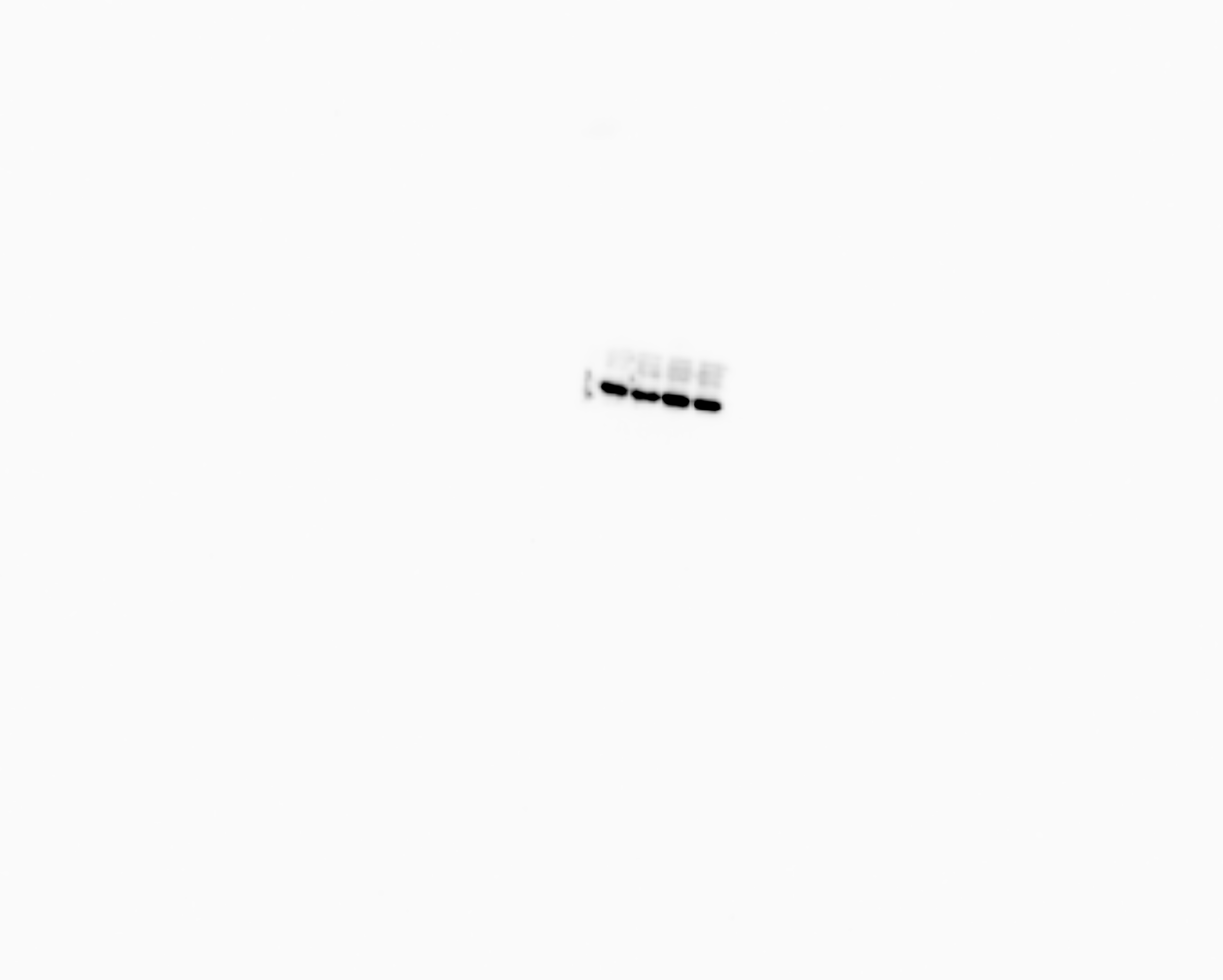

Supplement: Supplementary file 7 — Additional file 7. [file 12964_2024_1475_MOESM7_ESM.zip › Additional file 2/Figure 3G/Eca-109/oct4.tif]

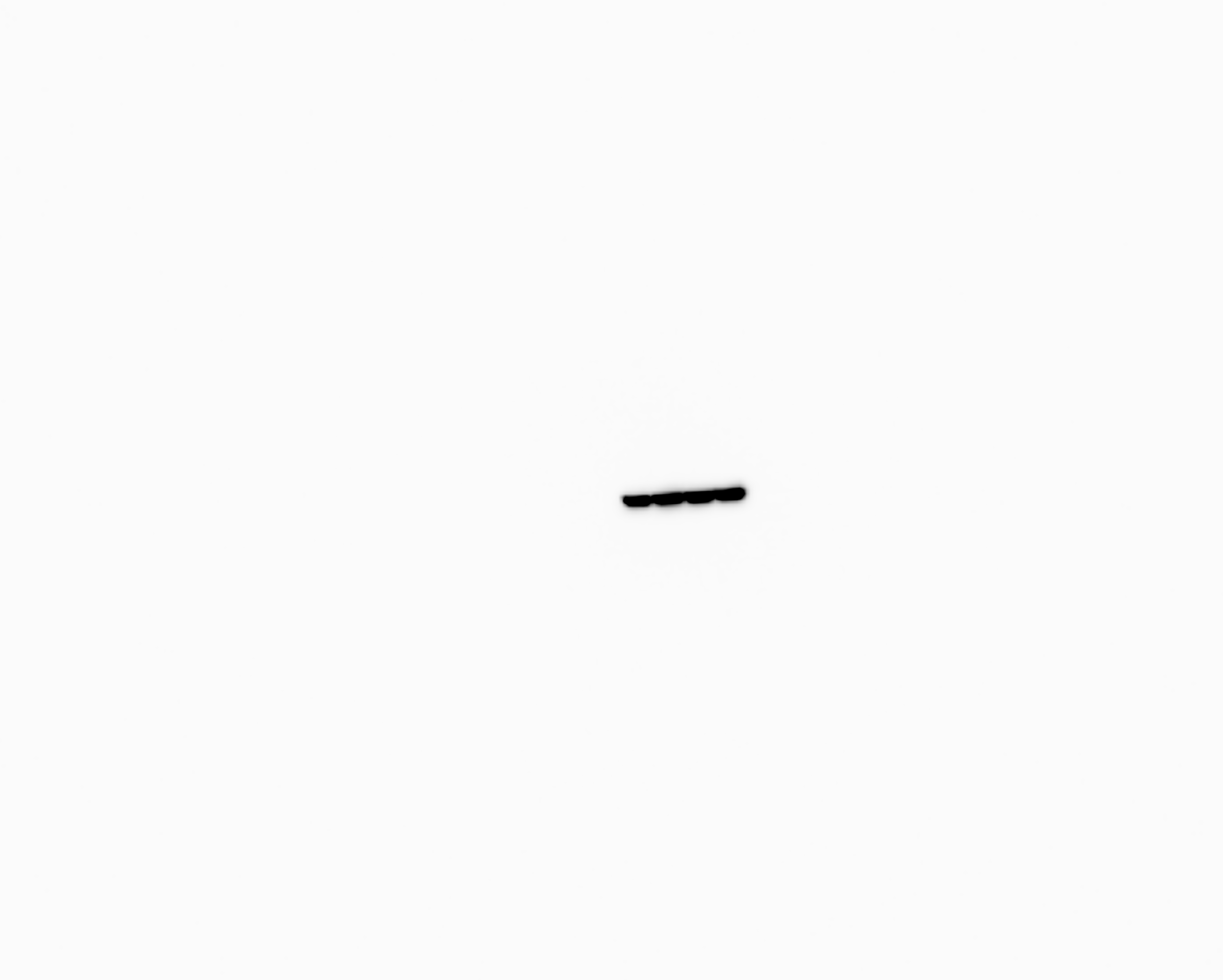

Supplement: Supplementary file 7 — Additional file 7. [file 12964_2024_1475_MOESM7_ESM.zip › Additional file 2/Figure 3G/Eca-109/a┬-actin.tif]

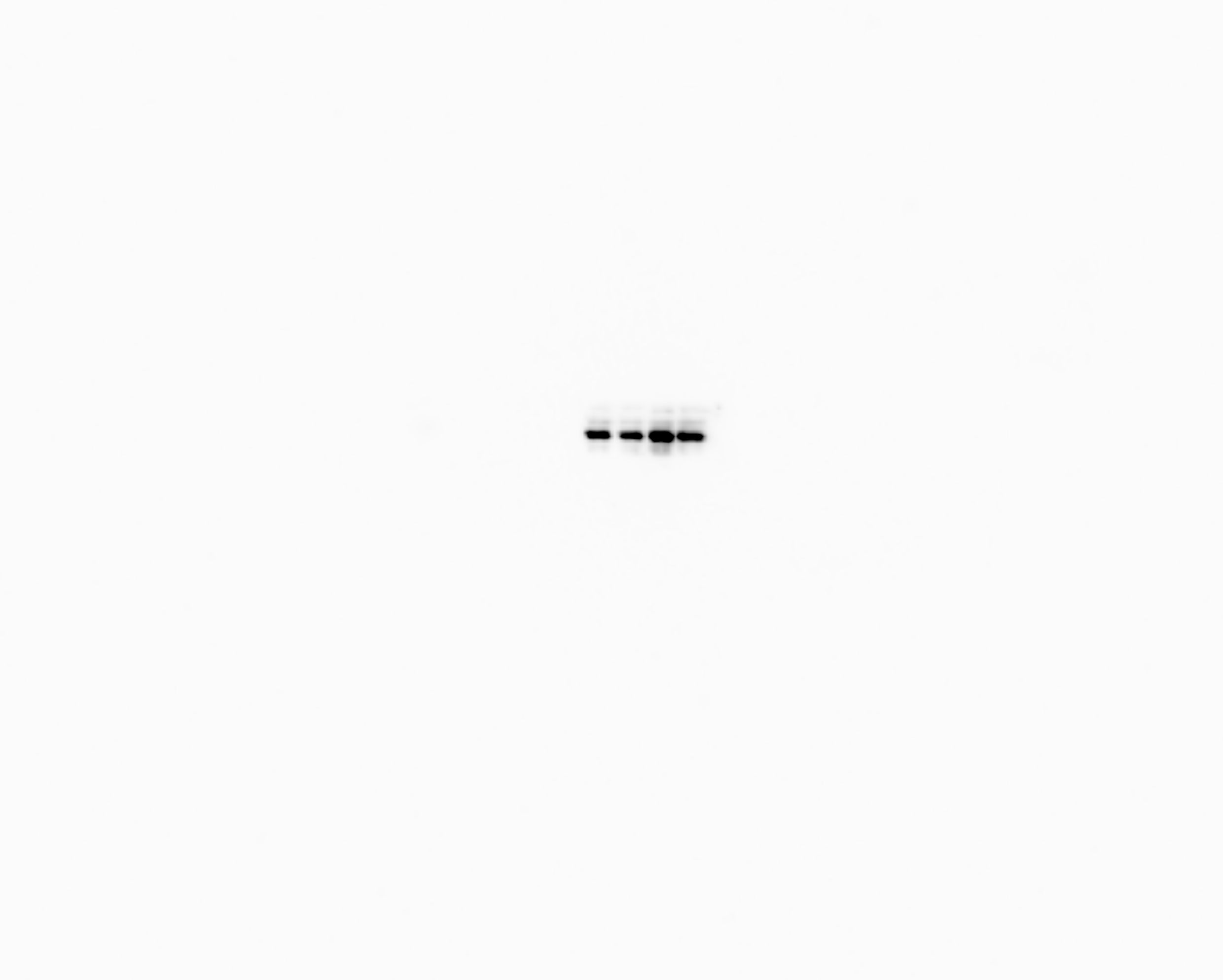

Supplement: Supplementary file 7 — Additional file 7. [file 12964_2024_1475_MOESM7_ESM.zip › Additional file 2/Figure 3G/TE-1/oct4.tif]

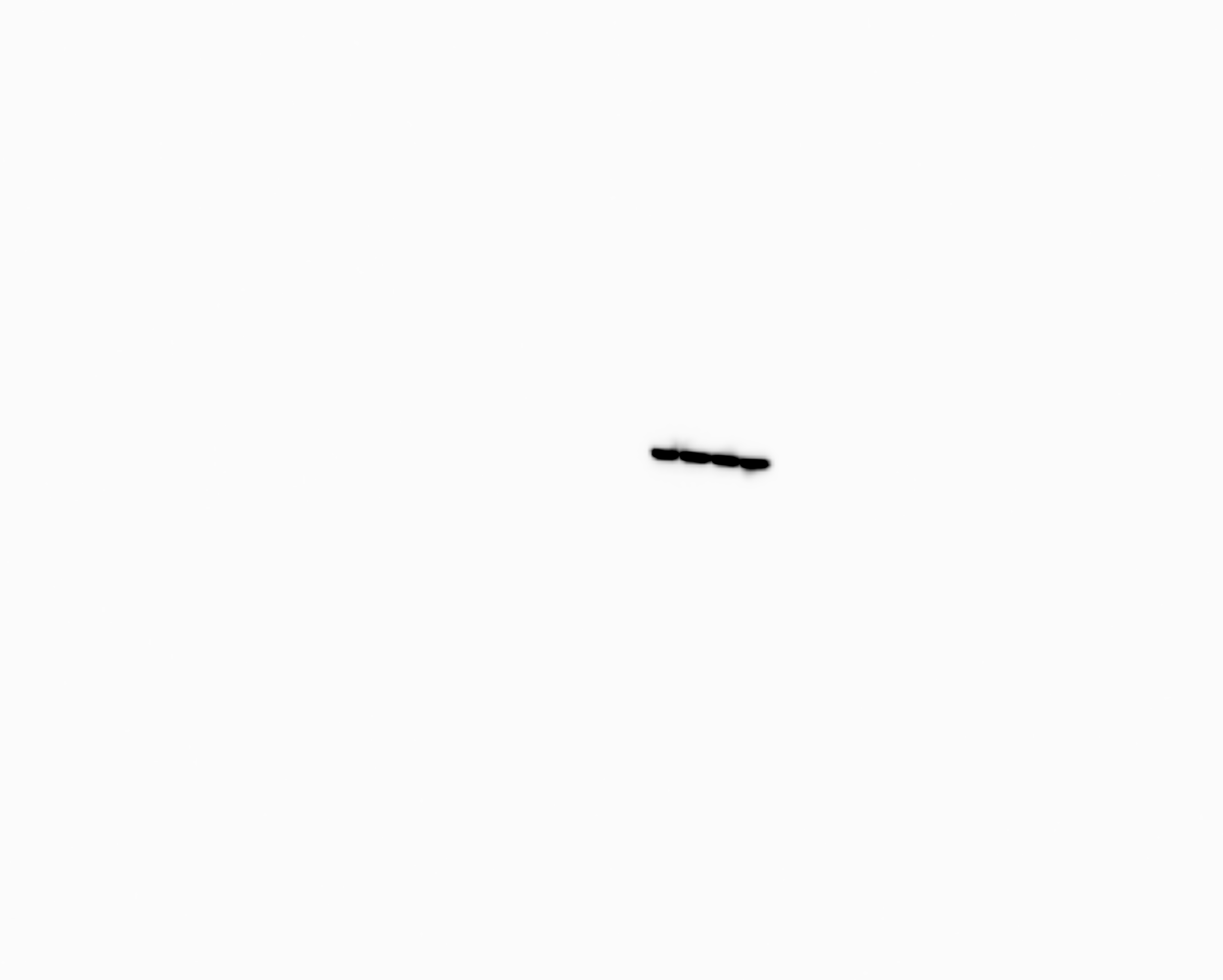

Supplement: Supplementary file 7 — Additional file 7. [file 12964_2024_1475_MOESM7_ESM.zip › Additional file 2/Figure 3G/TE-1/a┬-actin.tif]

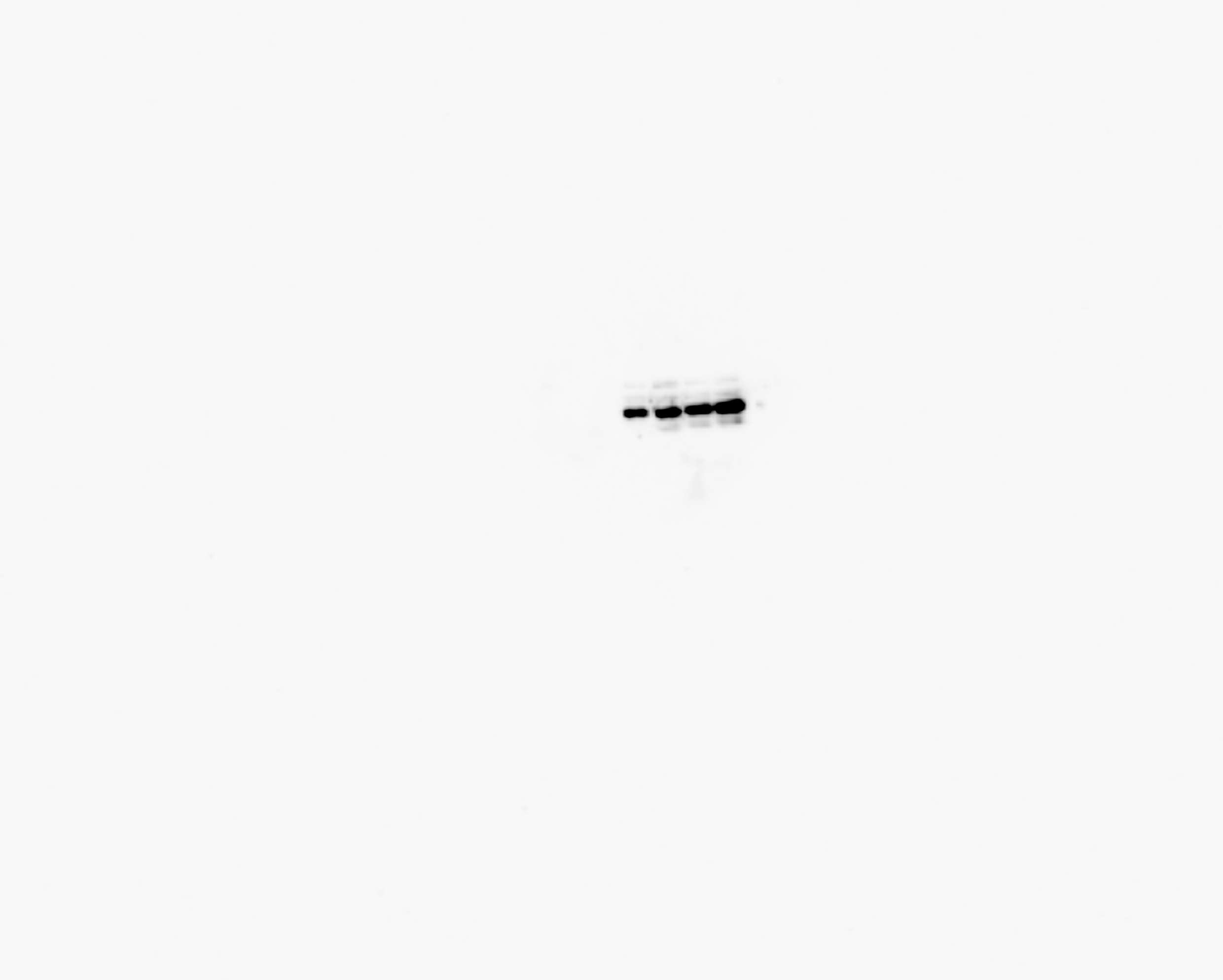

Supplement: Supplementary file 7 — Additional file 7. [file 12964_2024_1475_MOESM7_ESM.zip › Additional file 2/Figure 3H/KYSE-150/oct4.tif]

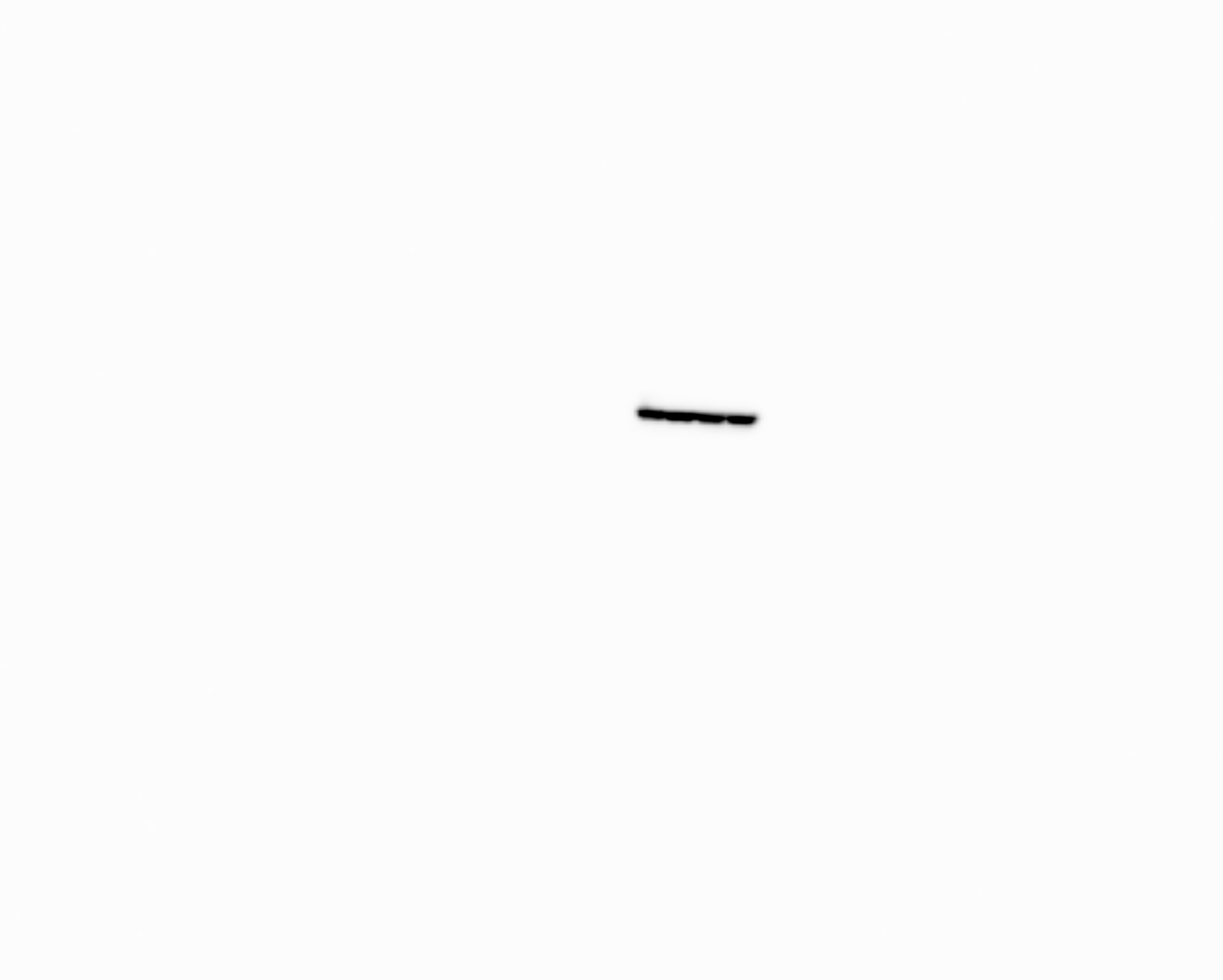

Supplement: Supplementary file 7 — Additional file 7. [file 12964_2024_1475_MOESM7_ESM.zip › Additional file 2/Figure 3H/KYSE-150/a┬-actin.tif]

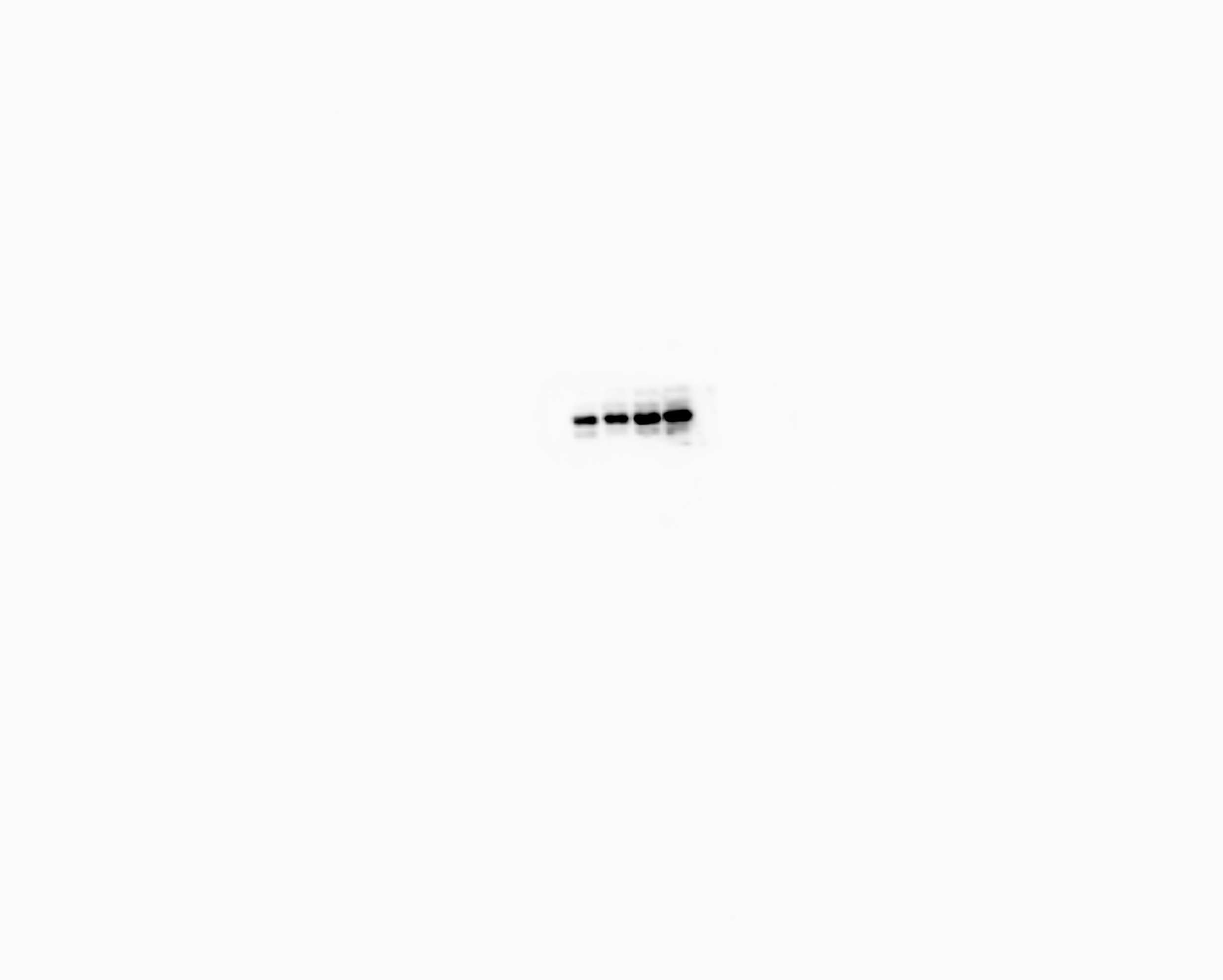

Supplement: Supplementary file 7 — Additional file 7. [file 12964_2024_1475_MOESM7_ESM.zip › Additional file 2/Figure 3H/KYSE-30/oct4.tif]

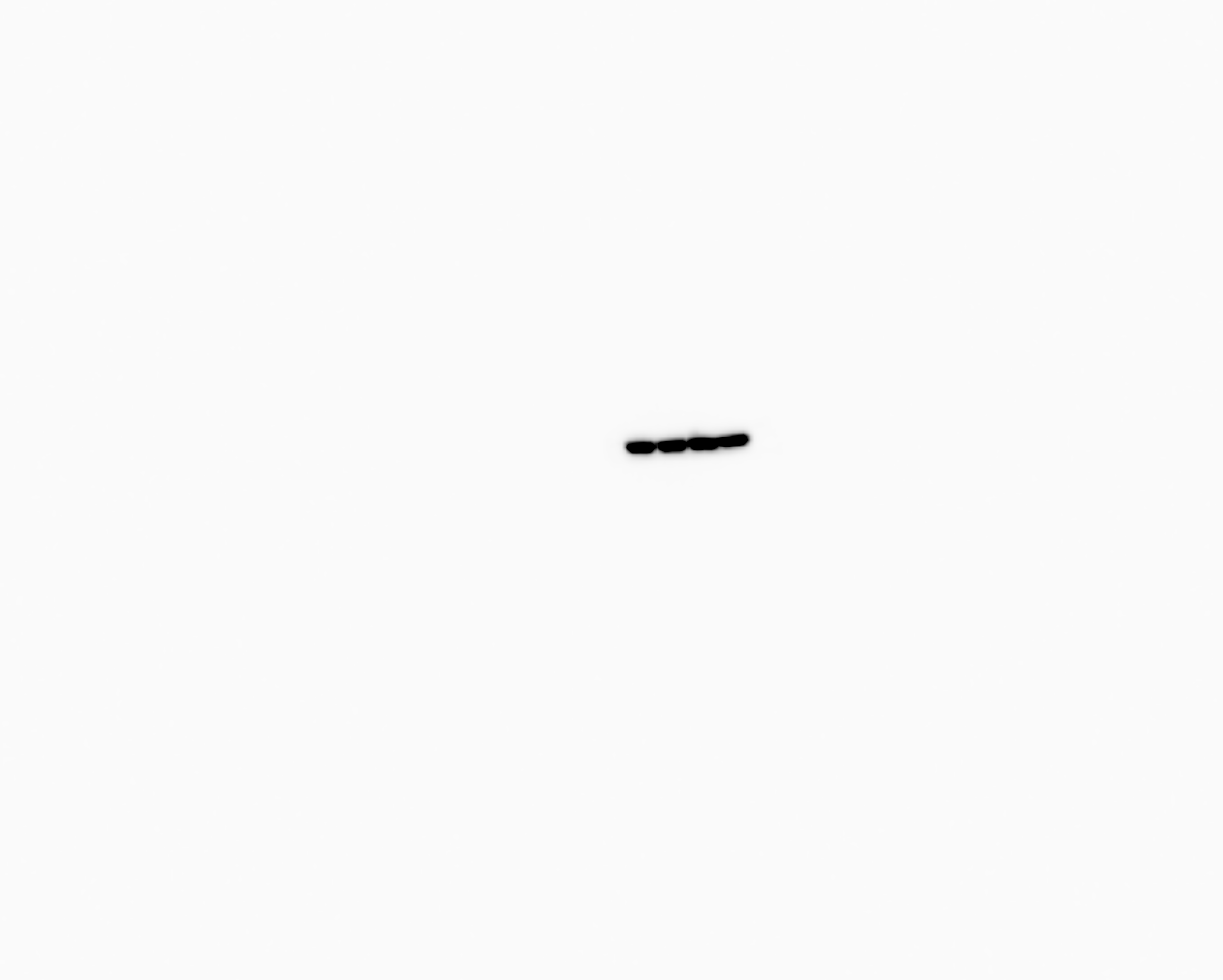

Supplement: Supplementary file 7 — Additional file 7. [file 12964_2024_1475_MOESM7_ESM.zip › Additional file 2/Figure 3H/KYSE-30/a┬-actin.tif]

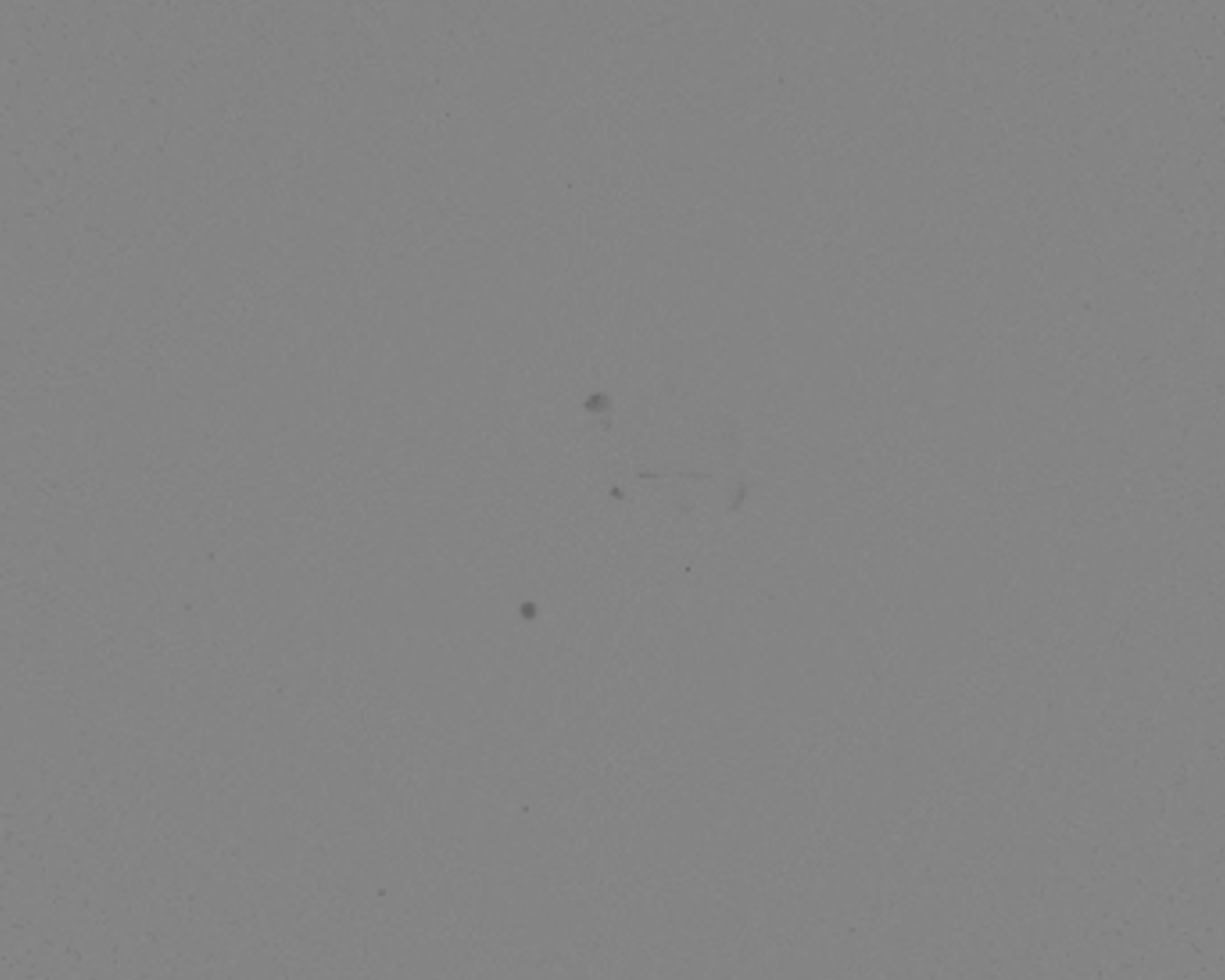

Supplement: Supplementary file 7 — Additional file 7. [file 12964_2024_1475_MOESM7_ESM.zip › Additional file 2/Figure 3I/Eca-109/IgG oct4.tif]

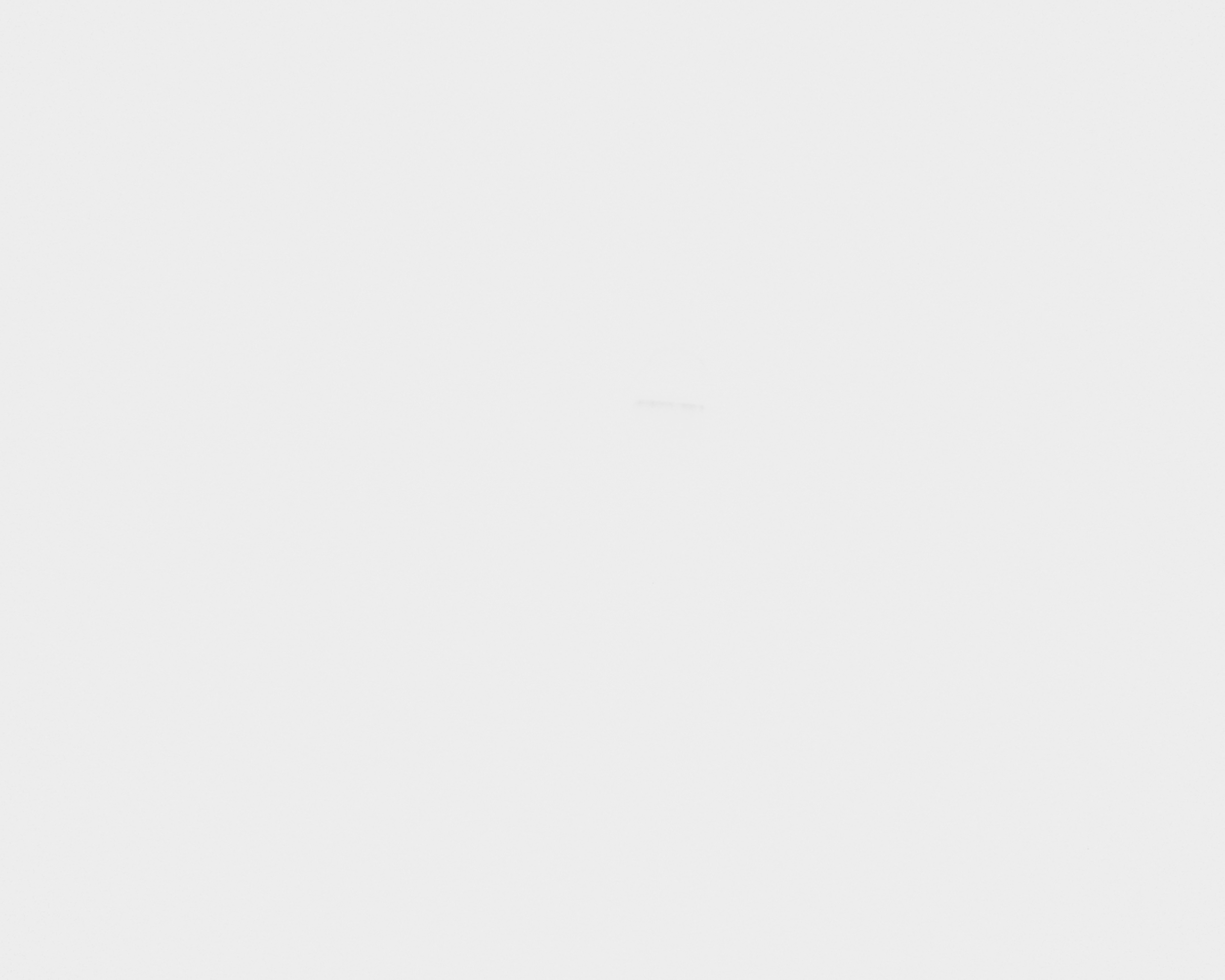

Supplement: Supplementary file 7 — Additional file 7. [file 12964_2024_1475_MOESM7_ESM.zip › Additional file 2/Figure 3I/Eca-109/IgG ubiquitin.tif]

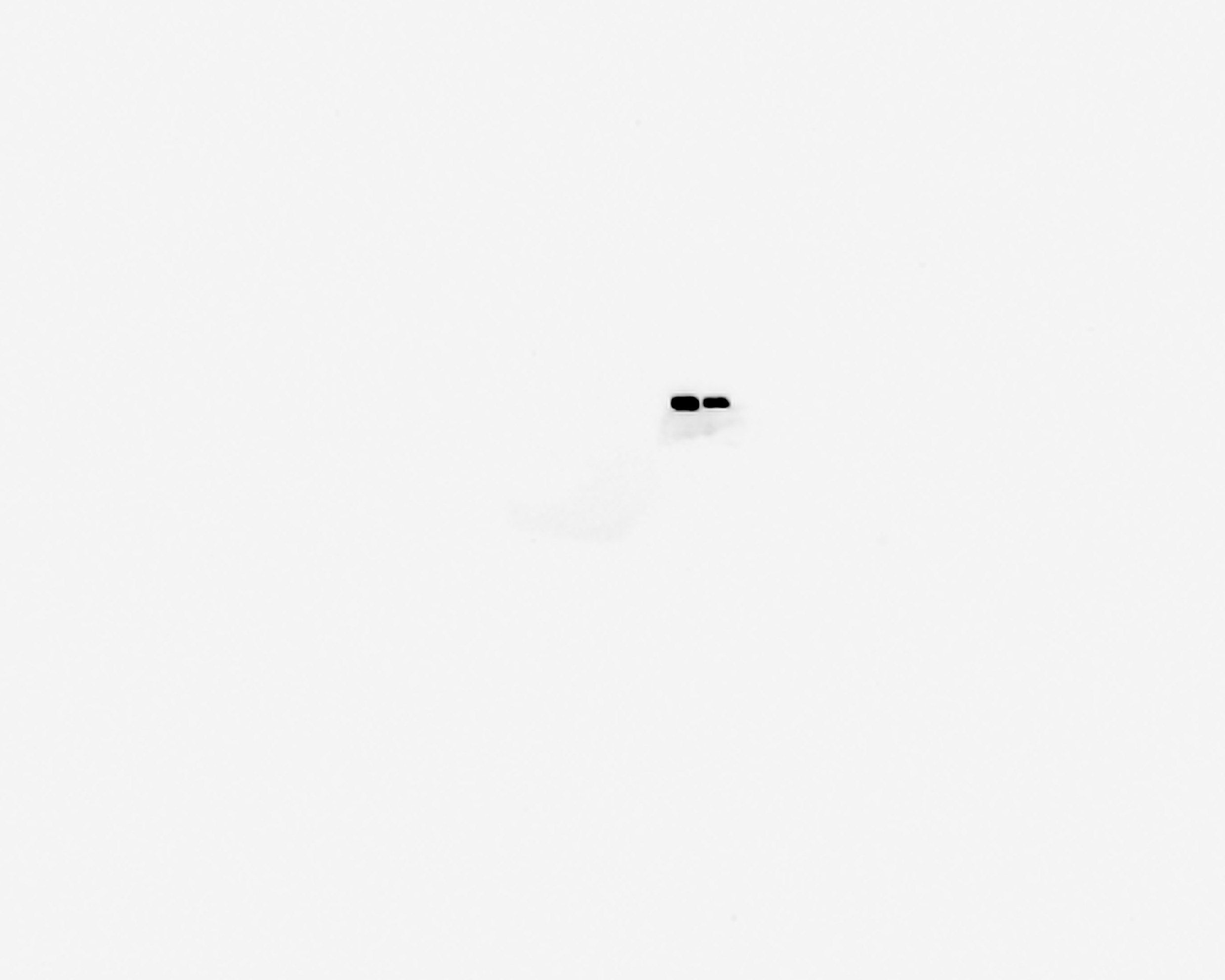

Supplement: Supplementary file 7 — Additional file 7. [file 12964_2024_1475_MOESM7_ESM.zip › Additional file 2/Figure 3I/Eca-109/input oct4.tif]

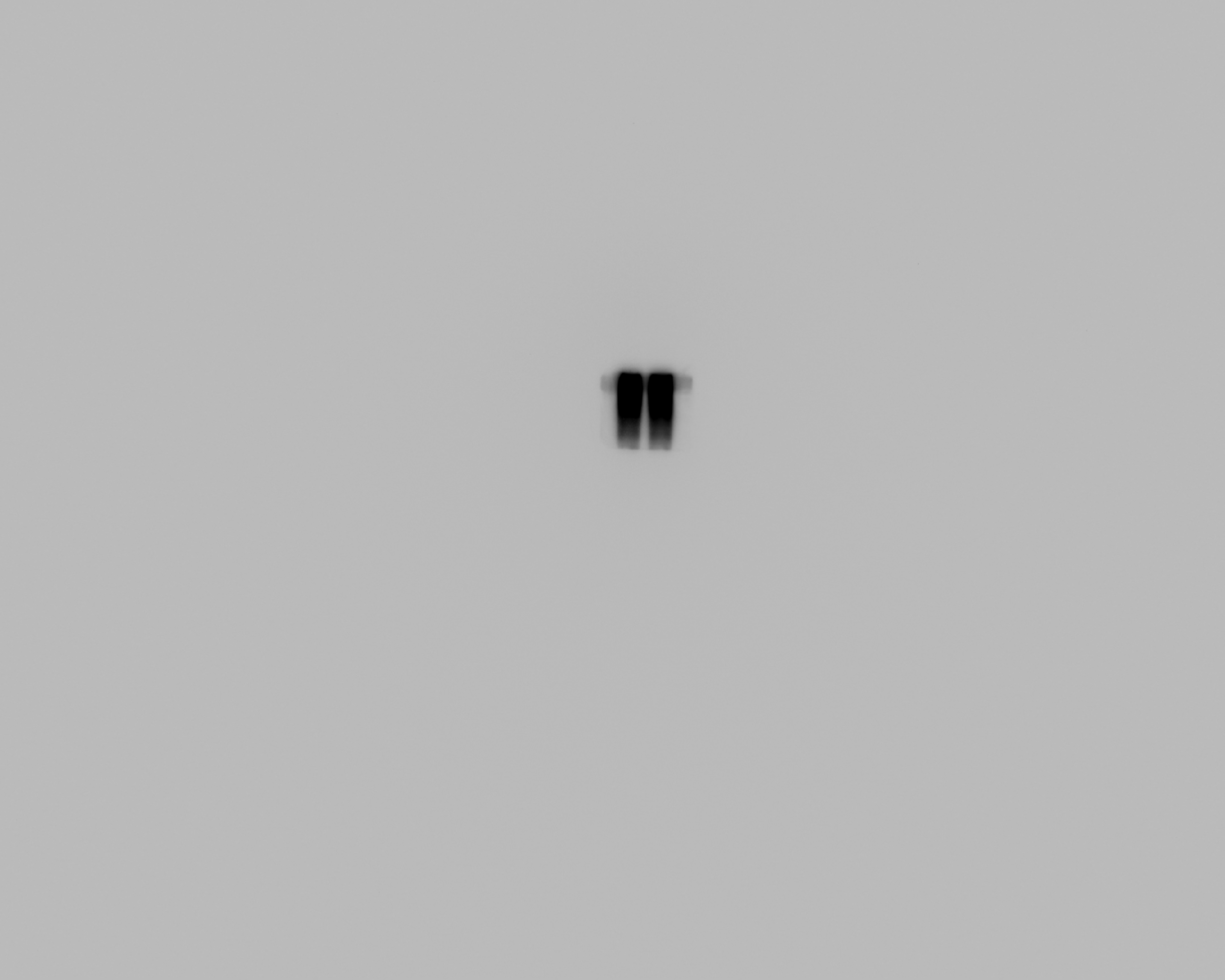

Supplement: Supplementary file 7 — Additional file 7. [file 12964_2024_1475_MOESM7_ESM.zip › Additional file 2/Figure 3I/Eca-109/input ubiquitin.tif]

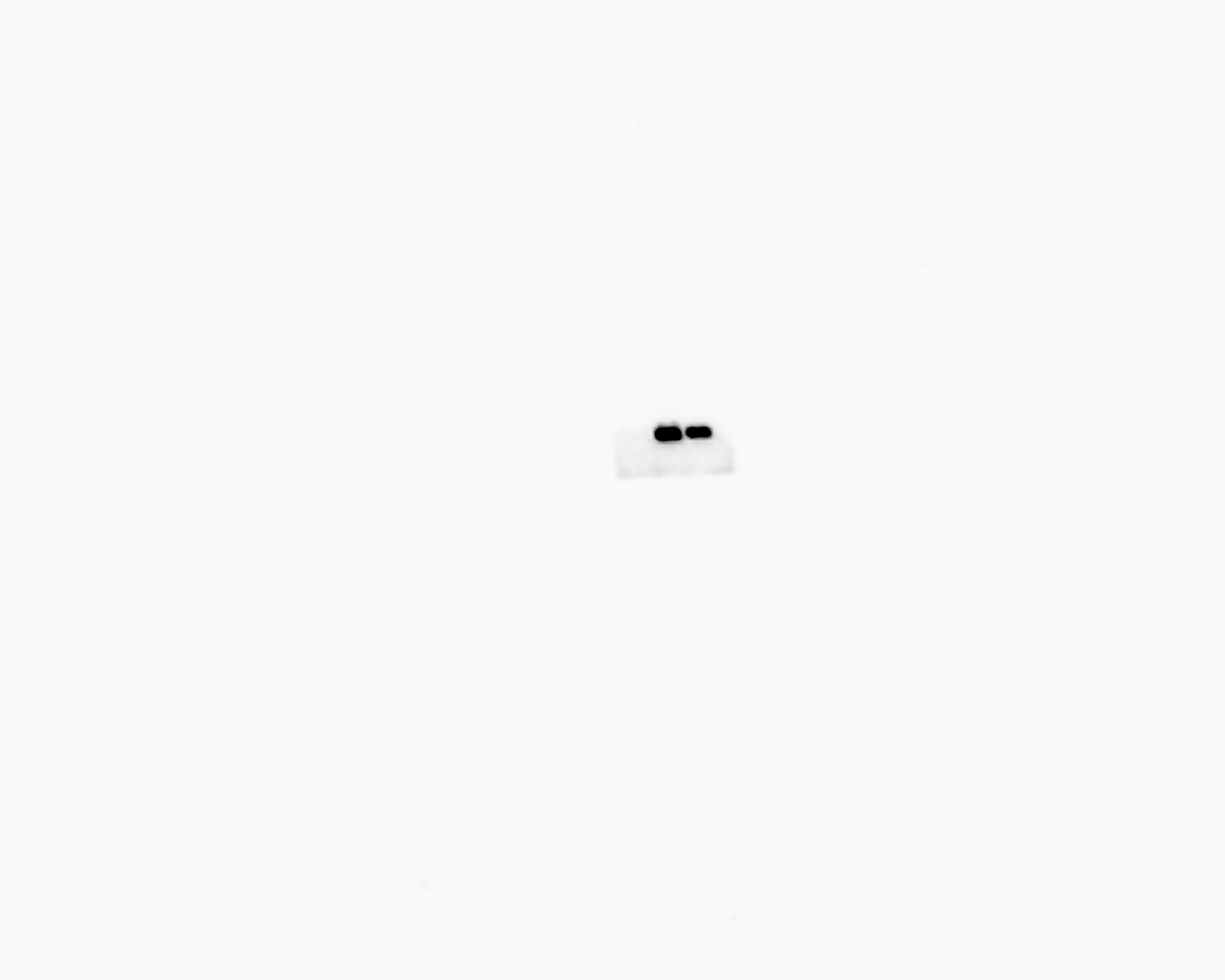

Supplement: Supplementary file 7 — Additional file 7. [file 12964_2024_1475_MOESM7_ESM.zip › Additional file 2/Figure 3I/Eca-109/ip oct4.tif]

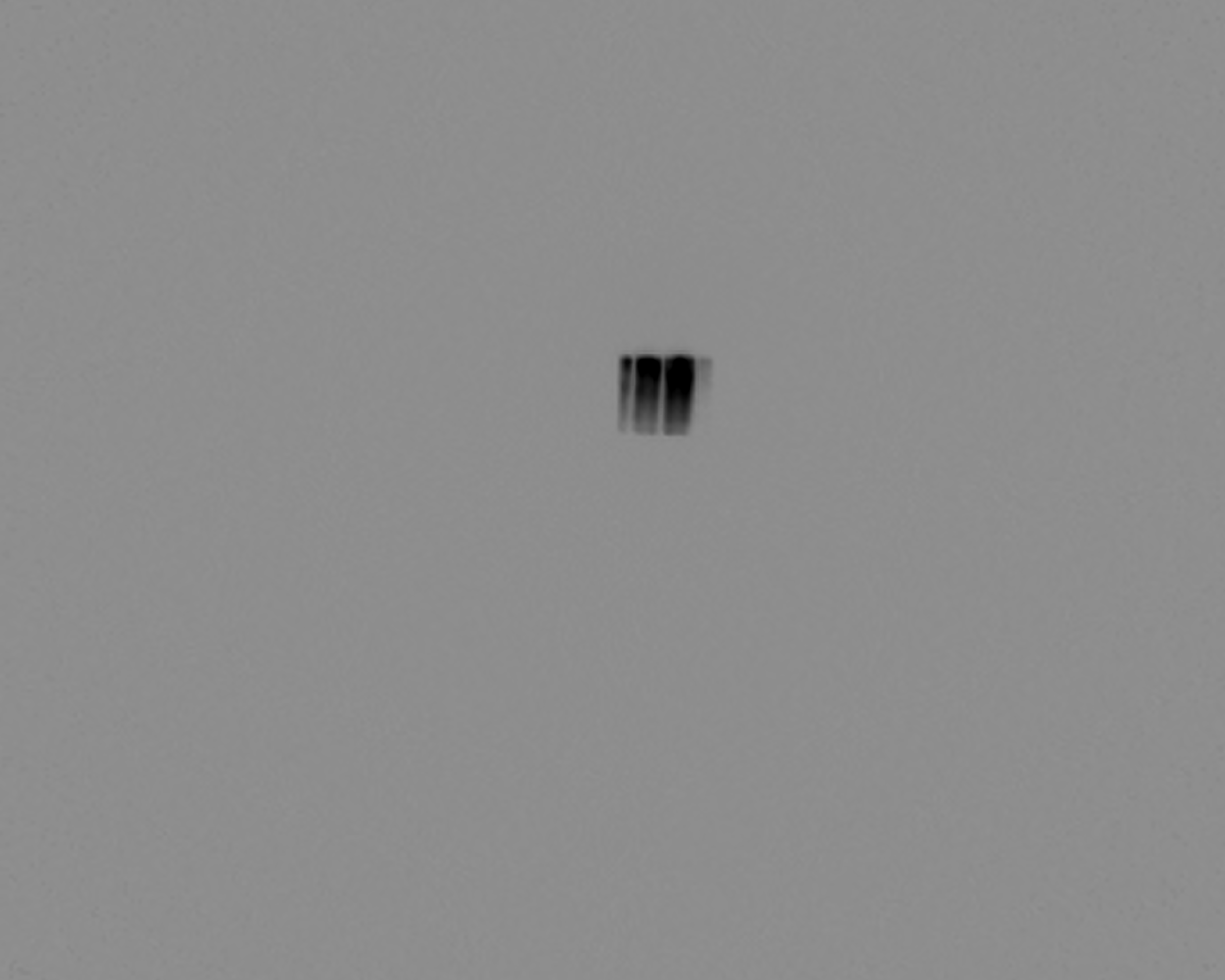

Supplement: Supplementary file 7 — Additional file 7. [file 12964_2024_1475_MOESM7_ESM.zip › Additional file 2/Figure 3I/Eca-109/ip ubiquitin.tif]

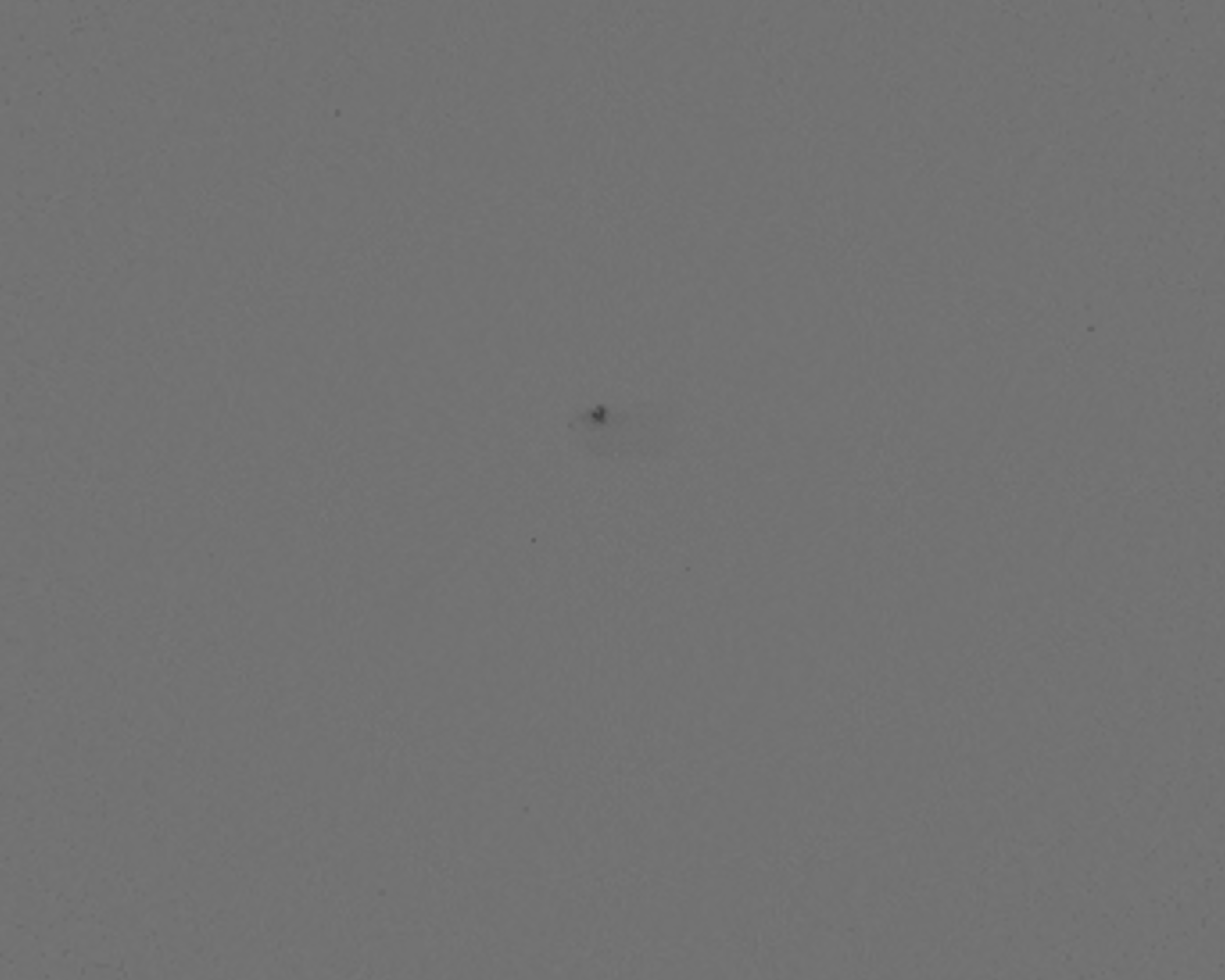

Supplement: Supplementary file 7 — Additional file 7. [file 12964_2024_1475_MOESM7_ESM.zip › Additional file 2/Figure 3I/TE-1/IgG oct4.tif]

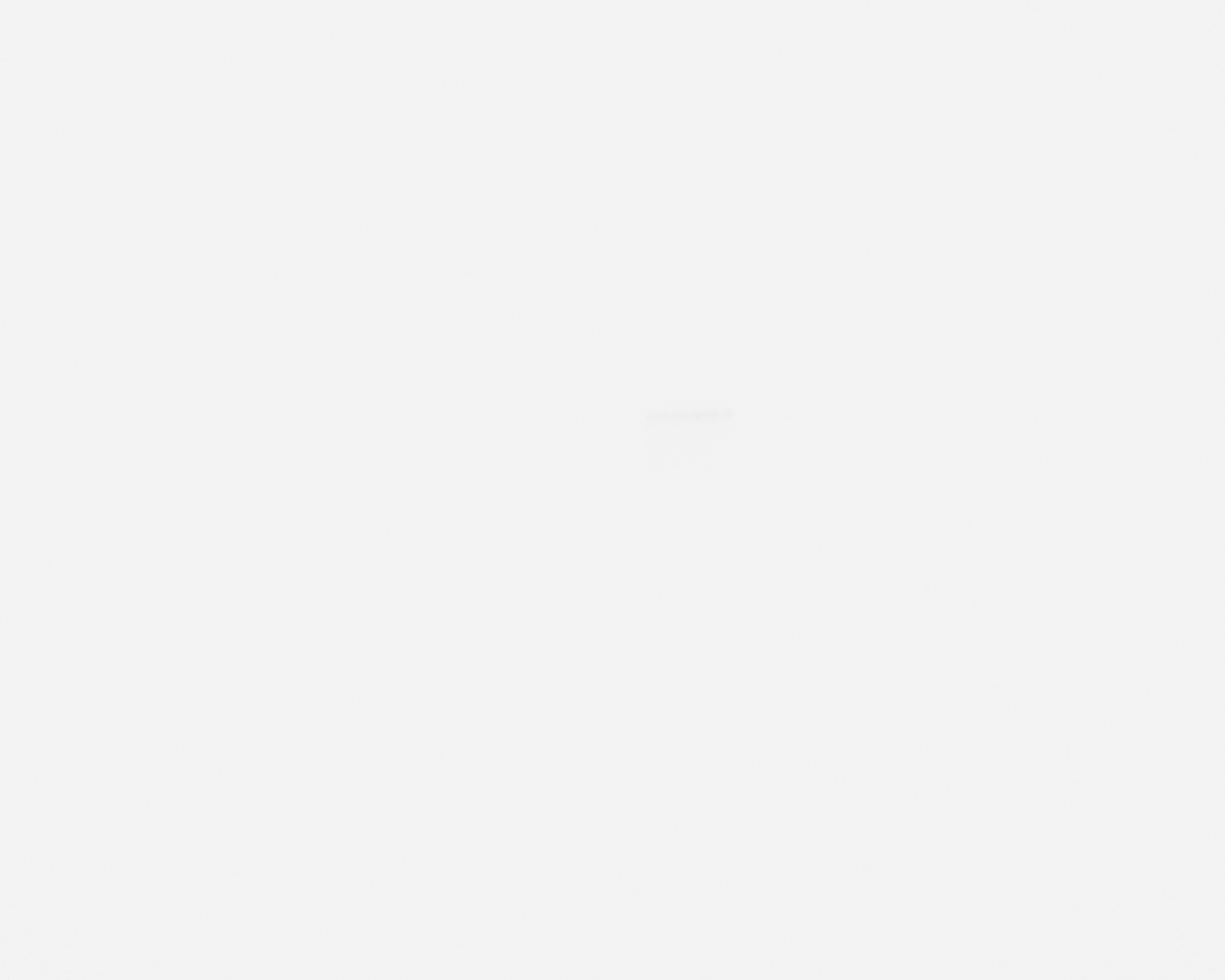

Supplement: Supplementary file 7 — Additional file 7. [file 12964_2024_1475_MOESM7_ESM.zip › Additional file 2/Figure 3I/TE-1/IgG ubiquitin.tif]

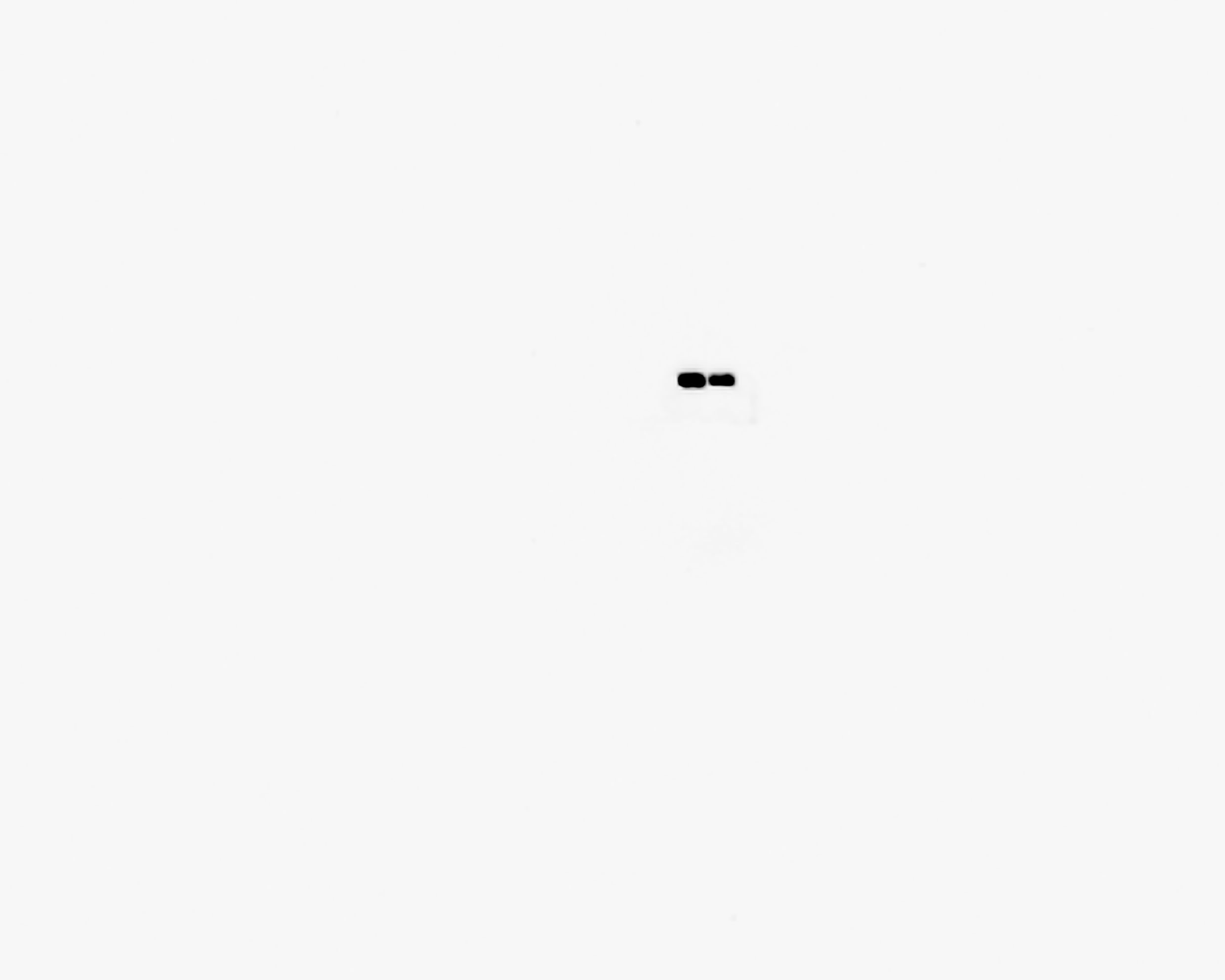

Supplement: Supplementary file 7 — Additional file 7. [file 12964_2024_1475_MOESM7_ESM.zip › Additional file 2/Figure 3I/TE-1/input oct4.tif]

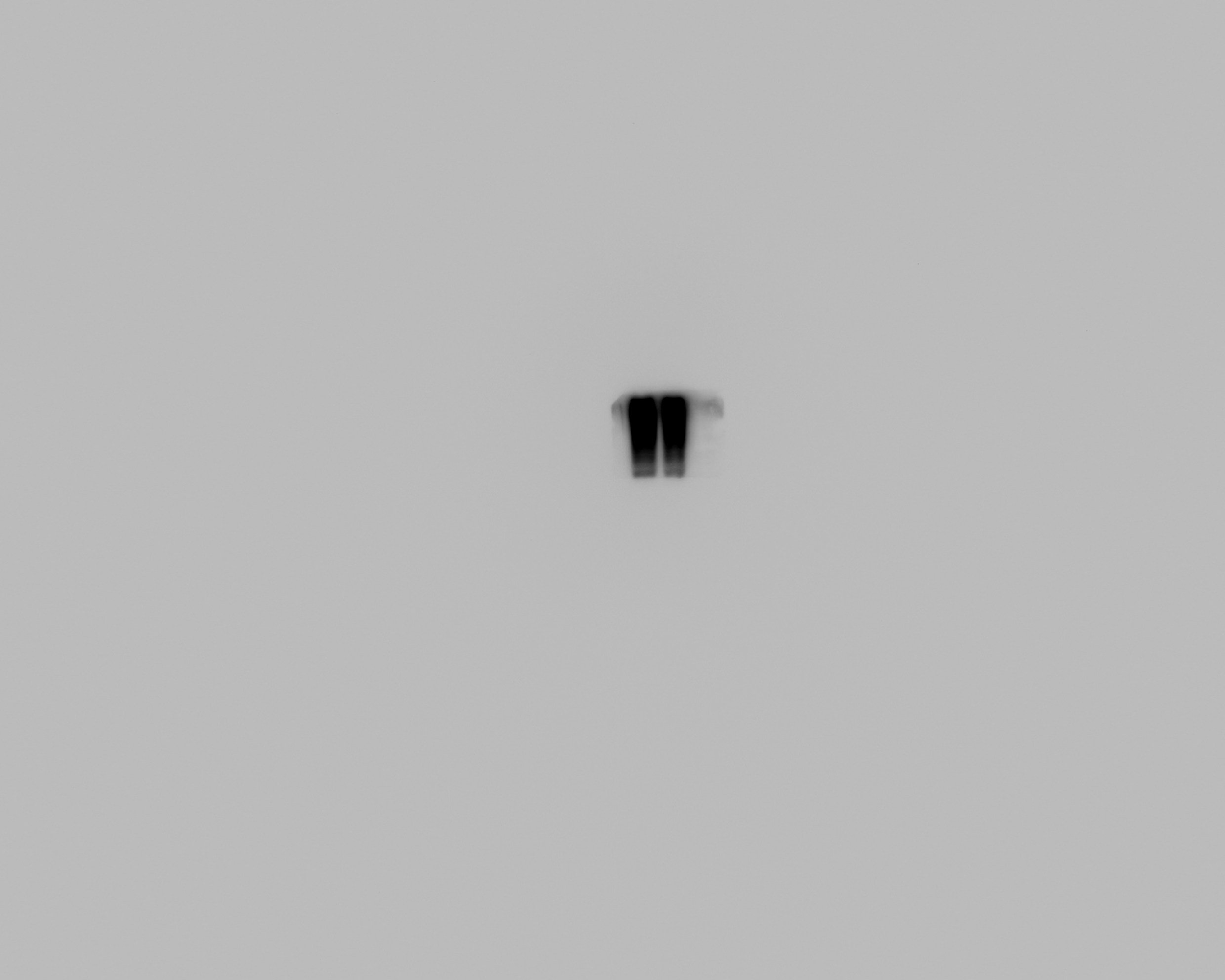

Supplement: Supplementary file 7 — Additional file 7. [file 12964_2024_1475_MOESM7_ESM.zip › Additional file 2/Figure 3I/TE-1/input ubiquitin.tif]

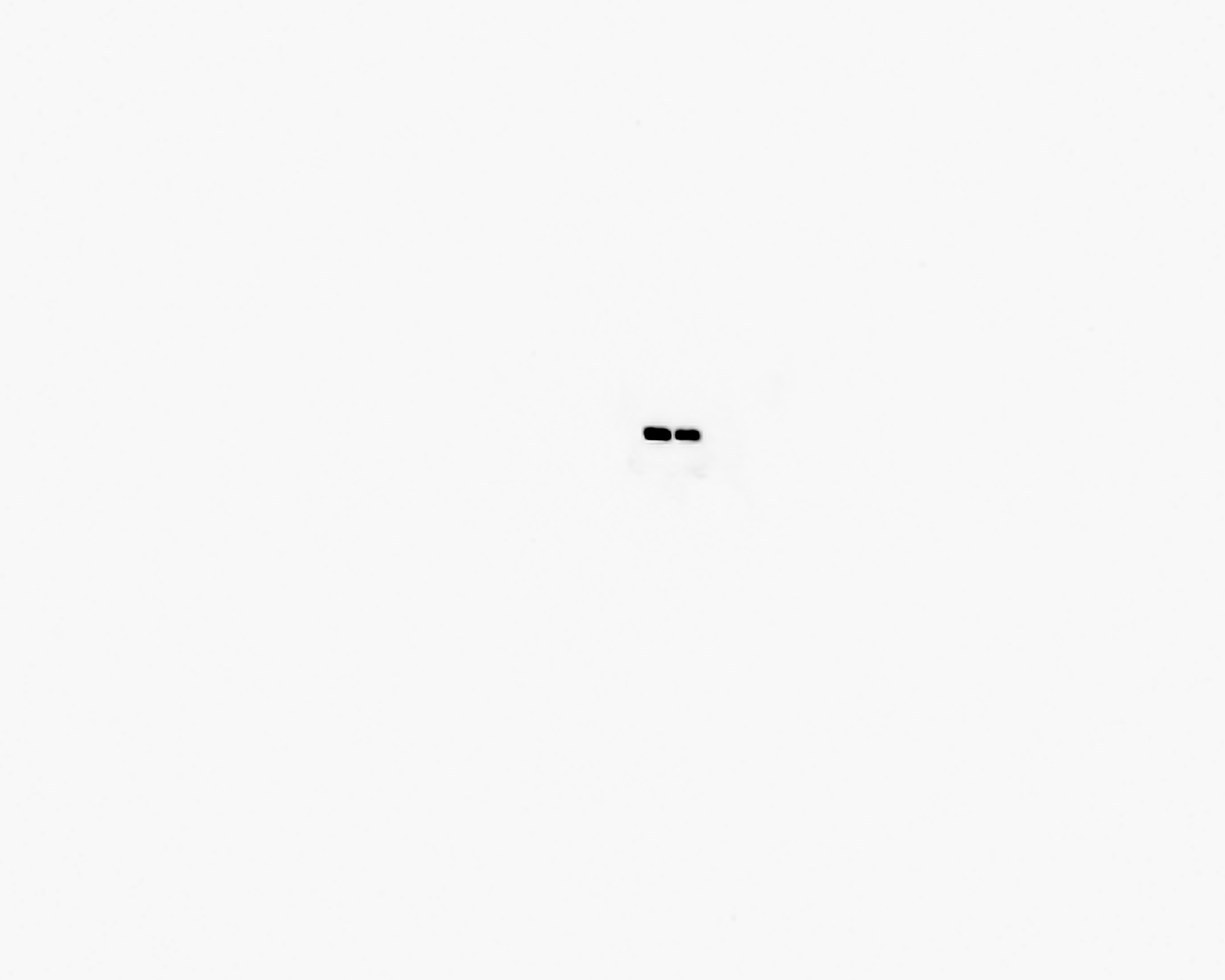

Supplement: Supplementary file 7 — Additional file 7. [file 12964_2024_1475_MOESM7_ESM.zip › Additional file 2/Figure 3I/TE-1/ip oct4.tif]

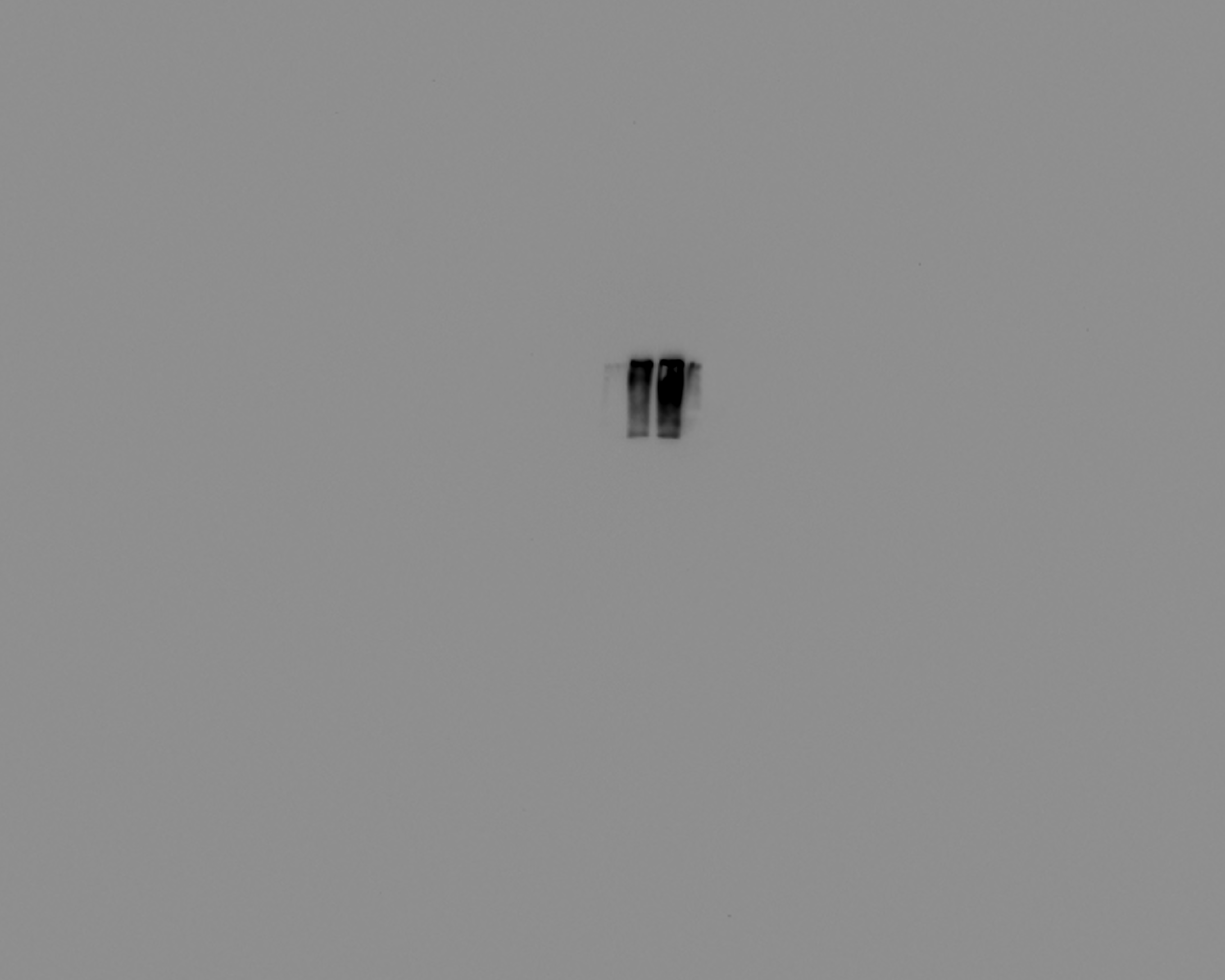

Supplement: Supplementary file 7 — Additional file 7. [file 12964_2024_1475_MOESM7_ESM.zip › Additional file 2/Figure 3I/TE-1/ip ubiquitin.tif]

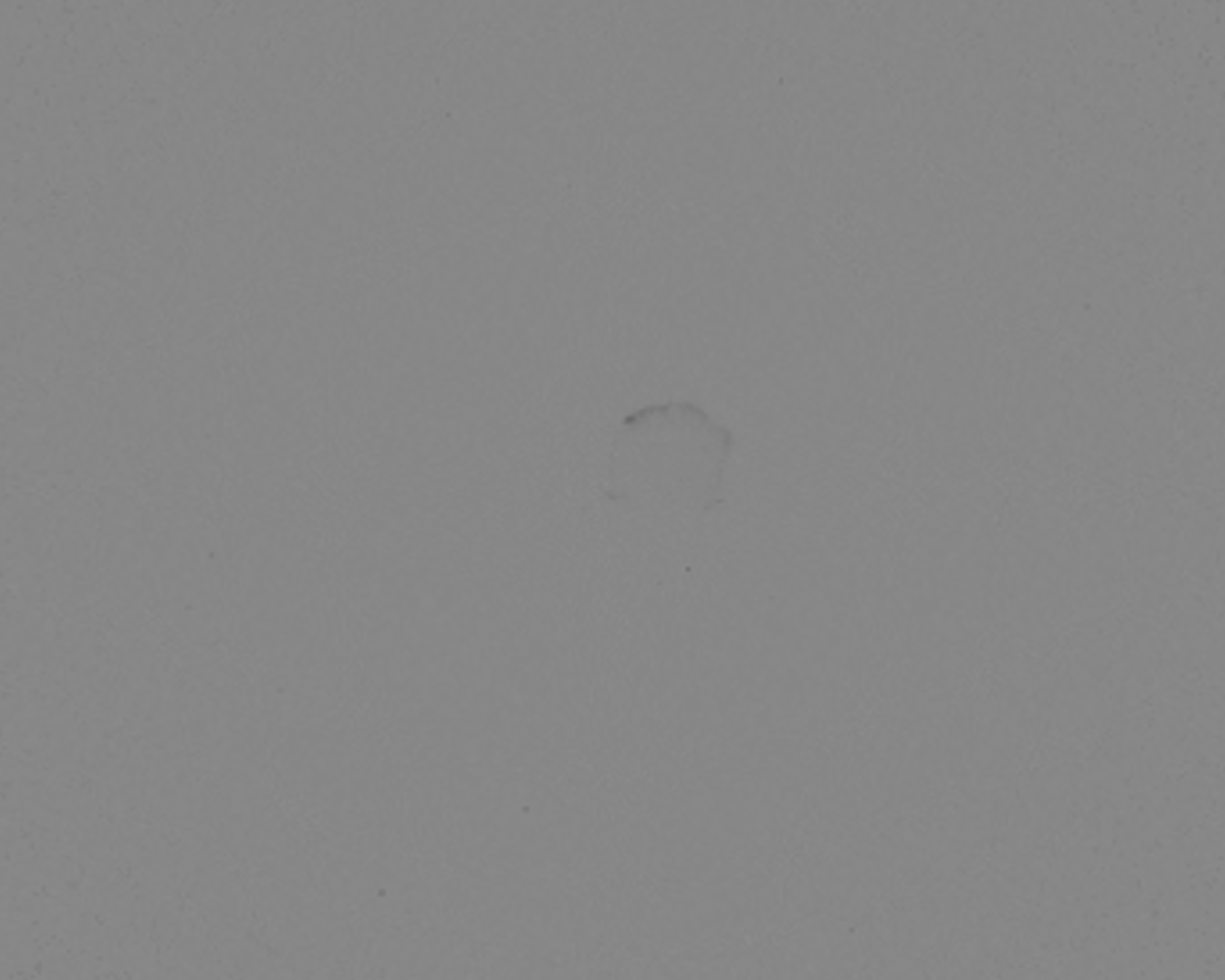

Supplement: Supplementary file 7 — Additional file 7. [file 12964_2024_1475_MOESM7_ESM.zip › Additional file 2/Figure 3J/KYSE-150/IgG oct4.tif]

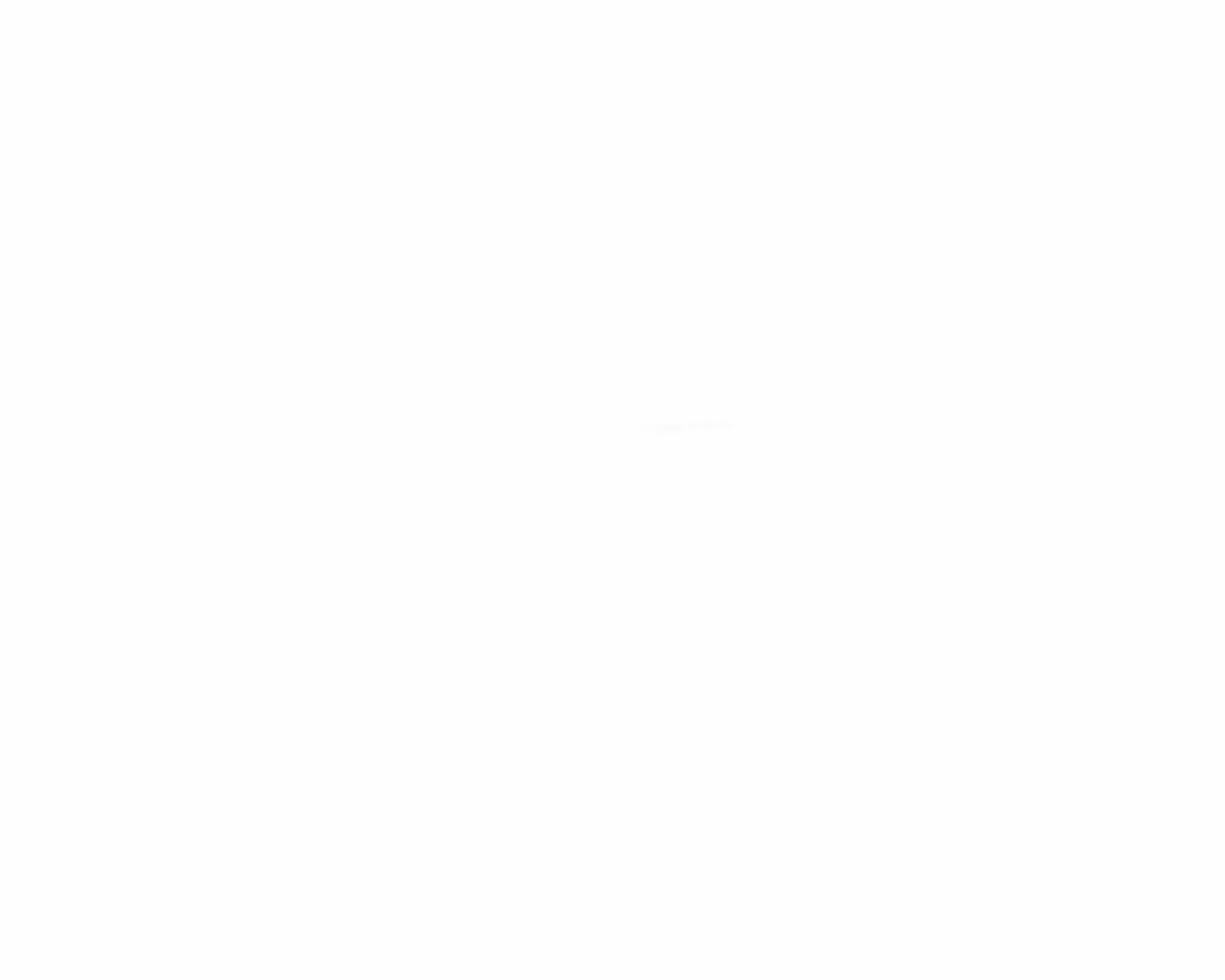

Supplement: Supplementary file 7 — Additional file 7. [file 12964_2024_1475_MOESM7_ESM.zip › Additional file 2/Figure 3J/KYSE-150/IgG ubiquitin.tif]

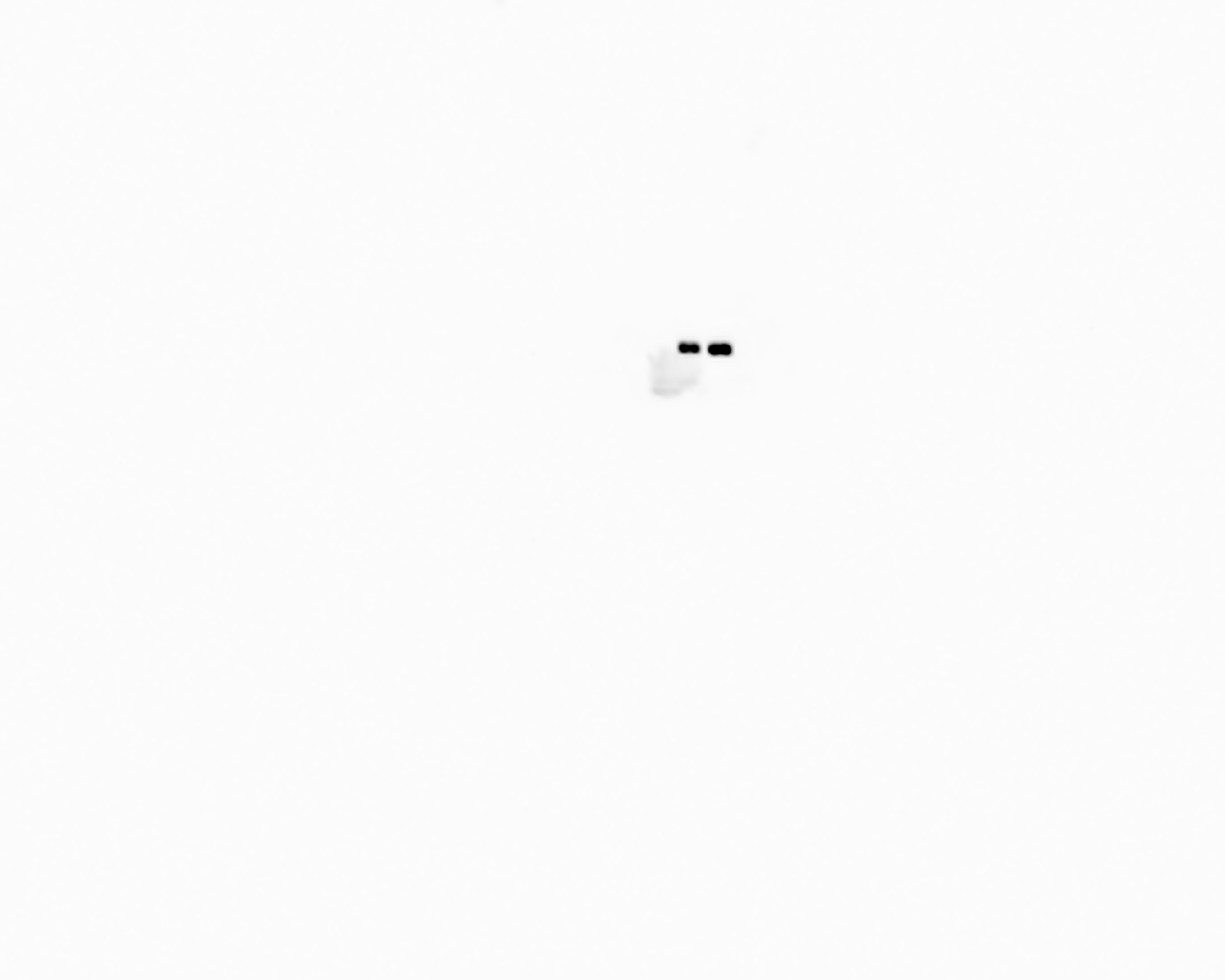

Supplement: Supplementary file 7 — Additional file 7. [file 12964_2024_1475_MOESM7_ESM.zip › Additional file 2/Figure 3J/KYSE-150/input oct4.tif]

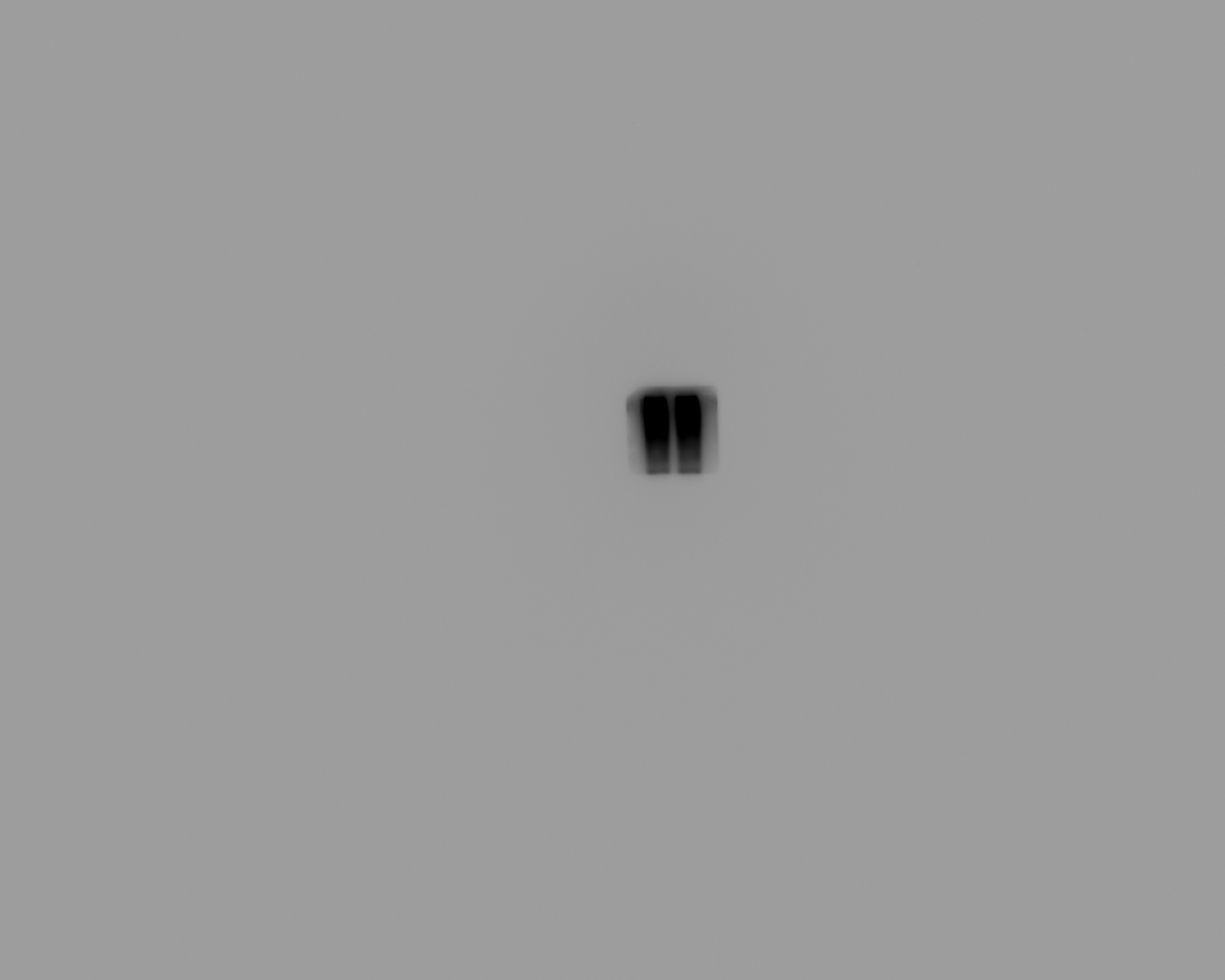

Supplement: Supplementary file 7 — Additional file 7. [file 12964_2024_1475_MOESM7_ESM.zip › Additional file 2/Figure 3J/KYSE-150/input ubiquitin.tif]

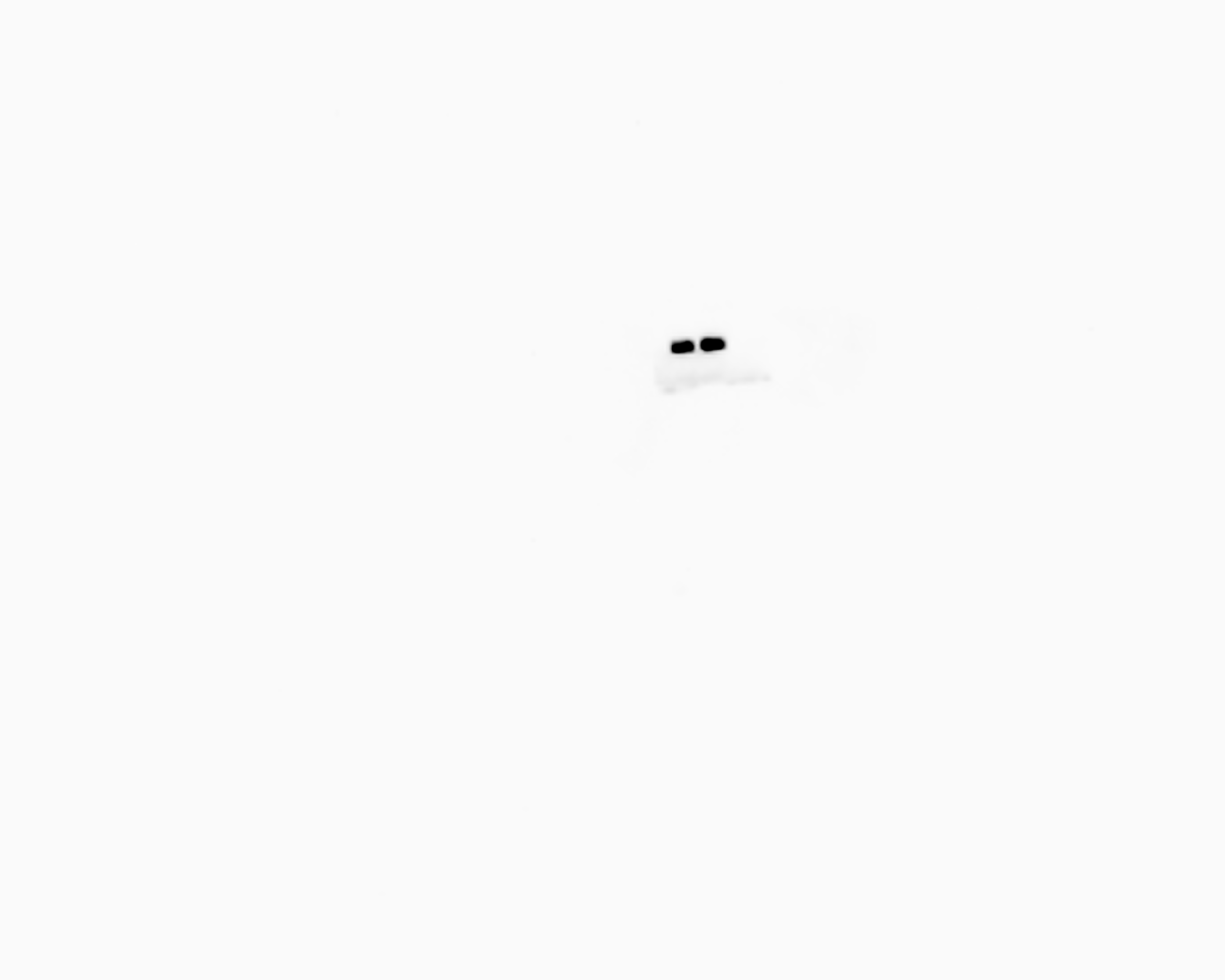

Supplement: Supplementary file 7 — Additional file 7. [file 12964_2024_1475_MOESM7_ESM.zip › Additional file 2/Figure 3J/KYSE-150/ip oct4.tif]

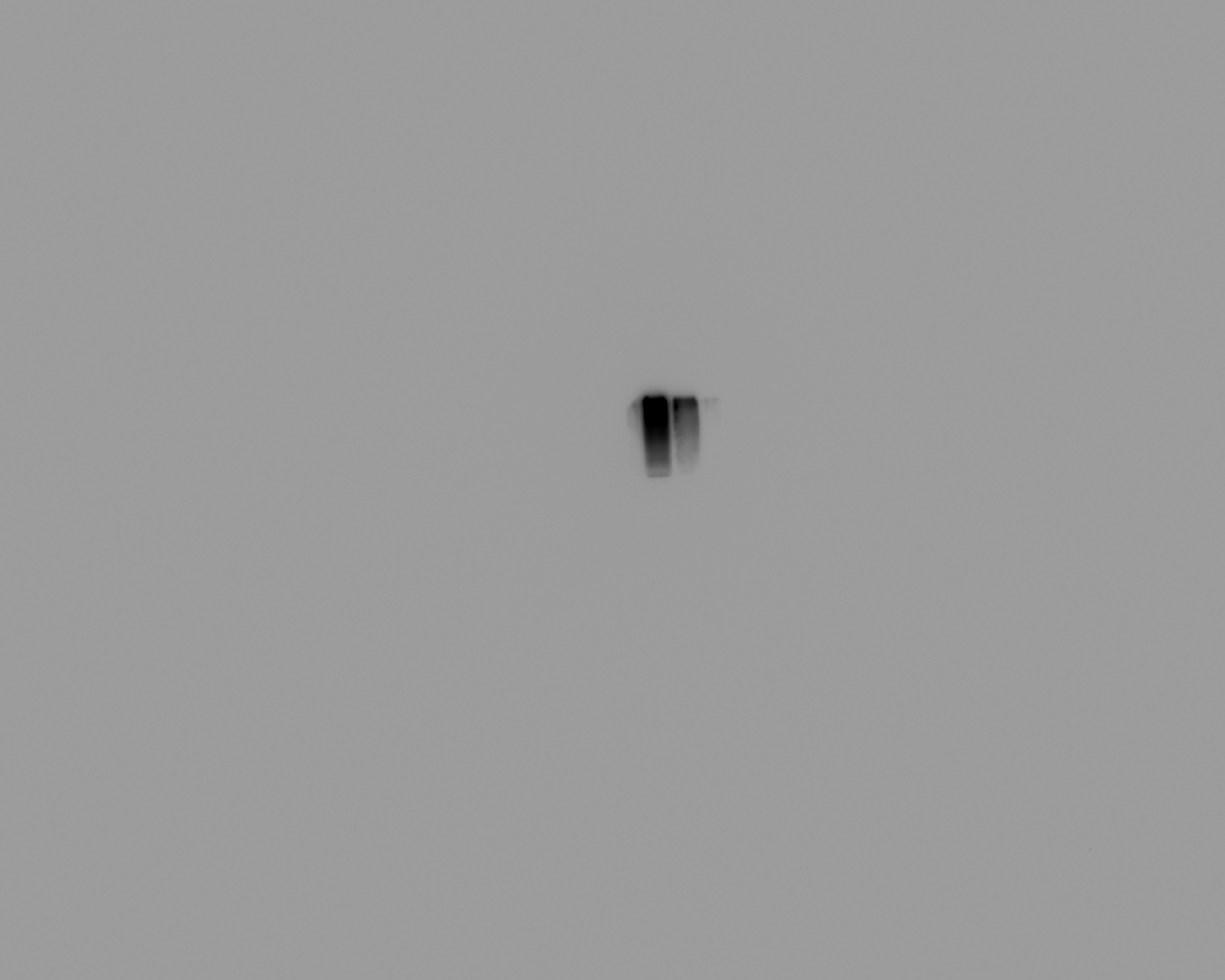

Supplement: Supplementary file 7 — Additional file 7. [file 12964_2024_1475_MOESM7_ESM.zip › Additional file 2/Figure 3J/KYSE-150/ip ubiquitin.tif]

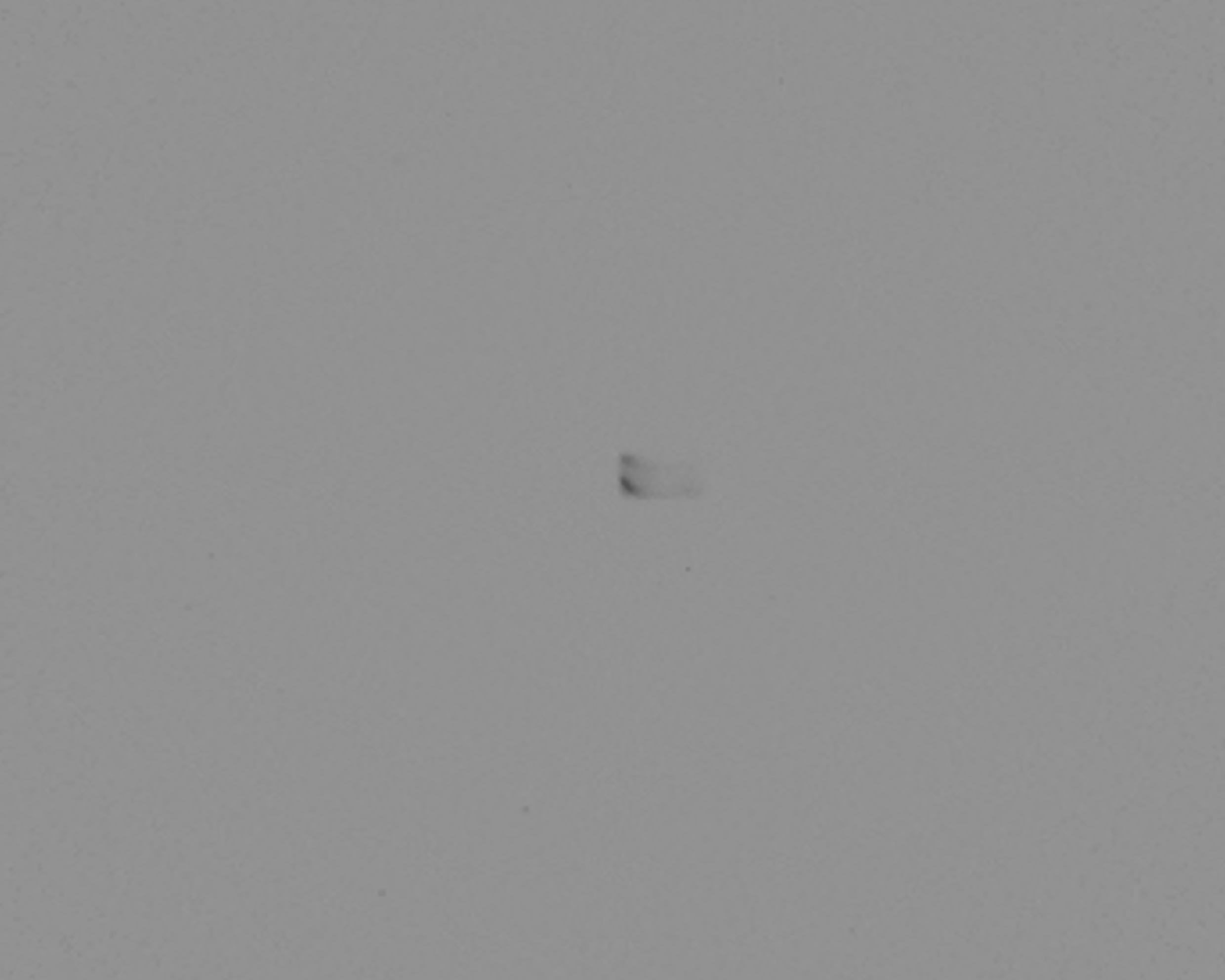

Supplement: Supplementary file 7 — Additional file 7. [file 12964_2024_1475_MOESM7_ESM.zip › Additional file 2/Figure 3J/KYSE-30/IgG oct4.tif]

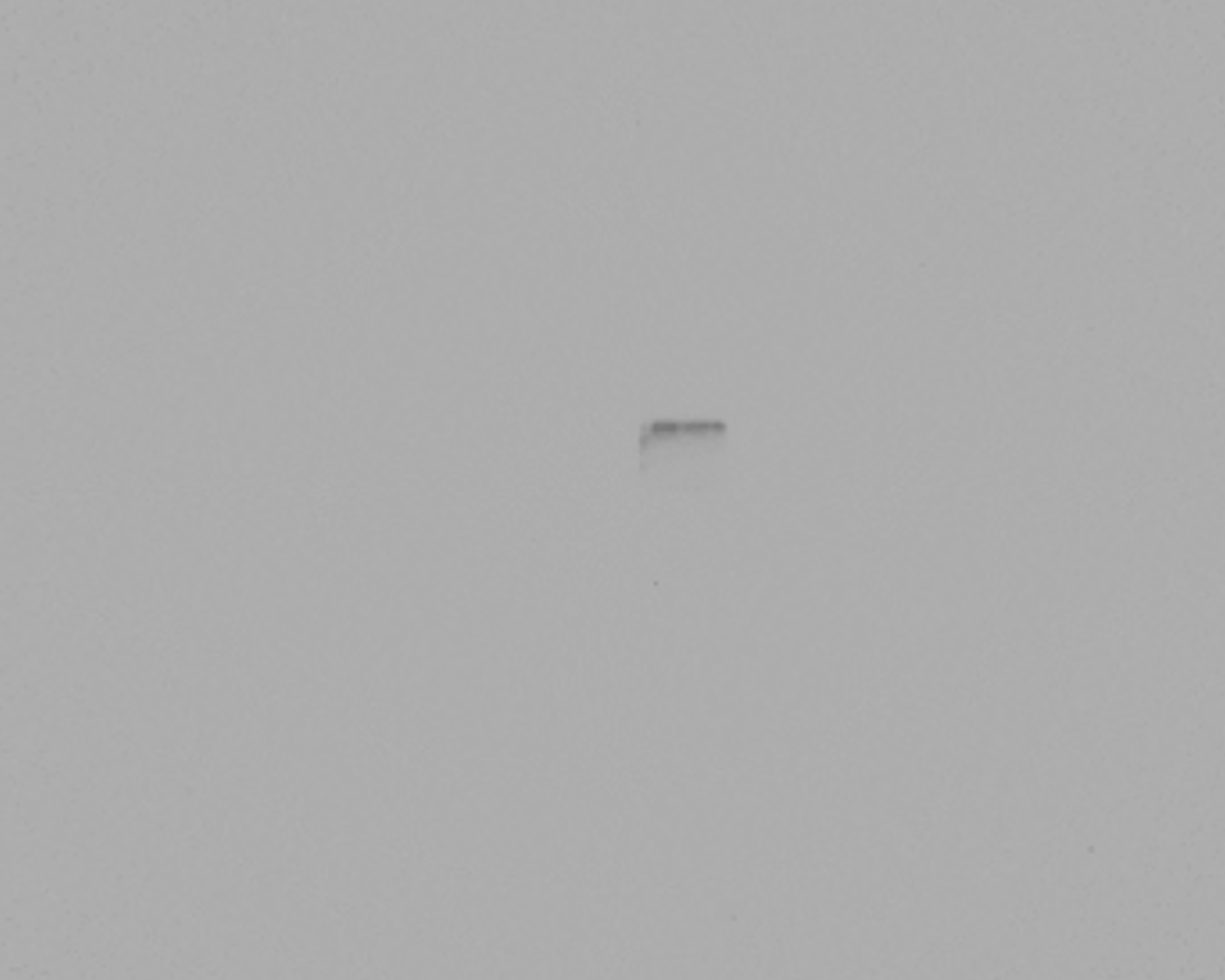

Supplement: Supplementary file 7 — Additional file 7. [file 12964_2024_1475_MOESM7_ESM.zip › Additional file 2/Figure 3J/KYSE-30/IgG ubiquitin.tif]

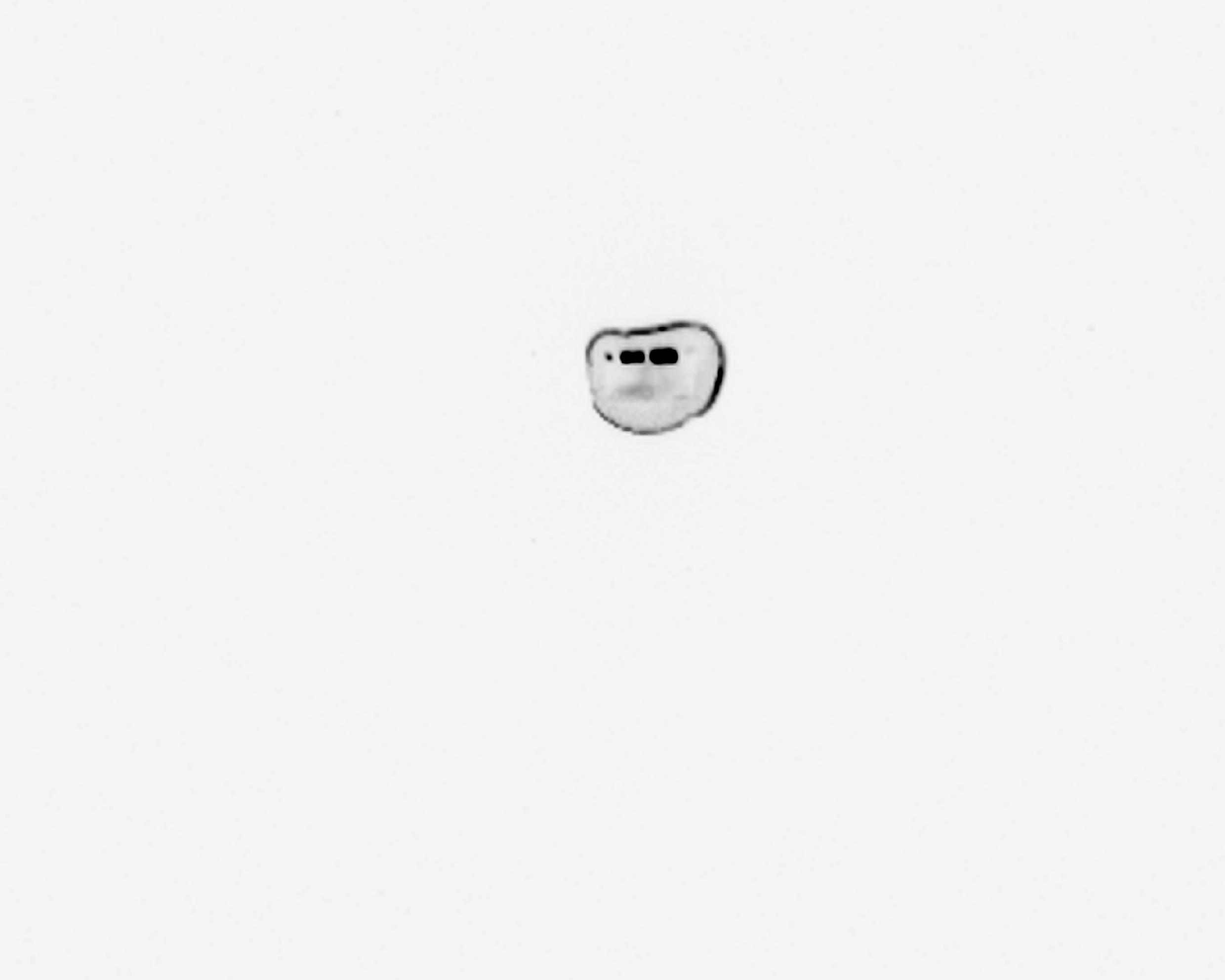

Supplement: Supplementary file 7 — Additional file 7. [file 12964_2024_1475_MOESM7_ESM.zip › Additional file 2/Figure 3J/KYSE-30/input oct4.tif]

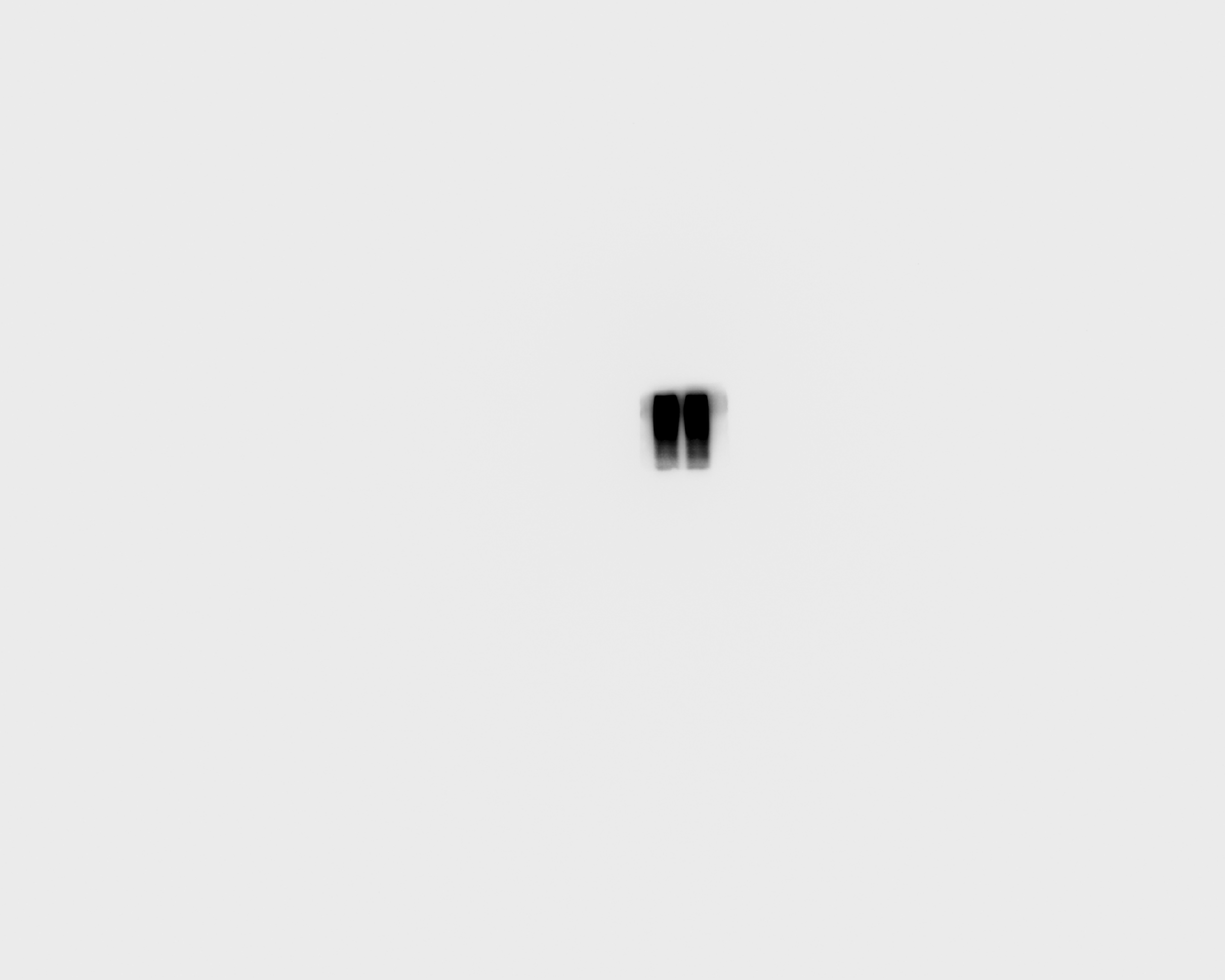

Supplement: Supplementary file 7 — Additional file 7. [file 12964_2024_1475_MOESM7_ESM.zip › Additional file 2/Figure 3J/KYSE-30/input ubiquitin.tif]

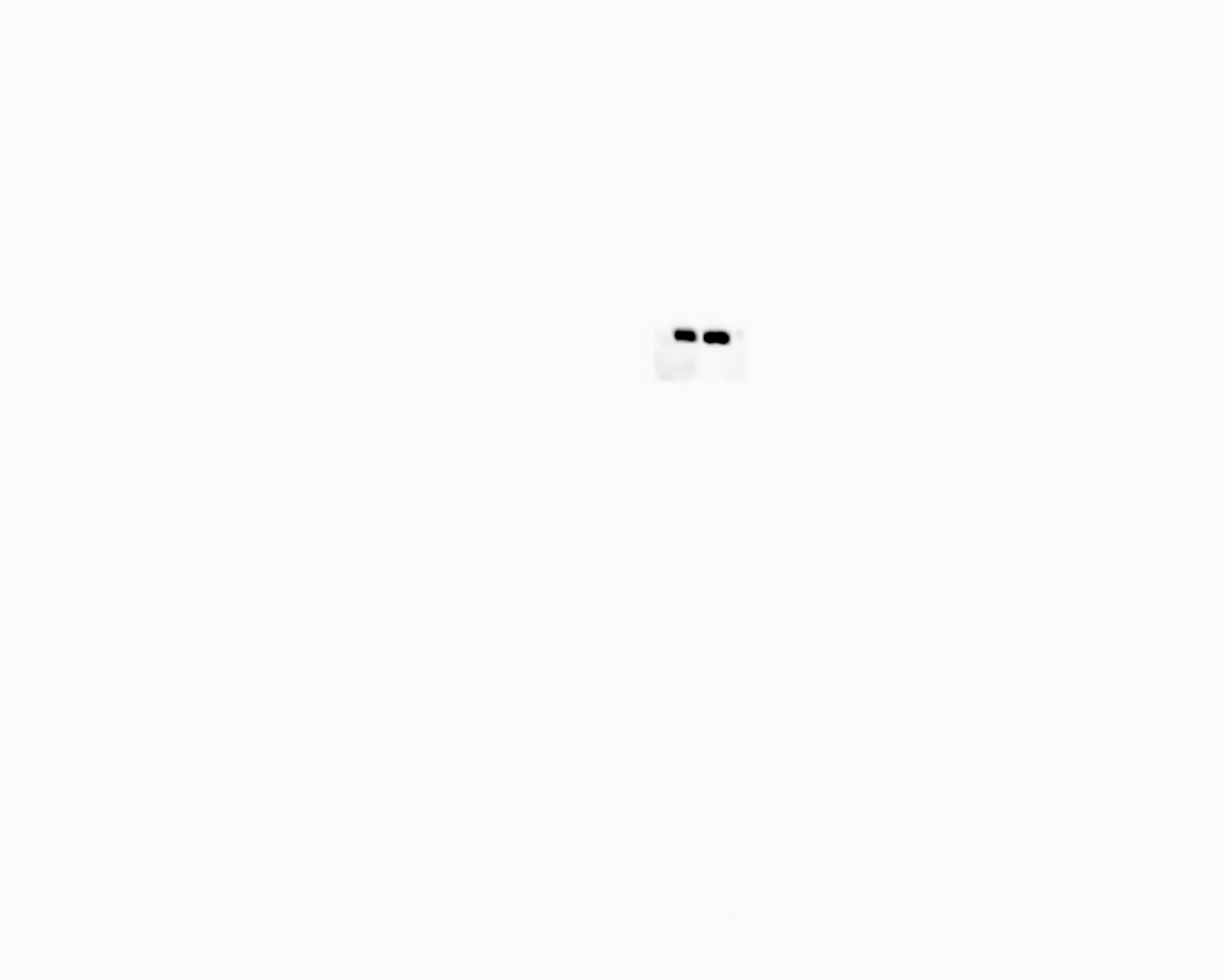

Supplement: Supplementary file 7 — Additional file 7. [file 12964_2024_1475_MOESM7_ESM.zip › Additional file 2/Figure 3J/KYSE-30/ip oct4.tif]

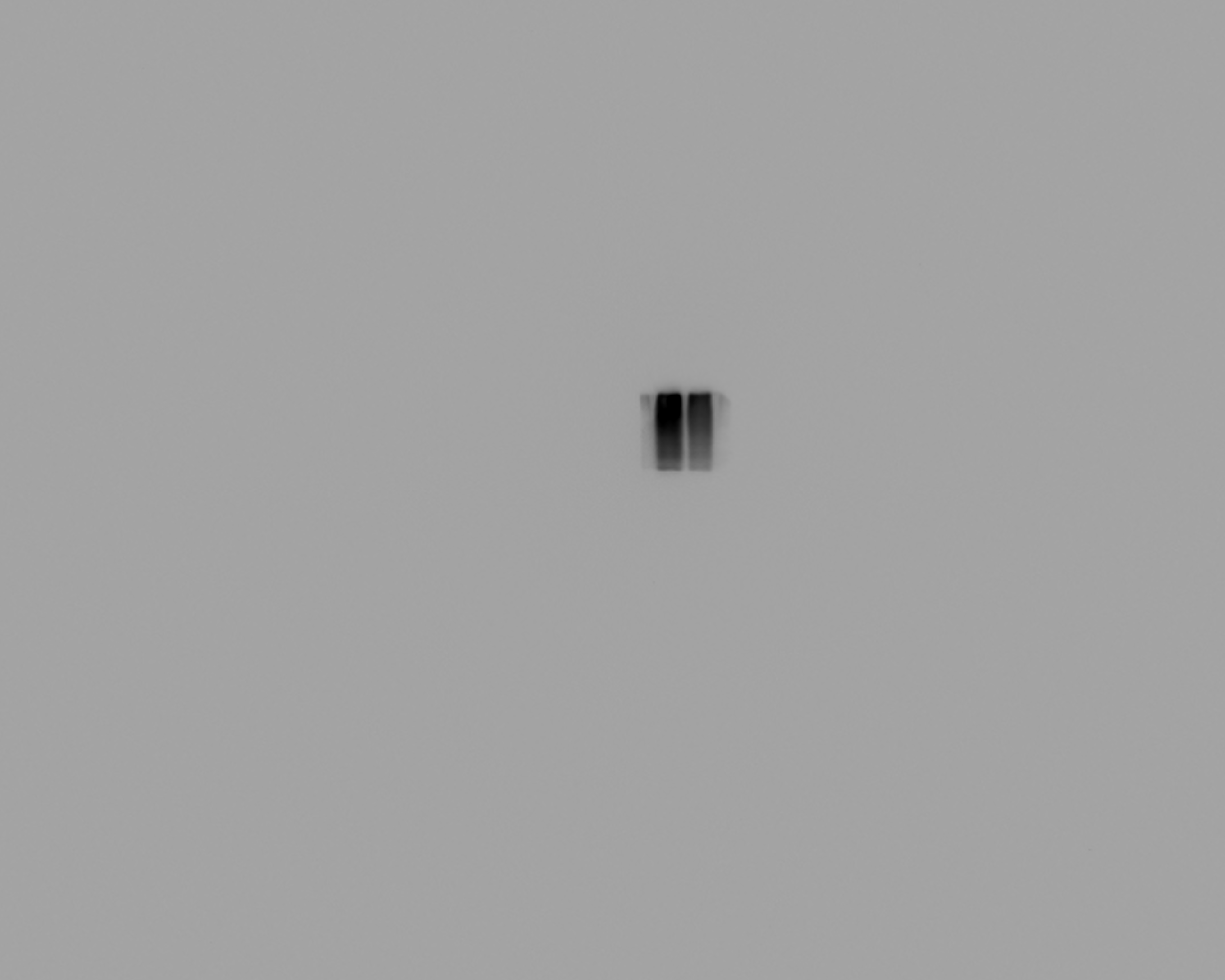

Supplement: Supplementary file 7 — Additional file 7. [file 12964_2024_1475_MOESM7_ESM.zip › Additional file 2/Figure 3J/KYSE-30/ip ubiquitin.tif]

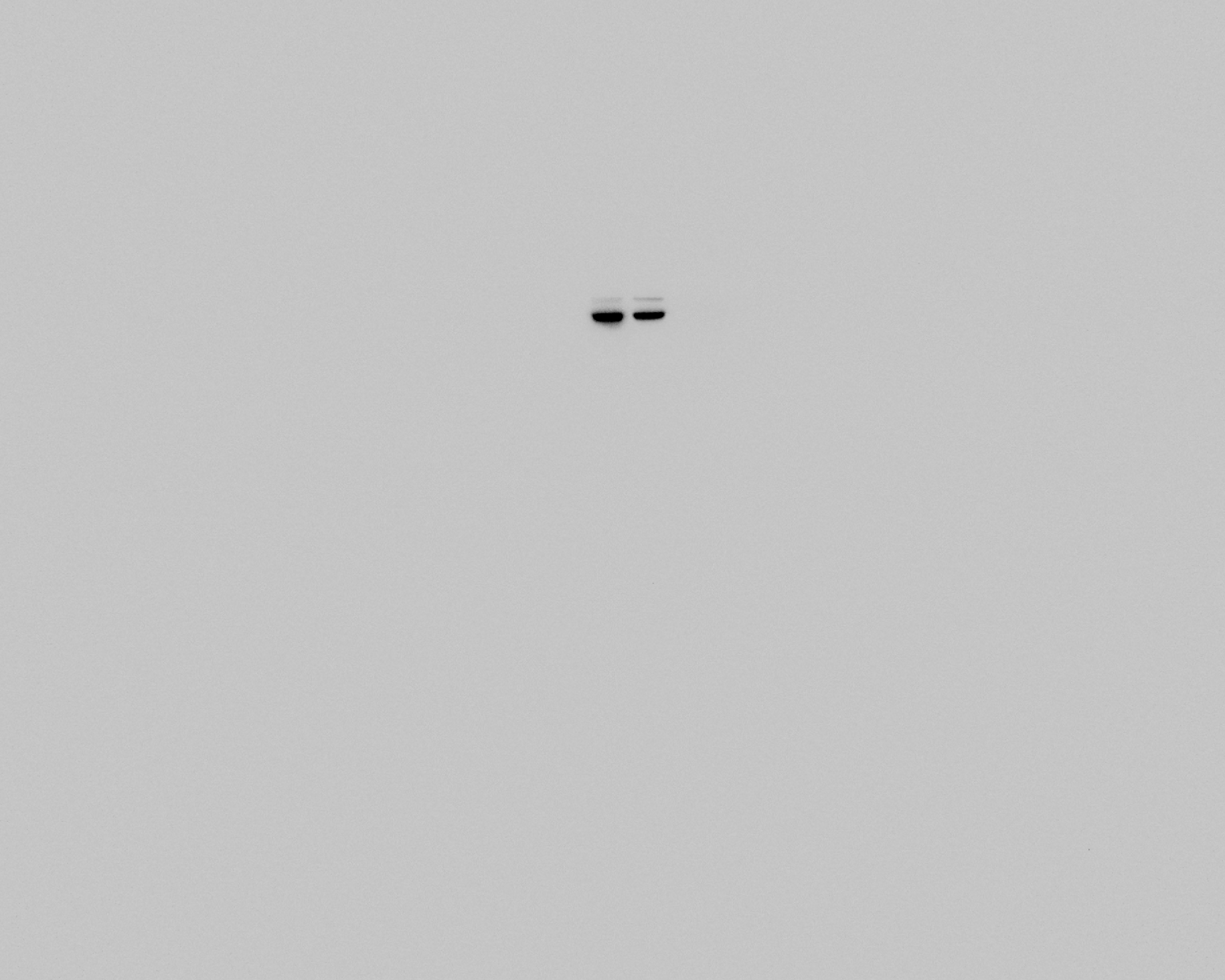

Supplement: Supplementary file 7 — Additional file 7. [file 12964_2024_1475_MOESM7_ESM.zip › Additional file 2/Figure 4F/Eca-109/oct4.tif]

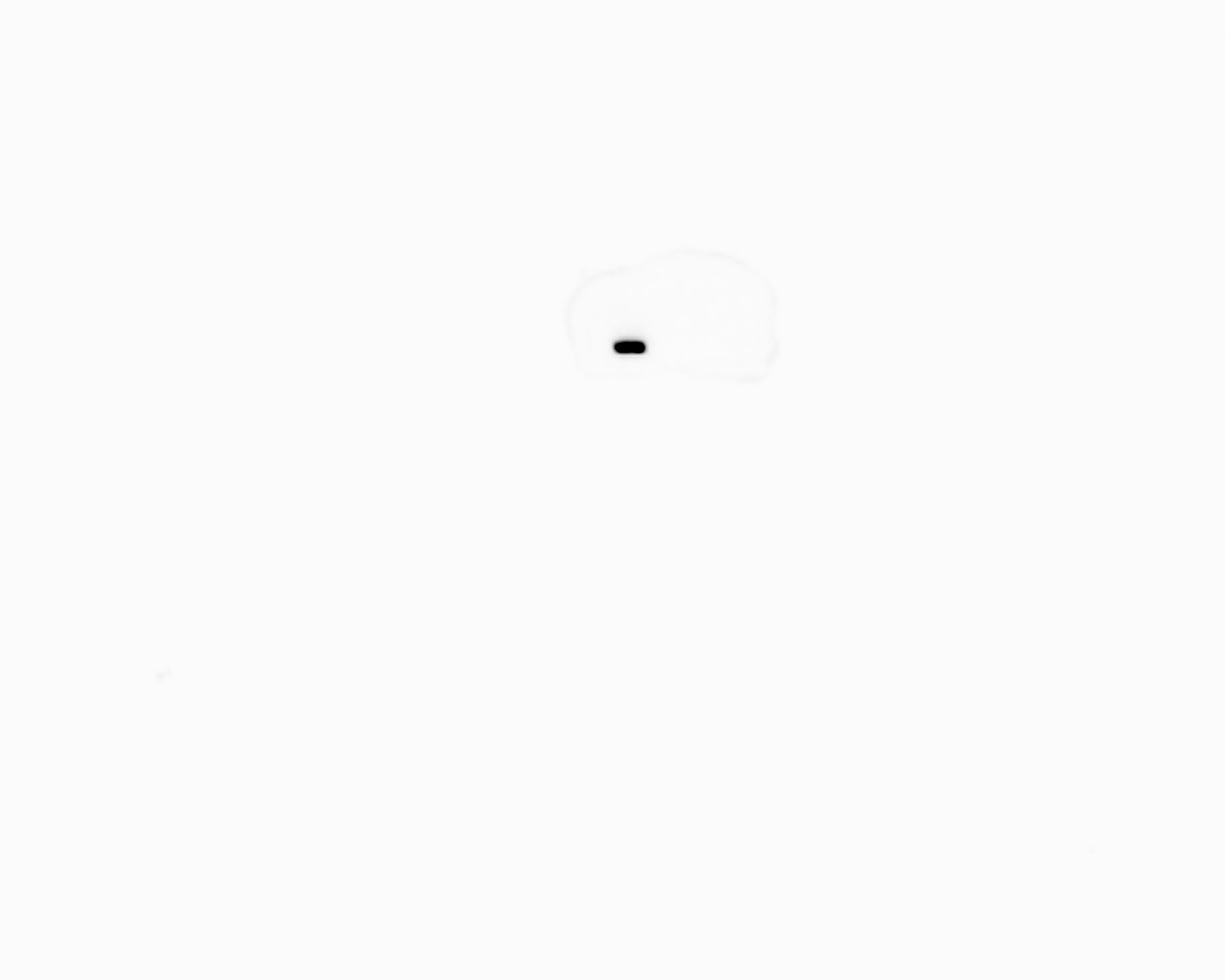

Supplement: Supplementary file 7 — Additional file 7. [file 12964_2024_1475_MOESM7_ESM.zip › Additional file 2/Figure 4F/Eca-109/a┬-actin.tif]
